# Draft Genome Sequence of a New *Fusarium* Isolate Belonging to *Fusarium tricinctum* Species Complex Collected From Hazelnut in Central Italy

**DOI:** 10.3389/fpls.2021.788584

**Published:** 2021-12-16

**Authors:** Silvia Turco, Alessandro Grottoli, Mounira Inas Drais, Carlo De Spirito, Luigi Faino, Massimo Reverberi, Valerio Cristofori, Angelo Mazzaglia

**Affiliations:** ^1^Dipartimento di Scienze Agrarie e Forestali, Università degli Studi della Tuscia, Viterbo, Italy; ^2^Consiglio per la Ricerca in Agricoltura e l’Analisi dell’Economia Agraria, Centro di Ricerca Difesa e Certificazione (CREA-DC), Rome, Italy; ^3^Dipartimento di Biologia Ambientale, Sapienza Università di Roma, Rome, Italy

**Keywords:** nut gray necrosis (NGN), *Fusarium tricinctum* species complex, hazelnut (*Corylus avellana* L.), genomics, hybrid assembly

## Abstract

In summer 2019, during a survey on the health status of a hazelnut orchard located in the Tuscia area (the province of Viterbo, Latium, Italy), nuts showing symptoms, such as brown-grayish spots at the bottom of the nuts progressing upward to the apex, and necrotic patches on the bracts and, sometimes, on the petioles, were found and collected for further studies. This syndrome is associated with the nut gray necrosis (NGN), whose main causal agent is *Fusarium lateritium*. Aiming to increase knowledge about this fungal pathogen, the whole-genome sequencing of a strain isolated from symptomatic hazelnut was performed using long Nanopore reads technology in combination with the higher precision of the Illumina reads, generating a high-quality genome assembly. The following phylogenetic and comparative genomics analysis suggested that this isolate is caused by the *F. tricinctum* species complex rather than *F. lateritium* one, as initially hypothesized. Thus, this study demonstrates that different *Fusarium* species can infect *Corylus avellana* producing the same symptomatology. In addition, it sheds light onto the genetic features of the pathogen in subject, clarifying facets about its biology, epidemiology, infection mechanisms, and host spectrum, with the future objective to develop specific and efficient control strategies.

## Introduction

*Corylus avellana* L. (hazelnut) is a shrub species belonging to the Betulaceae family. Italy is the second largest hazelnut producer in the world, with an average production of about 140,000 t/year spread among four regions (Campania, Latium, Sicily, and Piedmont), behind Turkey (ISTAT^1^, FAOstat Agriculture Data^2^). In the last decades, a fruit rot causing considerable yield losses has been observed and described as a new disease. The symptomatic fruits were characterized by the presence of brown-grayish spots at the bottom of the nuts progressing upward to the apex, and necrotic patches on the bracts and, less often, on the petioles (Belisario and Santori, 2009). Based on these symptoms, the disease has been named nut gray necrosis (NGN) and associated with *Fusarium lateritium* Nees [*Gibberella baccata* (Wallr.) Sacc.] as its causal agent (Santori et al., 2010; Vitale et al., 2011). *Fusarium* is a large cosmopolitan genus of filamentous ascomycetes fungi, ranked as one of the most economically destructive and species-rich groups in the world, including plant pathogens, saprophytes, and endophytes species, among others (O’Donnell et al., 2013, 2015). Among the numerous species, *F. lateritium* has been reported on numerous hosts, including woody fruit trees as well as shrubs and herbaceous plants, where it could induce wilting, tip or branch dieback, and cankers. *F. lateritium* has also been reported as the causal agent of twig canker on hazelnut, and fruit rot on walnut (Wollenweber, 1931) and olive (Elia, 1964). Several pathogenicity tests were conducted, supporting the involvement of this fungus in the NGN disease and twig canker of hazelnut (Belisario and Santori, 2009).

In late summer 2019, a survey on the health status of a hazelnut orchard located in the Tuscia area (the province of Viterbo, Latium, Italy) was carried out in order to combine an agronomic evaluation of the state of the field approach with a Supervisory Control and Data Acquisition (SCADA) system for the precision farming of orchards. Nuts showing NGN symptoms were found and collected for further laboratory analysis and molecular characterization.

Aiming to increase knowledge about this fungal pathogen, the genome of one fungal strain isolated from a typical NGN diseased nut was sequenced using both long- and short-reads sequencing technologies. The resulting genome when compared with other available *Fusarium* genomes showed that, despite a morphological similarity with *F. lateritium*, this isolate is related to the *F. tricinctum* species complex rather than to the *F. lateritium* one. Thus, this study aims to provide new insights about the complexity of *Fusarium* species that infect tissues and fruits of hazelnut and to better understand the genetics behind the pathogenic mechanisms of this fungal strain. The new information achieved represents the basis for a better focused and effective control strategy of this disease.

## Materials and Methods

### Fungal Isolation

Symptomatic nuts (Tonda Gentile Romana cv) were collected from a mature hazelnut orchard located in the Viterbo area (VT) (Latium, Italy; latitude 42°16′00.0″, longitude 12°17′00.0″, altitude 275 m). Small fragments of approximately 2 mm^2^ were taken from the nut surface and placed onto potato dextrose agar (PDA) plates supplemented with 0.2 g⋅L^–1^ streptomycin sulfate. The Petri plates were then incubated at 25°C until the fungal colonies were grown enough to be singularly further transferred onto new PDA plates to finally obtain monosporal isolates.

### Morphological and Molecular Identification

For morphological characterization, the cultures were grown on Spezieller Nahrstoffarmer agar (SNA) (Leslie and Summerell, 2006). After 10 days, the cultures were examined using a Nikon SMZ128 stereomicroscope (Tokyo, Japan), and the images were captured using Alexasoft TPS5000H CMOS camera (Florence, Italy). For microscopic analysis, the samples were prepared by mixing with lactophenol blue dye using Leica DM6 B optical microscope (Wetzlar, Germany), the images were captured using Leica DFC 7000 T camera (Wetzlar, Germany), elaborated using Leica Application Suite X program (version 4.12) (Wetzlar, Germany), and all the morphological characteristics were evaluated according to *The Fusarium Laboratory Manual* (Leslie and Summerell, 2006).

For each isolate, about 100 mg of mycelium was put into a sterile 2 ml microtube with 1 ml of lysis buffer (1 mM EDTA pH 8, 10 mM Tris–HCl pH 8, 100 mM NaCl, SDS 1%) for DNA extraction. After 5 min of centrifugation at 12,000 rpm, the supernatant was transferred into a new microtube, and the DNA was precipitated with isopropanol and 70% ethanol. To characterize the isolates at the *genus*/*species* level, two genes were amplified using two different pairs of primers, one targeting the internal transcribed spacer (ITS) (White et al., 1990) and the second one targeting the *Fusarium* specific translation elongation factor 1-alpha (Edel-Hermann et al., 2015), and the amplicons were Sanger sequenced at Eurofins genomics (Eurofins Genomics GmbH, Konstanz, Germany).

### Pathogenicity Tests

To confirm that the isolate in subject was responsible for the observed symptoms on fruits, the following pathogenicity tests were carried out. For inoculum preparation, the PT isolate of the *Fusarium* sp. was grown on SNA at 25°C for 10 days (Leslie and Summerell, 2006). Then, conidia were scraped from the fungal cultures and filtered through layers of cheesecloth to remove mycelial fragments. The resulting conidial suspension was quantified using a hemocytometer and diluted to a final concentration of 10^6^ spore/ml. Inoculations were performed using two techniques: (i) inoculation of young to fully formed fruits with 20 μl of conidial suspension (10^6^ conidia/ml) dropped between nut and bracts; and (ii) inoculation of the hazelnut mesocarp with 20 μl of conidial suspension (10^6^ conidia/ml). Control fruits were inoculated with water. Inoculated and control fruits were placed in a moist chamber at room temperature for 12 days. The appearance of any symptoms was monitored daily.

### High Molecular Weight DNA Extraction and Genome Sequencing

The fungal culture was initiated using ∼1 × 10^6^ conidia inoculated in 100 ml of Czapek Dox Yeast broth (Leslie and Summerell, 2006) and grown for 3 days at 25°C. Next, the genomic DNA was extracted following a modified Cetrimioum bromide (CTAB) method (Graham and Henry, 1997) and performed as described below.

The total mycelium grown in liquid media was lyophilized using a vacuum pump. An aliquot of 250 mg of lyophilized mycelium was fine grounded using a pestle and mortar in liquid nitrogen and transferred into a 2 ml tube. Then, 500 μl of CTAB buffer (10% CTAB, 25 mM EDTA pH 8.0, 200 mM Tris–HCl pH 8.0, 2.50 M NaCl) and 5 μl proteinase K (20 mg/ml) were added to the sample and the solution was gently mixed and incubated overnight at 56°C. After 5 min of agitated incubation with 500 μl of phenol:chloroform:isoamyl alcohol (25:24:1) and 10 min of 12,000 rpm centrifugation, the aqueous clear phase was transferred to a new tube and incubated with 500 μl of chloroform:isoamyl alcohol (24:1). After 10 min of centrifugation at 12,000 rpm, the aqueous phase was collected into a new tube and 0.6 VOL of cold isopropanol was added, followed by 30 min of centrifugation at 12,000 rpm for the DNA precipitation. Two cleaning/precipitation steps using 1 ml of 70% cold ethanol were performed by 10 min of centrifugation at 12,000 rpm. The pellet was dried, resuspended into a 100 μl of ultrapure sterile water, and treated with RNase at 37°C for 30 min. The DNA integrity was evaluated on a 1% agarose gel electrophoresis run, whereas the DNA purity was checked using Nanodrop™ spectrophotometer (Thermo Fisher Scientific). DNA sequencing was performed on a MinION Mk1b device (Oxford Nanopore Technologies, ONT, United Kingdom) using a R9.4.1 Flow Cell (ONT), after library preparation using the SQK-RBK004 Rapid Barcoding Kit (ONT). An aliquot of the same DNA sample was sequenced at Eurofins Genomics (Eurofins Genomics GmbH, Konstanz, Germany) with the genome sequencer Illumina NovaSeq 6000 S2 using the paired-end sequencing.

### Genome Assembly

Illumina reads quality was evaluated using FastQC (Andrews, 2010), NovaSeq 6000 adapters were trimmed using Trimmomatic version 0.39 (Bolger et al., 2014), and low-quality reads were removed using Sickle (Joshi and Fass, 2011). To get the draft genome sequence, four different assemblers were used separately: SPAdes version 3.11.1 (Nurk et al., 2013), Minimap2 version 2.12-r849-dirty (Li, 2018) in combination with Miniasm version 0.3-r179 (Li, 2016), MaSuRCA version 3.4.2 (Zimin et al., 2013), and Canu version 2.1.1 (Koren et al., 2017). SPAdes was used in a first trial using only Illumina reads and in a second run for a hybrid assembly using both Illumina and Nanopore reads. A hybrid assembly was also performed using MaSuRCA, whereas Miniasm-Minimap2 and Canu used only Nanopore reads. For all the assemblers, default parameters were used, except for the expected genome size that was set at 40 Mb in Canu.

The obtained assemblies were further polished using Nanopolish version 0.11.1 (Loman et al., 2015), Racon version 1.3.3 (Vaser et al., 2017), or Pilon version 1.23 (Walker et al., 2014). The assembly quality statistics, before and after polishing, were evaluated using QUAST version 5.0.2 (Gurevich et al., 2013), while BUSCO version 5.beta.1 (Simão et al., 2015) was used to assess the assembly completeness, using *hypocreales_db10* as ortholog lineage dataset, which consists of a set of 4,494 conserved profiles.

### Genome Annotation

The draft genome assembled using Canu, chosen for downstream analysis, was structurally annotated following the *de novo* MAKER pipeline version 3.01.03 (Holt and Yandell, 2011), using the built-in RepeatModeler to mask repetitive elements, SNAP and AUGUSTUS for an *ab initio* gene prediction, and Est2Genome and Protein2Genome to further refine introns and exons boundaries using Exonerate and tRNAscan-SE to identify the genes related to the tRNA biosynthesis. Transcripts and proteins concatenated from four closely related *Fusarium* species (*F. fujikuroi*, *F. graminearum*, *F. oxysporum* f. sp. *lycopersici*, and *F. verticillioides*) were given as gene models, and the maximum intron size was set as 2,500. Functional annotation was performed using BLASTp and SwissProt as a database.

The same annotation pipeline was applied to annotate the genomes of *F. culmorum, F. circinatum, F. oxysporum* f. sp. *koae 44*, *F. pseudograminearum* CS3270, *F. solani* IlSc-1, *F. tricinctum* INRA104, *F. tricinctum* NRRL25481, *F. tricinctum* T6, *F. verticilloides* BRIP 53263, BRIP 53590, and all the *F. avenaceum* isolates, except for the already annotated Fa05001 used for the following comparative genomics analysis.

### Comparative Genomics and Phylogenetic Analysis

The genome sequence of 27 others *Fusarium* species, together with their annotated proteins when available, were downloaded from the NCBI genome databases and used for phylogenetic and comparative genomics analysis (Table 1). Notably, the only *F. lateritium* genome sequence available in the NCBI database under the accession number GCA_014898835.1 refers to a strain isolated from a symptomatic elm tree in Pineville, Louisiana, United States (Kim et al., 2020).

**TABLE 1:** List of *Fusarium* species used for pairwise genome comparisons and phylogenetic analysis.

Species	Strain	Accession number
*Fusarium acuminatum*	F829	GCA_013363215.1
*Fusarium avenaceum*	F156N33	GCA_018282135.1
*Fusarium avenaceum*	Fa05001	GCA_000769215.1
*Fusarium avenaceum*	FaLH03	GCA_000769305.1
*Fusarium avenaceum*	FaLH27	GCA_000769295.1
*Fusarium avenaceum*	NRRL 13321	GCA_013753855.1
*Fusarium avenaceum*	S18/60	GCA_019055295.1
*Fusarium avenaceum*	S18/70	GCA_019055285.1
*Fusarium avenaceum*	S18/74	GCA_019055275.1
*Fusarium circinatum*	FSP34	GCA_497325.3
*Fusarium culmorum*	-	GCA_900074845.1
*Fusarium fujikuroi*	Augusto2	GCA_9663095.1
*Fusarium fujikuroi*	CSV1	GCA_9663055.1
*Fusarium graminearum*	-	GCA_900073075.1
*Fusarium graminearum*	PH-1	GCA_900044135.1
*Fusarium lateritium*	NRRL 13622	GCA_14898835.1
*Fusarium oxysporum* f. sp. *koae*	44	GCA_14857105.1
*Fusarium oxysporum* f. sp. *lycopersici*	4287	GCA_149955.2
*Fusarium pseudograminearum*	CS3096	GCA_303195.2
*Fusarium pseudograminearum*	CS3270	GCA_974265.2
*Fusarium solani*	IISc-1	GCA_13168735.1
*Fusarium tricinctum*	INRA104	GCA_900382705.2
*Fusarium tricinctum*	NRRL 25481	GCA_012977725.1
*Fusarium tricinctum*	T6	GCA_003045085.1
*Fusarium venenatum*	A3-5	GCA_900007375.1
*Fusarium verticillioides*	BRIP53263	GCA_3317015.2
*Fusarium verticillioides*	BRIP53590	GCA_3316995.2

For a phylogenetic analysis, the gene sequences of six housekeeping genes (EF-1α, RPB1, RPB2, beta tubulin, ITS, and LSU) were extracted from each genome and concatenated and aligned using MUSCLE (Edgar, 2004). The resulting alignment was used to build a maximum likelihood (ML) tree using raxmlHPC (Stamatakis, 2014) and visualized in a dendrogram using FigTree version 1.4.4^3^. A second ML phylogenetic tree was built using raxmlHPC on the core genome SNPs identified during a pan genome analysis performed using Panseq, with the run mode set to pan, the fragment size at 500 nucleotides, the percentage of identity at 90%, and the core genome threshold to 28 genomes, in order to find out the sequences in common among all the strains (Laing et al., 2010). To better discriminate between isolates belonging to the *F. tricinctum* species complex (FTSC), a phylogenetic ML tree was built on the alignment of the concatenated sequence of the DNA-directed RNA polymerase II largest (RPB1) and second largest subunit (RPB2) nucleotide sequence, as previously applied by O’Donnell et al. (2013); Ponts et al. (2020), and Crous et al. (2021). MUSCLE and RAxML were used as previously described, and the 63 *Fusarium* isolates used in this analysis are reported in Supplementary Table 1.

Orthologous proteins were identified using OrthoFinder, and the results were used to build a species tree, visualized using FigTree as well (Emms and Kelly, 2019). The average nucleotide identity (ANI) analysis was performed using the pyani script and ANIb as algorithm for the alignment (Pritchard et al., 2016).

### Characterization of Transcripts Involved in Pathogenesis

The putative secreted proteins involved in pathogenesis were identified using SignalP version 5.0b with the cutoff set ≥ 0.5 (Almagro Armenteros et al., 2019a). TargetP version 2.0 was used to identify signal peptide (SP), mitochondrial and chloroplast transit peptide (mTP and cTP, respectively), and potential cleavage sites (CS) (Almagro Armenteros et al., 2019b). Prediction of transmembrane proteins was performed using TMHMM version 2.0 (Krogh et al., 2001). Pathogen Host Interactions database (Urban et al., 2019) was used to find the similarity with pathogenicity and virulence-related genes, experimentally tested for roles in pathogenicity. Instead, Carbohydrate-Active Enzyme (CAZy) database (Lombard et al., 2014) was used to identify families of enzymes related to degradation, modification, and creation of glycosidic bonds, focusing on the Cell Wall Digestion Enzymes (CWDE).

The biosynthetic gene clusters (BGCs) were automatically searched and analyzed using AntiSMASH6 (Blin et al., 2021). The presence of the emerging mycotoxin enniatin was verified by blasting the available gene sequences from several *Fusarium* species (EF029060.1, NW_022158785.1, NW_022158526.1, KP000028.1, NC_030995.1, ENA| Z18755, NW_023502434.1, NW_023501408.1, and NW_023501343.1) (Fraeyman et al., 2017; Ponts et al., 2018).

## Results

### Strain Identification

**FIGURE 1:**
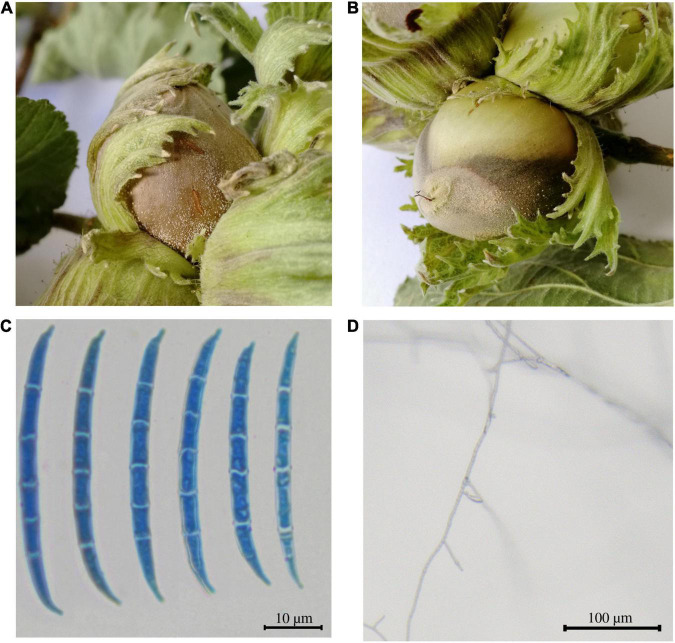
Symptomatic hazelnuts and microscopic characteristics of the *Fusarium* sp. isolate PT. **(A,B)** The symptomatic fruits were characterized by a brown grayish necrotic spot/patch on the nut shell, bracts and less often on the petioles. **(C)** Macroconidia and **(D)** monophiliades of the isolated fungus visualized through optical microscope.

A *Fusarium* sp. strain was isolated from symptomatic nuts collected from a hazelnut field located in the VT (Italy), which was being monitored in the framework of the PANTHEON project and named as *Fusarium* sp. isolate PT (Figures 1A,B). The morphological analysis performed to look at both macroscopic and microscopic characteristics revealed a mycelia color variable from white in the first 3–4 days to pale orange. Abundant medium-long, thin macroconidia with walls parallel for most of the spore length were observed, in the absence of microconidia and rare monophialides (Figures 1C,D). The characteristics found on the sample resembled the ones from the *F. lateritium* species when compared with *The Fusarium Laboratory Manual* (Leslie and Summerell, 2006). The sequenced ITS and the EF-1α region showed the highest identities (98.22 and 99.69% respectively) with *F. lateritium* sequences when blasted in the NCBI database (Supplementary Table 2).

### Pathogenicity Test

**FIGURE 2:**
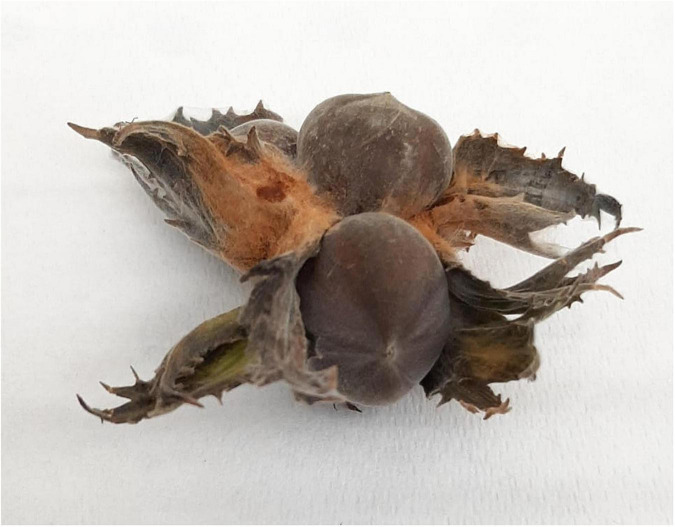
Pathogen reinoculation. The isolated pathogen was reinoculated in healthy hazelnuts, which soon developed the same symptoms as the hazelnut collected in the field close to the Viterbo area (VT), confirming the Kock’s postulate.

All fruits except the negative control developed symptoms similar to the one observed in the field (Figure 2). The *Fusarium* sp. isolate PT was consistently re-isolated from the inoculated nuts but not from the control fruits. The fungus re-isolated from diseased fruits showed the same morphology of the original isolate as well as the ITS, LSU, and β-tub, and EF-1α sequences were identical to those previously obtained, thus confirming Koch’s postulates.

### Genome Assembly and Annotation

A clean and high molecular weight DNA was obtained, with both 260/230 nm and 260/280 nm ratios falling between 1.8 and 2 and was further sequenced using both ONT and Illumina technologies. ONT MinION run produced ∼701 k reads (1.92 Gbp; ∼73 × coverage), with a mean read length of 2,740 bp and an N50 of 4,588. Illumina NovaSeq 6000 S2 sequencing produced 2 × ∼2.5 M reads (2 Mbp × 385 Mbp; ∼20 × coverage).

After low-quality reads and adapters removal done by sickle and trimmomatic, respectively, the total number of the paired-end Illumina reads resulted to be 4.8 M in total, with an average length of 151 bp and a GC content of 47%. Instead, the average length of the Nanopore reads, affected by the starting DNA fragments material, resulted in an average length of 2,740 bp, with a GC content of 47%.

The reads were assembled following several pipelines and, thus, different algorithms whose results are described by QUAST statistics (Table 2). SPAdes draft genome derived either by only Illumina reads or by a hybrid assembly between Illumina and Nanopore reads, resulted to be the one with the lowest N50, the richest in undetermined bases N (171 and 48, respectively) and assembled in the highest number of contigs (70), and thus, it was discarded for further downstream analysis. The genome assembly derived from Miniasm-Minimap2 without any polishing yielded 70 contigs and 40.28 Mb of genome length with an N50 of 1.028 Mb that slightly increased to 1.029 Mb after five iterative polishing steps. MaSuRCA assembly resulted in 53 contigs arranged in a total length of 40.44 Mb and an N50 of 1.49 Mb. One step of Pilon polishing seemed to be unnecessary since the quality did not change much, proving that MaSuRCA itself with its POLCA polishing step is already enough to reach a good quality genome. Overall, Canu assembly resulted to be the one with the best statistics, with a total genome length of 40.51 Mb arranged in only 27 contigs.

**TABLE 2:** Quast assembly statistics.

Assembly	SPAdes	SPAdes hybrid	Minimap2- Miniasm	Minimap2-Miniasm-Pilon	MaSuRCA	MaSuRCA Pilon	Canu	Canu-Pilon
# contigs	407	250	70	70	53	53	27	27
Largest contig	1,476,028	2,349,071	2,564,260	2,565,850	4,680,627	4,680,634	5,683,976	5,693,689
Total length	40,511,781	40,523,229	40,281,042	40,307,966	40,444,187	40,444,622	40,511,598	40,580,457
GC (%)	47.55	47.54	47.56	47.58	47.59	47.59	47.51	47.52
N50	505,135	933,909	1,028,994	1,029,726	1,490,135	1,490,144	2,950,366	2,955,107
N75	289,142	487,994	526,152	526,477	908,446	908,446	1,649,780	1,652,530
L50	26	16	14	14	9	9	5	5
L75	53	30	28	28	17	17	10	10
# N’s per 100 kbp	0.42	0.12	0	0	0	0	0	0

Then, the completeness of the three best quality assemblies was further evaluated by looking for the presence of 4,494 conserved ORF among the Hypocreales order (BUSCO). In fact, the polishing steps were needed to increase the quality and completeness of each of the three assemblies as well as to reduce the fragmented or missing BUSCO, leading to 99.6, 99.8, and 99.5% of completeness, respectively (Supplementary Dataset 1).

Based on the assembly statistics results, the draft genome assembled using Canu was chosen for downstream analysis. Its sequence was further verified by mapping the Illumina reads back on the 27 contigs used as reference to finally reach a high-quality consensus sequence (BWA version 0.7.17-r1188, samtools version 1.2, Burrows and Wheeler, 1994; Li et al., 2009). The genome was then annotated following MAKER pipeline and deposited on the NCBI genome database under the accession number JAHMRZ000000000.

### Phylogenetic Analysis

**FIGURE 3:**
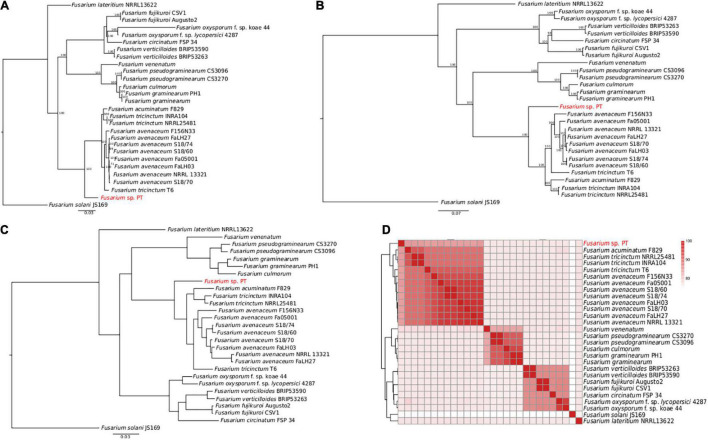
Phylogenetic analysis of 28 *Fusarium* strains. **(A)** The ML phylogenetic tree based on the alignment of the EF-1α, RPB1, RPB2, beta tubulin, ITS, and LSU concatenated nucleotide sequences. Only the bootstraps value higher than 70 are shown. **(B)** The ML phylogenetic tree based on the 4,319 core genome SNPs identified using Panseq. The number of bootstraps is indicated as well. **(C)** The ML phylogenetic tree of the orthologous proteins identified using OrthoFinder. In panels **(A–C)** *Fusarium solani* JS169 was used as outgroup. **(D)** Heatmap of the average nucleotide identity (ANI) performed using blastn showing the percentage of identify among the different *Fusarium* strains.

The aligned concatenated sequences of EF-1α, RPB1, RPB2, beta tubulin, ITS, and LSU (around 7,100 nucleotides) of the *Fusarium* isolates under comparison showed clear differences perfectly represented by the ML tree in Figure 3A. Interestingly, our *Fusarium* sp. isolate PT clustered together with the FTSC and not directly with the other *F. lateritium* strain, as one could expect from the symptoms associated to the NGN. This clustering is due to the low sequence similarity, ranging from 82 to 86% of similarity, respectively, between the EF-1α, RPB1, RPB2, and beta-tubulin genes of the sequenced *Fusarium* sp. isolate PT and the *F. lateritium*, even though the ITS and LSU sequences were 100 and 98% identical (Supplementary Dataset 2). On the contrary, the sequence similarity of these genes with the ones from the *F. tricinctum* species complex were, indeed, higher but never reaching the 100% similarity (Supplementary Dataset 2).

Accordingly, to further disentangle the phylogenetic relationship among the *Fusarium* strains, a ML tree was built on 4,319 SNPs found in the core genome alignment derived from Panseq. As shown in Figure 3B, the clustering of our *Fusarium* sp. isolate PT within the FTSC was confirmed. The same clusterization was obtained when the orthologous proteins were identified and a species tree was built using OrthoFinder (Figure 3C). The average nucleotide identity (ANI) performed using blastn gave an overall picture of the sequence identity between the *Fusarium* strains under comparison, as shown by the heatmap in Figure 3D, with the *Fusarium* sp. isolate PT again among the FTSC isolates.

Finally, a selection of 63 *Fusarium* isolates, belonging to the FTSC for which both RPB1 and RPB2 nucleotide sequences were available, was used to build an additional ML phylogenetic tree. As in the previous trees, the *Fusarium* sp. PT isolate constitutes an independent branch within the dendrogram (Supplementary Figure 1).

### Characterization of Transcripts Involved in Pathogenesis

It is well-known that proteins are secreted in many *Fusarium* species during the colonizing stages (Ma et al., 2010), and for this reason, we went looking for the transcripts involved in pathogenesis.

In fact, 564 analogous genes involved in pathogenicity were identified from the PHI database. Among these, 24 genes were detected to be putative secreted proteins using SignalP. The 408 carbohydrate-active enzymes were detected, approximately 220 of which encoding glycoside hydrolases (GHs) and 79 genes encoding glycosyltransferases (Gts) (Table 3). Among the 1,288 proteins with a signal peptide, TargetP identified 525 proteins with a mitochondrial transit peptide, whereas 1,813 proteins resulted to have a possible cleavage site (Supplementary Figure 2). The predicted transmembrane helices found using TMHMM are reported in Supplementary Dataset 3.

**TABLE 3:** Characterization of the transcripts involved in pathogenesis.

	*Fusarium* sp. PT
Total Transcripts	12,093
Secreted Proteins	519
PHI base	564
Unaffected pathogenicity	292
Reduced virulence	244
Lethal	45
Loss of pathogenicity	26
Hypervirulence	9
Chemistry target: resistance to chemical	1
Enhanced antagonism	1
PHI base secreted	24
Unaffected pathogenicity	13
Reduced virulence	9
Loss of pathogenicity	1
Lethal	1
Hypervirulence	2
CAZy enzymes	408
Biosynthetic Gene Clusters	39

Seventeen biosynthetic gene clusters belonging to T1PKSs, NRPs, and terpene synthases (Tss) were found using antiSMASH 6. Among the T1PKSs gene cluster, the fujikurin A, B, C, and D were found with 83% of similarity, bikaverin with 42 or 57% of similarity, and fusarielin H and oxyjavanicin with 50% of similarity. Besides gibepyrone A with 40% of similarity, the ACT-Toxin II shared 100% identity with the sequence deposited in the database. The NRP gene cluster is represented mainly by ilicicolin H (50% of similarity) and chrysogine (83%), whereas BGCs belonging to bassianolide, beauvericin, and fusariodione A showed a similarity below 20%. Finally, the koraiol and α-acorenol terpenes showed 100% of similarity, whereas squalestatin S1 and gibberellin were of 40% similarity.

The complete sequence of the enniatin gene was also identified, sharing 85 and 86% of similarity with the gene deriving from *F. scirpi* and *F. tricinctum* strain INRA104, respectively (Supplementary Table 3).

## Discussion

*Fusarium* is a wide fungal genus including numerous species, with an equally broad host range. Its classification has been traditionally based on morphological characters, such as asexual distinctive banana-shaped septate macroconidia (Leslie and Summerell, 2006). However, in the last decades, molecular approaches have made the species distinction more accurate, allowing the depiction of more than 300 phylogenetically distinct species (O’Donnell et al., 2004; Druzhinina et al., 2006). In most cases, the molecular analysis behind the species identification and the assignment of strains to definite species was, and still is, based on a multilocus sequence typing (MSLT) approach, meaning the comparison of complete or partial sequences of a bunch of housekeeping genes used, for example, in FUSARIUM-ID (Geiser et al., 2004; Park et al., 2010) and Fusarium MLST (O’Donnell et al., 2010). However, as already thoroughly discussed in several papers (see O’Donnell et al., 2015), even this approach during time has shown some limits. For instance, in *Fusarium*, ITS and LSU are often scarcely informative at species level and should be avoided, giving preference to EF1, RPB1, and RPB2.

In addition, it must be considered that the complexity of the genus and the reported criticalities in the selection of genes for species identification have sometimes led to misidentification of strains and, consequently, to species assignment in corresponding sequences when deposited in molecular databases.

It, therefore, seems clear why the new whole-genome sequencing (WGS) technologies are a powerful tool to solve these misunderstandings. Furthermore, they do not only allow accurate phylogenetic analysis but also provide the basic for a thorough understanding of molecular pathogenetic mechanisms involved in the plant-pathogen iteration, like the identification of the effector genes (Plissonneau et al., 2016; Möller and Stukenbrock, 2017). Knowledge of the infection pathways of the pathogen could allow to develop more effective control strategies. Thus, obtaining a high-quality genome as complete as possible, for those species who have not been sequenced yet, is an essential step to take.

In this study, we report the draft genome sequence of the strain *Fusarium* sp. isolate PT isolated from hazelnut in Central Italy, which initially was thought to be *F. lateritium*, due to the previous knowledge about the symptoms induced in the host and the morphology of the pathogen itself (Belisario and Santori, 2009; Santori et al., 2010; Vitale et al., 2011). In fact, its morphological traits observed under the optical microscope showed the typical banana-shaped septate macroconidia of *F. lateritium* (Leslie and Summerell, 2006). Furthermore, ITS and translation elongation factor 1-alpha sequencing corroborated this first hypothesis, guiding us toward a WGS with the aim to obtain a genome of this pathogen with the highest possible quality.

Accordingly, four different assembly approaches were applied, using both long reads obtained by ONT MinION and short reads by Illumina sequencing technologies, either in combination (hybrid assembly) or alone: (i) Miniasm-Minimap2, which use the overlap-layout-consensus (OLC); (ii) SPAdes uses the de Bruijn graph; (iii) MaSuRCA combines the de Bruijn graph with OLC, creating intermediate super-reads, further polished with POLCA; and (iv) Canu follows a MinHash Alignment Process (MHAP) based on k-mer weighting.

The advantage of using long reads compared with using only short reads has been extensively proved, especially for more complex genomes that are rich in transposons or tandem repeat (Goodwin et al., 2016; van Dijk et al., 2018). In addition, performing a hybrid assembly guarantee to take advantage, from one side, of the depth coverage and basecalling quality given by the Illumina reads and, on the other side, to increase the genome contiguity with the ONT reads (Chen et al., 2020). In fact, this method proved to be very successful in fungal genome assembly, either within the *Fusarium* world (Million et al., 2019; Degradi et al., 2021; Dvorianinova et al., 2021; Fan et al., 2021) or to extended species (Faino et al., 2015; Saud et al., 2021).

The same consideration applies to the assembly of the *Fusarium* sp. isolate in the study. In fact, the draft genome assembled by SPAdes using only Illumina reads gave the highest number of contigs but also undetermined nucleotides N, probably coming from repetitive regions or those gaps that Illumina reads could not fill. On the contrary, Canu, whose pipeline includes a *de novo* assembly from long reads followed by polishing with short reads, seemed to be the best strategy, also when compared with both MaSuRCA and Miniasm-Minimap2 hybrid assemblies. In fact, Canu produced less contigs (27) with the highest N50 (2,955,107bp) without Ns. In literature, good performance of Canu have been reported in *F. musae* (Degradi et al., 2021), *F. oxysporum* f. sp. *capsici* (Xingxing et al., 2021), and *F. oxysporum* f. sp. *lini* (Krasnov et al., 2020).

Accordingly, using the draft genome obtained using Canu for further analysis and annotation, different phylogenetic analysis surprising showed that the *Fusarium* sp. isolate PT is more closely related to *F. tricinctum* species complex than *F. lateritium.* These results were further supported by the species tree based on clustering of the orthologs proteins and on the dendrogram based on the ANI results.

Taken these results all together, it was once more demonstrated that species identification using only one or two housekeeping genes may led to wrong species assignment, as already shown for the ITS region in fungi such as *Aspergillus*, *Fusarium*, *Penicillium*, and *Trichoderma* (Raja et al., 2017).

In fact, polyphasic taxonomic approach, instead, gives a more robust classification of the species under examination, from the bacterial level to the more complicated *Fusarium* family (Das et al., 2014; Crous et al., 2021). In contrast, the still limited availability of WGSs in the databases may restrict the comparison range. But thanks to the drop in sequencing price and the constant increase in computational power, a WGS followed by comparative genomics analysis is becoming the best choice for a precise taxonomy identification. Moreover, each effort taken to increase the database availability contributes to fulfill gaps in fungal knowledge, especially for a variegated genus as the one of *Fusarium*.

At the same time, we did not go further in the attempt to assign a species name to the isolate in subject, leaving it at the status of undefined species. This agrees with the concept described by Summerell (2019) that the depiction of a new species must require the study of a number of strains with coherent genetic features that has to be sufficient to also describe the range of genetic variability within the new species.

From a strictly phytopathological point of view, this is the first report of a *Fusarium* isolate referable to the *F. tricinctum* species complex associated with the already known disease named NGN. In fact, to our knowledge, the only other report of *Fusarium* related to the *tricinctum* species complex associated with hazelnut comes from Iran and refers to a generic plant decline (Ghasemi and Davari, 2019). The NGN has been, instead, well studied and repeatedly attributed to the pathogenic action of *F. lateritium* (Belisario and Santori, 2009; Santori et al., 2010; Vitale et al., 2011), with a secondary role for *Alternaria* spp. Recently, the same disease was reported for the first time in La Araucanìa, Chile, and again different fungi were isolated from diseased fruit: *Fusarium* sp., even if the closest species according to ITS blast resulted *F. sporotrichioides*, but also *Alternaria alternata*, *Diaporthe* sp., *Phomopsis* sp., and *Neofusicoccum* sp. (Duran et al., 2020).

Several authors sustain the complexity of nut defects and rotting and the simultaneous occurrence of different pathogens in disease expression. Recently, Arciuolo et al. (2020), studying the fungal species associated with defective hazelnuts in Turkey, found that the prevalent fungi were *Alternaria*, *Aspergillus*, *Botryosphaeria*, *Diaporthe*, *Fusarium*, *Penicillium*, and *Pestalotiopsis*, proposing a major role for *Diaporthe* genus. In a previous paper, the species *D. eres* was demonstrated to be the main reason for the occurrence of brown spots on the kernel surface and of internal discoloration of nuts (Battilani et al., 2018).

In Oregon, Pscheidt et al. (2018), studying the fungi involved in kernel mold on hazelnut, found out that *Penicillium* spp., *Aspergillus* and *Cladosporium* spp., and *D. rudis* were frequently isolated, together with *F. lateritium* (identified by ITS and EF-1α).

All these studies demonstrate unequivocally that several fungi can concurrently invade and damage hazelnut generating various expressions of external and internal defects. Among them, *Fusarium* species undoubtedly have a key role in this type of disease. What remains to be assessed is the specific relevance of each of these species in the disease progression and which environmental factors influence the evolution of the disease. Investigating genomes as we did in this study, beside shedding light in the taxonomy of fungal species, particularly significant for the complex genus *Fusarium*, should allow to identify the genetic features of the pathogens involved in pathogenicity, thus representing milestones in understanding its evolution and eventually plan efficient control strategies to protect an important Italian crop as *C. avellana*.

## Data Availability Statement

The Genome sequence can be found on the NCBI database genome under the accession number provided. The raw sequencing data are available under request.

## Author Contributions

ST performed all the bioinformatics analysis, interpreted the results, prepared the figures, and wrote the manuscript in consultation with MD, LF, and AM. AG conceived and designed the study, collected the samples, and participated in the analysis. MD and CD performed the lab experiments. LF and MR provided the critical feedback and helped shape the research. VC and AM supervised the project and approved all the analysis. All authors contributed to the article and approved the submitted version.

## Conflict of Interest

The authors declare that the research was conducted in the absence of any commercial or financial relationships that could be construed as a potential conflict of interest.

## Publisher’s Note

All claims expressed in this article are solely those of the authors and do not necessarily represent those of their affiliated organizations, or those of the publisher, the editors and the reviewers. Any product that may be evaluated in this article, or claim that may be made by its manufacturer, is not guaranteed or endorsed by the publisher.

## Footnotes

1
https://www.istat.it/

2
http://www.fao.org/faostat/

3 FigTree (http://tree.bio.ed.ac.uk/software/figtree)

## Supplementary Material

Supplementary Figure 1 Phylogenetic tree of RPB1 and RPB2 concatenated sequences among FTSC strains. The nucleotide sequence of RPB1 and RPB2 genes of a selection of 63 strains belonging to the FTSC were concatenated, aligned, and used to build a ML tree using RAxML, from which only bootstraps higher than 60 are shown.

Supplementary Figure 2 Results of TargetP analysis. Cumulative count of predicted proteins containing a signal peptide (SP), mitochondrial translocation signal (mTP), a possible cleavage site (CS) or other possible signal (chloroplast, extracellular, and “other” localization).

Supplementary Table 1 List of 63 *Fusarium* isolates belonging to *Fusarium tricinctum* species complex (FTSC) used for pairwise comparison and phylogenetics analysis of the RPB1 and RPB2 gene sequences.

Supplementary Table 2 List of the first 100 most significant sequence resulted from the BLASTn aligment of the ITS and the EF-1α region of the isolated strain.

Supplementary Table 3 List of *Fusarium* strains showing identity with the enniatin gene of the *Fusarium* sp. isolate PT.
