# Supplementary material for: Draft Genome Sequence of a New Fusarium Isolate Belonging to Fusarium tricinctum Species Complex Collected From Hazelnut in Central Italy
Source: Front Plant Sci. 2021 Dec 16;12:788584. doi: 10.3389/fpls.2021.788584 (PMC8718101; doi:10.3389/fpls.2021.788584)
Supplement: Supplementary Figure 1 — Phylogenetic tree of RPB1 and RPB2 concatenated sequences among FTSC strains. The nucleotide sequence of RPB1 and RPB2 genes of a selection of 63 strains belonging to the FTSC were concatenated, aligned, and used to build a ML tree using RAxML, from which only bootstraps higher than 60 are shown. [file Data_Sheet_1.zip › Supplementary Dataset 1.DOCX]

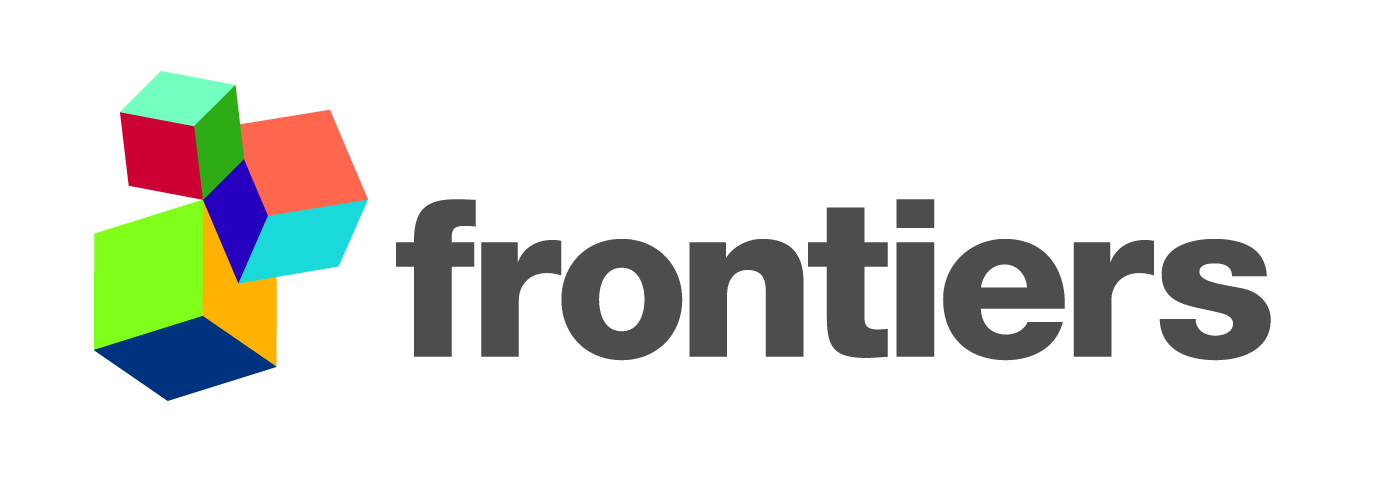


**Supplementary Dataset 1**

**Busco completess of the three best quality assemblies evaluated through comparison with 4,494 conserved ORF of the Hypocreales order.**

|  | Miniasm-Minimap2 | MaSuRCA | Canu |
| --- | --- | --- | --- |
| Complete BUSCOs (C) | 4475 | 4486 | 4469 |
| Complete and single-copy BUSCOs (S) | 4468 | 4478 | 4462 |
| Complete and duplicated BUSCOs (D) | 7 | 8 | 7 |
| Fragmented BUSCOs (F) | 1 | 0 | 2 |
| Missing BUSCOs (M) | 18 | 8 | 23 |
| Total BUSCO groups searched | 4494 | 4494 | 4494 |
| Total % completeness | 99.6 % | 99.8 % | 99.5 % |
