# Supplementary material for: Draft Genome Sequence of a New Fusarium Isolate Belonging to Fusarium tricinctum Species Complex Collected From Hazelnut in Central Italy
Source: Front Plant Sci. 2021 Dec 16;12:788584. doi: 10.3389/fpls.2021.788584 (PMC8718101; doi:10.3389/fpls.2021.788584)
Supplement: Supplementary Figure 1 — Phylogenetic tree of RPB1 and RPB2 concatenated sequences among FTSC strains. The nucleotide sequence of RPB1 and RPB2 genes of a selection of 63 strains belonging to the FTSC were concatenated, aligned, and used to build a ML tree using RAxML, from which only bootstraps higher than 60 are shown. [file Data_Sheet_1.zip › Supplementary Dataset 2.DOCX]

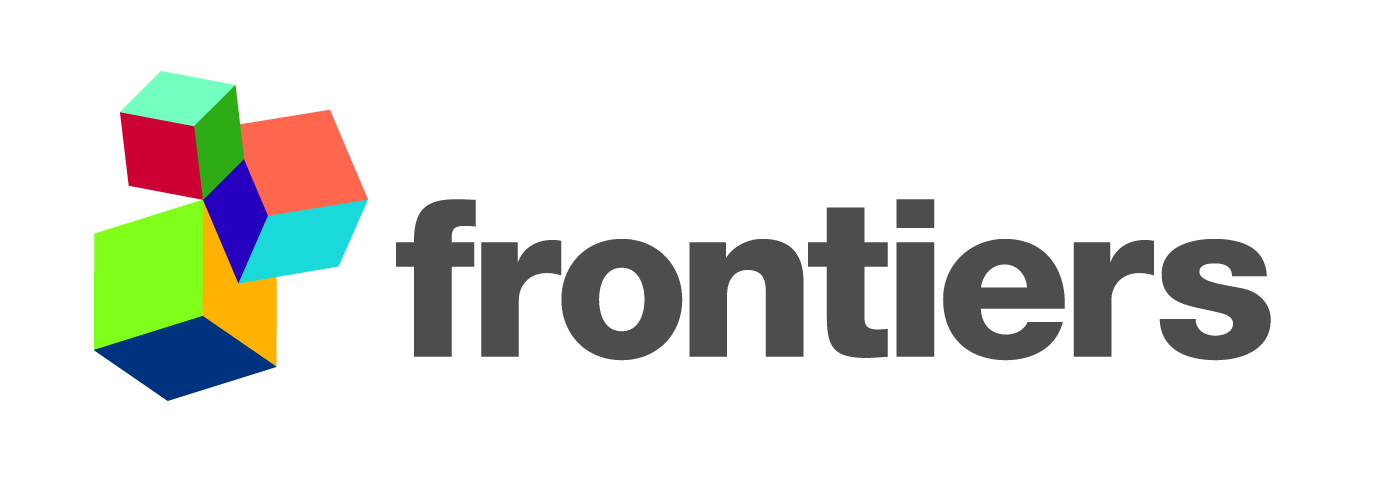


**Supplementary Dataset 2**

**BLASTn pairwise alignment between the *Fusarium* sp. PT strain and *Fusarium lateritium* NRRL13622**

RID: N8GS4CEW114

Job Title:Fusarium_PT_Tef1a

Program: BLASTN

Query: Fusarium_PT_Tef1a ID: lcl|Query_14973(dna) Length: 678

Subject:Fusarium_lateritium_NRRL13622 ID: lcl|Query_14975(dna) Length: 701

Sequences producing significant alignments:

Scientific Common Max Total Query E Per. Acc.

Description Name Name Taxid Score Score cover Value Ident Len Accession

Fusarium_lateritium_NRRL13622 0 558 558 100% 3e-163 82.06 701 Query_14975

Alignments:

>Fusarium_lateritium_NRRL13622

Sequence ID: Query_14975 Length: 701

Range 1: 1 to 701

Score:558 bits(302), Expect:3e-163,

Identities:590/719(82%), Gaps:59/719(8%), Strand: Plus/Plus

Query 1 AAGACTCACCTTAACGTCGTCGTCATCGGCCACGTCGACTCTGGCAAGTCGACCACTGTA 60

|||||||||||||||||||||||||||||||||||||||||||||||||||||||||||

Sbjct 1 AAGACTCACCTTAACGTCGTCGTCATCGGCCACGTCGACTCTGGCAAGTCGACCACTGTG 60

Query 61 AGTTCAACCATCAGCG--AGT-TGCTTATCTGCAC---TCGAGCCTGCCACATCTGGCGG 114

||| | ||| || || | | ||||||||||||| || | || || | ||||| |

Sbjct 61 AGTACTACCCTC-TCGACAATGTGCTTATCTGCACCCGTCAAACC--CC-CGCCTGGC-G 115

Query 115 GGGTACTACCG-CA-ACAC-T-TTGCTAA---CTTTTGACAGACCGGTCACTTGATCTAC 167

||||| | ||| || ||| | |||||| | || || ||||||||||||||||||||

Sbjct 116 GGGTA-T-CCGACATGCACATCATGCTAACGCCCTTGGATAGACCGGTCACTTGATCTAC 173

Query 168 CAGTGCGGTGGTATCGACAAGCGAACCATCGAGAAGTTCGAGAAGGTTAGTC-ATTATCC 226

|||||||||||||||||||||||||||||||||||||||||||||||| ||| ||| |||

Sbjct 174 CAGTGCGGTGGTATCGACAAGCGAACCATCGAGAAGTTCGAGAAGGTTGGTCCATTTTCC 233

Query 227 CTTCGATTCCGCGCGCT---CCCATCGAA---TCCTACGACTCGCTCCATCACTCGAATC 280

| ||| ||||| || |||||||| |||||||| ||||||| | || || ||

Sbjct 234 CA-CGAGCGCGCGCCCTTTGCCCATCGATTTGTCCTACGAATCGCTCCCTTAC--GACTC 290

Query 281 GCATC-CAT-TACCCCGCTCGAGTCCGAAAATTTTGCGGTGCGACCGTGAATTCTTTTTG 338

||| | | ||||||||||||| || ||||||||||||| |||||| |||| ||||||

Sbjct 291 GCAACACGCCTACCCCGCTCGAGCCCAAAAATTTTGCGGTTTGACCGT-AATT-TTTTTG 348

Query 339 GTGGGGTATCTTACCCCGCCACTCGAGTGACGGATGCGCTTGCCCTGTTCCC---ACA-A 394

|||||| |||||||||||||||||||| || | ||||||||||||| ||| ||| |

Sbjct 349 GTGGGGCATCTTACCCCGCCACTCGAGCGATAG--GCGCTTGCCCTGTCCCCTGCACACA 406

Query 395 AATTTCACTACCC-T--GC-CGCGCACCAACATGTC-T-TG-CAGTCACTAACCAT--TG 445

|| ||| ||||| | | ||||| || ||||| | || |||| ||||||| |

Sbjct 407 AAACTCAATACCCATTTGGGCGCGCGTCATCATGTGATCTGACAGTTGCTAACCACCATC 466

Query 446 GACAATAGGAAGCCGCCGAGCTCGGAAAGGGTTCCTTCAAGTATGCCTGGGTTCTTGACA 505

||||||||||||||||||||||||||||||||||||||||||| || |||||||||||||

Sbjct 467 GACAATAGGAAGCCGCCGAGCTCGGAAAGGGTTCCTTCAAGTACGCTTGGGTTCTTGACA 526

Query 506 AGCTCAAAGCCGAGCGTGAGCGTGGTATCACCATTGATATCGCTCTCTGGAAGTTCGAGA 565

|||||||||||||||||||||||||||||||||| || || || || |||||||||||||

Sbjct 527 AGCTCAAAGCCGAGCGTGAGCGTGGTATCACCATCGACATTGCCCTGTGGAAGTTCGAGA 586

Query 566 CTCCTCGCTACTATGTCACCGTCATTGGTATGTTGTTACTG-T--CT-CACGCCACCATG 621

|||| |||||||||||||||||||||||||||| || |||| | || || | || | |

Sbjct 587 CTCCCCGCTACTATGTCACCGTCATTGGTATGTCGTCACTGCTGTCTTCATTC-AC-ACG 644

Query 622 CCTCCATGCTAAC-C-TCTCTCAGATGCCCCCGGTCATCGTGACTTCATCAAGAACATG 678

|||| || ||||| | || |||| || |||||||| |||||||||||||||||||||

Sbjct 645 CCTC-AT-CTAACACATCATGCAGACGCTCCCGGTCACCGTGACTTCATCAAGAACATG 701

RID: N8M2R9CY11R

Job Title:Fusarium_lateritium_NRRL13622

Program: BLASTN

Query: Fusarium_lateritium_NRRL13622 ID: lcl|Query_40001(dna) Length: 1602

Subject:Fusarium_PT_RPB1 ID: lcl|Query_40003(dna) Length: 1604

Sequences producing significant alignments:

Scientific Common Max Total Query E Per. Acc.

Description Name Name Taxid Score Score cover Value Ident Len Accession

Fusarium_PT_RPB1 0 1805 1805 100% 0.0 87.06 1604 Query_40003

Alignments:

>Fusarium_PT_RPB1

Sequence ID: Query_40003 Length: 1604

Range 1: 1 to 1601

Score:1805 bits(977), Expect:0.0,

Identities:1400/1608(87%), Gaps:13/1608(0%), Strand: Plus/Plus

Query 1 TTCCTCACAAAGGAGCAGATCATGAACTGTATGCTTTGGGTGCCCAACTGGGATGGTGTC 60

|||||||| ||||| |||||||||||||| ||||| ||||||||||||||||| ||||||

Sbjct 1 TTCCTCACCAAGGAACAGATCATGAACTGCATGCTCTGGGTGCCCAACTGGGACGGTGTC 60

Query 61 ATTCCCCAGCCCGCTATCTACAAGCCTCGTCCTCGTTGGACTGGAAAGCAACTTATAAGC 120

||||| |||||||||||||| || ||||||||||||||||| || ||||| || || |||

Sbjct 61 ATTCCTCAGCCCGCTATCTATAAACCTCGTCCTCGTTGGACCGGTAAGCAGCTCATCAGC 120

Query 121 ATGGTTATCCCTAAGGAGGTTACCCTTTTCAATGGTACGGACAGTGGTGAAAATGCCCCT 180

||||| |||||||||||||||| |||||| |||||||||||| || ||| |||||||||

Sbjct 121 ATGGTCATCCCTAAGGAGGTTAGCCTTTTTAATGGTACGGACGGTAATGAGAATGCCCCT 180

Query 181 CTTAAGGACGAGGGTCTTTTGATCCAGGCCGGCCAGCTTATGTATGGTCTTCTCACCAAG 240

|||| ||| |||||||||||||| || ||||||| || ||||| |||||||||||||||

Sbjct 181 CTTAGGGATGAGGGTCTTTTGATTCAATCCGGCCAACTAATGTACGGTCTTCTCACCAAG 240

Query 241 AAGAGCGTTGGTGCTGCTGCCGGTGGTATTGTGCACATCAGCTACAACGAGCTTGGACCT 300

|||||||||||||||||||| |||||||| || || || || ||||| ||||| ||||||

Sbjct 241 AAGAGCGTTGGTGCTGCTGCTGGTGGTATCGTTCATATTAGTTACAATGAGCTGGGACCT 300

Query 301 GAGGGTGCGATGGCTTTCTTGAACGGTGTCCAGCAGACTGTCACTTACTGGCTACTGAAC 360

||||| || ||||||||||||||||||||||||||| ||| ||||||||||| ||||||

Sbjct 301 GAGGGAGCTATGGCTTTCTTGAACGGTGTCCAGCAGGTTGTGACTTACTGGCTTCTGAAC 360

Query 361 AACGGTCACAGCATTGGTATCGGTGACACAATTCCCGATGCGGCGACCATTGCCAAGGTC 420

||||||||||| |||||||| ||||||||| |||| ||||| | || |||||||||||

Sbjct 361 AACGGTCACAGTATTGGTATTGGTGACACAGTTCCTGATGCTGTTACTATTGCCAAGGTT 420

Query 421 CAGGTGCATATCGATGAGGAGAAGGCTGAGGTCGCTCGCTTGACAGCCATGGCTACTGCC 480

|||| |||||| |||||||| |||| ||| || ||||| |||||||| ||||| ||||||

Sbjct 421 CAGGCGCATATTGATGAGGAAAAGGATGAAGTTGCTCGTTTGACAGCGATGGCCACTGCC 480

Query 481 AACGAGCTTGAGGCCCTGCCCGGTATGAATGTCCGAGCAACATTCGAAAACAAGGTCTCG 540

||||||||||||||||| ||||||||||| || || ||||||||||| |||||||||||

Sbjct 481 AACGAGCTTGAGGCCCTTCCCGGTATGAACGTTCGTGCAACATTCGAGAACAAGGTCTCT 540

Query 541 ATGGCTCTTAACCAGGCCCGTGATAAGGCTGGTACCACAACACAGAAGAGTTTGAAGGAT 600

|||||||| ||||| |||||||| ||||||||||| ||||||||||||||||| ||||||

Sbjct 541 ATGGCTCTGAACCAAGCCCGTGACAAGGCTGGTACGACAACACAGAAGAGTTTAAAGGAT 600

Query 601 TCAAACAACGCCGTCACCATGGCTTCCTCAGGTTCCAAGGGTTCTTCCATCAACATTTCA 660

||||||||||| || ||||||||||| |||||||||||||||||||| ||||||||||||

Sbjct 601 TCAAACAACGCTGTTACCATGGCTTCTTCAGGTTCCAAGGGTTCTTCTATCAACATTTCA 660

Query 661 CAGATGACTGCCCTTGTCGGTCAGCAAATTGTTGAAGGCAAGCGTATTCCTTTCGGATTC 720

|||||||||||||||||||||||||||||||||||||||||||||||||||||||| |||

Sbjct 661 CAGATGACTGCCCTTGTCGGTCAGCAAATTGTTGAAGGCAAGCGTATTCCTTTCGGTTTC 720

Query 721 AAGTACCGCACCCTGCCTCACTTCACCAAGGATGATTACTCACCCGAAGCCCGTGGTTTC 780

|||||||| ||||||||||| ||||||||||| ||||||||||||||||| |||||||||

Sbjct 721 AAGTACCGTACCCTGCCTCATTTCACCAAGGACGATTACTCACCCGAAGCTCGTGGTTTC 780

Query 781 GTTTGAGAACTCTTATCTTCGTGGCCTTACTCCTAGCGAGTTCTTCTTCCACGCCATGGC 840

| |||||||||| || |||||||| ||||| || ||||||||||| ||||||||||||||

Sbjct 781 G-TTGAGAACTCCTACCTTCGTGGTCTTACCCCCAGCGAGTTCTTTTTCCACGCCATGGC 839

Query 841 TGGTCGAGAAGGTCTTATTGATACTGCAGTCAAGACTGCTGAAACAGGTTACATTCAGCG 900

||||||||||||||| ||||||||||| ||||||||||| ||||||||||||||||||||

Sbjct 840 TGGTCGAGAAGGTCTCATTGATACTGCTGTCAAGACTGCCGAAACAGGTTACATTCAGCG 899

Query 901 ACGACTGGTCAAGGCTCTGGAAGATCTTTCTGCGCGCTACGATGGAACCGTCCGAAACTC 960

|||| ||||||||||| |||||||||||||||||||||| |||||||| || ||||||||

Sbjct 900 ACGATTGGTCAAGGCTTTGGAAGATCTTTCTGCGCGCTATGATGGAACTGTTCGAAACTC 959

Query 961 TCTGGGTGATATTGTTCAGTTCCTCTACGGTGAAGATGGTCTCGATGCTATGATCATTGA 1020

|||||||||||||||||| ||||| ||||| || ||||||||||||||||||||||||||

Sbjct 960 TCTGGGTGATATTGTTCAATTCCTTTACGGCGAGGATGGTCTCGATGCTATGATCATTGA 1019

Query 1021 GAAACAGAAACTGGGAATCCTCAACATGTCCAACAGTGCTTTCGAAAAGAAGTACCGTCT 1080

||| ||||| |||||||| |||||||||||||||||||| ||||||||||||||||||||

Sbjct 1020 GAAGCAGAAGCTGGGAATTCTCAACATGTCCAACAGTGCATTCGAAAAGAAGTACCGTCT 1079

Query 1081 GGATCTTGCTAACCCCCCTGAGTGGTTCAAGCAAGACTACGAATTTGGTAACGAGTTGAC 1140

||||||||| ||||| ||||| ||||| | ||| ||||||||||| || |||||||||||

Sbjct 1080 GGATCTTGCCAACCCTCCTGATTGGTTTAGGCACGACTACGAATTCGGCAACGAGTTGAC 1139

Query 1141 TGGTGACA-AGCCTTCAATG-GCTCTTCTCGACTCCGAATGGGACAGGTTATT-GA-AGG 1196

|||||||| ||| ||| ||| || |||||||| |||||||| || | | || | |

Sbjct 1140 TGGTGACAGAGC-TTCCATGAGC-CTTCTCGATGAAGAATGGGA--GGCTCTGCGATACG 1195

Query 1197 ACCGCCGGGACATCCGACGA-ATCAACAAGTCCAAGATGAACGAGGAAATGATGCAGTTG 1255

| ||| | ||||| || ||||| | ||| ||| |||||||| |||||||| ||

Sbjct 1196 ATCGCAGAAGGATCCGG-GATATCAATAGGTCAAAGGGTAACGAGGAGATGATGCAATTA 1254

Query 1256 CCACTCAACATTACTCGTATCATCGAGTCGGCTAAGCGTGTGTTTAGCGTCAGGGCCAAC 1315

|| |||||||| || || ||||||||||| || |||||||| || | |||| ||||||

Sbjct 1255 CCTCTCAACATCACCCGCATCATCGAGTCTGCCAAGCGTGTATTCAATGTCAAGGCCAAT 1314

Query 1316 GACCGAAGCAACTTGCGACCCTCGGATGTCATTCCTGCTGTCCAGAA-CATGCTGAACAA 1374

|||||||| |||||||||||||||||||| || || | || |||| |||| || | ||

Sbjct 1315 GACCGAAGTAACTTGCGACCCTCGGATGTTATCCCCGGAGTG-AGAAACATGTTGGAAAA 1373

Query 1375 CATGAAGATTGTTCGTGGTACCGACGATATCTCGATAGAGGCTGACGCCAACGCCACCAT 1434

||||||||| ||||| ||||| ||||| || || | |||||||||||||||||| ||||

Sbjct 1374 CATGAAGATCGTTCGAGGTACTGACGAGATTTCATTGGAGGCTGACGCCAACGCCTCCAT 1433

Query 1435 TCTATTCAAGGGCTTGTTACGCTCTCGACTGGCCTTCAAGGAGGTGGTGAAGGAGCACCG 1494

||| ||||||| | | | || || ||||| ||||||||||| || || |||||||||||

Sbjct 1434 TCTCTTCAAGGCCCTCCTCCGTTCCCGACTAGCCTTCAAGGAAGTTGTCAAGGAGCACCG 1493

Query 1495 ATTGAACAAACTGGCTTTCGACCATATTCTGGGCGAACTTCAGAACAGATGGGATCGTGC 1554

||||| || ||||||||||| | || |||| ||||| ||||| |||||||| |||||

Sbjct 1494 CTTGAATAAGCTGGCTTTCGATTACATCTTGGGTGAACTCCAGAATAGATGGGACCGTGC 1553

Query 1555 CTTTGTTAACCCTGGTGAAATGGTTGGTGTGTTGGCTGCGCAGTCTAT 1602

||| || ||||||||||||||||||||||| ||||||| ||||||||

Sbjct 1554 CTTCGTCAACCCTGGTGAAATGGTTGGTGTTCTGGCTGCTCAGTCTAT 1601

RID: N8M3S0N111R

Job Title:Fusarium_PT_RPB2

Program: BLASTN

Query: Fusarium_PT_RPB2 ID: lcl|Query_44707(dna) Length: 1856

Subject:Fusarium_lateritium_NRRL13622 ID: lcl|Query_44709(dna) Length: 1858

Sequences producing significant alignments:

Scientific Common Max Total Query E Per. Acc.

Description Name Name Taxid Score Score cover Value Ident Len Accession

Fusarium_lateritium_NRRL13622 0 1352 1352 59% 0.0 88.61 1858 Query_44709

Alignments:

>Fusarium_lateritium_NRRL13622

Sequence ID: Query_44709 Length: 1858

Range 1: 742 to 1855

Score:1352 bits(732), Expect:0.0,

Identities:988/1115(89%), Gaps:4/1115(0%), Strand: Plus/Plus

Query 742 TTGAAGACAAAGGTCAATCCTACAACTCACGCGTACACACATTGCGAGATTCATCCCAGT 801

|||||||||||||| || || ||||||||| |||||| ||||| |||||||||||||||

Sbjct 742 TTGAAGACAAAGGTTAACCCCACAACTCACATGTACACCCATTGTGAGATTCATCCCAGT 801

Query 802 ATGATTCTTGGTATCTGTGCCAGTATCATTCCCTTCCCTGATCACAACCAGGTATGT--- 858

|||||||||||||| || |||||||||||||| ||||| ||||| ||||||||||||

Sbjct 802 ATGATTCTTGGTATTTGCGCCAGTATCATTCCATTCCCCGATCATAACCAGGTATGTGAC 861

Query 859 CATGACGCTTGATTTTCTTCGATCTTCACTAACAATACACAGTCTCCTCGTAACACCTAC 918

||| | ||||||| ||| || || |||||||| | |||||| |||||||||||||||

Sbjct 862 CATAAGTCTTGATTAACTT-GAGCTCAACTAACAACATACAGTCGCCTCGTAACACCTAC 920

Query 919 CAATCTGCCATGGGTAAACAAGCTATGGGTTTCTTCTTAACAAACTATTCTCGACGTATG 978

|||||||| ||||||||||||||||||||||||||||||||||| || |||||||| |||

Sbjct 921 CAATCTGCTATGGGTAAACAAGCTATGGGTTTCTTCTTAACAAATTACTCTCGACGCATG 980

Query 979 GATACCATGGCCAACATTCTCTACTACCCTCAAAAGCCCCTCGCCACTACTCGATCTATG 1038

|||||||||||||||||||||||||| || |||||||| |||||||||||||||||||||

Sbjct 981 GATACCATGGCCAACATTCTCTACTATCCGCAAAAGCCTCTCGCCACTACTCGATCTATG 1040

Query 1039 GAGCATCTCAAGTTCCGTGAACTGCCAGCTGGTCAAAACGCTATCGTTGCAATTGCCTGT 1098

||| ||||||||||||||||| ||||| || |||||||| || || || ||||| |||

Sbjct 1041 GAGTTCCTCAAGTTCCGTGAACTACCAGCCGGCCAAAACGCCATTGTCGCTATTGCTTGT 1100

Query 1099 TACTCAGGATACAACCAGGAAGATTCCGTTATTATGAATCAGAGCAGTATTGATAGAGGT 1158

|| ||||| ||||||||||||||||||||||||||||| ||||||||||| ||| |||||

Sbjct 1101 TATTCAGGTTACAACCAGGAAGATTCCGTTATTATGAACCAGAGCAGTATCGATCGAGGT 1160

Query 1159 CTATTCCGTAGTCTGTTCTTCCGATCGTACTCAGATCAGGAGAAGAAGGTCGGTCTCAAC 1218

|| ||||| ||||||||||||||||| ||||||||||||||||||||||| |||||||||

Sbjct 1161 CTGTTCCGCAGTCTGTTCTTCCGATCATACTCAGATCAGGAGAAGAAGGTTGGTCTCAAC 1220

Query 1219 TACACAGAAATATTTGAGAAGCCTTTCCAGCAGACAACACTTCGAATGAAGCACGGAACA 1278

||||| ||| | ||||||||||||||||||||| ||| |||||||||||||| |||||

Sbjct 1221 TACACCGAAGTGTTTGAGAAGCCTTTCCAGCAGCAAACGCTTCGAATGAAGCATGGAACT 1280

Query 1279 TACGACAAGCTTGACGAAGATGGTATTGTGGCTCCTGGTGTACGAGTTTCTGGTGAAGAT 1338

|||||||||||||| || ||||||||||||||||||||||| ||||| || |||||||||

Sbjct 1281 TACGACAAGCTTGATGAGGATGGTATTGTGGCTCCTGGTGTGCGAGTGTCGGGTGAAGAT 1340

Query 1339 ATCATCATTGGCAAGACGGCGCCCATCGATCAGGAGAATCAGGATCTGGGTACCAGAACC 1398

||||| || |||||||| || || ||||| |||||||| |||||||||||| |||| ||

Sbjct 1341 ATCATTATCGGCAAGACTGCACCGATCGACCAGGAGAACCAGGATCTGGGTGCCAGGACT 1400

Query 1399 CAATCGCACCAGCGTCGTGATATCTCGACACCGCTGCGAAGTACAGAAAACGGTATCGTT 1458

||| ||||||||||||| |||||||| |||||||||||||||||||| ||||||||||||

Sbjct 1401 CAAGCGCACCAGCGTCGCGATATCTCAACACCGCTGCGAAGTACAGAGAACGGTATCGTT 1460

Query 1459 GATCAAGTCATTCTGACAGTCAACGCCGACAACGTGAAGTACGTGAAGGTTCGAGTACGA 1518

|||||||||||| |||| ||||||||||| ||||| |||||||| |||||||| || ||

Sbjct 1461 GATCAAGTCATTTTGACGGTCAACGCCGATAACGTCAAGTACGTCAAGGTTCGTGTGAGA 1520

Query 1519 ACCACCAAGATTCCCCAGATTGGTGACAAGTTTGCTTCTCGTCACGGTCAGAAGGGTACC 1578

||||||||||| ||||||||||||||||||||||| ||||||||||| || |||||||||

Sbjct 1521 ACCACCAAGATCCCCCAGATTGGTGACAAGTTTGCCTCTCGTCACGGACAAAAGGGTACC 1580

Query 1579 ATTGGTGTCACTTATCGTCAGGAAGATATGCCTTTCAGCAGAGAAGGCCTGACTCCCGAT 1638

||||||||||| || || || |||||||||||||||||||||||||| ||||| || |||

Sbjct 1581 ATTGGTGTCACCTACCGACAAGAAGATATGCCTTTCAGCAGAGAAGGTCTGACCCCGGAT 1640

Query 1639 ATTATCATCAACCCTCACGCTATTCCCTCTCGAATGACAATTGCCCATTTGATTGAGTGT 1698

|||||||||||||||||||| |||||||| |||||||| |||||||||||||||||||||

Sbjct 1641 ATTATCATCAACCCTCACGCCATTCCCTCACGAATGACGATTGCCCATTTGATTGAGTGT 1700

Query 1699 CTTCTTAGTAAGGTCTCAACACTTGAAGGTATGGAGGGTGATGCAACACCGTTCACCGAC 1758

|| || |||||||||||||| || ||||||||||||||||| ||||| || ||||| ||

Sbjct 1701 CTCCTCAGTAAGGTCTCAACGCTCGAAGGTATGGAGGGTGACGCAACGCCTTTCACTGAT 1760

Query 1759 GTGACGGTCGACTCCGTCTCAGACCTTCTAAGAAAGCACGGCTACCAATCTCGAGGCTTC 1818

|| ||||||||||| ||||| || ||||| || || ||||| |||||||| ||||| |||

Sbjct 1761 GTTACGGTCGACTCTGTCTCGGATCTTCTGAGGAAACACGGTTACCAATCACGAGGTTTC 1820

Query 1819 GAGGTCATGTACAATGGTCACACTGGACGCAAGCT 1853

|||||||||||||| || ||||| || || |||||

Sbjct 1821 GAGGTCATGTACAACGGCCACACAGGTCGTAAGCT 1855

RID: N8M80DE311R

Job Title:Fusarium_PT_beta_tub

Program: BLASTN

Query: Fusarium_PT_beta_tub ID: lcl|Query_66965(dna) Length: 619

Subject:Fusarium_sp_PT_beta_tub ID: lcl|Query_66967(dna) Length: 619

Sequences producing significant alignments:

Scientific Common Max Total Query E Per. Acc.

Description Name Name Taxid Score Score cover Value Ident Len Accession

Fusarium_sp_PT_beta_tub 0 1144 1144 100% 0.0 100.00 619 Query_66967

Alignments:

>Fusarium_sp_PT_beta_tub

Sequence ID: Query_66967 Length: 619

Range 1: 1 to 619

Score:1144 bits(619), Expect:0.0,

Identities:619/619(100%), Gaps:0/619(0%), Strand: Plus/Plus

Query 1 AGAGAGTGGGTGATCTGGAAACCCTGGAGGCAGTCACAGCCCTCTGCCTCACGACGGACG 60

||||||||||||||||||||||||||||||||||||||||||||||||||||||||||||

Sbjct 1 AGAGAGTGGGTGATCTGGAAACCCTGGAGGCAGTCACAGCCCTCTGCCTCACGACGGACG 60

Query 61 ACATCGAGAACCTGATCGACAAGCTCGGCACCCTCAGTGTAGTGACCCTTGGCCCAGTTG 120

||||||||||||||||||||||||||||||||||||||||||||||||||||||||||||

Sbjct 61 ACATCGAGAACCTGATCGACAAGCTCGGCACCCTCAGTGTAGTGACCCTTGGCCCAGTTG 120

Query 121 TTGCCGGCACCGGATTGACCGAAAACGAAGTTGTCGGGTCGGAAAAGCTGGCCGAAAGGA 180

||||||||||||||||||||||||||||||||||||||||||||||||||||||||||||

Sbjct 121 TTGCCGGCACCGGATTGACCGAAAACGAAGTTGTCGGGTCGGAAAAGCTGGCCGAAAGGA 180

Query 181 CCGGCACGGACAGCGTCCATGGTACCAGGCTCAAGATCGACGAGGACGGCGCGAGGAACA 240

||||||||||||||||||||||||||||||||||||||||||||||||||||||||||||

Sbjct 181 CCGGCACGGACAGCGTCCATGGTACCAGGCTCAAGATCGACGAGGACGGCGCGAGGAACA 240

Query 241 TACTTGTTGCCAGAGGCCTAGGTGTGTGAGCTCGGATTTTCTAAAGATGGGCTGATTAAC 300

||||||||||||||||||||||||||||||||||||||||||||||||||||||||||||

Sbjct 241 TACTTGTTGCCAGAGGCCTAGGTGTGTGAGCTCGGATTTTCTAAAGATGGGCTGATTAAC 300

Query 301 ACATACCTCGTTGAAGTAGACGCTCATACGCTCGAGCTGGAGCTCGGAGGTACCGTTGTA 360

||||||||||||||||||||||||||||||||||||||||||||||||||||||||||||

Sbjct 301 ACATACCTCGTTGAAGTAGACGCTCATACGCTCGAGCTGGAGCTCGGAGGTACCGTTGTA 360

Query 361 GACACCATTGCTGTCGAGACCGTGCTCGCCAGAGATGGTCTGCCAGAAAGCAGCACCAAT 420

||||||||||||||||||||||||||||||||||||||||||||||||||||||||||||

Sbjct 361 GACACCATTGCTGTCGAGACCGTGCTCGCCAGAGATGGTCTGCCAGAAAGCAGCACCAAT 420

Query 421 TTGGTTACCCTAATAAACATTGTGAGCATTCCCCCGCACACGACAGTTGAAGAGGTGAGT 480

||||||||||||||||||||||||||||||||||||||||||||||||||||||||||||

Sbjct 421 TTGGTTACCCTAATAAACATTGTGAGCATTCCCCCGCACACGACAGTTGAAGAGGTGAGT 480

Query 481 ACTTACGCACTGACCGGTCTGAAGGTGAACCTATTGCAGAGAGAAAATATTAGGTTAGCC 540

||||||||||||||||||||||||||||||||||||||||||||||||||||||||||||

Sbjct 481 ACTTACGCACTGACCGGTCTGAAGGTGAACCTATTGCAGAGAGAAAATATTAGGTTAGCC 540

Query 541 TCATGTTCCTGAAGGTTATCGTTGATCAAGCTGCGAGCTATCGTGCGTTGTTGTTGAGCT 600

||||||||||||||||||||||||||||||||||||||||||||||||||||||||||||

Sbjct 541 TCATGTTCCTGAAGGTTATCGTTGATCAAGCTGCGAGCTATCGTGCGTTGTTGTTGAGCT 600

Query 601 TCCACCGCCCAGCGGGGTA 619

|||||||||||||||||||

Sbjct 601 TCCACCGCCCAGCGGGGTA 619

RID: N8M92KUP11R

Job Title:Fusarium_PT_ITS

Program: BLASTN

Query: Fusarium_PT_ITS ID: lcl|Query_428357(dna) Length: 1456

Subject:Fusarium_lateritium_NRRL13622 ID: lcl|Query_428359(dna) Length: 1456

Sequences producing significant alignments:

Scientific Common Max Total Query E Per. Acc.

Description Name Name Taxid Score Score cover Value Ident Len Accession

Fusarium_lateritium_NRRL13622 0 2689 2689 100% 0.0 100.00 1456 Query_428359

Alignments:

>Fusarium_lateritium_NRRL13622

Sequence ID: Query_428359 Length: 1456

Range 1: 1 to 1456

Score:2689 bits(1456), Expect:0.0,

Identities:1456/1456(100%), Gaps:0/1456(0%), Strand: Plus/Plus

Query 1 GTCGTTGATGACCATTACGCCAGCATCCTTGCAGATGCGCGAACCTCAGTCCCCGCCAGG 60

||||||||||||||||||||||||||||||||||||||||||||||||||||||||||||

Sbjct 1 GTCGTTGATGACCATTACGCCAGCATCCTTGCAGATGCGCGAACCTCAGTCCCCGCCAGG 60

Query 61 GTATTACACAATGGGCTATAACACTCCCCGAGGGGAGCCACATTCCCAAAGTCTTTATCC 120

||||||||||||||||||||||||||||||||||||||||||||||||||||||||||||

Sbjct 61 GTATTACACAATGGGCTATAACACTCCCCGAGGGGAGCCACATTCCCAAAGTCTTTATCC 120

Query 121 CCCGGCGAAAACTGATGCTGGCCTGAACTGGAGAAGTGCACTGGGGAGAACCCCAGATGA 180

||||||||||||||||||||||||||||||||||||||||||||||||||||||||||||

Sbjct 121 CCCGGCGAAAACTGATGCTGGCCTGAACTGGAGAAGTGCACTGGGGAGAACCCCAGATGA 180

Query 181 TTAACCAAGTCCAAGTCTGGTCACAAACGCTTCCCTTTCAACAATTTCACGTACTTTTTA 240

||||||||||||||||||||||||||||||||||||||||||||||||||||||||||||

Sbjct 181 TTAACCAAGTCCAAGTCTGGTCACAAACGCTTCCCTTTCAACAATTTCACGTACTTTTTA 240

Query 241 ACTCTCTTTTCAAAGTGCTTTTCATCTTTCGATCACTCTACTTGTGCGCTATCGGTCTCT 300

||||||||||||||||||||||||||||||||||||||||||||||||||||||||||||

Sbjct 241 ACTCTCTTTTCAAAGTGCTTTTCATCTTTCGATCACTCTACTTGTGCGCTATCGGTCTCT 300

Query 301 GGCCGGTATTTAGCTTTAGAAGACATATACCTCCCATTTAGAGCAGCATTCCCAAACTAC 360

||||||||||||||||||||||||||||||||||||||||||||||||||||||||||||

Sbjct 301 GGCCGGTATTTAGCTTTAGAAGACATATACCTCCCATTTAGAGCAGCATTCCCAAACTAC 360

Query 361 TCGACTCGTTGAAGGAGTTTTACAGAGATTTGGCATCCAACCAGACGGGGCTCTCACCCT 420

||||||||||||||||||||||||||||||||||||||||||||||||||||||||||||

Sbjct 361 TCGACTCGTTGAAGGAGTTTTACAGAGATTTGGCATCCAACCAGACGGGGCTCTCACCCT 420

Query 421 CTATGGCGTCCCGTTCCAGGGAACTCGGAAGGCACCGCATCAAAAACATCCTCTACAAAT 480

||||||||||||||||||||||||||||||||||||||||||||||||||||||||||||

Sbjct 421 CTATGGCGTCCCGTTCCAGGGAACTCGGAAGGCACCGCATCAAAAACATCCTCTACAAAT 480

Query 481 TACAACTCGGGCCCGAGAGCCAGATTTCAAATTTGAGCTGTTGCCGCTTCACTCGCCGTT 540

||||||||||||||||||||||||||||||||||||||||||||||||||||||||||||

Sbjct 481 TACAACTCGGGCCCGAGAGCCAGATTTCAAATTTGAGCTGTTGCCGCTTCACTCGCCGTT 540

Query 541 ACTAGGGCAATCCCTGTTGGTTTCTTTTCCTCCGCTTATTGATATGCTTAAGTTCAGCGG 600

||||||||||||||||||||||||||||||||||||||||||||||||||||||||||||

Sbjct 541 ACTAGGGCAATCCCTGTTGGTTTCTTTTCCTCCGCTTATTGATATGCTTAAGTTCAGCGG 600

Query 601 GTATTCCTACCTGATCCGAGGTCAACATTCAGAAGTTGGGTGTTTAACGGCATGGCCGCG 660

||||||||||||||||||||||||||||||||||||||||||||||||||||||||||||

Sbjct 601 GTATTCCTACCTGATCCGAGGTCAACATTCAGAAGTTGGGTGTTTAACGGCATGGCCGCG 660

Query 661 CCGCGTTCCAGTTGCGAGGTGTTAGCTACTACGCAATGGAGGCTGCAGCGAGACCGCCAC 720

||||||||||||||||||||||||||||||||||||||||||||||||||||||||||||

Sbjct 661 CCGCGTTCCAGTTGCGAGGTGTTAGCTACTACGCAATGGAGGCTGCAGCGAGACCGCCAC 720

Query 721 TAGATTTCGGAGACGGATTGACTAGGCAATCCGATCCCCAACACCAAACCCGGGGGTTTG 780

||||||||||||||||||||||||||||||||||||||||||||||||||||||||||||

Sbjct 721 TAGATTTCGGAGACGGATTGACTAGGCAATCCGATCCCCAACACCAAACCCGGGGGTTTG 780

Query 781 AGGGTTGAAATGACGCTCGAACAGGCATGCCCGCCAGAATACTGGCGGGCGCAATGTGCG 840

||||||||||||||||||||||||||||||||||||||||||||||||||||||||||||

Sbjct 781 AGGGTTGAAATGACGCTCGAACAGGCATGCCCGCCAGAATACTGGCGGGCGCAATGTGCG 840

Query 841 TTCAAAGATTCGATGATTCACTGAATTCTGCAATTCACATTACTTATCGCATTTTGCTGC 900

||||||||||||||||||||||||||||||||||||||||||||||||||||||||||||

Sbjct 841 TTCAAAGATTCGATGATTCACTGAATTCTGCAATTCACATTACTTATCGCATTTTGCTGC 900

Query 901 GTTCTTCATCGATGCCAGAACCAAGAGATCCGTTGTTGAAAGTTTTGATTTATTTGTTTG 960

||||||||||||||||||||||||||||||||||||||||||||||||||||||||||||

Sbjct 901 GTTCTTCATCGATGCCAGAACCAAGAGATCCGTTGTTGAAAGTTTTGATTTATTTGTTTG 960

Query 961 TGTTACTCAGAAGATACACTAAATACAATAGAGTTTGGGTTCCTCTGGCGGGCCGTCCCG 1020

||||||||||||||||||||||||||||||||||||||||||||||||||||||||||||

Sbjct 961 TGTTACTCAGAAGATACACTAAATACAATAGAGTTTGGGTTCCTCTGGCGGGCCGTCCCG 1020

Query 1021 TTTTACGGGGCGCGGGCTGATCCGCCGAGGCAACAATAAGGTATGTTCACAGGGGTTTGG 1080

||||||||||||||||||||||||||||||||||||||||||||||||||||||||||||

Sbjct 1021 TTTTACGGGGCGCGGGCTGATCCGCCGAGGCAACAATAAGGTATGTTCACAGGGGTTTGG 1080

Query 1081 GAGTTGTAAACTCGGTAATGATCCCTCCGCTGGTTCACCAACGGAGACCTTGTTACGACT 1140

||||||||||||||||||||||||||||||||||||||||||||||||||||||||||||

Sbjct 1081 GAGTTGTAAACTCGGTAATGATCCCTCCGCTGGTTCACCAACGGAGACCTTGTTACGACT 1140

Query 1141 TTTACTTCCTCTAAATGACCGAGTTTGGAGAGCTTTCCGGCCCTGAGTGGTAGTTGCCCA 1200

||||||||||||||||||||||||||||||||||||||||||||||||||||||||||||

Sbjct 1141 TTTACTTCCTCTAAATGACCGAGTTTGGAGAGCTTTCCGGCCCTGAGTGGTAGTTGCCCA 1200

Query 1201 CCTCTCTGGGCCAGTCCGGACGCCTCACTGAGCCATTCAATCGGTAGTAGCGACGGGCGG 1260

||||||||||||||||||||||||||||||||||||||||||||||||||||||||||||

Sbjct 1201 CCTCTCTGGGCCAGTCCGGACGCCTCACTGAGCCATTCAATCGGTAGTAGCGACGGGCGG 1260

Query 1261 TGTGTACAAAGGGCAGGGACGTAATCAACGCAAGCTGATGACTTGCGCTTACTAGGGATT 1320

||||||||||||||||||||||||||||||||||||||||||||||||||||||||||||

Sbjct 1261 TGTGTACAAAGGGCAGGGACGTAATCAACGCAAGCTGATGACTTGCGCTTACTAGGGATT 1320

Query 1321 CCTCGTTGAAGAGCAATAATTGCAATGCTCTATCCCCAGCACGACGGAGTTTAACAAGAT 1380

||||||||||||||||||||||||||||||||||||||||||||||||||||||||||||

Sbjct 1321 CCTCGTTGAAGAGCAATAATTGCAATGCTCTATCCCCAGCACGACGGAGTTTAACAAGAT 1380

Query 1381 TACCCGGACCTTTCGGACAAGGAAGTACTCGCTGGCTCCGTCAGTGTAGCGCGCGTGCGG 1440

||||||||||||||||||||||||||||||||||||||||||||||||||||||||||||

Sbjct 1381 TACCCGGACCTTTCGGACAAGGAAGTACTCGCTGGCTCCGTCAGTGTAGCGCGCGTGCGG 1440

Query 1441 CCCAGAACATCTAAGG 1456

||||||||||||||||

Sbjct 1441 CCCAGAACATCTAAGG 1456

RID: N8ME1MA711R

Job Title:Fusarium_sp_PT

Program: BLASTN

Query: Fusarium_sp_PT ID: lcl|Query_454035(dna) Length: 913

Subject:Fusarium_lateritium_NRRL13622 ID: lcl|Query_454037(dna) Length: 913

Sequences producing significant alignments:

Scientific Common Max Total Query E Per. Acc.

Description Name Name Taxid Score Score cover Value Ident Len Accession

Fusarium_lateritium_NRRL13622 0 1592 1592 100% 0.0 98.14 913 Query_454037

Alignments:

>Fusarium_lateritium_NRRL13622

Sequence ID: Query_454037 Length: 913

Range 1: 1 to 913

Score:1592 bits(862), Expect:0.0,

Identities:896/913(98%), Gaps:0/913(0%), Strand: Plus/Plus

Query 1 TCCTGAGGGAAACTTCGGCGGAAACCAGCTACTAGAAGGTTCGATTAGTCTTTCGCCCCC 60

||||||||||||||||||||||||||||||||||||||||||||||||||||||||||||

Sbjct 1 TCCTGAGGGAAACTTCGGCGGAAACCAGCTACTAGAAGGTTCGATTAGTCTTTCGCCCCC 60

Query 61 ATGCCCATATTTGACGATCGATTTGCACGTCAGAACCGCTGCGAGCCTCCACCAGAGTTT 120

||||||||||||||||||||||||||||||||||||||||||||||||||||||||||||

Sbjct 61 ATGCCCATATTTGACGATCGATTTGCACGTCAGAACCGCTGCGAGCCTCCACCAGAGTTT 120

Query 121 CCTCTGGCTTCACCCTATACAGGCATAGTTCACCTTCTTTCGGGTCCGGCCCCGTATGCT 180

||||||||||||||||||||||||||||||||||||||||||||||||||||||||||||

Sbjct 121 CCTCTGGCTTCACCCTATACAGGCATAGTTCACCTTCTTTCGGGTCCGGCCCCGTATGCT 180

Query 181 CTTACTCAAATCCATCCGAGAACATCAGGATCGGTCGATGATGCGCCGAAGCTCTCACCT 240

||||||||||||||||||||||||||||||||||||||||||||||||||||||||||||

Sbjct 181 CTTACTCAAATCCATCCGAGAACATCAGGATCGGTCGATGATGCGCCGAAGCTCTCACCT 240

Query 241 GCGTTCACTTTCATTACGCGTAGGGGTTTGACACCCGAACACTCGCATACGAAGACGACT 300

||||||||||||||||||||||||||||||||||||||||||||||||||||||||||||

Sbjct 241 GCGTTCACTTTCATTACGCGTAGGGGTTTGACACCCGAACACTCGCATACGAAGACGACT 300

Query 301 CCTTGGTCCGTGTTTCAAGACGGGTCGTTGATGACCATTACGCCAGCATCCTTGCAGAAG 360

|||||||||||||||||||||||||||||||||||||||||||||||||||||||||| |

Sbjct 301 CCTTGGTCCGTGTTTCAAGACGGGTCGTTGATGACCATTACGCCAGCATCCTTGCAGATG 360

Query 361 CGCGAACCTCAGTCCCCACCAGGGTATTACACAACGGGCTATAACACTCCCCGAAGAGAG 420

||||||||||||||||| |||||||||||||||| ||||||||||||||||||| | |||

Sbjct 361 CGCGAACCTCAGTCCCCGCCAGGGTATTACACAATGGGCTATAACACTCCCCGAGGGGAG 420

Query 421 CCACATTCCCGCTGCCTTTATCCCCCGGCGAAAACTGATGCTGGCCTGAACTGGAGAAGT 480

|||||||||| | |||||||||||||||||||||||||||||||||||||||||||||

Sbjct 421 CCACATTCCCAAAGTCTTTATCCCCCGGCGAAAACTGATGCTGGCCTGAACTGGAGAAGT 480

Query 481 GCACTGGGGAGAACCCCAGATGATTAACCAAGTCCAAGTCTGGTCATAAACGCTTCCCTT 540

|||||||||||||||||||||||||||||||||||||||||||||| |||||||||||||

Sbjct 481 GCACTGGGGAGAACCCCAGATGATTAACCAAGTCCAAGTCTGGTCACAAACGCTTCCCTT 540

Query 541 TCAACAATTTCACGTACTTTTTAACTCTCTTTTCAAAGTGCTTTTCATCTTTCGATCACT 600

||||||||||||||||||||||||||||||||||||||||||||||||||||||||||||

Sbjct 541 TCAACAATTTCACGTACTTTTTAACTCTCTTTTCAAAGTGCTTTTCATCTTTCGATCACT 600

Query 601 CTACTTGTGCGCTATCGGTCTCTGGCCAATATTTAGCTTTAGAAGACATATACCTCCCAT 660

||||||||||||||||||||||||||| |||||||||||||||||||||||||||||||

Sbjct 601 CTACTTGTGCGCTATCGGTCTCTGGCCGGTATTTAGCTTTAGAAGACATATACCTCCCAT 660

Query 661 TTAGAGCAGCATTCCCAAACTACTCGACTCGTCGAAGGAACTTTACAGAGATTTGGCATC 720

|||||||||||||||||||||||||||||||| |||||| |||||||||||||||||||

Sbjct 661 TTAGAGCAGCATTCCCAAACTACTCGACTCGTTGAAGGAGTTTTACAGAGATTTGGCATC 720

Query 721 CAACCAGACGGGGCTCTCACCCTCTATGGCGTCCCGTTCCAGGGAACTCGGAAGGCACCG 780

||||||||||||||||||||||||||||||||||||||||||||||||||||||||||||

Sbjct 721 CAACCAGACGGGGCTCTCACCCTCTATGGCGTCCCGTTCCAGGGAACTCGGAAGGCACCG 780

Query 781 CATCAAAAGTATCCTCTACAAATTACAACTCGGGCCCGAGAGCCAGATTTCAAATTTGAG 840

|||||||| ||||||||||||||||||||||||||||||||||||||||||||||||||

Sbjct 781 CATCAAAAACATCCTCTACAAATTACAACTCGGGCCCGAGAGCCAGATTTCAAATTTGAG 840

Query 841 CTGTTGCCGCTTCACTCGCCGTTACTAGGGCAATCCCTGTTGGTTTCTTTTCCTCCGCTT 900

||||||||||||||||||||||||||||||||||||||||||||||||||||||||||||

Sbjct 841 CTGTTGCCGCTTCACTCGCCGTTACTAGGGCAATCCCTGTTGGTTTCTTTTCCTCCGCTT 900

Query 901 ATTGATATGCTTA 913

|||||||||||||

Sbjct 901 ATTGATATGCTTA 913

**2 BLASTn pairwise alignment between the *Fusarium* sp. PT strain and *Fusarium tricinctum INRA104***

RID: N8N1ZBS5114

Job Title:Fusarium_PT_Tef1a

Program: BLASTN

Query: Fusarium_PT_Tef1a ID: lcl|Query_54633(dna) Length: 678

Subject:Fusarium_tricinctum_INRA104 ID: lcl|Query_54635(dna) Length: 682

Sequences producing significant alignments:

Scientific Common Max Total Query E Per. Acc.

Description Name Name Taxid Score Score cover Value Ident Len Accession

Fusarium_tricinctum_INRA104 0 996 996 100% 0.0 93.13 682 Query_54635

Alignments:

>Fusarium_tricinctum_INRA104

Sequence ID: Query_54635 Length: 682

Range 1: 1 to 682

Score:996 bits(539), Expect:0.0,

Identities:637/684(93%), Gaps:8/684(1%), Strand: Plus/Plus

Query 1 AAGACTCACCTTAACGTCGTCGTCATCGGCCACGTCGACTCTGGCAAGTCGACCACTGTA 60

||||||||||||||||||||||||||||||||||||||||||||||||||||||||||||

Sbjct 1 AAGACTCACCTTAACGTCGTCGTCATCGGCCACGTCGACTCTGGCAAGTCGACCACTGTA 60

Query 61 AGTTCAACCATCAGCGAGTTGCTTATCTGCACTCG-AGCCTGCCACATCTGGCGGGGGTA 119

||| |||||||||||| ||||||||| |||||||| | | |||| | ||||||||| ||

Sbjct 61 AGTACAACCATCAGCGGGTTGCTTATATGCACTCGGAATCCGCCAAACCTGGCGGGG-TA 119

Query 120 CTACCGCAACACTTTGCTAACTTTTGACAGACCGGTCACTTGATCTACCAGTGCGGTGGT 179

||| ||||| ||||||||||||||||||||||||||||||||||||||||||||||||

Sbjct 120 TCACCACAACATTTTGCTAACTTTTGACAGACCGGTCACTTGATCTACCAGTGCGGTGGT 179

Query 180 ATCGACAAGCGAACCATCGAGAAGTTCGAGAAGGTTAGTCATTATCCCTTCGATTCCGCG 239

||||||||||||||||||||||||||||||||||||||||| ||||||||||||| ||||

Sbjct 180 ATCGACAAGCGAACCATCGAGAAGTTCGAGAAGGTTAGTCAATATCCCTTCGATTACGCG 239

Query 240 CGCTCCCATCGAATCCTACGACTCGCTCCATCACTCGAATCGCATCCATTACCCCGCTCG 299

|||||||||||| ||| |||| ||||||| ||||||||| ||||||||||||||||||||

Sbjct 240 CGCTCCCATCGATTCCCACGATTCGCTCCCTCACTCGAAACGCATCCATTACCCCGCTCG 299

Query 300 AGTCCGAAAATTTTGCGGTGCGACCGTGAATTCTTTTTGGTGGGGTATCTTACCCCGCCA 359

|| |||| ||||||||||||||||||||| || ||| |||||||||||||||||||||||

Sbjct 300 AGCCCGAGAATTTTGCGGTGCGACCGTGATTTTTTTCTGGTGGGGTATCTTACCCCGCCA 359

Query 360 CTCGAGTGACGGATGCGCTTGCCCTGTTCCCACAAAATTTCACTACCCTGCCGCGCACCA 419

||||||||||||||||||||||||||||||||||||| | || |||||| |||||| |

Sbjct 360 CTCGAGTGACGGATGCGCTTGCCCTGTTCCCACAAAACCTTACCACCCTGTCGCGCA-CT 418

Query 420 ACATGTCTTGCAGTCACTAACCATTGGACAATAGGAAGCCGCCGAGCTCGGAAAGGGTTC 479

||||||||||||||||||||||| ||||||||||||||||||||||||||||||||||||

Sbjct 419 ACATGTCTTGCAGTCACTAACCACTGGACAATAGGAAGCCGCCGAGCTCGGAAAGGGTTC 478

Query 480 CTTCAAGTATGCCTGGGTTCTTGACAAGCTCAAAGCCGAGCGTGAGCGTGGTATCACCAT 539

||||||||| ||||||||||||||||||||||||||||||||||||||||||||||||||

Sbjct 479 CTTCAAGTACGCCTGGGTTCTTGACAAGCTCAAAGCCGAGCGTGAGCGTGGTATCACCAT 538

Query 540 TGATATCGCTCTCTGGAAGTTCGAGACTCCTCGCTACTATGTCACCGTCATTGGTATGTT 599

||||||||||||||||||||||||||||||||||||||||||||||||||||||||||||

Sbjct 539 TGATATCGCTCTCTGGAAGTTCGAGACTCCTCGCTACTATGTCACCGTCATTGGTATGTT 598

Query 600 GTTACTGTCTCACGCCACCATGCCT-C--CATGCTAACCTCTC--TCAGATGCCCCCGGT 654

|| |||||||||| | | ||||| | | ||||||||| |||| |||||||||||||||

Sbjct 599 GTCACTGTCTCACACTATCATGCTTGCATCATGCTAACATCTCTGTCAGATGCCCCCGGT 658

Query 655 CATCGTGACTTCATCAAGAACATG 678

|||||||| |||||||||||||||

Sbjct 659 CATCGTGATTTCATCAAGAACATG 682

RID: N8N3SGES114

Job Title:Fusarium_PT_RPB1

Program: BLASTN

Query: Fusarium_PT_RPB1 ID: lcl|Query_28949(dna) Length: 1604

Subject:Fusarium_tricinctum_INRA104 ID: lcl|Query_28951(dna) Length: 1604

Sequences producing significant alignments:

Scientific Common Max Total Query E Per. Acc.

Description Name Name Taxid Score Score cover Value Ident Len Accession

Fusarium_tricinctum_INRA104 0 2420 2420 100% 0.0 93.89 1604 Query_28951

Alignments:

>Fusarium_tricinctum_INRA104

Sequence ID: Query_28951 Length: 1604

Range 1: 1 to 1604

Score:2420 bits(1310), Expect:0.0,

Identities:1506/1604(94%), Gaps:0/1604(0%), Strand: Plus/Plus

Query 1 TTCCTCACCAAGGAACAGATCATGAACTGCATGCTCTGGGTGCCCAACTGGGACGGTGTC 60

|||||||||||||||||||||||||| |||||||||||||||||||||||||| |||||

Sbjct 1 TTCCTCACCAAGGAACAGATCATGAATTGCATGCTCTGGGTGCCCAACTGGGATGGTGTT 60

Query 61 ATTCCTCAGCCCGCTATCTATAAACCTCGTCCTCGTTGGACCGGTAAGCAGCTCATCAGC 120

|||||||| |||||||||||||| || |||||||||||||| ||||||||||| || |||

Sbjct 61 ATTCCTCAACCCGCTATCTATAAGCCCCGTCCTCGTTGGACTGGTAAGCAGCTTATTAGC 120

Query 121 ATGGTCATCCCTAAGGAGGTTAGCCTTTTTAATGGTACGGACGGTAATGAGAATGCCCCT 180

||||| |||||||||||||||||||| || || |||||||||| |||||||||||||||

Sbjct 121 ATGGTTATCCCTAAGGAGGTTAGCCTCTTCAACGGTACGGACGATAATGAGAATGCCCCC 180

Query 181 CTTAGGGATGAGGGTCTTTTGATTCAATCCGGCCAACTAATGTACGGTCTTCTCACCAAG 240

|||||||||||||||||| |||| || ||||| ||||| ||||||||||||||||| |||

Sbjct 181 CTTAGGGATGAGGGTCTTCTGATCCAGTCCGGTCAACTTATGTACGGTCTTCTCACAAAG 240

Query 241 AAGAGCGTTGGTGCTGCTGCTGGTGGTATCGTTCATATTAGTTACAATGAGCTGGGACCT 300

|||||||||||||||||||||||||||||||||||||| ||||| || ||| ||||||||

Sbjct 241 AAGAGCGTTGGTGCTGCTGCTGGTGGTATCGTTCATATCAGTTATAACGAGTTGGGACCT 300

Query 301 GAGGGAGCTATGGCTTTCTTGAACGGTGTCCAGCAGGTTGTGACTTACTGGCTTCTGAAC 360

||||||||||||||||||||||||||||| ||||| || ||||||||||||||| |||||

Sbjct 301 GAGGGAGCTATGGCTTTCTTGAACGGTGTTCAGCAAGTCGTGACTTACTGGCTTTTGAAC 360

Query 361 AACGGTCACAGTATTGGTATTGGTGACACAGTTCCTGATGCTGTTACTATTGCCAAGGTT 420

|||||||||||||||||||||||||||||| |||||||||||| |||||||| ||||||

Sbjct 361 AACGGTCACAGTATTGGTATTGGTGACACAATTCCTGATGCTGCCACTATTGCAAAGGTT 420

Query 421 CAGGCGCATATTGATGAGGAAAAGGATGAAGTTGCTCGTTTGACAGCGATGGCCACTGCC 480

|||| |||||| |||||||| |||| |||||| ||||| |||||||||||||||||||||

Sbjct 421 CAGGTGCATATCGATGAGGAGAAGGCTGAAGTCGCTCGCTTGACAGCGATGGCCACTGCC 480

Query 481 AACGAGCTTGAGGCCCTTCCCGGTATGAACGTTCGTGCAACATTCGAGAACAAGGTCTCT 540

|| ||||||||||| || || |||||||||||||||||||||||||| || |||||||||

Sbjct 481 AATGAGCTTGAGGCGCTCCCTGGTATGAACGTTCGTGCAACATTCGAAAATAAGGTCTCT 540

Query 541 ATGGCTCTGAACCAAGCCCGTGACAAGGCTGGTACGACAACACAGAAGAGTTTAAAGGAT 600

|||||||| ||||| |||||||||||||||||||||||||||||||||||||| || |||

Sbjct 541 ATGGCTCTCAACCAGGCCCGTGACAAGGCTGGTACGACAACACAGAAGAGTTTGAAAGAT 600

Query 601 TCAAACAACGCTGTTACCATGGCTTCTTCAGGTTCCAAGGGTTCTTCTATCAACATTTCA 660

||||||||||||||||||||||||||||||||||||||||||||||||||||||||||||

Sbjct 601 TCAAACAACGCTGTTACCATGGCTTCTTCAGGTTCCAAGGGTTCTTCTATCAACATTTCA 660

Query 661 CAGATGACTGCCCTTGTCGGTCAGCAAATTGTTGAAGGCAAGCGTATTCCTTTCGGTTTC 720

||||||||||| ||||||||||||||||||||||||||||| ||||||||||||||||||

Sbjct 661 CAGATGACTGCTCTTGTCGGTCAGCAAATTGTTGAAGGCAAACGTATTCCTTTCGGTTTC 720

Query 721 AAGTACCGTACCCTGCCTCATTTCACCAAGGACGATTACTCACCCGAAGCTCGTGGTTTC 780

|||||||| |||||||||||||||||||||||||||||||||||||||||||||||||||

Sbjct 721 AAGTACCGCACCCTGCCTCATTTCACCAAGGACGATTACTCACCCGAAGCTCGTGGTTTC 780

Query 781 GTTGAGAACTCCTACCTTCGTGGTCTTACCCCCAGCGAGTTCTTTTTCCACGCCATGGCT 840

||||||||||| |||||||||||||||||||||||||| ||||| |||||||||||||||

Sbjct 781 GTTGAGAACTCTTACCTTCGTGGTCTTACCCCCAGCGAATTCTTCTTCCACGCCATGGCT 840

Query 841 GGTCGAGAAGGTCTCATTGATACTGCTGTCAAGACTGCCGAAACAGGTTACATTCAGCGA 900

|||||||||||||||||||||||||| |||||||||||||||||||||||||||||||||

Sbjct 841 GGTCGAGAAGGTCTCATTGATACTGCGGTCAAGACTGCCGAAACAGGTTACATTCAGCGA 900

Query 901 CGATTGGTCAAGGCTTTGGAAGATCTTTCTGCGCGCTATGATGGAACTGTTCGAAACTCT 960

||||||||||||||| |||||||||||||||||||||| || ||||| ||||||||||||

Sbjct 901 CGATTGGTCAAGGCTCTGGAAGATCTTTCTGCGCGCTACGACGGAACGGTTCGAAACTCT 960

Query 961 CTGGGTGATATTGTTCAATTCCTTTACGGCGAGGATGGTCTCGATGCTATGATCATTGAG 1020

|||||||||||||||||||||| ||||||||||||||||||||||||||||||||||||

Sbjct 961 TTGGGTGATATTGTTCAATTCCTGTACGGCGAGGATGGTCTCGATGCTATGATCATTGAG 1020

Query 1021 AAGCAGAAGCTGGGAATTCTCAACATGTCCAACAGTGCATTCGAAAAGAAGTACCGTCTG 1080

||||||||||||||||||||||||||||| |||||||||||||||||||| |||||| ||

Sbjct 1021 AAGCAGAAGCTGGGAATTCTCAACATGTCGAACAGTGCATTCGAAAAGAAATACCGTTTG 1080

Query 1081 GATCTTGCCAACCCTCCTGATTGGTTTAGGCACGACTACGAATTCGGCAACGAGTTGACT 1140

||||||||||||||||||||||||||||||||||||||||||||||| ||||||||||||

Sbjct 1081 GATCTTGCCAACCCTCCTGATTGGTTTAGGCACGACTACGAATTCGGTAACGAGTTGACT 1140

Query 1141 GGTGACAGAGCTTCCATGAGCCTTCTCGATGAAGAATGGGAGGCTCTGCGATACGATCGC 1200

|||||||||||||||||||||||||||||||| |||||||||||||||||||| ||||||

Sbjct 1141 GGTGACAGAGCTTCCATGAGCCTTCTCGATGAGGAATGGGAGGCTCTGCGATATGATCGC 1200

Query 1201 AGAAGGATCCGGGATATCAATAGGTCAAAGGGTAACGAGGAGATGATGCAATTACCTCTC 1260

| |||||| || ||||| |||||||||||||||||||||||||||||| ||||||

Sbjct 1201 AAAAGGATTCGCCTCATCAACCAGTCAAAGGGTAACGAGGAGATGATGCAATTGCCTCTC 1260

Query 1261 AACATCACCCGCATCATCGAGTCTGCCAAGCGTGTATTCAATGTCAAGGCCAATGACCGA 1320

|| ||||| || ||||| |||||||||||||||||||||||||||||||| |||||||||

Sbjct 1261 AATATCACTCGTATCATTGAGTCTGCCAAGCGTGTATTCAATGTCAAGGCTAATGACCGA 1320

Query 1321 AGTAACTTGCGACCCTCGGATGTTATCCCCGGAGTGAGAAACATGTTGGAAAACATGAAG 1380

|||||| ||||||||||||||||||||||||||||||| |||||||||||||||||||||

Sbjct 1321 AGTAACCTGCGACCCTCGGATGTTATCCCCGGAGTGAGGAACATGTTGGAAAACATGAAG 1380

Query 1381 ATCGTTCGAGGTACTGACGAGATTTCATTGGAGGCTGACGCCAACGCCTCCATTCTCTTC 1440

|||||||| |||||||| ||||||||||||||||||||||||||||||||||||||||||

Sbjct 1381 ATCGTTCGGGGTACTGATGAGATTTCATTGGAGGCTGACGCCAACGCCTCCATTCTCTTC 1440

Query 1441 AAGGCCCTCCTCCGTTCCCGACTAGCCTTCAAGGAAGTTGTCAAGGAGCACCGCTTGAAT 1500

|||||||| ||||| ||||||||||||||||||||||||||||||||||||||||||||

Sbjct 1441 AAGGCCCTTCTCCGCTCCCGACTAGCCTTCAAGGAAGTTGTCAAGGAGCACCGCTTGAAC 1500

Query 1501 AAGCTGGCTTTCGATTACATCTTGGGTGAACTCCAGAATAGATGGGACCGTGCCTTCGTC 1560

|||||||||||||| |||||||||||||| |||||||| |||||||||||||| |||||

Sbjct 1501 AAGCTGGCTTTCGACTACATCTTGGGTGAGCTCCAGAACAGATGGGACCGTGCTTTCGTG 1560

Query 1561 AACCCTGGTGAAATGGTTGGTGTTCTGGCTGCTCAGTCTATTGG 1604

|||||||||||||||||||| ||| ||||||| |||||||||||

Sbjct 1561 AACCCTGGTGAAATGGTTGGCGTTTTGGCTGCCCAGTCTATTGG 1604

RID: N8N9Z335114

Job Title:Fusarium_sp_PT_RPB2

Program: BLASTN

Query: Fusarium_sp_PT ID: lcl|Query_46749(dna) Length: 1856

Subject:Fusarium_tricinctum_INRA104 ID: lcl|Query_46751(dna) Length: 1856

Sequences producing significant alignments:

Scientific Common Max Total Query E Per. Acc.

Description Name Name Taxid Score Score cover Value Ident Len Accession

Fusarium_tricinctum_INRA104 0 1827 2818 96% 0.0 96.23 1856 Query_46751

Alignments:

>Fusarium_tricinctum_INRA104

Sequence ID: Query_46751 Length: 1856

Range 1: 742 to 1856

Score:1827 bits(989), Expect:0.0,

Identities:1073/1115(96%), Gaps:0/1115(0%), Strand: Plus/Plus

Query 742 TTGAAGACAAAGGTCAATCCTACAACTCACGCGTACACACATTGCGAGATTCATCCCAGT 801

||||||||||||||||||||||| ||||||| |||||||||||| |||||||||||||||

Sbjct 742 TTGAAGACAAAGGTCAATCCTACGACTCACGTGTACACACATTGTGAGATTCATCCCAGT 801

Query 802 ATGATTCTTGGTATCTGTGCCAGTATCATTCCCTTCCCTGATCACAACCAGGTATGTCAT 861

|||||||||||||||||||||||||||||||||||||| ||||||||||||||||||||

Sbjct 802 ATGATTCTTGGTATCTGTGCCAGTATCATTCCCTTCCCCGATCACAACCAGGTATGTCAG 861

Query 862 GACGCTTGATTTTCTTCGATCTTCACTAACAATACACAGTCTCCTCGTAACACCTACCAA 921

||||||| ||||| | |||| | ||||||||||||||||||||||||||||||||||||

Sbjct 862 GACGCTTAATTTTGGTTGATCCTTACTAACAATACACAGTCTCCTCGTAACACCTACCAA 921

Query 922 TCTGCCATGGGTAAACAAGCTATGGGTTTCTTCTTAACAAACTATTCTCGACGTATGGAT 981

||||||||||||||||||||||||||||||||||||||||||||||||||||||||||||

Sbjct 922 TCTGCCATGGGTAAACAAGCTATGGGTTTCTTCTTAACAAACTATTCTCGACGTATGGAT 981

Query 982 ACCATGGCCAACATTCTCTACTACCCTCAAAAGCCCCTCGCCACTACTCGATCTATGGAG 1041

||||||||||||||||||||||||||||||||||||||||||||||||||||||||||||

Sbjct 982 ACCATGGCCAACATTCTCTACTACCCTCAAAAGCCCCTCGCCACTACTCGATCTATGGAG 1041

Query 1042 CATCTCAAGTTCCGTGAACTGCCAGCTGGTCAAAACGCTATCGTTGCAATTGCCTGTTAC 1101

|||||||||||||||||||||||||||||||||||||| ||||| |||||||||||||||

Sbjct 1042 CATCTCAAGTTCCGTGAACTGCCAGCTGGTCAAAACGCCATCGTCGCAATTGCCTGTTAC 1101

Query 1102 TCAGGATACAACCAGGAAGATTCCGTTATTATGAATCAGAGCAGTATTGATAGAGGTCTA 1161

||||||||||||||||||||||||||||||||||| ||||||||||||||||||||||||

Sbjct 1102 TCAGGATACAACCAGGAAGATTCCGTTATTATGAACCAGAGCAGTATTGATAGAGGTCTA 1161

Query 1162 TTCCGTAGTCTGTTCTTCCGATCGTACTCAGATCAGGAGAAGAAGGTCGGTCTCAACTAC 1221

||||| ||||||||||||||||||||||||||||||||||||||||||||||||||||||

Sbjct 1162 TTCCGCAGTCTGTTCTTCCGATCGTACTCAGATCAGGAGAAGAAGGTCGGTCTCAACTAC 1221

Query 1222 ACAGAAATATTTGAGAAGCCTTTCCAGCAGACAACACTTCGAATGAAGCACGGAACATAC 1281

|||||||| |||||||||||||||||||||||||||||||||||||||||||||||||||

Sbjct 1222 ACAGAAATCTTTGAGAAGCCTTTCCAGCAGACAACACTTCGAATGAAGCACGGAACATAC 1281

Query 1282 GACAAGCTTGACGAAGATGGTATTGTGGCTCCTGGTGTACGAGTTTCTGGTGAAGATATC 1341

||||||||||||||||||||||| ||||| ||||||||||||||||| ||||||||||||

Sbjct 1282 GACAAGCTTGACGAAGATGGTATCGTGGCCCCTGGTGTACGAGTTTCCGGTGAAGATATC 1341

Query 1342 ATCATTGGCAAGACGGCGCCCATCGATCAGGAGAATCAGGATCTGGGTACCAGAACCCAA 1401

|| |||||||||||||||||||||||||||||||| ||||||||||| ||||||||||||

Sbjct 1342 ATTATTGGCAAGACGGCGCCCATCGATCAGGAGAACCAGGATCTGGGCACCAGAACCCAA 1401

Query 1402 TCGCACCAGCGTCGTGATATCTCGACACCGCTGCGAAGTACAGAAAACGGTATCGTTGAT 1461

|||||||||||||| |||||||||||||||||||||||||||||||||||||||||||||

Sbjct 1402 TCGCACCAGCGTCGCGATATCTCGACACCGCTGCGAAGTACAGAAAACGGTATCGTTGAT 1461

Query 1462 CAAGTCATTCTGACAGTCAACGCCGACAACGTGAAGTACGTGAAGGTTCGAGTACGAACC 1521

||||||||| ||||||||||||||||||||||||||||||| |||||||| |||||||||

Sbjct 1462 CAAGTCATTTTGACAGTCAACGCCGACAACGTGAAGTACGTCAAGGTTCGTGTACGAACC 1521

Query 1522 ACCAAGATTCCCCAGATTGGTGACAAGTTTGCTTCTCGTCACGGTCAGAAGGGTACCATT 1581

||||||||||||||||||||||| ||||||||||||||||||||||| ||||||||||||

Sbjct 1522 ACCAAGATTCCCCAGATTGGTGATAAGTTTGCTTCTCGTCACGGTCAAAAGGGTACCATT 1581

Query 1582 GGTGTCACTTATCGTCAGGAAGATATGCCTTTCAGCAGAGAAGGCCTGACTCCCGATATT 1641

|||||||||||||| ||||| ||||||||||||||||||||||||||||||||||||||

Sbjct 1582 GGTGTCACTTATCGACAGGAGGATATGCCTTTCAGCAGAGAAGGCCTGACTCCCGATATC 1641

Query 1642 ATCATCAACCCTCACGCTATTCCCTCTCGAATGACAATTGCCCATTTGATTGAGTGTCTT 1701

|| |||||||||||||||||||||||||||||||||||||||||||||||||||||||||

Sbjct 1642 ATTATCAACCCTCACGCTATTCCCTCTCGAATGACAATTGCCCATTTGATTGAGTGTCTT 1701

Query 1702 CTTAGTAAGGTCTCAACACTTGAAGGTATGGAGGGTGATGCAACACCGTTCACCGACGTG 1761

|||||||||||||||||||||||||||||||||||||||||||| || ||||| || |||

Sbjct 1702 CTTAGTAAGGTCTCAACACTTGAAGGTATGGAGGGTGATGCAACGCCATTCACTGATGTG 1761

Query 1762 ACGGTCGACTCCGTCTCAGACCTTCTAAGAAAGCACGGCTACCAATCTCGAGGCTTCGAG 1821

|| |||||||| |||||||| ||||||||||||||||| ||||||||||||||||| |||

Sbjct 1762 ACAGTCGACTCTGTCTCAGAGCTTCTAAGAAAGCACGGATACCAATCTCGAGGCTTTGAG 1821

Query 1822 GTCATGTACAATGGTCACACTGGACGCAAGCTCCG 1856

|||||||||||||| ||||||||||||||||||||

Sbjct 1822 GTCATGTACAATGGCCACACTGGACGCAAGCTCCG 1856

Range 2: 1 to 674

Score:990 bits(536), Expect:0.0,

Identities:628/674(93%), Gaps:0/674(0%), Strand: Plus/Plus

Query 1 CCCATCGGACGAGATGGTAAGCTGGCCAAACCTCGTCAGCTACACAACACCCATTGGGGT 60

||||||||||||||||||||||||||||||||||||||||||||||||||||||||||||

Sbjct 1 CCCATCGGACGAGATGGTAAGCTGGCCAAACCTCGTCAGCTACACAACACCCATTGGGGT 60

Query 61 TTAGTGTGTCCCGCAGAAACACCTGAGGGACAGGCTTGTGGTCTGGTCAAGAACCTGTCA 120

|| |||||||| |||||||| ||||||||||||||||||||||||||||||||| ||||

Sbjct 61 TTGGTGTGTCCTGCAGAAACGCCTGAGGGACAGGCTTGTGGTCTGGTCAAGAACTTGTCT 120

Query 121 CTCATGTGTTACGTGAGTGTGGGTTCTCCTGCTGATCCTCTGATTGATTTCATGATCCAC 180

|| |||||||||||||||||||| ||||||||||||||||||||||| ||||||||||||

Sbjct 121 CTGATGTGTTACGTGAGTGTGGGCTCTCCTGCTGATCCTCTGATTGACTTCATGATCCAC 180

Query 181 AGAGGTATGGAAGTGGTTGAGGAGTATGAGCCAACAAGATACCCACACGCTACCAAGATT 240

||||||||||||||||||||||||||||||||||||||||||||||||||||||||||||

Sbjct 181 AGAGGTATGGAAGTGGTTGAGGAGTATGAGCCAACAAGATACCCACACGCTACCAAGATT 240

Query 241 TTCGTCAACGGTAGCTGGGTTGGTGTTCACTCTGACCCCAAGCACCTTGTGGACCAGGTT 300

|||||||||||||||||||| ||||||||||||||||||||||| |||||| |||| |||

Sbjct 241 TTCGTCAACGGTAGCTGGGTCGGTGTTCACTCTGACCCCAAGCATCTTGTGCACCAAGTT 300

Query 301 CTGTCCACCCGACGAAAGAATGTTGTTCAATTCGAAGTCTCACTTGTTCGTGATATTCGA 360

||||||||||| |||||||||| |||||||| ||||| |||||||||||||||||||||

Sbjct 301 TTGTCCACCCGAAGAAAGAATGTCGTTCAATTTGAAGTGTCACTTGTTCGTGATATTCGA 360

Query 361 GACCGAGAATTCAAGATCTTTTCCGATGCAGGCCGAGTCATGAGACCGGTCTTCACAGTA 420

|||||||||||||||||||| || ||||||||| ||||||||||||| ||||| |||||

Sbjct 361 GACCGAGAATTCAAGATCTTCTCTGATGCAGGCAGAGTCATGAGACCAGTCTTTACAGTG 420

Query 421 CAACAGGAGGACGACGATGAGACAGGTTTCCAAAAGGGACAACTTATACTGACCAAGGAT 480

|| |||||||| ||||| ||||| ||| | || |||||||| |||||||||||||||||

Sbjct 421 CAGCAGGAGGATGACGACGAGACTGGTGTTCAGAAGGGACAGCTTATACTGACCAAGGAG 480

Query 481 CTGATTACCAAGCTTGCCCAAGAGCAGGCCGAGCCATCTGATAATCCATCAGAGAAGCTC 540

||| |||||||||| |||||||||||||| |||||||||||| ||||||||||||||||

Sbjct 481 CTGGTTACCAAGCTCGCCCAAGAGCAGGCGGAGCCATCTGATGATCCATCAGAGAAGCTT 540

Query 541 GGCTGGGAGGGTCTTGTTCGCGCTGGAGTTATCGAGTATCTCGATGCCGAGGAAGAAGAA 600

|||||||||||||||||||| |||||||||||||||||||||||||| ||||||||||||

Sbjct 541 GGCTGGGAGGGTCTTGTTCGTGCTGGAGTTATCGAGTATCTCGATGCTGAGGAAGAAGAA 600

Query 601 ACGGCCATGATCTGCATGACGCCCGAGGATCTCGAAATTTTCCGCGAGCAAAAGAATGAT 660

|||||||||||||||||||| ||||||||||| ||| ||| ||||||||||||||||||

Sbjct 601 ACGGCCATGATCTGCATGACACCCGAGGATCTTGAACTTTACCGCGAGCAAAAGAATGAC 660

Query 661 GAGATGACCCTCAC 674

||| |||||||||

Sbjct 661 GAGGCGACCCTCAC 674

RID: N8NDHAYB114

Job Title:Fusarium_PT_beta_tub

Program: BLASTN

Query: Fusarium_PT_beta_tub ID: lcl|Query_55055(dna) Length: 619

Subject:Fusarium_tricinctum_INRA104 ID: lcl|Query_55057(dna) Length: 623

Sequences producing significant alignments:

Scientific Common Max Total Query E Per. Acc.

Description Name Name Taxid Score Score cover Value Ident Len Accession

Fusarium_tricinctum_INRA104 0 632 632 100% 0.0 85.21 623 Query_55057

Alignments:

>Fusarium_tricinctum_INRA104

Sequence ID: Query_55057 Length: 623

Range 1: 1 to 623

Score:632 bits(342), Expect:0.0,

Identities:536/629(85%), Gaps:16/629(2%), Strand: Plus/Plus

Query 1 AGAGAGTGGGTGATCTGGAAACCCTGGAGGCAGTCACAGCCCTCTGCCTCACGACGGACG 60

|||||||||||||||||||| |||||||||||||| |||||||| |||||||| ||||||

Sbjct 1 AGAGAGTGGGTGATCTGGAAGCCCTGGAGGCAGTCGCAGCCCTCGGCCTCACGGCGGACG 60

Query 61 ACATCGAGAACCTGATCGACAAGCTCGGCACCCTCAGTGTAGTGACCCTTGGCCCAGTTG 120

|||||||| ||||| ||||| ||||| || ||||| ||||| |||||||| |||||||||

Sbjct 61 ACATCGAGGACCTGGTCGACGAGCTCAGCTCCCTCGGTGTAATGACCCTTCGCCCAGTTG 120

Query 121 TTGCCGGCACCGGATTGACCGAAAACGAAGTTGTCGGGTCGGAAAAGCTGGCCGAAAGGA 180

|| || ||||| || || |||||||||||||||||||||||||||||||| |||||||||

Sbjct 121 TTTCCAGCACCAGACTGGCCGAAAACGAAGTTGTCGGGTCGGAAAAGCTGACCGAAAGGA 180

Query 181 CCGGCACGGACAGCGTCCATGGTACCAGGCTCAAGATCGACGAGGACGGCGCGAGGAACA 240

|| || ||||| || ||||||||||||||||| | ||||||||||||||| ||||| ||

Sbjct 181 CCAGCGCGGACGGCATCCATGGTACCAGGCTCCAAATCGACGAGGACGGCACGAGGGACG 240

Query 241 TACTTGTTGCCAGAGGCCTAGGTGTGTGAGC-T-CGGATTTTCTAAAGATGGGCTGATTA 298

||||||||||| || ||||| ||| || ||| | | | ||| || || ||| |

Sbjct 241 TACTTGTTGCCGGAAGCCTATGTGCGTCAGCCTACAG-TTTCCTTGCAATCAACTGTCTG 299

Query 299 ACACATACCTCGTTGAAGTAGACGCTCATACGCTCGAGCTGGAGCTCGGAGGTACCGTTG 358

||||||||||||||||||||||||||| ||||||||||| ||||| ||||| ||||||

Sbjct 300 CAACATACCTCGTTGAAGTAGACGCTCATGCGCTCGAGCTGAAGCTCCGAGGTGCCGTTG 359

Query 359 TAGACACCATTGCTGTCGAGACCGTGCTCGCCAGAGATGGTCTGCCAGAAAGCAGCACCA 418

|||||||||||||||||||| || ||||||||||||||||||||||||||||||||||||

Sbjct 360 TAGACACCATTGCTGTCGAGGCCATGCTCGCCAGAGATGGTCTGCCAGAAAGCAGCACCA 419

Query 419 ATTTGGTTACCCTAATAAACATTGTGAGCATTCCCCCGCAC--ACGACAGTTGAAGAGGT 476

|||||||||||||||| |||||| | |||| |||| || | |||| ||| || |

Sbjct 420 ATTTGGTTACCCTAATCAACATTCTTAGCAA-CCCCT-CATGGATGACA-TTGGAGCAAT 476

Query 477 GAGTACTTACGCACTGACCGGTCTGAAGGTGAACCTATTGCAGAGAGAAAATATTAGGTT 536

|||||||||||||||||||||||||||| ||||||||||||| | ||||||||| ||||

Sbjct 477 GAGTACTTACGCACTGACCGGTCTGAAGATGAACCTATTGCA-A-AGAAAATATATGGTT 534

Query 537 AGCCTCATGTTCCTG-A-A-GGTTATCG-TTGA--TCAAGCTGCGAGCTATCGTGCGTTG 590

|| |||||||||| | | | |||| | | || | ||||||||| ||||||||||| |||

Sbjct 535 AGTCTCATGTTCCAGCACAAGGTTGTTGATTAAGCTCAAGCTGCAAGCTATCGTGCATTG 594

Query 591 TTGTTGAGCTTCCACCGCCCAGCGGGGTA 619

|||| ||||| | ||| ||||||||||||

Sbjct 595 TTGTCGAGCTGCTACCACCCAGCGGGGTA 623

RID: N8NEH4JM114

Job Title:Fusarium_PT_ITS

Program: BLASTN

Query: Fusarium_PT_ITS ID: lcl|Query_35839(dna) Length: 1456

Subject:Fusarium_tricinctum_INRA104 ID: lcl|Query_35841(dna) Length: 1461

Sequences producing significant alignments:

Scientific Common Max Total Query E Per. Acc.

Description Name Name Taxid Score Score cover Value Ident Len Accession

Fusarium_tricinctum_INRA104 0 2427 2427 100% 0.0 96.72 1461 Query_35841

Alignments:

>Fusarium_tricinctum_INRA104

Sequence ID: Query_35841 Length: 1461

Range 1: 1 to 1461

Score:2427 bits(1314), Expect:0.0,

Identities:1415/1463(97%), Gaps:9/1463(0%), Strand: Plus/Plus

Query 1 GTCGTTGATGACCATTACGCCAGCATCCTTGCAGATGCGCGAACCTCAGTCCCCGCCAGG 60

||||||||||||||||||||||||||||||||||| ||||||||||||||| ||||||||

Sbjct 1 GTCGTTGATGACCATTACGCCAGCATCCTTGCAGAAGCGCGAACCTCAGTCTCCGCCAGG 60

Query 61 GTATTACACAATGGGCTATAACACTCCCCGAGGGGAGCCACATTCCCAAAGTCTTTATCC 120

||||||||||| ||||||||||||||||||| | |||| |||||||| ||| ||||||||

Sbjct 61 GTATTACACAACGGGCTATAACACTCCCCGAAGAGAGCTACATTCCCGAAGCCTTTATCC 120

Query 121 CCCGGCGAAAACTGATGCTGGCCTGAACTGGAGAAGTGCACTGGGGAGAACCCCAGATGA 180

|||||||||||||||||||||||||||||||| |||||||||||||||||||||||||||

Sbjct 121 CCCGGCGAAAACTGATGCTGGCCTGAACTGGAAAAGTGCACTGGGGAGAACCCCAGATGA 180

Query 181 TTAACCAAGTCCAAGTCTGGTCACAAACGCTTCCCTTTCAACAATTTCACGTACTTTTTA 240

||||||||||||||||||||||| ||||||||||||||||||||||||||||||||||||

Sbjct 181 TTAACCAAGTCCAAGTCTGGTCATAAACGCTTCCCTTTCAACAATTTCACGTACTTTTTA 240

Query 241 ACTCTCTTTTCAAAGTGCTTTTCATCTTTCGATCACTCTACTTGTGCGCTATCGGTCTCT 300

||||||||||||||||||||||||||||||||||||||||||||||||||||||||||||

Sbjct 241 ACTCTCTTTTCAAAGTGCTTTTCATCTTTCGATCACTCTACTTGTGCGCTATCGGTCTCT 300

Query 301 GGCCGGTATTTAGCTTTAGAAGACATATACCTCCCATTTAGAGCAGCATTCCCAAACTAC 360

|||| ||||||||||||||||||||||||||||||||||||||||||||||||||||||

Sbjct 301 GGCCAATATTTAGCTTTAGAAGACATATACCTCCCATTTAGAGCAGCATTCCCAAACTAC 360

Query 361 TCGACTCGTTGAAGGAGTTTTACAGAGATTTGGCATCCAACCAGACGGGGCTCTCACCCT 420

||||||||| |||||| ||||||||||||||||||||||||||||||||||||||||||

Sbjct 361 TCGACTCGTCGAAGGAACTTTACAGAGATTTGGCATCCAACCAGACGGGGCTCTCACCCT 420

Query 421 CTATGGCGTCCCGTTCCAGGGAACTCGGAAGGCACCGCATCAAAAACATCCTCTACAAAT 480

||||||||||||||||||||||||||||||||||||||||||||| |||||||||||||

Sbjct 421 CTATGGCGTCCCGTTCCAGGGAACTCGGAAGGCACCGCATCAAAAGTATCCTCTACAAAT 480

Query 481 TACAACTCGGGCCCGAGAGCCAGATTTCAAATTTGAGCTGTTGCCGCTTCACTCGCCGTT 540

|||||||||||||||| |||||||||||||||||||||||||||||||||||||||||||

Sbjct 481 TACAACTCGGGCCCGAAAGCCAGATTTCAAATTTGAGCTGTTGCCGCTTCACTCGCCGTT 540

Query 541 ACTAGGGCAATCCCTGTTGGTTTCTTTTCCTCCGCTTATTGATATGCTTAAGTTCAGCGG 600

||||||||||||||||||||||||||||||||||||||||||||||||||||||||||||

Sbjct 541 ACTAGGGCAATCCCTGTTGGTTTCTTTTCCTCCGCTTATTGATATGCTTAAGTTCAGCGG 600

Query 601 GTATTCCTACCTGATCCGAGGTCAACATTCAGAAGTTGGGTGTTTAACGGCATGGCCGCG 660

|||||||||||||||||||||||||||||||||||||||| |||| ||||||||||||||

Sbjct 601 GTATTCCTACCTGATCCGAGGTCAACATTCAGAAGTTGGG-GTTTTACGGCATGGCCGCG 659

Query 661 CCGCGTTCCAGTTGCGAGGTGTTAGCTACTACGCAATGGAGGCTGCAGCGAGACCGCCAC 720

|||||||||||||||||||||||||||||||||||||||||||||||||||||||||||

Sbjct 660 CCGCGTTCCAGTTGCGAGGTGTTAGCTACTACGCAATGGAGGCTGCAGCGAGACCGCCAA 719

Query 721 TAGATTTCGGAGACGG-ATTG---ACTA-GGCA-ATCCGATCCCCAACACCAAACCCGGG 774

| ||||||| | ||| | | | | |||| | ||||||||||||||||||||||||

Sbjct 720 TGTATTTCGGGGGCGGCACCGCCCAGAAGGGCAGAGCCGATCCCCAACACCAAACCCGGG 779

Query 775 GGTTTGAGGGTTGAAATGACGCTCGAACAGGCATGCCCGCCAGAATACTGGCGGGCGCAA 834

|| |||||||||||||||||||||||||||||||||||||| |||||| ||||||||||

Sbjct 780 GGCTTGAGGGTTGAAATGACGCTCGAACAGGCATGCCCGCCGGAATACCAGCGGGCGCAA 839

Query 835 TGTGCGTTCAAAGATTCGATGATTCACTGAATTCTGCAATTCACATTACTTATCGCATTT 894

||||||||||||||||||||||||||||||||||||||||||||||||||||||||||||

Sbjct 840 TGTGCGTTCAAAGATTCGATGATTCACTGAATTCTGCAATTCACATTACTTATCGCATTT 899

Query 895 TGCTGCGTTCTTCATCGATGCCAGAACCAAGAGATCCGTTGTTGAAAGTTTTGATTTATT 954

||||||||||||||||||||||||||||||||||||||||||||||||||||||||||||

Sbjct 900 TGCTGCGTTCTTCATCGATGCCAGAACCAAGAGATCCGTTGTTGAAAGTTTTGATTTATT 959

Query 955 TGTTTGTGTTACTCAGAAGATACACTAA-ATACAATAGAGTTTGGGTTCCTCTGGCGGGC 1013

||||||| ||||||||||| |||| ||| | ||| ||||||||||| |||||||||||||

Sbjct 960 TGTTTGTTTTACTCAGAAGTTACAATAAGAAACATTAGAGTTTGGG-TCCTCTGGCGGGC 1018

Query 1014 CGTCCCGTTTTACGGGGCGCGGGCTGATCCGCCGAGGCAACAATAAGGTATGTTCACAGG 1073

|||||||||||||||||||||||||||||||||||||||||| |||||||||||||||||

Sbjct 1019 CGTCCCGTTTTACGGGGCGCGGGCTGATCCGCCGAGGCAACATTAAGGTATGTTCACAGG 1078

Query 1074 GGTTTGGGAGTTGTAAACTCGGTAATGATCCCTCCGCTGGTTCACCAACGGAGACCTTGT 1133

||||||||||||||||||||||||||||||||||||||||||||||||||||||||||||

Sbjct 1079 GGTTTGGGAGTTGTAAACTCGGTAATGATCCCTCCGCTGGTTCACCAACGGAGACCTTGT 1138

Query 1134 TACGACTTTTACTTCCTCTAAATGACCGAGTTTGGAGAGCTTTCCGGCCCTGAGTGGTAG 1193

||||||||||||||||||||||||||||||||||||||||||||||||||||||||||||

Sbjct 1139 TACGACTTTTACTTCCTCTAAATGACCGAGTTTGGAGAGCTTTCCGGCCCTGAGTGGTAG 1198

Query 1194 TTGCCCACCTCTCTGGGCCAGTCCGGACGCCTCACTGAGCCATTCAATCGGTAGTAGCGA 1253

||||||||||||||||||||||||||||||||||||||||||||||||||||||||||||

Sbjct 1199 TTGCCCACCTCTCTGGGCCAGTCCGGACGCCTCACTGAGCCATTCAATCGGTAGTAGCGA 1258

Query 1254 CGGGCGGTGTGTACAAAGGGCAGGGACGTAATCAACGCAAGCTGATGACTTGCGCTTACT 1313

||||||||||||||||||||||||||||||||||||||||||||||||||||||||||||

Sbjct 1259 CGGGCGGTGTGTACAAAGGGCAGGGACGTAATCAACGCAAGCTGATGACTTGCGCTTACT 1318

Query 1314 AGGGATTCCTCGTTGAAGAGCAATAATTGCAATGCTCTATCCCCAGCACGACGGAGTTTA 1373

||||||||||||||||||||||||||||||||||||||||||||||||||||||||||||

Sbjct 1319 AGGGATTCCTCGTTGAAGAGCAATAATTGCAATGCTCTATCCCCAGCACGACGGAGTTTA 1378

Query 1374 ACAAGATTACCCGGACCTTTCGGACAAGGAAGTACTCGCTGGCTCCGTCAGTGTAGCGCG 1433

||||||||||||||||||||||||||||||||||||||||||||||||||||||||||||

Sbjct 1379 ACAAGATTACCCGGACCTTTCGGACAAGGAAGTACTCGCTGGCTCCGTCAGTGTAGCGCG 1438

Query 1434 CGTGCGGCCCAGAACATCTAAGG 1456

|||||||||||||||||||||||

Sbjct 1439 CGTGCGGCCCAGAACATCTAAGG 1461

RID: N8NGYBF9114

Job Title:Fusarium_sp_PT LSU

Program: BLASTN

Query: Fusarium_sp_PT ID: lcl|Query_26983(dna) Length: 913

Subject:Fusarium_tricinctum_INRA104 ID: lcl|Query_26985(dna) Length: 913

Sequences producing significant alignments:

Scientific Common Max Total Query E Per. Acc.

Description Name Name Taxid Score Score cover Value Ident Len Accession

Fusarium_tricinctum_INRA104 0 1648 1648 100% 0.0 99.23 913 Query_26985

Alignments:

>Fusarium_tricinctum_INRA104

Sequence ID: Query_26985 Length: 913

Range 1: 1 to 913

Score:1648 bits(892), Expect:0.0,

Identities:906/913(99%), Gaps:0/913(0%), Strand: Plus/Plus

Query 1 TCCTGAGGGAAACTTCGGCGGAAACCAGCTACTAGAAGGTTCGATTAGTCTTTCGCCCCC 60

||||||||||||||||||||||||||||||||||||||||||||||||||||||||||||

Sbjct 1 TCCTGAGGGAAACTTCGGCGGAAACCAGCTACTAGAAGGTTCGATTAGTCTTTCGCCCCC 60

Query 61 ATGCCCATATTTGACGATCGATTTGCACGTCAGAACCGCTGCGAGCCTCCACCAGAGTTT 120

||||||||||||||||||||||||||||||||||||||||||||||||||||||||||||

Sbjct 61 ATGCCCATATTTGACGATCGATTTGCACGTCAGAACCGCTGCGAGCCTCCACCAGAGTTT 120

Query 121 CCTCTGGCTTCACCCTATACAGGCATAGTTCACCTTCTTTCGGGTCCGGCCCCGTATGCT 180

||||||||||||||||||||||||||||||||||||||||||||||||||||||||||||

Sbjct 121 CCTCTGGCTTCACCCTATACAGGCATAGTTCACCTTCTTTCGGGTCCGGCCCCGTATGCT 180

Query 181 CTTACTCAAATCCATCCGAGAACATCAGGATCGGTCGATGATGCGCCGAAGCTCTCACCT 240

||||||||||||||||||||||||||||||||||||||||||||||||||||||||||||

Sbjct 181 CTTACTCAAATCCATCCGAGAACATCAGGATCGGTCGATGATGCGCCGAAGCTCTCACCT 240

Query 241 GCGTTCACTTTCATTACGCGTAGGGGTTTGACACCCGAACACTCGCATACGAAGACGACT 300

||||||||||||||||||||||||||||||||||||||||||||||||||||||||||||

Sbjct 241 GCGTTCACTTTCATTACGCGTAGGGGTTTGACACCCGAACACTCGCATACGAAGACGACT 300

Query 301 CCTTGGTCCGTGTTTCAAGACGGGTCGTTGATGACCATTACGCCAGCATCCTTGCAGAAG 360

||||||||||||||||||||||||||||||||||||||||||||||||||||||||||||

Sbjct 301 CCTTGGTCCGTGTTTCAAGACGGGTCGTTGATGACCATTACGCCAGCATCCTTGCAGAAG 360

Query 361 CGCGAACCTCAGTCCCCACCAGGGTATTACACAACGGGCTATAACACTCCCCGAAGAGAG 420

|||||||||||||| || ||||||||||||||||||||||||||||||||||||||||||

Sbjct 361 CGCGAACCTCAGTCTCCGCCAGGGTATTACACAACGGGCTATAACACTCCCCGAAGAGAG 420

Query 421 CCACATTCCCGCTGCCTTTATCCCCCGGCGAAAACTGATGCTGGCCTGAACTGGAGAAGT 480

| ||||||||| |||||||||||||||||||||||||||||||||||||||||| ||||

Sbjct 421 CTACATTCCCGAAGCCTTTATCCCCCGGCGAAAACTGATGCTGGCCTGAACTGGAAAAGT 480

Query 481 GCACTGGGGAGAACCCCAGATGATTAACCAAGTCCAAGTCTGGTCATAAACGCTTCCCTT 540

||||||||||||||||||||||||||||||||||||||||||||||||||||||||||||

Sbjct 481 GCACTGGGGAGAACCCCAGATGATTAACCAAGTCCAAGTCTGGTCATAAACGCTTCCCTT 540

Query 541 TCAACAATTTCACGTACTTTTTAACTCTCTTTTCAAAGTGCTTTTCATCTTTCGATCACT 600

||||||||||||||||||||||||||||||||||||||||||||||||||||||||||||

Sbjct 541 TCAACAATTTCACGTACTTTTTAACTCTCTTTTCAAAGTGCTTTTCATCTTTCGATCACT 600

Query 601 CTACTTGTGCGCTATCGGTCTCTGGCCAATATTTAGCTTTAGAAGACATATACCTCCCAT 660

||||||||||||||||||||||||||||||||||||||||||||||||||||||||||||

Sbjct 601 CTACTTGTGCGCTATCGGTCTCTGGCCAATATTTAGCTTTAGAAGACATATACCTCCCAT 660

Query 661 TTAGAGCAGCATTCCCAAACTACTCGACTCGTCGAAGGAACTTTACAGAGATTTGGCATC 720

||||||||||||||||||||||||||||||||||||||||||||||||||||||||||||

Sbjct 661 TTAGAGCAGCATTCCCAAACTACTCGACTCGTCGAAGGAACTTTACAGAGATTTGGCATC 720

Query 721 CAACCAGACGGGGCTCTCACCCTCTATGGCGTCCCGTTCCAGGGAACTCGGAAGGCACCG 780

||||||||||||||||||||||||||||||||||||||||||||||||||||||||||||

Sbjct 721 CAACCAGACGGGGCTCTCACCCTCTATGGCGTCCCGTTCCAGGGAACTCGGAAGGCACCG 780

Query 781 CATCAAAAGTATCCTCTACAAATTACAACTCGGGCCCGAGAGCCAGATTTCAAATTTGAG 840

||||||||||||||||||||||||||||||||||||||| ||||||||||||||||||||

Sbjct 781 CATCAAAAGTATCCTCTACAAATTACAACTCGGGCCCGAAAGCCAGATTTCAAATTTGAG 840

Query 841 CTGTTGCCGCTTCACTCGCCGTTACTAGGGCAATCCCTGTTGGTTTCTTTTCCTCCGCTT 900

||||||||||||||||||||||||||||||||||||||||||||||||||||||||||||

Sbjct 841 CTGTTGCCGCTTCACTCGCCGTTACTAGGGCAATCCCTGTTGGTTTCTTTTCCTCCGCTT 900

Query 901 ATTGATATGCTTA 913

|||||||||||||

Sbjct 901 ATTGATATGCTTA 913
