# Supplementary material for: Draft Genome Sequence of a New Fusarium Isolate Belonging to Fusarium tricinctum Species Complex Collected From Hazelnut in Central Italy
Source: Front Plant Sci. 2021 Dec 16;12:788584. doi: 10.3389/fpls.2021.788584 (PMC8718101; doi:10.3389/fpls.2021.788584)
Supplement: Supplementary Figure 1 — Phylogenetic tree of RPB1 and RPB2 concatenated sequences among FTSC strains. The nucleotide sequence of RPB1 and RPB2 genes of a selection of 63 strains belonging to the FTSC were concatenated, aligned, and used to build a ML tree using RAxML, from which only bootstraps higher than 60 are shown. [file Data_Sheet_1.zip › Supplementary Dataset 3.docx]

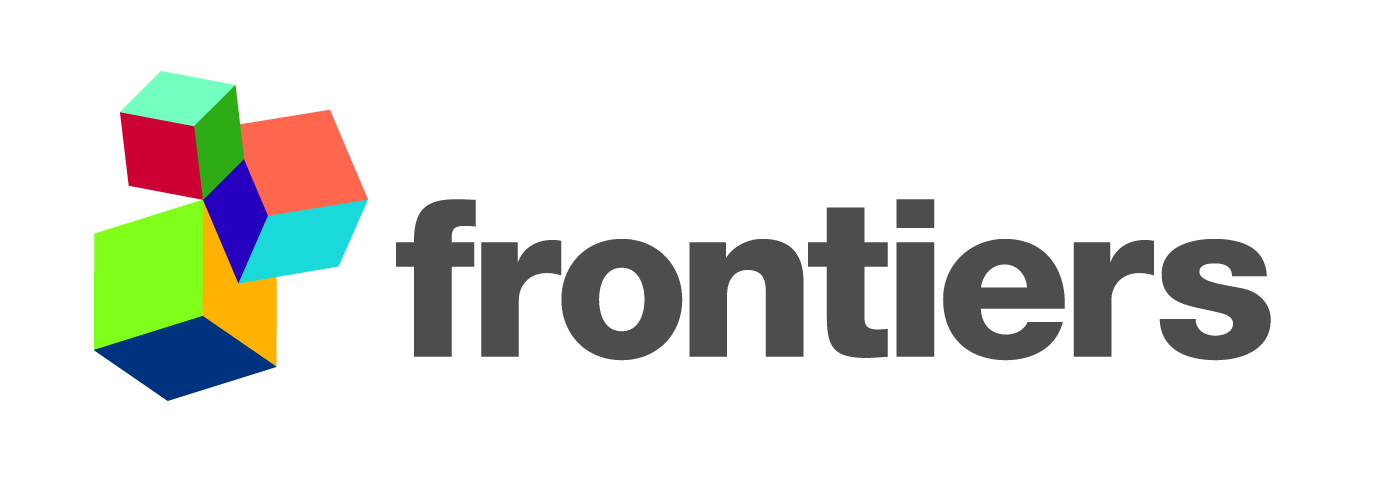


**Supplementary Dataset 3**

**Transmembrane proteins predicted by TMHMM2**

# Fusarium_sp-PT_008831-RA Length: 453

# Fusarium_sp-PT_008831-RA Number of predicted TMHs: 0

# Fusarium_sp-PT_008831-RA Exp number of AAs in TMHs: 1.11928

# Fusarium_sp-PT_008831-RA Exp number, first 60 AAs: 1.11585

# Fusarium_sp-PT_008831-RA Total prob of N-in: 0.05403

Fusarium_sp-PT_008831-RA TMHMM2.0 outside 1 453

# Fusarium_sp-PT_008832-RA Length: 534

# Fusarium_sp-PT_008832-RA Number of predicted TMHs: 0

# Fusarium_sp-PT_008832-RA Exp number of AAs in TMHs: 0.33345

# Fusarium_sp-PT_008832-RA Exp number, first 60 AAs: 0

# Fusarium_sp-PT_008832-RA Total prob of N-in: 0.01713

Fusarium_sp-PT_008832-RA TMHMM2.0 outside 1 534

# Fusarium_sp-PT_008833-RA Length: 330

# Fusarium_sp-PT_008833-RA Number of predicted TMHs: 0

# Fusarium_sp-PT_008833-RA Exp number of AAs in TMHs: 0.10597

# Fusarium_sp-PT_008833-RA Exp number, first 60 AAs: 0

# Fusarium_sp-PT_008833-RA Total prob of N-in: 0.00433

Fusarium_sp-PT_008833-RA TMHMM2.0 outside 1 330

# Fusarium_sp-PT_008835-RA Length: 859

# Fusarium_sp-PT_008835-RA Number of predicted TMHs: 0

# Fusarium_sp-PT_008835-RA Exp number of AAs in TMHs: 0.01431

# Fusarium_sp-PT_008835-RA Exp number, first 60 AAs: 0.00101

# Fusarium_sp-PT_008835-RA Total prob of N-in: 0.00073

Fusarium_sp-PT_008835-RA TMHMM2.0 outside 1 859

# Fusarium_sp-PT_008837-RA Length: 412

# Fusarium_sp-PT_008837-RA Number of predicted TMHs: 0

# Fusarium_sp-PT_008837-RA Exp number of AAs in TMHs: 2.99336

# Fusarium_sp-PT_008837-RA Exp number, first 60 AAs: 1.0815

# Fusarium_sp-PT_008837-RA Total prob of N-in: 0.01761

Fusarium_sp-PT_008837-RA TMHMM2.0 outside 1 412

# Fusarium_sp-PT_008838-RA Length: 92

# Fusarium_sp-PT_008838-RA Number of predicted TMHs: 0

# Fusarium_sp-PT_008838-RA Exp number of AAs in TMHs: 0

# Fusarium_sp-PT_008838-RA Exp number, first 60 AAs: 0

# Fusarium_sp-PT_008838-RA Total prob of N-in: 0.20770

Fusarium_sp-PT_008838-RA TMHMM2.0 outside 1 92

# Fusarium_sp-PT_008840-RA Length: 996

# Fusarium_sp-PT_008840-RA Number of predicted TMHs: 0

# Fusarium_sp-PT_008840-RA Exp number of AAs in TMHs: 0.30853

# Fusarium_sp-PT_008840-RA Exp number, first 60 AAs: 0.00186

# Fusarium_sp-PT_008840-RA Total prob of N-in: 0.01412

Fusarium_sp-PT_008840-RA TMHMM2.0 outside 1 996

# Fusarium_sp-PT_008841-RA Length: 493

# Fusarium_sp-PT_008841-RA Number of predicted TMHs: 1

# Fusarium_sp-PT_008841-RA Exp number of AAs in TMHs: 21.96698

# Fusarium_sp-PT_008841-RA Exp number, first 60 AAs: 21.96443

# Fusarium_sp-PT_008841-RA Total prob of N-in: 0.99977

# Fusarium_sp-PT_008841-RA POSSIBLE N-term signal sequence

Fusarium_sp-PT_008841-RA TMHMM2.0 inside 1 12

Fusarium_sp-PT_008841-RA TMHMM2.0 TMhelix 13 35

Fusarium_sp-PT_008841-RA TMHMM2.0 outside 36 493

# Fusarium_sp-PT_008842-RA Length: 372

# Fusarium_sp-PT_008842-RA Number of predicted TMHs: 1

# Fusarium_sp-PT_008842-RA Exp number of AAs in TMHs: 22.2698399999999

# Fusarium_sp-PT_008842-RA Exp number, first 60 AAs: 22.26518

# Fusarium_sp-PT_008842-RA Total prob of N-in: 0.80896

# Fusarium_sp-PT_008842-RA POSSIBLE N-term signal sequence

Fusarium_sp-PT_008842-RA TMHMM2.0 inside 1 12

Fusarium_sp-PT_008842-RA TMHMM2.0 TMhelix 13 35

Fusarium_sp-PT_008842-RA TMHMM2.0 outside 36 372

# Fusarium_sp-PT_008844-RA Length: 692

# Fusarium_sp-PT_008844-RA Number of predicted TMHs: 0

# Fusarium_sp-PT_008844-RA Exp number of AAs in TMHs: 0.08499

# Fusarium_sp-PT_008844-RA Exp number, first 60 AAs: 0

# Fusarium_sp-PT_008844-RA Total prob of N-in: 0.00120

Fusarium_sp-PT_008844-RA TMHMM2.0 outside 1 692

# Fusarium_sp-PT_008846-RA Length: 261

# Fusarium_sp-PT_008846-RA Number of predicted TMHs: 0

# Fusarium_sp-PT_008846-RA Exp number of AAs in TMHs: 0

# Fusarium_sp-PT_008846-RA Exp number, first 60 AAs: 0

# Fusarium_sp-PT_008846-RA Total prob of N-in: 0.01506

Fusarium_sp-PT_008846-RA TMHMM2.0 outside 1 261

# Fusarium_sp-PT_008847-RA Length: 508

# Fusarium_sp-PT_008847-RA Number of predicted TMHs: 0

# Fusarium_sp-PT_008847-RA Exp number of AAs in TMHs: 0.02414

# Fusarium_sp-PT_008847-RA Exp number, first 60 AAs: 0.01605

# Fusarium_sp-PT_008847-RA Total prob of N-in: 0.00656

Fusarium_sp-PT_008847-RA TMHMM2.0 outside 1 508

# Fusarium_sp-PT_008848-RA Length: 778

# Fusarium_sp-PT_008848-RA Number of predicted TMHs: 0

# Fusarium_sp-PT_008848-RA Exp number of AAs in TMHs: 0.00125

# Fusarium_sp-PT_008848-RA Exp number, first 60 AAs: 0

# Fusarium_sp-PT_008848-RA Total prob of N-in: 0.00006

Fusarium_sp-PT_008848-RA TMHMM2.0 outside 1 778

# Fusarium_sp-PT_008849-RA Length: 1220

# Fusarium_sp-PT_008849-RA Number of predicted TMHs: 0

# Fusarium_sp-PT_008849-RA Exp number of AAs in TMHs: 7.72203999999999

# Fusarium_sp-PT_008849-RA Exp number, first 60 AAs: 5.01077

# Fusarium_sp-PT_008849-RA Total prob of N-in: 0.26008

Fusarium_sp-PT_008849-RA TMHMM2.0 outside 1 1220

# Fusarium_sp-PT_008852-RA Length: 528

# Fusarium_sp-PT_008852-RA Number of predicted TMHs: 0

# Fusarium_sp-PT_008852-RA Exp number of AAs in TMHs: 0.02296

# Fusarium_sp-PT_008852-RA Exp number, first 60 AAs: 0.02088

# Fusarium_sp-PT_008852-RA Total prob of N-in: 0.00280

Fusarium_sp-PT_008852-RA TMHMM2.0 outside 1 528

# Fusarium_sp-PT_008853-RA Length: 709

# Fusarium_sp-PT_008853-RA Number of predicted TMHs: 0

# Fusarium_sp-PT_008853-RA Exp number of AAs in TMHs: 0.1708

# Fusarium_sp-PT_008853-RA Exp number, first 60 AAs: 0

# Fusarium_sp-PT_008853-RA Total prob of N-in: 0.00098

Fusarium_sp-PT_008853-RA TMHMM2.0 outside 1 709

# Fusarium_sp-PT_008854-RA Length: 369

# Fusarium_sp-PT_008854-RA Number of predicted TMHs: 0

# Fusarium_sp-PT_008854-RA Exp number of AAs in TMHs: 0.00098

# Fusarium_sp-PT_008854-RA Exp number, first 60 AAs: 0.0008

# Fusarium_sp-PT_008854-RA Total prob of N-in: 0.01061

Fusarium_sp-PT_008854-RA TMHMM2.0 outside 1 369

# Fusarium_sp-PT_008829-RA Length: 996

# Fusarium_sp-PT_008829-RA Number of predicted TMHs: 0

# Fusarium_sp-PT_008829-RA Exp number of AAs in TMHs: 4.97258

# Fusarium_sp-PT_008829-RA Exp number, first 60 AAs: 0.00276

# Fusarium_sp-PT_008829-RA Total prob of N-in: 0.22221

Fusarium_sp-PT_008829-RA TMHMM2.0 outside 1 996

# Fusarium_sp-PT_008830-RA Length: 732

# Fusarium_sp-PT_008830-RA Number of predicted TMHs: 8

# Fusarium_sp-PT_008830-RA Exp number of AAs in TMHs: 196.87557

# Fusarium_sp-PT_008830-RA Exp number, first 60 AAs: 22.60362

# Fusarium_sp-PT_008830-RA Total prob of N-in: 0.06763

# Fusarium_sp-PT_008830-RA POSSIBLE N-term signal sequence

Fusarium_sp-PT_008830-RA TMHMM2.0 outside 1 4

Fusarium_sp-PT_008830-RA TMHMM2.0 TMhelix 5 27

Fusarium_sp-PT_008830-RA TMHMM2.0 inside 28 366

Fusarium_sp-PT_008830-RA TMHMM2.0 TMhelix 367 386

Fusarium_sp-PT_008830-RA TMHMM2.0 outside 387 400

Fusarium_sp-PT_008830-RA TMHMM2.0 TMhelix 401 418

Fusarium_sp-PT_008830-RA TMHMM2.0 inside 419 429

Fusarium_sp-PT_008830-RA TMHMM2.0 TMhelix 430 452

Fusarium_sp-PT_008830-RA TMHMM2.0 outside 453 502

Fusarium_sp-PT_008830-RA TMHMM2.0 TMhelix 503 525

Fusarium_sp-PT_008830-RA TMHMM2.0 inside 526 531

Fusarium_sp-PT_008830-RA TMHMM2.0 TMhelix 532 554

Fusarium_sp-PT_008830-RA TMHMM2.0 outside 555 557

Fusarium_sp-PT_008830-RA TMHMM2.0 TMhelix 558 580

Fusarium_sp-PT_008830-RA TMHMM2.0 inside 581 591

Fusarium_sp-PT_008830-RA TMHMM2.0 TMhelix 592 611

Fusarium_sp-PT_008830-RA TMHMM2.0 outside 612 732

# Fusarium_sp-PT_008834-RA Length: 907

# Fusarium_sp-PT_008834-RA Number of predicted TMHs: 0

# Fusarium_sp-PT_008834-RA Exp number of AAs in TMHs: 8.95481999999998

# Fusarium_sp-PT_008834-RA Exp number, first 60 AAs: 8.94936

# Fusarium_sp-PT_008834-RA Total prob of N-in: 0.47921

Fusarium_sp-PT_008834-RA TMHMM2.0 outside 1 907

# Fusarium_sp-PT_008836-RA Length: 189

# Fusarium_sp-PT_008836-RA Number of predicted TMHs: 4

# Fusarium_sp-PT_008836-RA Exp number of AAs in TMHs: 81.90238

# Fusarium_sp-PT_008836-RA Exp number, first 60 AAs: 26.59446

# Fusarium_sp-PT_008836-RA Total prob of N-in: 0.96337

# Fusarium_sp-PT_008836-RA POSSIBLE N-term signal sequence

Fusarium_sp-PT_008836-RA TMHMM2.0 inside 1 12

Fusarium_sp-PT_008836-RA TMHMM2.0 TMhelix 13 32

Fusarium_sp-PT_008836-RA TMHMM2.0 outside 33 51

Fusarium_sp-PT_008836-RA TMHMM2.0 TMhelix 52 74

Fusarium_sp-PT_008836-RA TMHMM2.0 inside 75 94

Fusarium_sp-PT_008836-RA TMHMM2.0 TMhelix 95 117

Fusarium_sp-PT_008836-RA TMHMM2.0 outside 118 148

Fusarium_sp-PT_008836-RA TMHMM2.0 TMhelix 149 168

Fusarium_sp-PT_008836-RA TMHMM2.0 inside 169 189

# Fusarium_sp-PT_008843-RA Length: 227

# Fusarium_sp-PT_008843-RA Number of predicted TMHs: 0

# Fusarium_sp-PT_008843-RA Exp number of AAs in TMHs: 0.0950799999999999

# Fusarium_sp-PT_008843-RA Exp number, first 60 AAs: 0.08211

# Fusarium_sp-PT_008843-RA Total prob of N-in: 0.00495

Fusarium_sp-PT_008843-RA TMHMM2.0 outside 1 227

# Fusarium_sp-PT_008845-RA Length: 331

# Fusarium_sp-PT_008845-RA Number of predicted TMHs: 0

# Fusarium_sp-PT_008845-RA Exp number of AAs in TMHs: 0.05846

# Fusarium_sp-PT_008845-RA Exp number, first 60 AAs: 0.0004

# Fusarium_sp-PT_008845-RA Total prob of N-in: 0.00227

Fusarium_sp-PT_008845-RA TMHMM2.0 outside 1 331

# Fusarium_sp-PT_008850-RA Length: 221

# Fusarium_sp-PT_008850-RA Number of predicted TMHs: 0

# Fusarium_sp-PT_008850-RA Exp number of AAs in TMHs: 0.25992

# Fusarium_sp-PT_008850-RA Exp number, first 60 AAs: 0.2498

# Fusarium_sp-PT_008850-RA Total prob of N-in: 0.05907

Fusarium_sp-PT_008850-RA TMHMM2.0 outside 1 221

# Fusarium_sp-PT_008851-RA Length: 747

# Fusarium_sp-PT_008851-RA Number of predicted TMHs: 0

# Fusarium_sp-PT_008851-RA Exp number of AAs in TMHs: 0.000690000000000001

# Fusarium_sp-PT_008851-RA Exp number, first 60 AAs: 0

# Fusarium_sp-PT_008851-RA Total prob of N-in: 0.00006

Fusarium_sp-PT_008851-RA TMHMM2.0 outside 1 747

# Fusarium_sp-PT_008839-RA Length: 57

# Fusarium_sp-PT_008839-RA Number of predicted TMHs: 0

# Fusarium_sp-PT_008839-RA Exp number of AAs in TMHs: 0

# Fusarium_sp-PT_008839-RA Exp number, first 60 AAs: 0

# Fusarium_sp-PT_008839-RA Total prob of N-in: 0.25531

Fusarium_sp-PT_008839-RA TMHMM2.0 outside 1 57

# Fusarium_sp-PT_008855-RA Length: 479

# Fusarium_sp-PT_008855-RA Number of predicted TMHs: 5

# Fusarium_sp-PT_008855-RA Exp number of AAs in TMHs: 96.9808200000002

# Fusarium_sp-PT_008855-RA Exp number, first 60 AAs: 0

# Fusarium_sp-PT_008855-RA Total prob of N-in: 0.29017

Fusarium_sp-PT_008855-RA TMHMM2.0 outside 1 102

Fusarium_sp-PT_008855-RA TMHMM2.0 TMhelix 103 122

Fusarium_sp-PT_008855-RA TMHMM2.0 inside 123 133

Fusarium_sp-PT_008855-RA TMHMM2.0 TMhelix 134 156

Fusarium_sp-PT_008855-RA TMHMM2.0 outside 157 170

Fusarium_sp-PT_008855-RA TMHMM2.0 TMhelix 171 188

Fusarium_sp-PT_008855-RA TMHMM2.0 inside 189 299

Fusarium_sp-PT_008855-RA TMHMM2.0 TMhelix 300 319

Fusarium_sp-PT_008855-RA TMHMM2.0 outside 320 328

Fusarium_sp-PT_008855-RA TMHMM2.0 TMhelix 329 348

Fusarium_sp-PT_008855-RA TMHMM2.0 inside 349 479

# Fusarium_sp-PT_008857-RA Length: 1641

# Fusarium_sp-PT_008857-RA Number of predicted TMHs: 0

# Fusarium_sp-PT_008857-RA Exp number of AAs in TMHs: 2.99916000000001

# Fusarium_sp-PT_008857-RA Exp number, first 60 AAs: 2.94757

# Fusarium_sp-PT_008857-RA Total prob of N-in: 0.12455

Fusarium_sp-PT_008857-RA TMHMM2.0 outside 1 1641

# Fusarium_sp-PT_008858-RA Length: 947

# Fusarium_sp-PT_008858-RA Number of predicted TMHs: 0

# Fusarium_sp-PT_008858-RA Exp number of AAs in TMHs: 0.01006

# Fusarium_sp-PT_008858-RA Exp number, first 60 AAs: 0.00059

# Fusarium_sp-PT_008858-RA Total prob of N-in: 0.00013

Fusarium_sp-PT_008858-RA TMHMM2.0 outside 1 947

# Fusarium_sp-PT_008859-RA Length: 325

# Fusarium_sp-PT_008859-RA Number of predicted TMHs: 0

# Fusarium_sp-PT_008859-RA Exp number of AAs in TMHs: 14.2456

# Fusarium_sp-PT_008859-RA Exp number, first 60 AAs: 14.24236

# Fusarium_sp-PT_008859-RA Total prob of N-in: 0.65795

# Fusarium_sp-PT_008859-RA POSSIBLE N-term signal sequence

Fusarium_sp-PT_008859-RA TMHMM2.0 outside 1 325

# Fusarium_sp-PT_008860-RA Length: 765

# Fusarium_sp-PT_008860-RA Number of predicted TMHs: 0

# Fusarium_sp-PT_008860-RA Exp number of AAs in TMHs: 5.18615

# Fusarium_sp-PT_008860-RA Exp number, first 60 AAs: 0

# Fusarium_sp-PT_008860-RA Total prob of N-in: 0.00231

Fusarium_sp-PT_008860-RA TMHMM2.0 outside 1 765

# Fusarium_sp-PT_008861-RA Length: 132

# Fusarium_sp-PT_008861-RA Number of predicted TMHs: 0

# Fusarium_sp-PT_008861-RA Exp number of AAs in TMHs: 0.70778

# Fusarium_sp-PT_008861-RA Exp number, first 60 AAs: 0.70778

# Fusarium_sp-PT_008861-RA Total prob of N-in: 0.17827

Fusarium_sp-PT_008861-RA TMHMM2.0 outside 1 132

# Fusarium_sp-PT_008871-RA Length: 919

# Fusarium_sp-PT_008871-RA Number of predicted TMHs: 0

# Fusarium_sp-PT_008871-RA Exp number of AAs in TMHs: 0.05426

# Fusarium_sp-PT_008871-RA Exp number, first 60 AAs: 0

# Fusarium_sp-PT_008871-RA Total prob of N-in: 0.00001

Fusarium_sp-PT_008871-RA TMHMM2.0 outside 1 919

# Fusarium_sp-PT_008873-RA Length: 401

# Fusarium_sp-PT_008873-RA Number of predicted TMHs: 0

# Fusarium_sp-PT_008873-RA Exp number of AAs in TMHs: 0.00393

# Fusarium_sp-PT_008873-RA Exp number, first 60 AAs: 0

# Fusarium_sp-PT_008873-RA Total prob of N-in: 0.00594

Fusarium_sp-PT_008873-RA TMHMM2.0 outside 1 401

# Fusarium_sp-PT_008879-RA Length: 188

# Fusarium_sp-PT_008879-RA Number of predicted TMHs: 1

# Fusarium_sp-PT_008879-RA Exp number of AAs in TMHs: 22.60504

# Fusarium_sp-PT_008879-RA Exp number, first 60 AAs: 0.0005

# Fusarium_sp-PT_008879-RA Total prob of N-in: 0.17258

Fusarium_sp-PT_008879-RA TMHMM2.0 outside 1 131

Fusarium_sp-PT_008879-RA TMHMM2.0 TMhelix 132 154

Fusarium_sp-PT_008879-RA TMHMM2.0 inside 155 188

# Fusarium_sp-PT_008880-RA Length: 423

# Fusarium_sp-PT_008880-RA Number of predicted TMHs: 0

# Fusarium_sp-PT_008880-RA Exp number of AAs in TMHs: 0.00111

# Fusarium_sp-PT_008880-RA Exp number, first 60 AAs: 0.00105

# Fusarium_sp-PT_008880-RA Total prob of N-in: 0.00439

Fusarium_sp-PT_008880-RA TMHMM2.0 outside 1 423

# Fusarium_sp-PT_008881-RA Length: 654

# Fusarium_sp-PT_008881-RA Number of predicted TMHs: 14

# Fusarium_sp-PT_008881-RA Exp number of AAs in TMHs: 304.70034

# Fusarium_sp-PT_008881-RA Exp number, first 60 AAs: 0.00019

# Fusarium_sp-PT_008881-RA Total prob of N-in: 0.91305

Fusarium_sp-PT_008881-RA TMHMM2.0 inside 1 154

Fusarium_sp-PT_008881-RA TMHMM2.0 TMhelix 155 177

Fusarium_sp-PT_008881-RA TMHMM2.0 outside 178 191

Fusarium_sp-PT_008881-RA TMHMM2.0 TMhelix 192 214

Fusarium_sp-PT_008881-RA TMHMM2.0 inside 215 220

Fusarium_sp-PT_008881-RA TMHMM2.0 TMhelix 221 240

Fusarium_sp-PT_008881-RA TMHMM2.0 outside 241 249

Fusarium_sp-PT_008881-RA TMHMM2.0 TMhelix 250 272

Fusarium_sp-PT_008881-RA TMHMM2.0 inside 273 278

Fusarium_sp-PT_008881-RA TMHMM2.0 TMhelix 279 301

Fusarium_sp-PT_008881-RA TMHMM2.0 outside 302 310

Fusarium_sp-PT_008881-RA TMHMM2.0 TMhelix 311 330

Fusarium_sp-PT_008881-RA TMHMM2.0 inside 331 350

Fusarium_sp-PT_008881-RA TMHMM2.0 TMhelix 351 373

Fusarium_sp-PT_008881-RA TMHMM2.0 outside 374 376

Fusarium_sp-PT_008881-RA TMHMM2.0 TMhelix 377 399

Fusarium_sp-PT_008881-RA TMHMM2.0 inside 400 419

Fusarium_sp-PT_008881-RA TMHMM2.0 TMhelix 420 442

Fusarium_sp-PT_008881-RA TMHMM2.0 outside 443 461

Fusarium_sp-PT_008881-RA TMHMM2.0 TMhelix 462 484

Fusarium_sp-PT_008881-RA TMHMM2.0 inside 485 485

Fusarium_sp-PT_008881-RA TMHMM2.0 TMhelix 486 503

Fusarium_sp-PT_008881-RA TMHMM2.0 outside 504 512

Fusarium_sp-PT_008881-RA TMHMM2.0 TMhelix 513 535

Fusarium_sp-PT_008881-RA TMHMM2.0 inside 536 547

Fusarium_sp-PT_008881-RA TMHMM2.0 TMhelix 548 570

Fusarium_sp-PT_008881-RA TMHMM2.0 outside 571 616

Fusarium_sp-PT_008881-RA TMHMM2.0 TMhelix 617 639

Fusarium_sp-PT_008881-RA TMHMM2.0 inside 640 654

# Fusarium_sp-PT_008862-RA Length: 318

# Fusarium_sp-PT_008862-RA Number of predicted TMHs: 0

# Fusarium_sp-PT_008862-RA Exp number of AAs in TMHs: 0.42023

# Fusarium_sp-PT_008862-RA Exp number, first 60 AAs: 0.28697

# Fusarium_sp-PT_008862-RA Total prob of N-in: 0.01881

Fusarium_sp-PT_008862-RA TMHMM2.0 outside 1 318

# Fusarium_sp-PT_008863-RA Length: 562

# Fusarium_sp-PT_008863-RA Number of predicted TMHs: 0

# Fusarium_sp-PT_008863-RA Exp number of AAs in TMHs: 9.79307999999997

# Fusarium_sp-PT_008863-RA Exp number, first 60 AAs: 9.6468

# Fusarium_sp-PT_008863-RA Total prob of N-in: 0.46351

Fusarium_sp-PT_008863-RA TMHMM2.0 outside 1 562

# Fusarium_sp-PT_008864-RA Length: 190

# Fusarium_sp-PT_008864-RA Number of predicted TMHs: 0

# Fusarium_sp-PT_008864-RA Exp number of AAs in TMHs: 0.00154

# Fusarium_sp-PT_008864-RA Exp number, first 60 AAs: 0.00154

# Fusarium_sp-PT_008864-RA Total prob of N-in: 0.15692

Fusarium_sp-PT_008864-RA TMHMM2.0 outside 1 190

# Fusarium_sp-PT_008866-RA Length: 209

# Fusarium_sp-PT_008866-RA Number of predicted TMHs: 0

# Fusarium_sp-PT_008866-RA Exp number of AAs in TMHs: 0.00912

# Fusarium_sp-PT_008866-RA Exp number, first 60 AAs: 0

# Fusarium_sp-PT_008866-RA Total prob of N-in: 0.09977

Fusarium_sp-PT_008866-RA TMHMM2.0 outside 1 209

# Fusarium_sp-PT_008867-RA Length: 275

# Fusarium_sp-PT_008867-RA Number of predicted TMHs: 0

# Fusarium_sp-PT_008867-RA Exp number of AAs in TMHs: 1.01532

# Fusarium_sp-PT_008867-RA Exp number, first 60 AAs: 1.00866

# Fusarium_sp-PT_008867-RA Total prob of N-in: 0.07442

Fusarium_sp-PT_008867-RA TMHMM2.0 outside 1 275

# Fusarium_sp-PT_008868-RA Length: 183

# Fusarium_sp-PT_008868-RA Number of predicted TMHs: 0

# Fusarium_sp-PT_008868-RA Exp number of AAs in TMHs: 0.02589

# Fusarium_sp-PT_008868-RA Exp number, first 60 AAs: 0

# Fusarium_sp-PT_008868-RA Total prob of N-in: 0.08425

Fusarium_sp-PT_008868-RA TMHMM2.0 outside 1 183

# Fusarium_sp-PT_008870-RA Length: 246

# Fusarium_sp-PT_008870-RA Number of predicted TMHs: 0

# Fusarium_sp-PT_008870-RA Exp number of AAs in TMHs: 0.19334

# Fusarium_sp-PT_008870-RA Exp number, first 60 AAs: 0.0013

# Fusarium_sp-PT_008870-RA Total prob of N-in: 0.05185

Fusarium_sp-PT_008870-RA TMHMM2.0 outside 1 246

# Fusarium_sp-PT_008872-RA Length: 425

# Fusarium_sp-PT_008872-RA Number of predicted TMHs: 6

# Fusarium_sp-PT_008872-RA Exp number of AAs in TMHs: 119.79843

# Fusarium_sp-PT_008872-RA Exp number, first 60 AAs: 41.67089

# Fusarium_sp-PT_008872-RA Total prob of N-in: 0.44402

# Fusarium_sp-PT_008872-RA POSSIBLE N-term signal sequence

Fusarium_sp-PT_008872-RA TMHMM2.0 outside 1 4

Fusarium_sp-PT_008872-RA TMHMM2.0 TMhelix 5 27

Fusarium_sp-PT_008872-RA TMHMM2.0 inside 28 39

Fusarium_sp-PT_008872-RA TMHMM2.0 TMhelix 40 59

Fusarium_sp-PT_008872-RA TMHMM2.0 outside 60 68

Fusarium_sp-PT_008872-RA TMHMM2.0 TMhelix 69 86

Fusarium_sp-PT_008872-RA TMHMM2.0 inside 87 105

Fusarium_sp-PT_008872-RA TMHMM2.0 TMhelix 106 123

Fusarium_sp-PT_008872-RA TMHMM2.0 outside 124 137

Fusarium_sp-PT_008872-RA TMHMM2.0 TMhelix 138 157

Fusarium_sp-PT_008872-RA TMHMM2.0 inside 158 161

Fusarium_sp-PT_008872-RA TMHMM2.0 TMhelix 162 184

Fusarium_sp-PT_008872-RA TMHMM2.0 outside 185 425

# Fusarium_sp-PT_008874-RA Length: 420

# Fusarium_sp-PT_008874-RA Number of predicted TMHs: 0

# Fusarium_sp-PT_008874-RA Exp number of AAs in TMHs: 6.63561

# Fusarium_sp-PT_008874-RA Exp number, first 60 AAs: 0.00023

# Fusarium_sp-PT_008874-RA Total prob of N-in: 0.10461

Fusarium_sp-PT_008874-RA TMHMM2.0 outside 1 420

# Fusarium_sp-PT_008875-RA Length: 394

# Fusarium_sp-PT_008875-RA Number of predicted TMHs: 6

# Fusarium_sp-PT_008875-RA Exp number of AAs in TMHs: 146.72668

# Fusarium_sp-PT_008875-RA Exp number, first 60 AAs: 34.18448

# Fusarium_sp-PT_008875-RA Total prob of N-in: 0.00368

# Fusarium_sp-PT_008875-RA POSSIBLE N-term signal sequence

Fusarium_sp-PT_008875-RA TMHMM2.0 outside 1 14

Fusarium_sp-PT_008875-RA TMHMM2.0 TMhelix 15 35

Fusarium_sp-PT_008875-RA TMHMM2.0 inside 36 47

Fusarium_sp-PT_008875-RA TMHMM2.0 TMhelix 48 67

Fusarium_sp-PT_008875-RA TMHMM2.0 outside 68 94

Fusarium_sp-PT_008875-RA TMHMM2.0 TMhelix 95 117

Fusarium_sp-PT_008875-RA TMHMM2.0 inside 118 137

Fusarium_sp-PT_008875-RA TMHMM2.0 TMhelix 138 160

Fusarium_sp-PT_008875-RA TMHMM2.0 outside 161 174

Fusarium_sp-PT_008875-RA TMHMM2.0 TMhelix 175 196

Fusarium_sp-PT_008875-RA TMHMM2.0 inside 197 208

Fusarium_sp-PT_008875-RA TMHMM2.0 TMhelix 209 231

Fusarium_sp-PT_008875-RA TMHMM2.0 outside 232 394

# Fusarium_sp-PT_008876-RA Length: 546

# Fusarium_sp-PT_008876-RA Number of predicted TMHs: 0

# Fusarium_sp-PT_008876-RA Exp number of AAs in TMHs: 0.00344

# Fusarium_sp-PT_008876-RA Exp number, first 60 AAs: 0

# Fusarium_sp-PT_008876-RA Total prob of N-in: 0.00116

Fusarium_sp-PT_008876-RA TMHMM2.0 outside 1 546

# Fusarium_sp-PT_008877-RA Length: 262

# Fusarium_sp-PT_008877-RA Number of predicted TMHs: 0

# Fusarium_sp-PT_008877-RA Exp number of AAs in TMHs: 0.25688

# Fusarium_sp-PT_008877-RA Exp number, first 60 AAs: 0.00095

# Fusarium_sp-PT_008877-RA Total prob of N-in: 0.04225

Fusarium_sp-PT_008877-RA TMHMM2.0 outside 1 262

# Fusarium_sp-PT_008878-RA Length: 1163

# Fusarium_sp-PT_008878-RA Number of predicted TMHs: 0

# Fusarium_sp-PT_008878-RA Exp number of AAs in TMHs: 0

# Fusarium_sp-PT_008878-RA Exp number, first 60 AAs: 0

# Fusarium_sp-PT_008878-RA Total prob of N-in: 0.00000

Fusarium_sp-PT_008878-RA TMHMM2.0 outside 1 1163

# Fusarium_sp-PT_008882-RA Length: 1154

# Fusarium_sp-PT_008882-RA Number of predicted TMHs: 0

# Fusarium_sp-PT_008882-RA Exp number of AAs in TMHs: 0.19752

# Fusarium_sp-PT_008882-RA Exp number, first 60 AAs: 0.01136

# Fusarium_sp-PT_008882-RA Total prob of N-in: 0.00771

Fusarium_sp-PT_008882-RA TMHMM2.0 outside 1 1154

# Fusarium_sp-PT_008865-RA Length: 505

# Fusarium_sp-PT_008865-RA Number of predicted TMHs: 0

# Fusarium_sp-PT_008865-RA Exp number of AAs in TMHs: 0.02164

# Fusarium_sp-PT_008865-RA Exp number, first 60 AAs: 0.0206

# Fusarium_sp-PT_008865-RA Total prob of N-in: 0.00276

Fusarium_sp-PT_008865-RA TMHMM2.0 outside 1 505

# Fusarium_sp-PT_008884-RA Length: 922

# Fusarium_sp-PT_008884-RA Number of predicted TMHs: 6

# Fusarium_sp-PT_008884-RA Exp number of AAs in TMHs: 116.11608

# Fusarium_sp-PT_008884-RA Exp number, first 60 AAs: 0

# Fusarium_sp-PT_008884-RA Total prob of N-in: 0.13839

Fusarium_sp-PT_008884-RA TMHMM2.0 outside 1 675

Fusarium_sp-PT_008884-RA TMHMM2.0 TMhelix 676 693

Fusarium_sp-PT_008884-RA TMHMM2.0 inside 694 704

Fusarium_sp-PT_008884-RA TMHMM2.0 TMhelix 705 724

Fusarium_sp-PT_008884-RA TMHMM2.0 outside 725 733

Fusarium_sp-PT_008884-RA TMHMM2.0 TMhelix 734 754

Fusarium_sp-PT_008884-RA TMHMM2.0 inside 755 792

Fusarium_sp-PT_008884-RA TMHMM2.0 TMhelix 793 815

Fusarium_sp-PT_008884-RA TMHMM2.0 outside 816 818

Fusarium_sp-PT_008884-RA TMHMM2.0 TMhelix 819 838

Fusarium_sp-PT_008884-RA TMHMM2.0 inside 839 844

Fusarium_sp-PT_008884-RA TMHMM2.0 TMhelix 845 867

Fusarium_sp-PT_008884-RA TMHMM2.0 outside 868 922

# Fusarium_sp-PT_008885-RA Length: 563

# Fusarium_sp-PT_008885-RA Number of predicted TMHs: 0

# Fusarium_sp-PT_008885-RA Exp number of AAs in TMHs: 0.0004

# Fusarium_sp-PT_008885-RA Exp number, first 60 AAs: 0

# Fusarium_sp-PT_008885-RA Total prob of N-in: 0.00351

Fusarium_sp-PT_008885-RA TMHMM2.0 outside 1 563

# Fusarium_sp-PT_008886-RA Length: 605

# Fusarium_sp-PT_008886-RA Number of predicted TMHs: 0

# Fusarium_sp-PT_008886-RA Exp number of AAs in TMHs: 0.06383

# Fusarium_sp-PT_008886-RA Exp number, first 60 AAs: 0.00209

# Fusarium_sp-PT_008886-RA Total prob of N-in: 0.00052

Fusarium_sp-PT_008886-RA TMHMM2.0 outside 1 605

# Fusarium_sp-PT_008887-RA Length: 498

# Fusarium_sp-PT_008887-RA Number of predicted TMHs: 0

# Fusarium_sp-PT_008887-RA Exp number of AAs in TMHs: 0.01687

# Fusarium_sp-PT_008887-RA Exp number, first 60 AAs: 0.00386

# Fusarium_sp-PT_008887-RA Total prob of N-in: 0.00450

Fusarium_sp-PT_008887-RA TMHMM2.0 outside 1 498

# Fusarium_sp-PT_008898-RA Length: 538

# Fusarium_sp-PT_008898-RA Number of predicted TMHs: 0

# Fusarium_sp-PT_008898-RA Exp number of AAs in TMHs: 0.0085

# Fusarium_sp-PT_008898-RA Exp number, first 60 AAs: 0.0085

# Fusarium_sp-PT_008898-RA Total prob of N-in: 0.00189

Fusarium_sp-PT_008898-RA TMHMM2.0 outside 1 538

# Fusarium_sp-PT_008899-RA Length: 688

# Fusarium_sp-PT_008899-RA Number of predicted TMHs: 0

# Fusarium_sp-PT_008899-RA Exp number of AAs in TMHs: 0.00544

# Fusarium_sp-PT_008899-RA Exp number, first 60 AAs: 0.00018

# Fusarium_sp-PT_008899-RA Total prob of N-in: 0.00060

Fusarium_sp-PT_008899-RA TMHMM2.0 outside 1 688

# Fusarium_sp-PT_008900-RA Length: 378

# Fusarium_sp-PT_008900-RA Number of predicted TMHs: 0

# Fusarium_sp-PT_008900-RA Exp number of AAs in TMHs: 1.45949

# Fusarium_sp-PT_008900-RA Exp number, first 60 AAs: 0.0002

# Fusarium_sp-PT_008900-RA Total prob of N-in: 0.02139

Fusarium_sp-PT_008900-RA TMHMM2.0 outside 1 378

# Fusarium_sp-PT_008903-RA Length: 490

# Fusarium_sp-PT_008903-RA Number of predicted TMHs: 8

# Fusarium_sp-PT_008903-RA Exp number of AAs in TMHs: 194.78215

# Fusarium_sp-PT_008903-RA Exp number, first 60 AAs: 0.04001

# Fusarium_sp-PT_008903-RA Total prob of N-in: 0.78963

Fusarium_sp-PT_008903-RA TMHMM2.0 inside 1 126

Fusarium_sp-PT_008903-RA TMHMM2.0 TMhelix 127 149

Fusarium_sp-PT_008903-RA TMHMM2.0 outside 150 153

Fusarium_sp-PT_008903-RA TMHMM2.0 TMhelix 154 176

Fusarium_sp-PT_008903-RA TMHMM2.0 inside 177 188

Fusarium_sp-PT_008903-RA TMHMM2.0 TMhelix 189 211

Fusarium_sp-PT_008903-RA TMHMM2.0 outside 212 225

Fusarium_sp-PT_008903-RA TMHMM2.0 TMhelix 226 245

Fusarium_sp-PT_008903-RA TMHMM2.0 inside 246 319

Fusarium_sp-PT_008903-RA TMHMM2.0 TMhelix 320 342

Fusarium_sp-PT_008903-RA TMHMM2.0 outside 343 382

Fusarium_sp-PT_008903-RA TMHMM2.0 TMhelix 383 405

Fusarium_sp-PT_008903-RA TMHMM2.0 inside 406 417

Fusarium_sp-PT_008903-RA TMHMM2.0 TMhelix 418 437

Fusarium_sp-PT_008903-RA TMHMM2.0 outside 438 451

Fusarium_sp-PT_008903-RA TMHMM2.0 TMhelix 452 471

Fusarium_sp-PT_008903-RA TMHMM2.0 inside 472 490

# Fusarium_sp-PT_008905-RA Length: 255

# Fusarium_sp-PT_008905-RA Number of predicted TMHs: 0

# Fusarium_sp-PT_008905-RA Exp number of AAs in TMHs: 0.0053

# Fusarium_sp-PT_008905-RA Exp number, first 60 AAs: 0

# Fusarium_sp-PT_008905-RA Total prob of N-in: 0.02075

Fusarium_sp-PT_008905-RA TMHMM2.0 outside 1 255

# Fusarium_sp-PT_008907-RA Length: 213

# Fusarium_sp-PT_008907-RA Number of predicted TMHs: 5

# Fusarium_sp-PT_008907-RA Exp number of AAs in TMHs: 96.48477

# Fusarium_sp-PT_008907-RA Exp number, first 60 AAs: 35.87561

# Fusarium_sp-PT_008907-RA Total prob of N-in: 0.32206

# Fusarium_sp-PT_008907-RA POSSIBLE N-term signal sequence

Fusarium_sp-PT_008907-RA TMHMM2.0 outside 1 9

Fusarium_sp-PT_008907-RA TMHMM2.0 TMhelix 10 31

Fusarium_sp-PT_008907-RA TMHMM2.0 inside 32 43

Fusarium_sp-PT_008907-RA TMHMM2.0 TMhelix 44 61

Fusarium_sp-PT_008907-RA TMHMM2.0 outside 62 64

Fusarium_sp-PT_008907-RA TMHMM2.0 TMhelix 65 82

Fusarium_sp-PT_008907-RA TMHMM2.0 inside 83 94

Fusarium_sp-PT_008907-RA TMHMM2.0 TMhelix 95 112

Fusarium_sp-PT_008907-RA TMHMM2.0 outside 113 136

Fusarium_sp-PT_008907-RA TMHMM2.0 TMhelix 137 159

Fusarium_sp-PT_008907-RA TMHMM2.0 inside 160 213

# Fusarium_sp-PT_008914-RA Length: 390

# Fusarium_sp-PT_008914-RA Number of predicted TMHs: 0

# Fusarium_sp-PT_008914-RA Exp number of AAs in TMHs: 0.00141

# Fusarium_sp-PT_008914-RA Exp number, first 60 AAs: 0

# Fusarium_sp-PT_008914-RA Total prob of N-in: 0.00423

Fusarium_sp-PT_008914-RA TMHMM2.0 outside 1 390

# Fusarium_sp-PT_008916-RA Length: 472

# Fusarium_sp-PT_008916-RA Number of predicted TMHs: 8

# Fusarium_sp-PT_008916-RA Exp number of AAs in TMHs: 204.68203

# Fusarium_sp-PT_008916-RA Exp number, first 60 AAs: 20.46085

# Fusarium_sp-PT_008916-RA Total prob of N-in: 0.93232

# Fusarium_sp-PT_008916-RA POSSIBLE N-term signal sequence

Fusarium_sp-PT_008916-RA TMHMM2.0 inside 1 31

Fusarium_sp-PT_008916-RA TMHMM2.0 TMhelix 32 54

Fusarium_sp-PT_008916-RA TMHMM2.0 outside 55 109

Fusarium_sp-PT_008916-RA TMHMM2.0 TMhelix 110 132

Fusarium_sp-PT_008916-RA TMHMM2.0 inside 133 138

Fusarium_sp-PT_008916-RA TMHMM2.0 TMhelix 139 161

Fusarium_sp-PT_008916-RA TMHMM2.0 outside 162 170

Fusarium_sp-PT_008916-RA TMHMM2.0 TMhelix 171 193

Fusarium_sp-PT_008916-RA TMHMM2.0 inside 194 294

Fusarium_sp-PT_008916-RA TMHMM2.0 TMhelix 295 317

Fusarium_sp-PT_008916-RA TMHMM2.0 outside 318 331

Fusarium_sp-PT_008916-RA TMHMM2.0 TMhelix 332 354

Fusarium_sp-PT_008916-RA TMHMM2.0 inside 355 360

Fusarium_sp-PT_008916-RA TMHMM2.0 TMhelix 361 380

Fusarium_sp-PT_008916-RA TMHMM2.0 outside 381 394

Fusarium_sp-PT_008916-RA TMHMM2.0 TMhelix 395 417

Fusarium_sp-PT_008916-RA TMHMM2.0 inside 418 472

# Fusarium_sp-PT_008888-RA Length: 297

# Fusarium_sp-PT_008888-RA Number of predicted TMHs: 0

# Fusarium_sp-PT_008888-RA Exp number of AAs in TMHs: 0.22383

# Fusarium_sp-PT_008888-RA Exp number, first 60 AAs: 0.01155

# Fusarium_sp-PT_008888-RA Total prob of N-in: 0.13796

Fusarium_sp-PT_008888-RA TMHMM2.0 outside 1 297

# Fusarium_sp-PT_008889-RA Length: 1010

# Fusarium_sp-PT_008889-RA Number of predicted TMHs: 0

# Fusarium_sp-PT_008889-RA Exp number of AAs in TMHs: 0.00128

# Fusarium_sp-PT_008889-RA Exp number, first 60 AAs: 0

# Fusarium_sp-PT_008889-RA Total prob of N-in: 0.00003

Fusarium_sp-PT_008889-RA TMHMM2.0 outside 1 1010

# Fusarium_sp-PT_008890-RA Length: 323

# Fusarium_sp-PT_008890-RA Number of predicted TMHs: 0

# Fusarium_sp-PT_008890-RA Exp number of AAs in TMHs: 0.10711

# Fusarium_sp-PT_008890-RA Exp number, first 60 AAs: 0.02486

# Fusarium_sp-PT_008890-RA Total prob of N-in: 0.00661

Fusarium_sp-PT_008890-RA TMHMM2.0 outside 1 323

# Fusarium_sp-PT_008891-RA Length: 394

# Fusarium_sp-PT_008891-RA Number of predicted TMHs: 0

# Fusarium_sp-PT_008891-RA Exp number of AAs in TMHs: 0.07222

# Fusarium_sp-PT_008891-RA Exp number, first 60 AAs: 0.06694

# Fusarium_sp-PT_008891-RA Total prob of N-in: 0.02098

Fusarium_sp-PT_008891-RA TMHMM2.0 outside 1 394

# Fusarium_sp-PT_008892-RA Length: 381

# Fusarium_sp-PT_008892-RA Number of predicted TMHs: 0

# Fusarium_sp-PT_008892-RA Exp number of AAs in TMHs: 0.0494

# Fusarium_sp-PT_008892-RA Exp number, first 60 AAs: 0

# Fusarium_sp-PT_008892-RA Total prob of N-in: 0.01215

Fusarium_sp-PT_008892-RA TMHMM2.0 outside 1 381

# Fusarium_sp-PT_008893-RA Length: 735

# Fusarium_sp-PT_008893-RA Number of predicted TMHs: 0

# Fusarium_sp-PT_008893-RA Exp number of AAs in TMHs: 5.53029

# Fusarium_sp-PT_008893-RA Exp number, first 60 AAs: 0.00028

# Fusarium_sp-PT_008893-RA Total prob of N-in: 0.00085

Fusarium_sp-PT_008893-RA TMHMM2.0 outside 1 735

# Fusarium_sp-PT_008894-RA Length: 345

# Fusarium_sp-PT_008894-RA Number of predicted TMHs: 0

# Fusarium_sp-PT_008894-RA Exp number of AAs in TMHs: 0.22178

# Fusarium_sp-PT_008894-RA Exp number, first 60 AAs: 0

# Fusarium_sp-PT_008894-RA Total prob of N-in: 0.01033

Fusarium_sp-PT_008894-RA TMHMM2.0 outside 1 345

# Fusarium_sp-PT_008895-RA Length: 330

# Fusarium_sp-PT_008895-RA Number of predicted TMHs: 0

# Fusarium_sp-PT_008895-RA Exp number of AAs in TMHs: 0.01302

# Fusarium_sp-PT_008895-RA Exp number, first 60 AAs: 0.00157

# Fusarium_sp-PT_008895-RA Total prob of N-in: 0.01288

Fusarium_sp-PT_008895-RA TMHMM2.0 outside 1 330

# Fusarium_sp-PT_008896-RA Length: 414

# Fusarium_sp-PT_008896-RA Number of predicted TMHs: 0

# Fusarium_sp-PT_008896-RA Exp number of AAs in TMHs: 0.00851

# Fusarium_sp-PT_008896-RA Exp number, first 60 AAs: 0.00016

# Fusarium_sp-PT_008896-RA Total prob of N-in: 0.00173

Fusarium_sp-PT_008896-RA TMHMM2.0 outside 1 414

# Fusarium_sp-PT_008897-RA Length: 1830

# Fusarium_sp-PT_008897-RA Number of predicted TMHs: 0

# Fusarium_sp-PT_008897-RA Exp number of AAs in TMHs: 0.01743

# Fusarium_sp-PT_008897-RA Exp number, first 60 AAs: 2e-05

# Fusarium_sp-PT_008897-RA Total prob of N-in: 0.00026

Fusarium_sp-PT_008897-RA TMHMM2.0 outside 1 1830

# Fusarium_sp-PT_008901-RA Length: 607

# Fusarium_sp-PT_008901-RA Number of predicted TMHs: 0

# Fusarium_sp-PT_008901-RA Exp number of AAs in TMHs: 0.02136

# Fusarium_sp-PT_008901-RA Exp number, first 60 AAs: 9e-05

# Fusarium_sp-PT_008901-RA Total prob of N-in: 0.00108

Fusarium_sp-PT_008901-RA TMHMM2.0 outside 1 607

# Fusarium_sp-PT_008902-RA Length: 588

# Fusarium_sp-PT_008902-RA Number of predicted TMHs: 0

# Fusarium_sp-PT_008902-RA Exp number of AAs in TMHs: 0.0419999999999999

# Fusarium_sp-PT_008902-RA Exp number, first 60 AAs: 0

# Fusarium_sp-PT_008902-RA Total prob of N-in: 0.00333

Fusarium_sp-PT_008902-RA TMHMM2.0 outside 1 588

# Fusarium_sp-PT_008904-RA Length: 157

# Fusarium_sp-PT_008904-RA Number of predicted TMHs: 1

# Fusarium_sp-PT_008904-RA Exp number of AAs in TMHs: 22.85317

# Fusarium_sp-PT_008904-RA Exp number, first 60 AAs: 10.92709

# Fusarium_sp-PT_008904-RA Total prob of N-in: 0.00102

# Fusarium_sp-PT_008904-RA POSSIBLE N-term signal sequence

Fusarium_sp-PT_008904-RA TMHMM2.0 outside 1 49

Fusarium_sp-PT_008904-RA TMHMM2.0 TMhelix 50 72

Fusarium_sp-PT_008904-RA TMHMM2.0 inside 73 157

# Fusarium_sp-PT_008906-RA Length: 280

# Fusarium_sp-PT_008906-RA Number of predicted TMHs: 0

# Fusarium_sp-PT_008906-RA Exp number of AAs in TMHs: 0.00615999999999999

# Fusarium_sp-PT_008906-RA Exp number, first 60 AAs: 0.00031

# Fusarium_sp-PT_008906-RA Total prob of N-in: 0.06614

Fusarium_sp-PT_008906-RA TMHMM2.0 outside 1 280

# Fusarium_sp-PT_008909-RA Length: 142

# Fusarium_sp-PT_008909-RA Number of predicted TMHs: 0

# Fusarium_sp-PT_008909-RA Exp number of AAs in TMHs: 0

# Fusarium_sp-PT_008909-RA Exp number, first 60 AAs: 0

# Fusarium_sp-PT_008909-RA Total prob of N-in: 0.16696

Fusarium_sp-PT_008909-RA TMHMM2.0 outside 1 142

# Fusarium_sp-PT_008910-RA Length: 409

# Fusarium_sp-PT_008910-RA Number of predicted TMHs: 8

# Fusarium_sp-PT_008910-RA Exp number of AAs in TMHs: 174.41662

# Fusarium_sp-PT_008910-RA Exp number, first 60 AAs: 18.73189

# Fusarium_sp-PT_008910-RA Total prob of N-in: 0.98015

# Fusarium_sp-PT_008910-RA POSSIBLE N-term signal sequence

Fusarium_sp-PT_008910-RA TMHMM2.0 inside 1 6

Fusarium_sp-PT_008910-RA TMHMM2.0 TMhelix 7 24

Fusarium_sp-PT_008910-RA TMHMM2.0 outside 25 99

Fusarium_sp-PT_008910-RA TMHMM2.0 TMhelix 100 122

Fusarium_sp-PT_008910-RA TMHMM2.0 inside 123 133

Fusarium_sp-PT_008910-RA TMHMM2.0 TMhelix 134 156

Fusarium_sp-PT_008910-RA TMHMM2.0 outside 157 159

Fusarium_sp-PT_008910-RA TMHMM2.0 TMhelix 160 182

Fusarium_sp-PT_008910-RA TMHMM2.0 inside 183 202

Fusarium_sp-PT_008910-RA TMHMM2.0 TMhelix 203 225

Fusarium_sp-PT_008910-RA TMHMM2.0 outside 226 244

Fusarium_sp-PT_008910-RA TMHMM2.0 TMhelix 245 267

Fusarium_sp-PT_008910-RA TMHMM2.0 inside 268 287

Fusarium_sp-PT_008910-RA TMHMM2.0 TMhelix 288 307

Fusarium_sp-PT_008910-RA TMHMM2.0 outside 308 326

Fusarium_sp-PT_008910-RA TMHMM2.0 TMhelix 327 349

Fusarium_sp-PT_008910-RA TMHMM2.0 inside 350 409

# Fusarium_sp-PT_008911-RA Length: 961

# Fusarium_sp-PT_008911-RA Number of predicted TMHs: 0

# Fusarium_sp-PT_008911-RA Exp number of AAs in TMHs: 0.846919999999998

# Fusarium_sp-PT_008911-RA Exp number, first 60 AAs: 0.56613

# Fusarium_sp-PT_008911-RA Total prob of N-in: 0.03151

Fusarium_sp-PT_008911-RA TMHMM2.0 outside 1 961

# Fusarium_sp-PT_008912-RA Length: 478

# Fusarium_sp-PT_008912-RA Number of predicted TMHs: 5

# Fusarium_sp-PT_008912-RA Exp number of AAs in TMHs: 107.58496

# Fusarium_sp-PT_008912-RA Exp number, first 60 AAs: 0

# Fusarium_sp-PT_008912-RA Total prob of N-in: 0.99997

Fusarium_sp-PT_008912-RA TMHMM2.0 inside 1 183

Fusarium_sp-PT_008912-RA TMHMM2.0 TMhelix 184 206

Fusarium_sp-PT_008912-RA TMHMM2.0 outside 207 215

Fusarium_sp-PT_008912-RA TMHMM2.0 TMhelix 216 238

Fusarium_sp-PT_008912-RA TMHMM2.0 inside 239 257

Fusarium_sp-PT_008912-RA TMHMM2.0 TMhelix 258 275

Fusarium_sp-PT_008912-RA TMHMM2.0 outside 276 289

Fusarium_sp-PT_008912-RA TMHMM2.0 TMhelix 290 312

Fusarium_sp-PT_008912-RA TMHMM2.0 inside 313 331

Fusarium_sp-PT_008912-RA TMHMM2.0 TMhelix 332 351

Fusarium_sp-PT_008912-RA TMHMM2.0 outside 352 478

# Fusarium_sp-PT_008913-RA Length: 852

# Fusarium_sp-PT_008913-RA Number of predicted TMHs: 0

# Fusarium_sp-PT_008913-RA Exp number of AAs in TMHs: 0.00152

# Fusarium_sp-PT_008913-RA Exp number, first 60 AAs: 0

# Fusarium_sp-PT_008913-RA Total prob of N-in: 0.00008

Fusarium_sp-PT_008913-RA TMHMM2.0 outside 1 852

# Fusarium_sp-PT_008908-RA Length: 81

# Fusarium_sp-PT_008908-RA Number of predicted TMHs: 0

# Fusarium_sp-PT_008908-RA Exp number of AAs in TMHs: 0

# Fusarium_sp-PT_008908-RA Exp number, first 60 AAs: 0

# Fusarium_sp-PT_008908-RA Total prob of N-in: 0.12742

Fusarium_sp-PT_008908-RA TMHMM2.0 outside 1 81

# Fusarium_sp-PT_008919-RA Length: 921

# Fusarium_sp-PT_008919-RA Number of predicted TMHs: 0

# Fusarium_sp-PT_008919-RA Exp number of AAs in TMHs: 0.52209

# Fusarium_sp-PT_008919-RA Exp number, first 60 AAs: 0

# Fusarium_sp-PT_008919-RA Total prob of N-in: 0.00910

Fusarium_sp-PT_008919-RA TMHMM2.0 outside 1 921

# Fusarium_sp-PT_008920-RA Length: 159

# Fusarium_sp-PT_008920-RA Number of predicted TMHs: 0

# Fusarium_sp-PT_008920-RA Exp number of AAs in TMHs: 0.00035

# Fusarium_sp-PT_008920-RA Exp number, first 60 AAs: 0.00016

# Fusarium_sp-PT_008920-RA Total prob of N-in: 0.52866

Fusarium_sp-PT_008920-RA TMHMM2.0 inside 1 159

# Fusarium_sp-PT_008923-RA Length: 203

# Fusarium_sp-PT_008923-RA Number of predicted TMHs: 0

# Fusarium_sp-PT_008923-RA Exp number of AAs in TMHs: 1.46613

# Fusarium_sp-PT_008923-RA Exp number, first 60 AAs: 0.34631

# Fusarium_sp-PT_008923-RA Total prob of N-in: 0.09060

Fusarium_sp-PT_008923-RA TMHMM2.0 outside 1 203

# Fusarium_sp-PT_008924-RA Length: 525

# Fusarium_sp-PT_008924-RA Number of predicted TMHs: 0

# Fusarium_sp-PT_008924-RA Exp number of AAs in TMHs: 0.0226

# Fusarium_sp-PT_008924-RA Exp number, first 60 AAs: 0

# Fusarium_sp-PT_008924-RA Total prob of N-in: 0.00246

Fusarium_sp-PT_008924-RA TMHMM2.0 outside 1 525

# Fusarium_sp-PT_008926-RA Length: 534

# Fusarium_sp-PT_008926-RA Number of predicted TMHs: 12

# Fusarium_sp-PT_008926-RA Exp number of AAs in TMHs: 256.87442

# Fusarium_sp-PT_008926-RA Exp number, first 60 AAs: 6.64419

# Fusarium_sp-PT_008926-RA Total prob of N-in: 0.76265

Fusarium_sp-PT_008926-RA TMHMM2.0 inside 1 52

Fusarium_sp-PT_008926-RA TMHMM2.0 TMhelix 53 75

Fusarium_sp-PT_008926-RA TMHMM2.0 outside 76 84

Fusarium_sp-PT_008926-RA TMHMM2.0 TMhelix 85 107

Fusarium_sp-PT_008926-RA TMHMM2.0 inside 108 139

Fusarium_sp-PT_008926-RA TMHMM2.0 TMhelix 140 162

Fusarium_sp-PT_008926-RA TMHMM2.0 outside 163 176

Fusarium_sp-PT_008926-RA TMHMM2.0 TMhelix 177 199

Fusarium_sp-PT_008926-RA TMHMM2.0 inside 200 205

Fusarium_sp-PT_008926-RA TMHMM2.0 TMhelix 206 228

Fusarium_sp-PT_008926-RA TMHMM2.0 outside 229 252

Fusarium_sp-PT_008926-RA TMHMM2.0 TMhelix 253 275

Fusarium_sp-PT_008926-RA TMHMM2.0 inside 276 295

Fusarium_sp-PT_008926-RA TMHMM2.0 TMhelix 296 318

Fusarium_sp-PT_008926-RA TMHMM2.0 outside 319 337

Fusarium_sp-PT_008926-RA TMHMM2.0 TMhelix 338 360

Fusarium_sp-PT_008926-RA TMHMM2.0 inside 361 388

Fusarium_sp-PT_008926-RA TMHMM2.0 TMhelix 389 411

Fusarium_sp-PT_008926-RA TMHMM2.0 outside 412 414

Fusarium_sp-PT_008926-RA TMHMM2.0 TMhelix 415 437

Fusarium_sp-PT_008926-RA TMHMM2.0 inside 438 457

Fusarium_sp-PT_008926-RA TMHMM2.0 TMhelix 458 480

Fusarium_sp-PT_008926-RA TMHMM2.0 outside 481 489

Fusarium_sp-PT_008926-RA TMHMM2.0 TMhelix 490 509

Fusarium_sp-PT_008926-RA TMHMM2.0 inside 510 534

# Fusarium_sp-PT_008928-RA Length: 660

# Fusarium_sp-PT_008928-RA Number of predicted TMHs: 3

# Fusarium_sp-PT_008928-RA Exp number of AAs in TMHs: 82.6738200000001

# Fusarium_sp-PT_008928-RA Exp number, first 60 AAs: 11.41803

# Fusarium_sp-PT_008928-RA Total prob of N-in: 0.24992

# Fusarium_sp-PT_008928-RA POSSIBLE N-term signal sequence

Fusarium_sp-PT_008928-RA TMHMM2.0 outside 1 47

Fusarium_sp-PT_008928-RA TMHMM2.0 TMhelix 48 70

Fusarium_sp-PT_008928-RA TMHMM2.0 inside 71 82

Fusarium_sp-PT_008928-RA TMHMM2.0 TMhelix 83 105

Fusarium_sp-PT_008928-RA TMHMM2.0 outside 106 571

Fusarium_sp-PT_008928-RA TMHMM2.0 TMhelix 572 594

Fusarium_sp-PT_008928-RA TMHMM2.0 inside 595 660

# Fusarium_sp-PT_008931-RA Length: 406

# Fusarium_sp-PT_008931-RA Number of predicted TMHs: 0

# Fusarium_sp-PT_008931-RA Exp number of AAs in TMHs: 0.14495

# Fusarium_sp-PT_008931-RA Exp number, first 60 AAs: 0.00375

# Fusarium_sp-PT_008931-RA Total prob of N-in: 0.01263

Fusarium_sp-PT_008931-RA TMHMM2.0 outside 1 406

# Fusarium_sp-PT_008935-RA Length: 324

# Fusarium_sp-PT_008935-RA Number of predicted TMHs: 0

# Fusarium_sp-PT_008935-RA Exp number of AAs in TMHs: 0.01062

# Fusarium_sp-PT_008935-RA Exp number, first 60 AAs: 0.00156

# Fusarium_sp-PT_008935-RA Total prob of N-in: 0.01800

Fusarium_sp-PT_008935-RA TMHMM2.0 outside 1 324

# Fusarium_sp-PT_008937-RA Length: 331

# Fusarium_sp-PT_008937-RA Number of predicted TMHs: 0

# Fusarium_sp-PT_008937-RA Exp number of AAs in TMHs: 12.95814

# Fusarium_sp-PT_008937-RA Exp number, first 60 AAs: 0.00136

# Fusarium_sp-PT_008937-RA Total prob of N-in: 0.41248

Fusarium_sp-PT_008937-RA TMHMM2.0 outside 1 331

# Fusarium_sp-PT_008939-RA Length: 818

# Fusarium_sp-PT_008939-RA Number of predicted TMHs: 0

# Fusarium_sp-PT_008939-RA Exp number of AAs in TMHs: 0.0601900000000001

# Fusarium_sp-PT_008939-RA Exp number, first 60 AAs: 0.01264

# Fusarium_sp-PT_008939-RA Total prob of N-in: 0.00291

Fusarium_sp-PT_008939-RA TMHMM2.0 outside 1 818

# Fusarium_sp-PT_008940-RA Length: 920

# Fusarium_sp-PT_008940-RA Number of predicted TMHs: 0

# Fusarium_sp-PT_008940-RA Exp number of AAs in TMHs: 0.00491

# Fusarium_sp-PT_008940-RA Exp number, first 60 AAs: 0.00233

# Fusarium_sp-PT_008940-RA Total prob of N-in: 0.00015

Fusarium_sp-PT_008940-RA TMHMM2.0 outside 1 920

# Fusarium_sp-PT_008941-RA Length: 380

# Fusarium_sp-PT_008941-RA Number of predicted TMHs: 0

# Fusarium_sp-PT_008941-RA Exp number of AAs in TMHs: 0.02489

# Fusarium_sp-PT_008941-RA Exp number, first 60 AAs: 0.00096

# Fusarium_sp-PT_008941-RA Total prob of N-in: 0.00137

Fusarium_sp-PT_008941-RA TMHMM2.0 outside 1 380

# Fusarium_sp-PT_008945-RA Length: 376

# Fusarium_sp-PT_008945-RA Number of predicted TMHs: 6

# Fusarium_sp-PT_008945-RA Exp number of AAs in TMHs: 131.94101

# Fusarium_sp-PT_008945-RA Exp number, first 60 AAs: 35.41416

# Fusarium_sp-PT_008945-RA Total prob of N-in: 0.01199

# Fusarium_sp-PT_008945-RA POSSIBLE N-term signal sequence

Fusarium_sp-PT_008945-RA TMHMM2.0 outside 1 19

Fusarium_sp-PT_008945-RA TMHMM2.0 TMhelix 20 42

Fusarium_sp-PT_008945-RA TMHMM2.0 inside 43 48

Fusarium_sp-PT_008945-RA TMHMM2.0 TMhelix 49 71

Fusarium_sp-PT_008945-RA TMHMM2.0 outside 72 80

Fusarium_sp-PT_008945-RA TMHMM2.0 TMhelix 81 103

Fusarium_sp-PT_008945-RA TMHMM2.0 inside 104 115

Fusarium_sp-PT_008945-RA TMHMM2.0 TMhelix 116 138

Fusarium_sp-PT_008945-RA TMHMM2.0 outside 139 157

Fusarium_sp-PT_008945-RA TMHMM2.0 TMhelix 158 176

Fusarium_sp-PT_008945-RA TMHMM2.0 inside 177 196

Fusarium_sp-PT_008945-RA TMHMM2.0 TMhelix 197 216

Fusarium_sp-PT_008945-RA TMHMM2.0 outside 217 376

# Fusarium_sp-PT_008946-RA Length: 230

# Fusarium_sp-PT_008946-RA Number of predicted TMHs: 0

# Fusarium_sp-PT_008946-RA Exp number of AAs in TMHs: 0.00341

# Fusarium_sp-PT_008946-RA Exp number, first 60 AAs: 0

# Fusarium_sp-PT_008946-RA Total prob of N-in: 0.01601

Fusarium_sp-PT_008946-RA TMHMM2.0 outside 1 230

# Fusarium_sp-PT_008949-RA Length: 506

# Fusarium_sp-PT_008949-RA Number of predicted TMHs: 0

# Fusarium_sp-PT_008949-RA Exp number of AAs in TMHs: 0.26893

# Fusarium_sp-PT_008949-RA Exp number, first 60 AAs: 0.01083

# Fusarium_sp-PT_008949-RA Total prob of N-in: 0.00646

Fusarium_sp-PT_008949-RA TMHMM2.0 outside 1 506

# Fusarium_sp-PT_008917-RA Length: 545

# Fusarium_sp-PT_008917-RA Number of predicted TMHs: 11

# Fusarium_sp-PT_008917-RA Exp number of AAs in TMHs: 240.94603

# Fusarium_sp-PT_008917-RA Exp number, first 60 AAs: 23.44873

# Fusarium_sp-PT_008917-RA Total prob of N-in: 0.99172

# Fusarium_sp-PT_008917-RA POSSIBLE N-term signal sequence

Fusarium_sp-PT_008917-RA TMHMM2.0 inside 1 20

Fusarium_sp-PT_008917-RA TMHMM2.0 TMhelix 21 43

Fusarium_sp-PT_008917-RA TMHMM2.0 outside 44 71

Fusarium_sp-PT_008917-RA TMHMM2.0 TMhelix 72 94

Fusarium_sp-PT_008917-RA TMHMM2.0 inside 95 98

Fusarium_sp-PT_008917-RA TMHMM2.0 TMhelix 99 116

Fusarium_sp-PT_008917-RA TMHMM2.0 outside 117 125

Fusarium_sp-PT_008917-RA TMHMM2.0 TMhelix 126 148

Fusarium_sp-PT_008917-RA TMHMM2.0 inside 149 159

Fusarium_sp-PT_008917-RA TMHMM2.0 TMhelix 160 182

Fusarium_sp-PT_008917-RA TMHMM2.0 outside 183 196

Fusarium_sp-PT_008917-RA TMHMM2.0 TMhelix 197 219

Fusarium_sp-PT_008917-RA TMHMM2.0 inside 220 289

Fusarium_sp-PT_008917-RA TMHMM2.0 TMhelix 290 312

Fusarium_sp-PT_008917-RA TMHMM2.0 outside 313 326

Fusarium_sp-PT_008917-RA TMHMM2.0 TMhelix 327 349

Fusarium_sp-PT_008917-RA TMHMM2.0 inside 350 355

Fusarium_sp-PT_008917-RA TMHMM2.0 TMhelix 356 378

Fusarium_sp-PT_008917-RA TMHMM2.0 outside 379 392

Fusarium_sp-PT_008917-RA TMHMM2.0 TMhelix 393 415

Fusarium_sp-PT_008917-RA TMHMM2.0 inside 416 459

Fusarium_sp-PT_008917-RA TMHMM2.0 TMhelix 460 482

Fusarium_sp-PT_008917-RA TMHMM2.0 outside 483 545

# Fusarium_sp-PT_008918-RA Length: 749

# Fusarium_sp-PT_008918-RA Number of predicted TMHs: 0

# Fusarium_sp-PT_008918-RA Exp number of AAs in TMHs: 0.01624

# Fusarium_sp-PT_008918-RA Exp number, first 60 AAs: 0

# Fusarium_sp-PT_008918-RA Total prob of N-in: 0.00075

Fusarium_sp-PT_008918-RA TMHMM2.0 outside 1 749

# Fusarium_sp-PT_008921-RA Length: 200

# Fusarium_sp-PT_008921-RA Number of predicted TMHs: 0

# Fusarium_sp-PT_008921-RA Exp number of AAs in TMHs: 0.41215

# Fusarium_sp-PT_008921-RA Exp number, first 60 AAs: 0.04814

# Fusarium_sp-PT_008921-RA Total prob of N-in: 0.05134

Fusarium_sp-PT_008921-RA TMHMM2.0 outside 1 200

# Fusarium_sp-PT_008922-RA Length: 542

# Fusarium_sp-PT_008922-RA Number of predicted TMHs: 0

# Fusarium_sp-PT_008922-RA Exp number of AAs in TMHs: 1.03112

# Fusarium_sp-PT_008922-RA Exp number, first 60 AAs: 0.0397

# Fusarium_sp-PT_008922-RA Total prob of N-in: 0.03352

Fusarium_sp-PT_008922-RA TMHMM2.0 outside 1 542

# Fusarium_sp-PT_008929-RA Length: 346

# Fusarium_sp-PT_008929-RA Number of predicted TMHs: 5

# Fusarium_sp-PT_008929-RA Exp number of AAs in TMHs: 108.67687

# Fusarium_sp-PT_008929-RA Exp number, first 60 AAs: 33.68446

# Fusarium_sp-PT_008929-RA Total prob of N-in: 0.00733

# Fusarium_sp-PT_008929-RA POSSIBLE N-term signal sequence

Fusarium_sp-PT_008929-RA TMHMM2.0 outside 1 19

Fusarium_sp-PT_008929-RA TMHMM2.0 TMhelix 20 39

Fusarium_sp-PT_008929-RA TMHMM2.0 inside 40 45

Fusarium_sp-PT_008929-RA TMHMM2.0 TMhelix 46 64

Fusarium_sp-PT_008929-RA TMHMM2.0 outside 65 73

Fusarium_sp-PT_008929-RA TMHMM2.0 TMhelix 74 96

Fusarium_sp-PT_008929-RA TMHMM2.0 inside 97 104

Fusarium_sp-PT_008929-RA TMHMM2.0 TMhelix 105 127

Fusarium_sp-PT_008929-RA TMHMM2.0 outside 128 184

Fusarium_sp-PT_008929-RA TMHMM2.0 TMhelix 185 207

Fusarium_sp-PT_008929-RA TMHMM2.0 inside 208 346

# Fusarium_sp-PT_008930-RA Length: 574

# Fusarium_sp-PT_008930-RA Number of predicted TMHs: 1

# Fusarium_sp-PT_008930-RA Exp number of AAs in TMHs: 21.42586

# Fusarium_sp-PT_008930-RA Exp number, first 60 AAs: 0

# Fusarium_sp-PT_008930-RA Total prob of N-in: 0.00247

Fusarium_sp-PT_008930-RA TMHMM2.0 outside 1 510

Fusarium_sp-PT_008930-RA TMHMM2.0 TMhelix 511 533

Fusarium_sp-PT_008930-RA TMHMM2.0 inside 534 574

# Fusarium_sp-PT_008934-RA Length: 324

# Fusarium_sp-PT_008934-RA Number of predicted TMHs: 0

# Fusarium_sp-PT_008934-RA Exp number of AAs in TMHs: 0.06789

# Fusarium_sp-PT_008934-RA Exp number, first 60 AAs: 0.00341

# Fusarium_sp-PT_008934-RA Total prob of N-in: 0.01981

Fusarium_sp-PT_008934-RA TMHMM2.0 outside 1 324

# Fusarium_sp-PT_008936-RA Length: 150

# Fusarium_sp-PT_008936-RA Number of predicted TMHs: 0

# Fusarium_sp-PT_008936-RA Exp number of AAs in TMHs: 2.6036

# Fusarium_sp-PT_008936-RA Exp number, first 60 AAs: 0.00023

# Fusarium_sp-PT_008936-RA Total prob of N-in: 0.17090

Fusarium_sp-PT_008936-RA TMHMM2.0 outside 1 150

# Fusarium_sp-PT_008938-RA Length: 522

# Fusarium_sp-PT_008938-RA Number of predicted TMHs: 0

# Fusarium_sp-PT_008938-RA Exp number of AAs in TMHs: 0.0596700000000001

# Fusarium_sp-PT_008938-RA Exp number, first 60 AAs: 0.00038

# Fusarium_sp-PT_008938-RA Total prob of N-in: 0.00519

Fusarium_sp-PT_008938-RA TMHMM2.0 outside 1 522

# Fusarium_sp-PT_008943-RA Length: 582

# Fusarium_sp-PT_008943-RA Number of predicted TMHs: 0

# Fusarium_sp-PT_008943-RA Exp number of AAs in TMHs: 0

# Fusarium_sp-PT_008943-RA Exp number, first 60 AAs: 0

# Fusarium_sp-PT_008943-RA Total prob of N-in: 0.00054

Fusarium_sp-PT_008943-RA TMHMM2.0 outside 1 582

# Fusarium_sp-PT_008944-RA Length: 413

# Fusarium_sp-PT_008944-RA Number of predicted TMHs: 0

# Fusarium_sp-PT_008944-RA Exp number of AAs in TMHs: 0.0339800000000001

# Fusarium_sp-PT_008944-RA Exp number, first 60 AAs: 0.00011

# Fusarium_sp-PT_008944-RA Total prob of N-in: 0.00363

Fusarium_sp-PT_008944-RA TMHMM2.0 outside 1 413

# Fusarium_sp-PT_008947-RA Length: 503

# Fusarium_sp-PT_008947-RA Number of predicted TMHs: 11

# Fusarium_sp-PT_008947-RA Exp number of AAs in TMHs: 238.47343

# Fusarium_sp-PT_008947-RA Exp number, first 60 AAs: 1.77184

# Fusarium_sp-PT_008947-RA Total prob of N-in: 0.10770

Fusarium_sp-PT_008947-RA TMHMM2.0 outside 1 84

Fusarium_sp-PT_008947-RA TMHMM2.0 TMhelix 85 107

Fusarium_sp-PT_008947-RA TMHMM2.0 inside 108 118

Fusarium_sp-PT_008947-RA TMHMM2.0 TMhelix 119 141

Fusarium_sp-PT_008947-RA TMHMM2.0 outside 142 144

Fusarium_sp-PT_008947-RA TMHMM2.0 TMhelix 145 164

Fusarium_sp-PT_008947-RA TMHMM2.0 inside 165 175

Fusarium_sp-PT_008947-RA TMHMM2.0 TMhelix 176 198

Fusarium_sp-PT_008947-RA TMHMM2.0 outside 199 207

Fusarium_sp-PT_008947-RA TMHMM2.0 TMhelix 208 230

Fusarium_sp-PT_008947-RA TMHMM2.0 inside 231 275

Fusarium_sp-PT_008947-RA TMHMM2.0 TMhelix 276 298

Fusarium_sp-PT_008947-RA TMHMM2.0 outside 299 312

Fusarium_sp-PT_008947-RA TMHMM2.0 TMhelix 313 332

Fusarium_sp-PT_008947-RA TMHMM2.0 inside 333 343

Fusarium_sp-PT_008947-RA TMHMM2.0 TMhelix 344 361

Fusarium_sp-PT_008947-RA TMHMM2.0 outside 362 365

Fusarium_sp-PT_008947-RA TMHMM2.0 TMhelix 366 385

Fusarium_sp-PT_008947-RA TMHMM2.0 inside 386 397

Fusarium_sp-PT_008947-RA TMHMM2.0 TMhelix 398 420

Fusarium_sp-PT_008947-RA TMHMM2.0 outside 421 429

Fusarium_sp-PT_008947-RA TMHMM2.0 TMhelix 430 452

Fusarium_sp-PT_008947-RA TMHMM2.0 inside 453 503

# Fusarium_sp-PT_008948-RA Length: 422

# Fusarium_sp-PT_008948-RA Number of predicted TMHs: 3

# Fusarium_sp-PT_008948-RA Exp number of AAs in TMHs: 62.76265

# Fusarium_sp-PT_008948-RA Exp number, first 60 AAs: 1.85562

# Fusarium_sp-PT_008948-RA Total prob of N-in: 0.83073

Fusarium_sp-PT_008948-RA TMHMM2.0 inside 1 74

Fusarium_sp-PT_008948-RA TMHMM2.0 TMhelix 75 97

Fusarium_sp-PT_008948-RA TMHMM2.0 outside 98 116

Fusarium_sp-PT_008948-RA TMHMM2.0 TMhelix 117 139

Fusarium_sp-PT_008948-RA TMHMM2.0 inside 140 159

Fusarium_sp-PT_008948-RA TMHMM2.0 TMhelix 160 182

Fusarium_sp-PT_008948-RA TMHMM2.0 outside 183 422

# Fusarium_sp-PT_008950-RA Length: 1280

# Fusarium_sp-PT_008950-RA Number of predicted TMHs: 10

# Fusarium_sp-PT_008950-RA Exp number of AAs in TMHs: 227.81967

# Fusarium_sp-PT_008950-RA Exp number, first 60 AAs: 0.49191

# Fusarium_sp-PT_008950-RA Total prob of N-in: 0.02444

Fusarium_sp-PT_008950-RA TMHMM2.0 outside 1 79

Fusarium_sp-PT_008950-RA TMHMM2.0 TMhelix 80 102

Fusarium_sp-PT_008950-RA TMHMM2.0 inside 103 163

Fusarium_sp-PT_008950-RA TMHMM2.0 TMhelix 164 186

Fusarium_sp-PT_008950-RA TMHMM2.0 outside 187 226

Fusarium_sp-PT_008950-RA TMHMM2.0 TMhelix 227 249

Fusarium_sp-PT_008950-RA TMHMM2.0 inside 250 748

Fusarium_sp-PT_008950-RA TMHMM2.0 TMhelix 749 771

Fusarium_sp-PT_008950-RA TMHMM2.0 outside 772 799

Fusarium_sp-PT_008950-RA TMHMM2.0 TMhelix 800 822

Fusarium_sp-PT_008950-RA TMHMM2.0 inside 823 842

Fusarium_sp-PT_008950-RA TMHMM2.0 TMhelix 843 865

Fusarium_sp-PT_008950-RA TMHMM2.0 outside 866 902

Fusarium_sp-PT_008950-RA TMHMM2.0 TMhelix 903 925

Fusarium_sp-PT_008950-RA TMHMM2.0 inside 926 959

Fusarium_sp-PT_008950-RA TMHMM2.0 TMhelix 960 982

Fusarium_sp-PT_008950-RA TMHMM2.0 outside 983 1007

Fusarium_sp-PT_008950-RA TMHMM2.0 TMhelix 1008 1030

Fusarium_sp-PT_008950-RA TMHMM2.0 inside 1031 1042

Fusarium_sp-PT_008950-RA TMHMM2.0 TMhelix 1043 1065

Fusarium_sp-PT_008950-RA TMHMM2.0 outside 1066 1280

# Fusarium_sp-PT_008927-RA Length: 744

# Fusarium_sp-PT_008927-RA Number of predicted TMHs: 7

# Fusarium_sp-PT_008927-RA Exp number of AAs in TMHs: 166.18878

# Fusarium_sp-PT_008927-RA Exp number, first 60 AAs: 0.65846

# Fusarium_sp-PT_008927-RA Total prob of N-in: 0.74416

Fusarium_sp-PT_008927-RA TMHMM2.0 outside 1 174

Fusarium_sp-PT_008927-RA TMHMM2.0 TMhelix 175 197

Fusarium_sp-PT_008927-RA TMHMM2.0 inside 198 311

Fusarium_sp-PT_008927-RA TMHMM2.0 TMhelix 312 334

Fusarium_sp-PT_008927-RA TMHMM2.0 outside 335 348

Fusarium_sp-PT_008927-RA TMHMM2.0 TMhelix 349 371

Fusarium_sp-PT_008927-RA TMHMM2.0 inside 372 383

Fusarium_sp-PT_008927-RA TMHMM2.0 TMhelix 384 402

Fusarium_sp-PT_008927-RA TMHMM2.0 outside 403 458

Fusarium_sp-PT_008927-RA TMHMM2.0 TMhelix 459 478

Fusarium_sp-PT_008927-RA TMHMM2.0 inside 479 484

Fusarium_sp-PT_008927-RA TMHMM2.0 TMhelix 485 507

Fusarium_sp-PT_008927-RA TMHMM2.0 outside 508 516

Fusarium_sp-PT_008927-RA TMHMM2.0 TMhelix 517 539

Fusarium_sp-PT_008927-RA TMHMM2.0 inside 540 744

# Fusarium_sp-PT_008932-RA Length: 254

# Fusarium_sp-PT_008932-RA Number of predicted TMHs: 0

# Fusarium_sp-PT_008932-RA Exp number of AAs in TMHs: 0.03014

# Fusarium_sp-PT_008932-RA Exp number, first 60 AAs: 0.00123

# Fusarium_sp-PT_008932-RA Total prob of N-in: 0.00603

Fusarium_sp-PT_008932-RA TMHMM2.0 outside 1 254

# Fusarium_sp-PT_008925-RA Length: 167

# Fusarium_sp-PT_008925-RA Number of predicted TMHs: 1

# Fusarium_sp-PT_008925-RA Exp number of AAs in TMHs: 19.09375

# Fusarium_sp-PT_008925-RA Exp number, first 60 AAs: 0

# Fusarium_sp-PT_008925-RA Total prob of N-in: 0.81542

Fusarium_sp-PT_008925-RA TMHMM2.0 inside 1 142

Fusarium_sp-PT_008925-RA TMHMM2.0 TMhelix 143 165

Fusarium_sp-PT_008925-RA TMHMM2.0 outside 166 167

# Fusarium_sp-PT_008942-RA Length: 115

# Fusarium_sp-PT_008942-RA Number of predicted TMHs: 0

# Fusarium_sp-PT_008942-RA Exp number of AAs in TMHs: 0.01061

# Fusarium_sp-PT_008942-RA Exp number, first 60 AAs: 0.01049

# Fusarium_sp-PT_008942-RA Total prob of N-in: 0.41307

Fusarium_sp-PT_008942-RA TMHMM2.0 outside 1 115

# Fusarium_sp-PT_008933-RA Length: 48

# Fusarium_sp-PT_008933-RA Number of predicted TMHs: 0

# Fusarium_sp-PT_008933-RA Exp number of AAs in TMHs: 0

# Fusarium_sp-PT_008933-RA Exp number, first 60 AAs: 0

# Fusarium_sp-PT_008933-RA Total prob of N-in: 0.77526

Fusarium_sp-PT_008933-RA TMHMM2.0 inside 1 48

# Fusarium_sp-PT_008955-RA Length: 740

# Fusarium_sp-PT_008955-RA Number of predicted TMHs: 0

# Fusarium_sp-PT_008955-RA Exp number of AAs in TMHs: 0.00056

# Fusarium_sp-PT_008955-RA Exp number, first 60 AAs: 0

# Fusarium_sp-PT_008955-RA Total prob of N-in: 0.00071

Fusarium_sp-PT_008955-RA TMHMM2.0 outside 1 740

# Fusarium_sp-PT_008956-RA Length: 238

# Fusarium_sp-PT_008956-RA Number of predicted TMHs: 0

# Fusarium_sp-PT_008956-RA Exp number of AAs in TMHs: 0.0172

# Fusarium_sp-PT_008956-RA Exp number, first 60 AAs: 0

# Fusarium_sp-PT_008956-RA Total prob of N-in: 0.18404

Fusarium_sp-PT_008956-RA TMHMM2.0 outside 1 238

# Fusarium_sp-PT_008958-RA Length: 448

# Fusarium_sp-PT_008958-RA Number of predicted TMHs: 1

# Fusarium_sp-PT_008958-RA Exp number of AAs in TMHs: 18.91383

# Fusarium_sp-PT_008958-RA Exp number, first 60 AAs: 0

# Fusarium_sp-PT_008958-RA Total prob of N-in: 0.86999

Fusarium_sp-PT_008958-RA TMHMM2.0 inside 1 72

Fusarium_sp-PT_008958-RA TMHMM2.0 TMhelix 73 95

Fusarium_sp-PT_008958-RA TMHMM2.0 outside 96 448

# Fusarium_sp-PT_008960-RA Length: 541

# Fusarium_sp-PT_008960-RA Number of predicted TMHs: 0

# Fusarium_sp-PT_008960-RA Exp number of AAs in TMHs: 0.00082

# Fusarium_sp-PT_008960-RA Exp number, first 60 AAs: 0

# Fusarium_sp-PT_008960-RA Total prob of N-in: 0.00063

Fusarium_sp-PT_008960-RA TMHMM2.0 outside 1 541

# Fusarium_sp-PT_008961-RA Length: 867

# Fusarium_sp-PT_008961-RA Number of predicted TMHs: 0

# Fusarium_sp-PT_008961-RA Exp number of AAs in TMHs: 0.01541

# Fusarium_sp-PT_008961-RA Exp number, first 60 AAs: 0.00018

# Fusarium_sp-PT_008961-RA Total prob of N-in: 0.00067

Fusarium_sp-PT_008961-RA TMHMM2.0 outside 1 867

# Fusarium_sp-PT_008962-RA Length: 258

# Fusarium_sp-PT_008962-RA Number of predicted TMHs: 0

# Fusarium_sp-PT_008962-RA Exp number of AAs in TMHs: 0.00431

# Fusarium_sp-PT_008962-RA Exp number, first 60 AAs: 0.00016

# Fusarium_sp-PT_008962-RA Total prob of N-in: 0.06752

Fusarium_sp-PT_008962-RA TMHMM2.0 outside 1 258

# Fusarium_sp-PT_008964-RA Length: 238

# Fusarium_sp-PT_008964-RA Number of predicted TMHs: 0

# Fusarium_sp-PT_008964-RA Exp number of AAs in TMHs: 0.01961

# Fusarium_sp-PT_008964-RA Exp number, first 60 AAs: 0.00672

# Fusarium_sp-PT_008964-RA Total prob of N-in: 0.01653

Fusarium_sp-PT_008964-RA TMHMM2.0 outside 1 238

# Fusarium_sp-PT_008969-RA Length: 297

# Fusarium_sp-PT_008969-RA Number of predicted TMHs: 0

# Fusarium_sp-PT_008969-RA Exp number of AAs in TMHs: 1.85347

# Fusarium_sp-PT_008969-RA Exp number, first 60 AAs: 0.00558

# Fusarium_sp-PT_008969-RA Total prob of N-in: 0.17395

Fusarium_sp-PT_008969-RA TMHMM2.0 outside 1 297

# Fusarium_sp-PT_008971-RA Length: 532

# Fusarium_sp-PT_008971-RA Number of predicted TMHs: 12

# Fusarium_sp-PT_008971-RA Exp number of AAs in TMHs: 258.38057

# Fusarium_sp-PT_008971-RA Exp number, first 60 AAs: 21.09261

# Fusarium_sp-PT_008971-RA Total prob of N-in: 0.99380

# Fusarium_sp-PT_008971-RA POSSIBLE N-term signal sequence

Fusarium_sp-PT_008971-RA TMHMM2.0 inside 1 11

Fusarium_sp-PT_008971-RA TMHMM2.0 TMhelix 12 31

Fusarium_sp-PT_008971-RA TMHMM2.0 outside 32 61

Fusarium_sp-PT_008971-RA TMHMM2.0 TMhelix 62 84

Fusarium_sp-PT_008971-RA TMHMM2.0 inside 85 96

Fusarium_sp-PT_008971-RA TMHMM2.0 TMhelix 97 119

Fusarium_sp-PT_008971-RA TMHMM2.0 outside 120 122

Fusarium_sp-PT_008971-RA TMHMM2.0 TMhelix 123 140

Fusarium_sp-PT_008971-RA TMHMM2.0 inside 141 152

Fusarium_sp-PT_008971-RA TMHMM2.0 TMhelix 153 175

Fusarium_sp-PT_008971-RA TMHMM2.0 outside 176 184

Fusarium_sp-PT_008971-RA TMHMM2.0 TMhelix 185 207

Fusarium_sp-PT_008971-RA TMHMM2.0 inside 208 276

Fusarium_sp-PT_008971-RA TMHMM2.0 TMhelix 277 299

Fusarium_sp-PT_008971-RA TMHMM2.0 outside 300 313

Fusarium_sp-PT_008971-RA TMHMM2.0 TMhelix 314 336

Fusarium_sp-PT_008971-RA TMHMM2.0 inside 337 342

Fusarium_sp-PT_008971-RA TMHMM2.0 TMhelix 343 365

Fusarium_sp-PT_008971-RA TMHMM2.0 outside 366 374

Fusarium_sp-PT_008971-RA TMHMM2.0 TMhelix 375 397

Fusarium_sp-PT_008971-RA TMHMM2.0 inside 398 417

Fusarium_sp-PT_008971-RA TMHMM2.0 TMhelix 418 437

Fusarium_sp-PT_008971-RA TMHMM2.0 outside 438 440

Fusarium_sp-PT_008971-RA TMHMM2.0 TMhelix 441 463

Fusarium_sp-PT_008971-RA TMHMM2.0 inside 464 532

# Fusarium_sp-PT_008972-RA Length: 545

# Fusarium_sp-PT_008972-RA Number of predicted TMHs: 0

# Fusarium_sp-PT_008972-RA Exp number of AAs in TMHs: 13.2018

# Fusarium_sp-PT_008972-RA Exp number, first 60 AAs: 13.20098

# Fusarium_sp-PT_008972-RA Total prob of N-in: 0.62462

# Fusarium_sp-PT_008972-RA POSSIBLE N-term signal sequence

Fusarium_sp-PT_008972-RA TMHMM2.0 outside 1 545

# Fusarium_sp-PT_008974-RA Length: 701

# Fusarium_sp-PT_008974-RA Number of predicted TMHs: 0

# Fusarium_sp-PT_008974-RA Exp number of AAs in TMHs: 0.00837

# Fusarium_sp-PT_008974-RA Exp number, first 60 AAs: 0

# Fusarium_sp-PT_008974-RA Total prob of N-in: 0.00016

Fusarium_sp-PT_008974-RA TMHMM2.0 outside 1 701

# Fusarium_sp-PT_008976-RA Length: 922

# Fusarium_sp-PT_008976-RA Number of predicted TMHs: 9

# Fusarium_sp-PT_008976-RA Exp number of AAs in TMHs: 187.93983

# Fusarium_sp-PT_008976-RA Exp number, first 60 AAs: 0

# Fusarium_sp-PT_008976-RA Total prob of N-in: 0.02816

Fusarium_sp-PT_008976-RA TMHMM2.0 outside 1 539

Fusarium_sp-PT_008976-RA TMHMM2.0 TMhelix 540 559

Fusarium_sp-PT_008976-RA TMHMM2.0 inside 560 565

Fusarium_sp-PT_008976-RA TMHMM2.0 TMhelix 566 584

Fusarium_sp-PT_008976-RA TMHMM2.0 outside 585 587

Fusarium_sp-PT_008976-RA TMHMM2.0 TMhelix 588 610

Fusarium_sp-PT_008976-RA TMHMM2.0 inside 611 616

Fusarium_sp-PT_008976-RA TMHMM2.0 TMhelix 617 639

Fusarium_sp-PT_008976-RA TMHMM2.0 outside 640 653

Fusarium_sp-PT_008976-RA TMHMM2.0 TMhelix 654 676

Fusarium_sp-PT_008976-RA TMHMM2.0 inside 677 696

Fusarium_sp-PT_008976-RA TMHMM2.0 TMhelix 697 716

Fusarium_sp-PT_008976-RA TMHMM2.0 outside 717 740

Fusarium_sp-PT_008976-RA TMHMM2.0 TMhelix 741 763

Fusarium_sp-PT_008976-RA TMHMM2.0 inside 764 825

Fusarium_sp-PT_008976-RA TMHMM2.0 TMhelix 826 848

Fusarium_sp-PT_008976-RA TMHMM2.0 outside 849 890

Fusarium_sp-PT_008976-RA TMHMM2.0 TMhelix 891 913

Fusarium_sp-PT_008976-RA TMHMM2.0 inside 914 922

# Fusarium_sp-PT_008977-RA Length: 341

# Fusarium_sp-PT_008977-RA Number of predicted TMHs: 0

# Fusarium_sp-PT_008977-RA Exp number of AAs in TMHs: 2.22532000000001

# Fusarium_sp-PT_008977-RA Exp number, first 60 AAs: 2.20242

# Fusarium_sp-PT_008977-RA Total prob of N-in: 0.15472

Fusarium_sp-PT_008977-RA TMHMM2.0 outside 1 341

# Fusarium_sp-PT_008979-RA Length: 360

# Fusarium_sp-PT_008979-RA Number of predicted TMHs: 0

# Fusarium_sp-PT_008979-RA Exp number of AAs in TMHs: 0.11508

# Fusarium_sp-PT_008979-RA Exp number, first 60 AAs: 0.00108

# Fusarium_sp-PT_008979-RA Total prob of N-in: 0.01950

Fusarium_sp-PT_008979-RA TMHMM2.0 outside 1 360

# Fusarium_sp-PT_008980-RA Length: 456

# Fusarium_sp-PT_008980-RA Number of predicted TMHs: 0

# Fusarium_sp-PT_008980-RA Exp number of AAs in TMHs: 1.77698

# Fusarium_sp-PT_008980-RA Exp number, first 60 AAs: 3e-05

# Fusarium_sp-PT_008980-RA Total prob of N-in: 0.07381

Fusarium_sp-PT_008980-RA TMHMM2.0 outside 1 456

# Fusarium_sp-PT_008982-RA Length: 415

# Fusarium_sp-PT_008982-RA Number of predicted TMHs: 0

# Fusarium_sp-PT_008982-RA Exp number of AAs in TMHs: 0.62394

# Fusarium_sp-PT_008982-RA Exp number, first 60 AAs: 0.62394

# Fusarium_sp-PT_008982-RA Total prob of N-in: 0.03925

Fusarium_sp-PT_008982-RA TMHMM2.0 outside 1 415

# Fusarium_sp-PT_008951-RA Length: 503

# Fusarium_sp-PT_008951-RA Number of predicted TMHs: 0

# Fusarium_sp-PT_008951-RA Exp number of AAs in TMHs: 0.72083

# Fusarium_sp-PT_008951-RA Exp number, first 60 AAs: 0.70986

# Fusarium_sp-PT_008951-RA Total prob of N-in: 0.03872

Fusarium_sp-PT_008951-RA TMHMM2.0 outside 1 503

# Fusarium_sp-PT_008952-RA Length: 297

# Fusarium_sp-PT_008952-RA Number of predicted TMHs: 2

# Fusarium_sp-PT_008952-RA Exp number of AAs in TMHs: 43.91836

# Fusarium_sp-PT_008952-RA Exp number, first 60 AAs: 0.01066

# Fusarium_sp-PT_008952-RA Total prob of N-in: 0.95563

Fusarium_sp-PT_008952-RA TMHMM2.0 inside 1 108

Fusarium_sp-PT_008952-RA TMHMM2.0 TMhelix 109 131

Fusarium_sp-PT_008952-RA TMHMM2.0 outside 132 140

Fusarium_sp-PT_008952-RA TMHMM2.0 TMhelix 141 163

Fusarium_sp-PT_008952-RA TMHMM2.0 inside 164 297

# Fusarium_sp-PT_008953-RA Length: 449

# Fusarium_sp-PT_008953-RA Number of predicted TMHs: 0

# Fusarium_sp-PT_008953-RA Exp number of AAs in TMHs: 0.04904

# Fusarium_sp-PT_008953-RA Exp number, first 60 AAs: 0.00601999999999999

# Fusarium_sp-PT_008953-RA Total prob of N-in: 0.00346

Fusarium_sp-PT_008953-RA TMHMM2.0 outside 1 449

# Fusarium_sp-PT_008954-RA Length: 394

# Fusarium_sp-PT_008954-RA Number of predicted TMHs: 2

# Fusarium_sp-PT_008954-RA Exp number of AAs in TMHs: 40.84968

# Fusarium_sp-PT_008954-RA Exp number, first 60 AAs: 0.00508

# Fusarium_sp-PT_008954-RA Total prob of N-in: 0.43796

Fusarium_sp-PT_008954-RA TMHMM2.0 outside 1 294

Fusarium_sp-PT_008954-RA TMHMM2.0 TMhelix 295 317

Fusarium_sp-PT_008954-RA TMHMM2.0 inside 318 323

Fusarium_sp-PT_008954-RA TMHMM2.0 TMhelix 324 343

Fusarium_sp-PT_008954-RA TMHMM2.0 outside 344 394

# Fusarium_sp-PT_008957-RA Length: 195

# Fusarium_sp-PT_008957-RA Number of predicted TMHs: 0

# Fusarium_sp-PT_008957-RA Exp number of AAs in TMHs: 0

# Fusarium_sp-PT_008957-RA Exp number, first 60 AAs: 0

# Fusarium_sp-PT_008957-RA Total prob of N-in: 0.03623

Fusarium_sp-PT_008957-RA TMHMM2.0 outside 1 195

# Fusarium_sp-PT_008959-RA Length: 231

# Fusarium_sp-PT_008959-RA Number of predicted TMHs: 2

# Fusarium_sp-PT_008959-RA Exp number of AAs in TMHs: 45.8687

# Fusarium_sp-PT_008959-RA Exp number, first 60 AAs: 0.01487

# Fusarium_sp-PT_008959-RA Total prob of N-in: 0.99689

Fusarium_sp-PT_008959-RA TMHMM2.0 inside 1 98

Fusarium_sp-PT_008959-RA TMHMM2.0 TMhelix 99 121

Fusarium_sp-PT_008959-RA TMHMM2.0 outside 122 140

Fusarium_sp-PT_008959-RA TMHMM2.0 TMhelix 141 163

Fusarium_sp-PT_008959-RA TMHMM2.0 inside 164 231

# Fusarium_sp-PT_008963-RA Length: 311

# Fusarium_sp-PT_008963-RA Number of predicted TMHs: 0

# Fusarium_sp-PT_008963-RA Exp number of AAs in TMHs: 0.299

# Fusarium_sp-PT_008963-RA Exp number, first 60 AAs: 0.00810999999999999

# Fusarium_sp-PT_008963-RA Total prob of N-in: 0.10511

Fusarium_sp-PT_008963-RA TMHMM2.0 outside 1 311

# Fusarium_sp-PT_008965-RA Length: 662

# Fusarium_sp-PT_008965-RA Number of predicted TMHs: 0

# Fusarium_sp-PT_008965-RA Exp number of AAs in TMHs: 0.28055

# Fusarium_sp-PT_008965-RA Exp number, first 60 AAs: 0.01892

# Fusarium_sp-PT_008965-RA Total prob of N-in: 0.01156

Fusarium_sp-PT_008965-RA TMHMM2.0 outside 1 662

# Fusarium_sp-PT_008966-RA Length: 94

# Fusarium_sp-PT_008966-RA Number of predicted TMHs: 1

# Fusarium_sp-PT_008966-RA Exp number of AAs in TMHs: 19.31575

# Fusarium_sp-PT_008966-RA Exp number, first 60 AAs: 7.43742

# Fusarium_sp-PT_008966-RA Total prob of N-in: 0.55503

Fusarium_sp-PT_008966-RA TMHMM2.0 inside 1 53

Fusarium_sp-PT_008966-RA TMHMM2.0 TMhelix 54 73

Fusarium_sp-PT_008966-RA TMHMM2.0 outside 74 94

# Fusarium_sp-PT_008967-RA Length: 462

# Fusarium_sp-PT_008967-RA Number of predicted TMHs: 0

# Fusarium_sp-PT_008967-RA Exp number of AAs in TMHs: 0.00713999999999999

# Fusarium_sp-PT_008967-RA Exp number, first 60 AAs: 0.00298

# Fusarium_sp-PT_008967-RA Total prob of N-in: 0.01706

Fusarium_sp-PT_008967-RA TMHMM2.0 outside 1 462

# Fusarium_sp-PT_008968-RA Length: 406

# Fusarium_sp-PT_008968-RA Number of predicted TMHs: 6

# Fusarium_sp-PT_008968-RA Exp number of AAs in TMHs: 134.85516

# Fusarium_sp-PT_008968-RA Exp number, first 60 AAs: 27.11651

# Fusarium_sp-PT_008968-RA Total prob of N-in: 0.02290

# Fusarium_sp-PT_008968-RA POSSIBLE N-term signal sequence

Fusarium_sp-PT_008968-RA TMHMM2.0 outside 1 19

Fusarium_sp-PT_008968-RA TMHMM2.0 TMhelix 20 42

Fusarium_sp-PT_008968-RA TMHMM2.0 inside 43 54

Fusarium_sp-PT_008968-RA TMHMM2.0 TMhelix 55 77

Fusarium_sp-PT_008968-RA TMHMM2.0 outside 78 91

Fusarium_sp-PT_008968-RA TMHMM2.0 TMhelix 92 112

Fusarium_sp-PT_008968-RA TMHMM2.0 inside 113 132

Fusarium_sp-PT_008968-RA TMHMM2.0 TMhelix 133 155

Fusarium_sp-PT_008968-RA TMHMM2.0 outside 156 174

Fusarium_sp-PT_008968-RA TMHMM2.0 TMhelix 175 197

Fusarium_sp-PT_008968-RA TMHMM2.0 inside 198 209

Fusarium_sp-PT_008968-RA TMHMM2.0 TMhelix 210 229

Fusarium_sp-PT_008968-RA TMHMM2.0 outside 230 406

# Fusarium_sp-PT_008970-RA Length: 477

# Fusarium_sp-PT_008970-RA Number of predicted TMHs: 0

# Fusarium_sp-PT_008970-RA Exp number of AAs in TMHs: 0.04911

# Fusarium_sp-PT_008970-RA Exp number, first 60 AAs: 0

# Fusarium_sp-PT_008970-RA Total prob of N-in: 0.01443

Fusarium_sp-PT_008970-RA TMHMM2.0 outside 1 477

# Fusarium_sp-PT_008973-RA Length: 312

# Fusarium_sp-PT_008973-RA Number of predicted TMHs: 0

# Fusarium_sp-PT_008973-RA Exp number of AAs in TMHs: 0.02392

# Fusarium_sp-PT_008973-RA Exp number, first 60 AAs: 0.000900000000000001

# Fusarium_sp-PT_008973-RA Total prob of N-in: 0.01940

Fusarium_sp-PT_008973-RA TMHMM2.0 outside 1 312

# Fusarium_sp-PT_008975-RA Length: 276

# Fusarium_sp-PT_008975-RA Number of predicted TMHs: 1

# Fusarium_sp-PT_008975-RA Exp number of AAs in TMHs: 21.25701

# Fusarium_sp-PT_008975-RA Exp number, first 60 AAs: 0.00157

# Fusarium_sp-PT_008975-RA Total prob of N-in: 0.88859

Fusarium_sp-PT_008975-RA TMHMM2.0 inside 1 245

Fusarium_sp-PT_008975-RA TMHMM2.0 TMhelix 246 268

Fusarium_sp-PT_008975-RA TMHMM2.0 outside 269 276

# Fusarium_sp-PT_008978-RA Length: 345

# Fusarium_sp-PT_008978-RA Number of predicted TMHs: 0

# Fusarium_sp-PT_008978-RA Exp number of AAs in TMHs: 6.14514999999999

# Fusarium_sp-PT_008978-RA Exp number, first 60 AAs: 0.00346

# Fusarium_sp-PT_008978-RA Total prob of N-in: 0.03832

Fusarium_sp-PT_008978-RA TMHMM2.0 outside 1 345

# Fusarium_sp-PT_008981-RA Length: 596

# Fusarium_sp-PT_008981-RA Number of predicted TMHs: 0

# Fusarium_sp-PT_008981-RA Exp number of AAs in TMHs: 0.05883

# Fusarium_sp-PT_008981-RA Exp number, first 60 AAs: 4e-05

# Fusarium_sp-PT_008981-RA Total prob of N-in: 0.00022

Fusarium_sp-PT_008981-RA TMHMM2.0 outside 1 596

# Fusarium_sp-PT_008983-RA Length: 388

# Fusarium_sp-PT_008983-RA Number of predicted TMHs: 0

# Fusarium_sp-PT_008983-RA Exp number of AAs in TMHs: 1.1714

# Fusarium_sp-PT_008983-RA Exp number, first 60 AAs: 0.90935

# Fusarium_sp-PT_008983-RA Total prob of N-in: 0.07346

Fusarium_sp-PT_008983-RA TMHMM2.0 outside 1 388

# Fusarium_sp-PT_008984-RA Length: 1511

# Fusarium_sp-PT_008984-RA Number of predicted TMHs: 0

# Fusarium_sp-PT_008984-RA Exp number of AAs in TMHs: 0.00453

# Fusarium_sp-PT_008984-RA Exp number, first 60 AAs: 0.00016

# Fusarium_sp-PT_008984-RA Total prob of N-in: 0.00005

Fusarium_sp-PT_008984-RA TMHMM2.0 outside 1 1511

# Fusarium_sp-PT_008987-RA Length: 122

# Fusarium_sp-PT_008987-RA Number of predicted TMHs: 0

# Fusarium_sp-PT_008987-RA Exp number of AAs in TMHs: 0.02168

# Fusarium_sp-PT_008987-RA Exp number, first 60 AAs: 0

# Fusarium_sp-PT_008987-RA Total prob of N-in: 0.07967

Fusarium_sp-PT_008987-RA TMHMM2.0 outside 1 122

# Fusarium_sp-PT_008988-RA Length: 249

# Fusarium_sp-PT_008988-RA Number of predicted TMHs: 0

# Fusarium_sp-PT_008988-RA Exp number of AAs in TMHs: 6.98206999999999

# Fusarium_sp-PT_008988-RA Exp number, first 60 AAs: 4.88421

# Fusarium_sp-PT_008988-RA Total prob of N-in: 0.23157

Fusarium_sp-PT_008988-RA TMHMM2.0 outside 1 249

# Fusarium_sp-PT_008992-RA Length: 440

# Fusarium_sp-PT_008992-RA Number of predicted TMHs: 1

# Fusarium_sp-PT_008992-RA Exp number of AAs in TMHs: 19.65276

# Fusarium_sp-PT_008992-RA Exp number, first 60 AAs: 19.65252

# Fusarium_sp-PT_008992-RA Total prob of N-in: 0.96178

# Fusarium_sp-PT_008992-RA POSSIBLE N-term signal sequence

Fusarium_sp-PT_008992-RA TMHMM2.0 inside 1 6

Fusarium_sp-PT_008992-RA TMHMM2.0 TMhelix 7 26

Fusarium_sp-PT_008992-RA TMHMM2.0 outside 27 440

# Fusarium_sp-PT_008994-RA Length: 293

# Fusarium_sp-PT_008994-RA Number of predicted TMHs: 0

# Fusarium_sp-PT_008994-RA Exp number of AAs in TMHs: 0.00035

# Fusarium_sp-PT_008994-RA Exp number, first 60 AAs: 0

# Fusarium_sp-PT_008994-RA Total prob of N-in: 0.07090

Fusarium_sp-PT_008994-RA TMHMM2.0 outside 1 293

# Fusarium_sp-PT_008995-RA Length: 969

# Fusarium_sp-PT_008995-RA Number of predicted TMHs: 8

# Fusarium_sp-PT_008995-RA Exp number of AAs in TMHs: 189.60121

# Fusarium_sp-PT_008995-RA Exp number, first 60 AAs: 0.00163

# Fusarium_sp-PT_008995-RA Total prob of N-in: 0.55087

Fusarium_sp-PT_008995-RA TMHMM2.0 outside 1 85

Fusarium_sp-PT_008995-RA TMHMM2.0 TMhelix 86 103

Fusarium_sp-PT_008995-RA TMHMM2.0 inside 104 109

Fusarium_sp-PT_008995-RA TMHMM2.0 TMhelix 110 132

Fusarium_sp-PT_008995-RA TMHMM2.0 outside 133 195

Fusarium_sp-PT_008995-RA TMHMM2.0 TMhelix 196 218

Fusarium_sp-PT_008995-RA TMHMM2.0 inside 219 224

Fusarium_sp-PT_008995-RA TMHMM2.0 TMhelix 225 244

Fusarium_sp-PT_008995-RA TMHMM2.0 outside 245 298

Fusarium_sp-PT_008995-RA TMHMM2.0 TMhelix 299 321

Fusarium_sp-PT_008995-RA TMHMM2.0 inside 322 391

Fusarium_sp-PT_008995-RA TMHMM2.0 TMhelix 392 414

Fusarium_sp-PT_008995-RA TMHMM2.0 outside 415 417

Fusarium_sp-PT_008995-RA TMHMM2.0 TMhelix 418 437

Fusarium_sp-PT_008995-RA TMHMM2.0 inside 438 569

Fusarium_sp-PT_008995-RA TMHMM2.0 TMhelix 570 592

Fusarium_sp-PT_008995-RA TMHMM2.0 outside 593 969

# Fusarium_sp-PT_008996-RA Length: 445

# Fusarium_sp-PT_008996-RA Number of predicted TMHs: 1

# Fusarium_sp-PT_008996-RA Exp number of AAs in TMHs: 23.75973

# Fusarium_sp-PT_008996-RA Exp number, first 60 AAs: 21.10289

# Fusarium_sp-PT_008996-RA Total prob of N-in: 0.92648

# Fusarium_sp-PT_008996-RA POSSIBLE N-term signal sequence

Fusarium_sp-PT_008996-RA TMHMM2.0 inside 1 20

Fusarium_sp-PT_008996-RA TMHMM2.0 TMhelix 21 43

Fusarium_sp-PT_008996-RA TMHMM2.0 outside 44 445

# Fusarium_sp-PT_008997-RA Length: 696

# Fusarium_sp-PT_008997-RA Number of predicted TMHs: 0

# Fusarium_sp-PT_008997-RA Exp number of AAs in TMHs: 4.38089999999999

# Fusarium_sp-PT_008997-RA Exp number, first 60 AAs: 0

# Fusarium_sp-PT_008997-RA Total prob of N-in: 0.00060

Fusarium_sp-PT_008997-RA TMHMM2.0 outside 1 696

# Fusarium_sp-PT_008998-RA Length: 781

# Fusarium_sp-PT_008998-RA Number of predicted TMHs: 0

# Fusarium_sp-PT_008998-RA Exp number of AAs in TMHs: 0.00114

# Fusarium_sp-PT_008998-RA Exp number, first 60 AAs: 0

# Fusarium_sp-PT_008998-RA Total prob of N-in: 0.00018

Fusarium_sp-PT_008998-RA TMHMM2.0 outside 1 781

# Fusarium_sp-PT_008999-RA Length: 1095

# Fusarium_sp-PT_008999-RA Number of predicted TMHs: 0

# Fusarium_sp-PT_008999-RA Exp number of AAs in TMHs: 0.63059

# Fusarium_sp-PT_008999-RA Exp number, first 60 AAs: 0.37265

# Fusarium_sp-PT_008999-RA Total prob of N-in: 0.02420

Fusarium_sp-PT_008999-RA TMHMM2.0 outside 1 1095

# Fusarium_sp-PT_009001-RA Length: 279

# Fusarium_sp-PT_009001-RA Number of predicted TMHs: 1

# Fusarium_sp-PT_009001-RA Exp number of AAs in TMHs: 21.59423

# Fusarium_sp-PT_009001-RA Exp number, first 60 AAs: 0

# Fusarium_sp-PT_009001-RA Total prob of N-in: 0.63851

Fusarium_sp-PT_009001-RA TMHMM2.0 inside 1 127

Fusarium_sp-PT_009001-RA TMHMM2.0 TMhelix 128 150

Fusarium_sp-PT_009001-RA TMHMM2.0 outside 151 279

# Fusarium_sp-PT_009004-RA Length: 482

# Fusarium_sp-PT_009004-RA Number of predicted TMHs: 0

# Fusarium_sp-PT_009004-RA Exp number of AAs in TMHs: 0.00122

# Fusarium_sp-PT_009004-RA Exp number, first 60 AAs: 0.00087

# Fusarium_sp-PT_009004-RA Total prob of N-in: 0.00069

Fusarium_sp-PT_009004-RA TMHMM2.0 outside 1 482

# Fusarium_sp-PT_009007-RA Length: 367

# Fusarium_sp-PT_009007-RA Number of predicted TMHs: 1

# Fusarium_sp-PT_009007-RA Exp number of AAs in TMHs: 22.21841

# Fusarium_sp-PT_009007-RA Exp number, first 60 AAs: 0.0003

# Fusarium_sp-PT_009007-RA Total prob of N-in: 0.06391

Fusarium_sp-PT_009007-RA TMHMM2.0 outside 1 343

Fusarium_sp-PT_009007-RA TMHMM2.0 TMhelix 344 366

Fusarium_sp-PT_009007-RA TMHMM2.0 inside 367 367

# Fusarium_sp-PT_008986-RA Length: 159

# Fusarium_sp-PT_008986-RA Number of predicted TMHs: 0

# Fusarium_sp-PT_008986-RA Exp number of AAs in TMHs: 0

# Fusarium_sp-PT_008986-RA Exp number, first 60 AAs: 0

# Fusarium_sp-PT_008986-RA Total prob of N-in: 0.15229

Fusarium_sp-PT_008986-RA TMHMM2.0 outside 1 159

# Fusarium_sp-PT_008990-RA Length: 580

# Fusarium_sp-PT_008990-RA Number of predicted TMHs: 0

# Fusarium_sp-PT_008990-RA Exp number of AAs in TMHs: 0.01994

# Fusarium_sp-PT_008990-RA Exp number, first 60 AAs: 0

# Fusarium_sp-PT_008990-RA Total prob of N-in: 0.00047

Fusarium_sp-PT_008990-RA TMHMM2.0 outside 1 580

# Fusarium_sp-PT_008991-RA Length: 749

# Fusarium_sp-PT_008991-RA Number of predicted TMHs: 0

# Fusarium_sp-PT_008991-RA Exp number of AAs in TMHs: 0.56598

# Fusarium_sp-PT_008991-RA Exp number, first 60 AAs: 0.56525

# Fusarium_sp-PT_008991-RA Total prob of N-in: 0.02803

Fusarium_sp-PT_008991-RA TMHMM2.0 outside 1 749

# Fusarium_sp-PT_008993-RA Length: 114

# Fusarium_sp-PT_008993-RA Number of predicted TMHs: 0

# Fusarium_sp-PT_008993-RA Exp number of AAs in TMHs: 5.9119

# Fusarium_sp-PT_008993-RA Exp number, first 60 AAs: 0

# Fusarium_sp-PT_008993-RA Total prob of N-in: 0.17860

Fusarium_sp-PT_008993-RA TMHMM2.0 outside 1 114

# Fusarium_sp-PT_009002-RA Length: 162

# Fusarium_sp-PT_009002-RA Number of predicted TMHs: 0

# Fusarium_sp-PT_009002-RA Exp number of AAs in TMHs: 0.47606

# Fusarium_sp-PT_009002-RA Exp number, first 60 AAs: 0

# Fusarium_sp-PT_009002-RA Total prob of N-in: 0.43089

Fusarium_sp-PT_009002-RA TMHMM2.0 outside 1 162

# Fusarium_sp-PT_009003-RA Length: 377

# Fusarium_sp-PT_009003-RA Number of predicted TMHs: 0

# Fusarium_sp-PT_009003-RA Exp number of AAs in TMHs: 0.1968

# Fusarium_sp-PT_009003-RA Exp number, first 60 AAs: 0.00921

# Fusarium_sp-PT_009003-RA Total prob of N-in: 0.01946

Fusarium_sp-PT_009003-RA TMHMM2.0 outside 1 377

# Fusarium_sp-PT_009005-RA Length: 331

# Fusarium_sp-PT_009005-RA Number of predicted TMHs: 2

# Fusarium_sp-PT_009005-RA Exp number of AAs in TMHs: 45.89933

# Fusarium_sp-PT_009005-RA Exp number, first 60 AAs: 1.46225

# Fusarium_sp-PT_009005-RA Total prob of N-in: 0.92375

Fusarium_sp-PT_009005-RA TMHMM2.0 inside 1 130

Fusarium_sp-PT_009005-RA TMHMM2.0 TMhelix 131 153

Fusarium_sp-PT_009005-RA TMHMM2.0 outside 154 162

Fusarium_sp-PT_009005-RA TMHMM2.0 TMhelix 163 185

Fusarium_sp-PT_009005-RA TMHMM2.0 inside 186 331

# Fusarium_sp-PT_009006-RA Length: 1015

# Fusarium_sp-PT_009006-RA Number of predicted TMHs: 7

# Fusarium_sp-PT_009006-RA Exp number of AAs in TMHs: 158.64392

# Fusarium_sp-PT_009006-RA Exp number, first 60 AAs: 0.00038

# Fusarium_sp-PT_009006-RA Total prob of N-in: 0.16099

Fusarium_sp-PT_009006-RA TMHMM2.0 outside 1 285

Fusarium_sp-PT_009006-RA TMHMM2.0 TMhelix 286 308

Fusarium_sp-PT_009006-RA TMHMM2.0 inside 309 757

Fusarium_sp-PT_009006-RA TMHMM2.0 TMhelix 758 777

Fusarium_sp-PT_009006-RA TMHMM2.0 outside 778 805

Fusarium_sp-PT_009006-RA TMHMM2.0 TMhelix 806 828

Fusarium_sp-PT_009006-RA TMHMM2.0 inside 829 847

Fusarium_sp-PT_009006-RA TMHMM2.0 TMhelix 848 870

Fusarium_sp-PT_009006-RA TMHMM2.0 outside 871 884

Fusarium_sp-PT_009006-RA TMHMM2.0 TMhelix 885 907

Fusarium_sp-PT_009006-RA TMHMM2.0 inside 908 913

Fusarium_sp-PT_009006-RA TMHMM2.0 TMhelix 914 936

Fusarium_sp-PT_009006-RA TMHMM2.0 outside 937 990

Fusarium_sp-PT_009006-RA TMHMM2.0 TMhelix 991 1013

Fusarium_sp-PT_009006-RA TMHMM2.0 inside 1014 1015

# Fusarium_sp-PT_009009-RA Length: 1618

# Fusarium_sp-PT_009009-RA Number of predicted TMHs: 0

# Fusarium_sp-PT_009009-RA Exp number of AAs in TMHs: 0.00022

# Fusarium_sp-PT_009009-RA Exp number, first 60 AAs: 0

# Fusarium_sp-PT_009009-RA Total prob of N-in: 0.00000

Fusarium_sp-PT_009009-RA TMHMM2.0 outside 1 1618

# Fusarium_sp-PT_009010-RA Length: 894

# Fusarium_sp-PT_009010-RA Number of predicted TMHs: 5

# Fusarium_sp-PT_009010-RA Exp number of AAs in TMHs: 110.13095

# Fusarium_sp-PT_009010-RA Exp number, first 60 AAs: 43.94836

# Fusarium_sp-PT_009010-RA Total prob of N-in: 0.99884

# Fusarium_sp-PT_009010-RA POSSIBLE N-term signal sequence

Fusarium_sp-PT_009010-RA TMHMM2.0 inside 1 6

Fusarium_sp-PT_009010-RA TMHMM2.0 TMhelix 7 26

Fusarium_sp-PT_009010-RA TMHMM2.0 outside 27 35

Fusarium_sp-PT_009010-RA TMHMM2.0 TMhelix 36 58

Fusarium_sp-PT_009010-RA TMHMM2.0 inside 59 62

Fusarium_sp-PT_009010-RA TMHMM2.0 TMhelix 63 85

Fusarium_sp-PT_009010-RA TMHMM2.0 outside 86 113

Fusarium_sp-PT_009010-RA TMHMM2.0 TMhelix 114 136

Fusarium_sp-PT_009010-RA TMHMM2.0 inside 137 217

Fusarium_sp-PT_009010-RA TMHMM2.0 TMhelix 218 240

Fusarium_sp-PT_009010-RA TMHMM2.0 outside 241 894

# Fusarium_sp-PT_008989-RA Length: 159

# Fusarium_sp-PT_008989-RA Number of predicted TMHs: 3

# Fusarium_sp-PT_008989-RA Exp number of AAs in TMHs: 61.61292

# Fusarium_sp-PT_008989-RA Exp number, first 60 AAs: 35.70901

# Fusarium_sp-PT_008989-RA Total prob of N-in: 0.03329

# Fusarium_sp-PT_008989-RA POSSIBLE N-term signal sequence

Fusarium_sp-PT_008989-RA TMHMM2.0 outside 1 3

Fusarium_sp-PT_008989-RA TMHMM2.0 TMhelix 4 26

Fusarium_sp-PT_008989-RA TMHMM2.0 inside 27 46

Fusarium_sp-PT_008989-RA TMHMM2.0 TMhelix 47 66

Fusarium_sp-PT_008989-RA TMHMM2.0 outside 67 80

Fusarium_sp-PT_008989-RA TMHMM2.0 TMhelix 81 98

Fusarium_sp-PT_008989-RA TMHMM2.0 inside 99 159

# Fusarium_sp-PT_009008-RA Length: 52

# Fusarium_sp-PT_009008-RA Number of predicted TMHs: 0

# Fusarium_sp-PT_009008-RA Exp number of AAs in TMHs: 0.00033

# Fusarium_sp-PT_009008-RA Exp number, first 60 AAs: 0.00033

# Fusarium_sp-PT_009008-RA Total prob of N-in: 0.62588

Fusarium_sp-PT_009008-RA TMHMM2.0 inside 1 52

# Fusarium_sp-PT_009011-RA Length: 1041

# Fusarium_sp-PT_009011-RA Number of predicted TMHs: 0

# Fusarium_sp-PT_009011-RA Exp number of AAs in TMHs: 0.06633

# Fusarium_sp-PT_009011-RA Exp number, first 60 AAs: 0.04729

# Fusarium_sp-PT_009011-RA Total prob of N-in: 0.00271

Fusarium_sp-PT_009011-RA TMHMM2.0 outside 1 1041

# Fusarium_sp-PT_009013-RA Length: 822

# Fusarium_sp-PT_009013-RA Number of predicted TMHs: 0

# Fusarium_sp-PT_009013-RA Exp number of AAs in TMHs: 0.000910000000000001

# Fusarium_sp-PT_009013-RA Exp number, first 60 AAs: 0

# Fusarium_sp-PT_009013-RA Total prob of N-in: 0.00001

Fusarium_sp-PT_009013-RA TMHMM2.0 outside 1 822

# Fusarium_sp-PT_009015-RA Length: 516

# Fusarium_sp-PT_009015-RA Number of predicted TMHs: 0

# Fusarium_sp-PT_009015-RA Exp number of AAs in TMHs: 0.6967

# Fusarium_sp-PT_009015-RA Exp number, first 60 AAs: 0.03393

# Fusarium_sp-PT_009015-RA Total prob of N-in: 0.00320

Fusarium_sp-PT_009015-RA TMHMM2.0 outside 1 516

# Fusarium_sp-PT_009018-RA Length: 151

# Fusarium_sp-PT_009018-RA Number of predicted TMHs: 0

# Fusarium_sp-PT_009018-RA Exp number of AAs in TMHs: 0.03924

# Fusarium_sp-PT_009018-RA Exp number, first 60 AAs: 0.03777

# Fusarium_sp-PT_009018-RA Total prob of N-in: 0.41567

Fusarium_sp-PT_009018-RA TMHMM2.0 outside 1 151

# Fusarium_sp-PT_009019-RA Length: 933

# Fusarium_sp-PT_009019-RA Number of predicted TMHs: 0

# Fusarium_sp-PT_009019-RA Exp number of AAs in TMHs: 0.03

# Fusarium_sp-PT_009019-RA Exp number, first 60 AAs: 0

# Fusarium_sp-PT_009019-RA Total prob of N-in: 0.00157

Fusarium_sp-PT_009019-RA TMHMM2.0 outside 1 933

# Fusarium_sp-PT_009021-RA Length: 375

# Fusarium_sp-PT_009021-RA Number of predicted TMHs: 0

# Fusarium_sp-PT_009021-RA Exp number of AAs in TMHs: 0.00588

# Fusarium_sp-PT_009021-RA Exp number, first 60 AAs: 0

# Fusarium_sp-PT_009021-RA Total prob of N-in: 0.00313

Fusarium_sp-PT_009021-RA TMHMM2.0 outside 1 375

# Fusarium_sp-PT_009022-RA Length: 320

# Fusarium_sp-PT_009022-RA Number of predicted TMHs: 0

# Fusarium_sp-PT_009022-RA Exp number of AAs in TMHs: 0.00123

# Fusarium_sp-PT_009022-RA Exp number, first 60 AAs: 0.00021

# Fusarium_sp-PT_009022-RA Total prob of N-in: 0.09052

Fusarium_sp-PT_009022-RA TMHMM2.0 outside 1 320

# Fusarium_sp-PT_009024-RA Length: 143

# Fusarium_sp-PT_009024-RA Number of predicted TMHs: 0

# Fusarium_sp-PT_009024-RA Exp number of AAs in TMHs: 0.06576

# Fusarium_sp-PT_009024-RA Exp number, first 60 AAs: 0.00045

# Fusarium_sp-PT_009024-RA Total prob of N-in: 0.07711

Fusarium_sp-PT_009024-RA TMHMM2.0 outside 1 143

# Fusarium_sp-PT_009032-RA Length: 508

# Fusarium_sp-PT_009032-RA Number of predicted TMHs: 0

# Fusarium_sp-PT_009032-RA Exp number of AAs in TMHs: 0.00219

# Fusarium_sp-PT_009032-RA Exp number, first 60 AAs: 0

# Fusarium_sp-PT_009032-RA Total prob of N-in: 0.00054

Fusarium_sp-PT_009032-RA TMHMM2.0 outside 1 508

# Fusarium_sp-PT_009034-RA Length: 269

# Fusarium_sp-PT_009034-RA Number of predicted TMHs: 1

# Fusarium_sp-PT_009034-RA Exp number of AAs in TMHs: 22.13411

# Fusarium_sp-PT_009034-RA Exp number, first 60 AAs: 22.12294

# Fusarium_sp-PT_009034-RA Total prob of N-in: 0.27853

# Fusarium_sp-PT_009034-RA POSSIBLE N-term signal sequence

Fusarium_sp-PT_009034-RA TMHMM2.0 outside 1 24

Fusarium_sp-PT_009034-RA TMHMM2.0 TMhelix 25 47

Fusarium_sp-PT_009034-RA TMHMM2.0 inside 48 269

# Fusarium_sp-PT_009036-RA Length: 1271

# Fusarium_sp-PT_009036-RA Number of predicted TMHs: 0

# Fusarium_sp-PT_009036-RA Exp number of AAs in TMHs: 0

# Fusarium_sp-PT_009036-RA Exp number, first 60 AAs: 0

# Fusarium_sp-PT_009036-RA Total prob of N-in: 0.00000

Fusarium_sp-PT_009036-RA TMHMM2.0 outside 1 1271

# Fusarium_sp-PT_009037-RA Length: 286

# Fusarium_sp-PT_009037-RA Number of predicted TMHs: 1

# Fusarium_sp-PT_009037-RA Exp number of AAs in TMHs: 22.42445

# Fusarium_sp-PT_009037-RA Exp number, first 60 AAs: 0

# Fusarium_sp-PT_009037-RA Total prob of N-in: 0.02401

Fusarium_sp-PT_009037-RA TMHMM2.0 outside 1 181

Fusarium_sp-PT_009037-RA TMHMM2.0 TMhelix 182 204

Fusarium_sp-PT_009037-RA TMHMM2.0 inside 205 286

# Fusarium_sp-PT_009038-RA Length: 667

# Fusarium_sp-PT_009038-RA Number of predicted TMHs: 0

# Fusarium_sp-PT_009038-RA Exp number of AAs in TMHs: 0.02256

# Fusarium_sp-PT_009038-RA Exp number, first 60 AAs: 0.00184

# Fusarium_sp-PT_009038-RA Total prob of N-in: 0.00088

Fusarium_sp-PT_009038-RA TMHMM2.0 outside 1 667

# Fusarium_sp-PT_009040-RA Length: 428

# Fusarium_sp-PT_009040-RA Number of predicted TMHs: 0

# Fusarium_sp-PT_009040-RA Exp number of AAs in TMHs: 0.00827999999999998

# Fusarium_sp-PT_009040-RA Exp number, first 60 AAs: 0

# Fusarium_sp-PT_009040-RA Total prob of N-in: 0.01536

Fusarium_sp-PT_009040-RA TMHMM2.0 outside 1 428

# Fusarium_sp-PT_009041-RA Length: 266

# Fusarium_sp-PT_009041-RA Number of predicted TMHs: 0

# Fusarium_sp-PT_009041-RA Exp number of AAs in TMHs: 0.12362

# Fusarium_sp-PT_009041-RA Exp number, first 60 AAs: 0.00832

# Fusarium_sp-PT_009041-RA Total prob of N-in: 0.00688

Fusarium_sp-PT_009041-RA TMHMM2.0 outside 1 266

# Fusarium_sp-PT_009042-RA Length: 320

# Fusarium_sp-PT_009042-RA Number of predicted TMHs: 0

# Fusarium_sp-PT_009042-RA Exp number of AAs in TMHs: 0.0368900000000001

# Fusarium_sp-PT_009042-RA Exp number, first 60 AAs: 0.00952

# Fusarium_sp-PT_009042-RA Total prob of N-in: 0.01268

Fusarium_sp-PT_009042-RA TMHMM2.0 outside 1 320

# Fusarium_sp-PT_009012-RA Length: 131

# Fusarium_sp-PT_009012-RA Number of predicted TMHs: 4

# Fusarium_sp-PT_009012-RA Exp number of AAs in TMHs: 83.35678

# Fusarium_sp-PT_009012-RA Exp number, first 60 AAs: 42.09267

# Fusarium_sp-PT_009012-RA Total prob of N-in: 0.47099

# Fusarium_sp-PT_009012-RA POSSIBLE N-term signal sequence

Fusarium_sp-PT_009012-RA TMHMM2.0 inside 1 6

Fusarium_sp-PT_009012-RA TMHMM2.0 TMhelix 7 29

Fusarium_sp-PT_009012-RA TMHMM2.0 outside 30 32

Fusarium_sp-PT_009012-RA TMHMM2.0 TMhelix 33 52

Fusarium_sp-PT_009012-RA TMHMM2.0 inside 53 63

Fusarium_sp-PT_009012-RA TMHMM2.0 TMhelix 64 83

Fusarium_sp-PT_009012-RA TMHMM2.0 outside 84 86

Fusarium_sp-PT_009012-RA TMHMM2.0 TMhelix 87 109

Fusarium_sp-PT_009012-RA TMHMM2.0 inside 110 131

# Fusarium_sp-PT_009014-RA Length: 241

# Fusarium_sp-PT_009014-RA Number of predicted TMHs: 0

# Fusarium_sp-PT_009014-RA Exp number of AAs in TMHs: 0.38062

# Fusarium_sp-PT_009014-RA Exp number, first 60 AAs: 0.31133

# Fusarium_sp-PT_009014-RA Total prob of N-in: 0.10378

Fusarium_sp-PT_009014-RA TMHMM2.0 outside 1 241

# Fusarium_sp-PT_009016-RA Length: 449

# Fusarium_sp-PT_009016-RA Number of predicted TMHs: 0

# Fusarium_sp-PT_009016-RA Exp number of AAs in TMHs: 0.0443

# Fusarium_sp-PT_009016-RA Exp number, first 60 AAs: 0

# Fusarium_sp-PT_009016-RA Total prob of N-in: 0.00138

Fusarium_sp-PT_009016-RA TMHMM2.0 outside 1 449

# Fusarium_sp-PT_009017-RA Length: 430

# Fusarium_sp-PT_009017-RA Number of predicted TMHs: 0

# Fusarium_sp-PT_009017-RA Exp number of AAs in TMHs: 0.24107

# Fusarium_sp-PT_009017-RA Exp number, first 60 AAs: 0.00818

# Fusarium_sp-PT_009017-RA Total prob of N-in: 0.00683

Fusarium_sp-PT_009017-RA TMHMM2.0 outside 1 430

# Fusarium_sp-PT_009020-RA Length: 361

# Fusarium_sp-PT_009020-RA Number of predicted TMHs: 6

# Fusarium_sp-PT_009020-RA Exp number of AAs in TMHs: 135.04845

# Fusarium_sp-PT_009020-RA Exp number, first 60 AAs: 21.70148

# Fusarium_sp-PT_009020-RA Total prob of N-in: 0.00866

# Fusarium_sp-PT_009020-RA POSSIBLE N-term signal sequence

Fusarium_sp-PT_009020-RA TMHMM2.0 outside 1 29

Fusarium_sp-PT_009020-RA TMHMM2.0 TMhelix 30 52

Fusarium_sp-PT_009020-RA TMHMM2.0 inside 53 124

Fusarium_sp-PT_009020-RA TMHMM2.0 TMhelix 125 147

Fusarium_sp-PT_009020-RA TMHMM2.0 outside 148 150

Fusarium_sp-PT_009020-RA TMHMM2.0 TMhelix 151 173

Fusarium_sp-PT_009020-RA TMHMM2.0 inside 174 200

Fusarium_sp-PT_009020-RA TMHMM2.0 TMhelix 201 223

Fusarium_sp-PT_009020-RA TMHMM2.0 outside 224 237

Fusarium_sp-PT_009020-RA TMHMM2.0 TMhelix 238 260

Fusarium_sp-PT_009020-RA TMHMM2.0 inside 261 320

Fusarium_sp-PT_009020-RA TMHMM2.0 TMhelix 321 343

Fusarium_sp-PT_009020-RA TMHMM2.0 outside 344 361

# Fusarium_sp-PT_009023-RA Length: 1153

# Fusarium_sp-PT_009023-RA Number of predicted TMHs: 9

# Fusarium_sp-PT_009023-RA Exp number of AAs in TMHs: 195.38887

# Fusarium_sp-PT_009023-RA Exp number, first 60 AAs: 12.81293

# Fusarium_sp-PT_009023-RA Total prob of N-in: 0.97449

# Fusarium_sp-PT_009023-RA POSSIBLE N-term signal sequence

Fusarium_sp-PT_009023-RA TMHMM2.0 inside 1 77

Fusarium_sp-PT_009023-RA TMHMM2.0 TMhelix 78 100

Fusarium_sp-PT_009023-RA TMHMM2.0 outside 101 109

Fusarium_sp-PT_009023-RA TMHMM2.0 TMhelix 110 128

Fusarium_sp-PT_009023-RA TMHMM2.0 inside 129 134

Fusarium_sp-PT_009023-RA TMHMM2.0 TMhelix 135 152

Fusarium_sp-PT_009023-RA TMHMM2.0 outside 153 161

Fusarium_sp-PT_009023-RA TMHMM2.0 TMhelix 162 184

Fusarium_sp-PT_009023-RA TMHMM2.0 inside 185 649

Fusarium_sp-PT_009023-RA TMHMM2.0 TMhelix 650 668

Fusarium_sp-PT_009023-RA TMHMM2.0 outside 669 682

Fusarium_sp-PT_009023-RA TMHMM2.0 TMhelix 683 705

Fusarium_sp-PT_009023-RA TMHMM2.0 inside 706 717

Fusarium_sp-PT_009023-RA TMHMM2.0 TMhelix 718 740

Fusarium_sp-PT_009023-RA TMHMM2.0 outside 741 754

Fusarium_sp-PT_009023-RA TMHMM2.0 TMhelix 755 777

Fusarium_sp-PT_009023-RA TMHMM2.0 inside 778 783

Fusarium_sp-PT_009023-RA TMHMM2.0 TMhelix 784 806

Fusarium_sp-PT_009023-RA TMHMM2.0 outside 807 1153

# Fusarium_sp-PT_009025-RA Length: 298

# Fusarium_sp-PT_009025-RA Number of predicted TMHs: 0

# Fusarium_sp-PT_009025-RA Exp number of AAs in TMHs: 0.00014

# Fusarium_sp-PT_009025-RA Exp number, first 60 AAs: 0.00014

# Fusarium_sp-PT_009025-RA Total prob of N-in: 0.03140

Fusarium_sp-PT_009025-RA TMHMM2.0 outside 1 298

# Fusarium_sp-PT_009026-RA Length: 1012

# Fusarium_sp-PT_009026-RA Number of predicted TMHs: 0

# Fusarium_sp-PT_009026-RA Exp number of AAs in TMHs: 0.00159

# Fusarium_sp-PT_009026-RA Exp number, first 60 AAs: 0

# Fusarium_sp-PT_009026-RA Total prob of N-in: 0.00001

Fusarium_sp-PT_009026-RA TMHMM2.0 outside 1 1012

# Fusarium_sp-PT_009027-RA Length: 152

# Fusarium_sp-PT_009027-RA Number of predicted TMHs: 0

# Fusarium_sp-PT_009027-RA Exp number of AAs in TMHs: 0

# Fusarium_sp-PT_009027-RA Exp number, first 60 AAs: 0

# Fusarium_sp-PT_009027-RA Total prob of N-in: 0.12725

Fusarium_sp-PT_009027-RA TMHMM2.0 outside 1 152

# Fusarium_sp-PT_009028-RA Length: 815

# Fusarium_sp-PT_009028-RA Number of predicted TMHs: 0

# Fusarium_sp-PT_009028-RA Exp number of AAs in TMHs: 0.00446

# Fusarium_sp-PT_009028-RA Exp number, first 60 AAs: 0

# Fusarium_sp-PT_009028-RA Total prob of N-in: 0.00047

Fusarium_sp-PT_009028-RA TMHMM2.0 outside 1 815

# Fusarium_sp-PT_009029-RA Length: 189

# Fusarium_sp-PT_009029-RA Number of predicted TMHs: 1

# Fusarium_sp-PT_009029-RA Exp number of AAs in TMHs: 21.41619

# Fusarium_sp-PT_009029-RA Exp number, first 60 AAs: 21.41619

# Fusarium_sp-PT_009029-RA Total prob of N-in: 0.38555

# Fusarium_sp-PT_009029-RA POSSIBLE N-term signal sequence

Fusarium_sp-PT_009029-RA TMHMM2.0 outside 1 4

Fusarium_sp-PT_009029-RA TMHMM2.0 TMhelix 5 27

Fusarium_sp-PT_009029-RA TMHMM2.0 inside 28 189

# Fusarium_sp-PT_009030-RA Length: 463

# Fusarium_sp-PT_009030-RA Number of predicted TMHs: 0

# Fusarium_sp-PT_009030-RA Exp number of AAs in TMHs: 0.00355

# Fusarium_sp-PT_009030-RA Exp number, first 60 AAs: 0.00333

# Fusarium_sp-PT_009030-RA Total prob of N-in: 0.00093

Fusarium_sp-PT_009030-RA TMHMM2.0 outside 1 463

# Fusarium_sp-PT_009031-RA Length: 365

# Fusarium_sp-PT_009031-RA Number of predicted TMHs: 0

# Fusarium_sp-PT_009031-RA Exp number of AAs in TMHs: 2.38181

# Fusarium_sp-PT_009031-RA Exp number, first 60 AAs: 2.34377

# Fusarium_sp-PT_009031-RA Total prob of N-in: 0.00819

Fusarium_sp-PT_009031-RA TMHMM2.0 outside 1 365

# Fusarium_sp-PT_009033-RA Length: 681

# Fusarium_sp-PT_009033-RA Number of predicted TMHs: 0

# Fusarium_sp-PT_009033-RA Exp number of AAs in TMHs: 2.25949

# Fusarium_sp-PT_009033-RA Exp number, first 60 AAs: 0.00213

# Fusarium_sp-PT_009033-RA Total prob of N-in: 0.00034

Fusarium_sp-PT_009033-RA TMHMM2.0 outside 1 681

# Fusarium_sp-PT_009035-RA Length: 1180

# Fusarium_sp-PT_009035-RA Number of predicted TMHs: 0

# Fusarium_sp-PT_009035-RA Exp number of AAs in TMHs: 0.0434200000000002

# Fusarium_sp-PT_009035-RA Exp number, first 60 AAs: 0.03128

# Fusarium_sp-PT_009035-RA Total prob of N-in: 0.00167

Fusarium_sp-PT_009035-RA TMHMM2.0 outside 1 1180

# Fusarium_sp-PT_009039-RA Length: 348

# Fusarium_sp-PT_009039-RA Number of predicted TMHs: 0

# Fusarium_sp-PT_009039-RA Exp number of AAs in TMHs: 0.83873

# Fusarium_sp-PT_009039-RA Exp number, first 60 AAs: 0.49905

# Fusarium_sp-PT_009039-RA Total prob of N-in: 0.04612

Fusarium_sp-PT_009039-RA TMHMM2.0 outside 1 348

# Fusarium_sp-PT_009044-RA Length: 320

# Fusarium_sp-PT_009044-RA Number of predicted TMHs: 0

# Fusarium_sp-PT_009044-RA Exp number of AAs in TMHs: 0.0198

# Fusarium_sp-PT_009044-RA Exp number, first 60 AAs: 0.01234

# Fusarium_sp-PT_009044-RA Total prob of N-in: 0.01581

Fusarium_sp-PT_009044-RA TMHMM2.0 outside 1 320

# Fusarium_sp-PT_009045-RA Length: 218

# Fusarium_sp-PT_009045-RA Number of predicted TMHs: 0

# Fusarium_sp-PT_009045-RA Exp number of AAs in TMHs: 0.00018

# Fusarium_sp-PT_009045-RA Exp number, first 60 AAs: 0

# Fusarium_sp-PT_009045-RA Total prob of N-in: 0.43468

Fusarium_sp-PT_009045-RA TMHMM2.0 outside 1 218

# Fusarium_sp-PT_009043-RA Length: 311

# Fusarium_sp-PT_009043-RA Number of predicted TMHs: 0

# Fusarium_sp-PT_009043-RA Exp number of AAs in TMHs: 0.00014

# Fusarium_sp-PT_009043-RA Exp number, first 60 AAs: 0

# Fusarium_sp-PT_009043-RA Total prob of N-in: 0.03277

Fusarium_sp-PT_009043-RA TMHMM2.0 outside 1 311

# Fusarium_sp-PT_009047-RA Length: 567

# Fusarium_sp-PT_009047-RA Number of predicted TMHs: 12

# Fusarium_sp-PT_009047-RA Exp number of AAs in TMHs: 249.90575

# Fusarium_sp-PT_009047-RA Exp number, first 60 AAs: 22.0338

# Fusarium_sp-PT_009047-RA Total prob of N-in: 0.99555

# Fusarium_sp-PT_009047-RA POSSIBLE N-term signal sequence

Fusarium_sp-PT_009047-RA TMHMM2.0 inside 1 28

Fusarium_sp-PT_009047-RA TMHMM2.0 TMhelix 29 51

Fusarium_sp-PT_009047-RA TMHMM2.0 outside 52 65

Fusarium_sp-PT_009047-RA TMHMM2.0 TMhelix 66 88

Fusarium_sp-PT_009047-RA TMHMM2.0 inside 89 99

Fusarium_sp-PT_009047-RA TMHMM2.0 TMhelix 100 119

Fusarium_sp-PT_009047-RA TMHMM2.0 outside 120 128

Fusarium_sp-PT_009047-RA TMHMM2.0 TMhelix 129 151

Fusarium_sp-PT_009047-RA TMHMM2.0 inside 152 157

Fusarium_sp-PT_009047-RA TMHMM2.0 TMhelix 158 180

Fusarium_sp-PT_009047-RA TMHMM2.0 outside 181 201

Fusarium_sp-PT_009047-RA TMHMM2.0 TMhelix 202 224

Fusarium_sp-PT_009047-RA TMHMM2.0 inside 225 333

Fusarium_sp-PT_009047-RA TMHMM2.0 TMhelix 334 351

Fusarium_sp-PT_009047-RA TMHMM2.0 outside 352 385

Fusarium_sp-PT_009047-RA TMHMM2.0 TMhelix 386 408

Fusarium_sp-PT_009047-RA TMHMM2.0 inside 409 414

Fusarium_sp-PT_009047-RA TMHMM2.0 TMhelix 415 437

Fusarium_sp-PT_009047-RA TMHMM2.0 outside 438 446

Fusarium_sp-PT_009047-RA TMHMM2.0 TMhelix 447 469

Fusarium_sp-PT_009047-RA TMHMM2.0 inside 470 477

Fusarium_sp-PT_009047-RA TMHMM2.0 TMhelix 478 500

Fusarium_sp-PT_009047-RA TMHMM2.0 outside 501 514

Fusarium_sp-PT_009047-RA TMHMM2.0 TMhelix 515 532

Fusarium_sp-PT_009047-RA TMHMM2.0 inside 533 567

# Fusarium_sp-PT_009048-RA Length: 960

# Fusarium_sp-PT_009048-RA Number of predicted TMHs: 0

# Fusarium_sp-PT_009048-RA Exp number of AAs in TMHs: 1.73571

# Fusarium_sp-PT_009048-RA Exp number, first 60 AAs: 0

# Fusarium_sp-PT_009048-RA Total prob of N-in: 0.00100

Fusarium_sp-PT_009048-RA TMHMM2.0 outside 1 960

# Fusarium_sp-PT_009051-RA Length: 316

# Fusarium_sp-PT_009051-RA Number of predicted TMHs: 0

# Fusarium_sp-PT_009051-RA Exp number of AAs in TMHs: 0.00034

# Fusarium_sp-PT_009051-RA Exp number, first 60 AAs: 0.00034

# Fusarium_sp-PT_009051-RA Total prob of N-in: 0.00966

Fusarium_sp-PT_009051-RA TMHMM2.0 outside 1 316

# Fusarium_sp-PT_009052-RA Length: 414

# Fusarium_sp-PT_009052-RA Number of predicted TMHs: 0

# Fusarium_sp-PT_009052-RA Exp number of AAs in TMHs: 0.13605

# Fusarium_sp-PT_009052-RA Exp number, first 60 AAs: 0.1092

# Fusarium_sp-PT_009052-RA Total prob of N-in: 0.00754

Fusarium_sp-PT_009052-RA TMHMM2.0 outside 1 414

# Fusarium_sp-PT_009054-RA Length: 676

# Fusarium_sp-PT_009054-RA Number of predicted TMHs: 0

# Fusarium_sp-PT_009054-RA Exp number of AAs in TMHs: 0.03413

# Fusarium_sp-PT_009054-RA Exp number, first 60 AAs: 0.00168

# Fusarium_sp-PT_009054-RA Total prob of N-in: 0.00026

Fusarium_sp-PT_009054-RA TMHMM2.0 outside 1 676

# Fusarium_sp-PT_009057-RA Length: 1123

# Fusarium_sp-PT_009057-RA Number of predicted TMHs: 0

# Fusarium_sp-PT_009057-RA Exp number of AAs in TMHs: 5.55502

# Fusarium_sp-PT_009057-RA Exp number, first 60 AAs: 0

# Fusarium_sp-PT_009057-RA Total prob of N-in: 0.00003

Fusarium_sp-PT_009057-RA TMHMM2.0 outside 1 1123

# Fusarium_sp-PT_009059-RA Length: 236

# Fusarium_sp-PT_009059-RA Number of predicted TMHs: 0

# Fusarium_sp-PT_009059-RA Exp number of AAs in TMHs: 0.0528900000000001

# Fusarium_sp-PT_009059-RA Exp number, first 60 AAs: 0.05274

# Fusarium_sp-PT_009059-RA Total prob of N-in: 0.02785

Fusarium_sp-PT_009059-RA TMHMM2.0 outside 1 236

# Fusarium_sp-PT_009061-RA Length: 486

# Fusarium_sp-PT_009061-RA Number of predicted TMHs: 0

# Fusarium_sp-PT_009061-RA Exp number of AAs in TMHs: 0.08899

# Fusarium_sp-PT_009061-RA Exp number, first 60 AAs: 0

# Fusarium_sp-PT_009061-RA Total prob of N-in: 0.00118

Fusarium_sp-PT_009061-RA TMHMM2.0 outside 1 486

# Fusarium_sp-PT_009063-RA Length: 307

# Fusarium_sp-PT_009063-RA Number of predicted TMHs: 0

# Fusarium_sp-PT_009063-RA Exp number of AAs in TMHs: 0.01548

# Fusarium_sp-PT_009063-RA Exp number, first 60 AAs: 0.00018

# Fusarium_sp-PT_009063-RA Total prob of N-in: 0.06577

Fusarium_sp-PT_009063-RA TMHMM2.0 outside 1 307

# Fusarium_sp-PT_009065-RA Length: 656

# Fusarium_sp-PT_009065-RA Number of predicted TMHs: 0

# Fusarium_sp-PT_009065-RA Exp number of AAs in TMHs: 0.00479999999999999

# Fusarium_sp-PT_009065-RA Exp number, first 60 AAs: 0

# Fusarium_sp-PT_009065-RA Total prob of N-in: 0.00210

Fusarium_sp-PT_009065-RA TMHMM2.0 outside 1 656

# Fusarium_sp-PT_009069-RA Length: 941

# Fusarium_sp-PT_009069-RA Number of predicted TMHs: 0

# Fusarium_sp-PT_009069-RA Exp number of AAs in TMHs: 2.40339

# Fusarium_sp-PT_009069-RA Exp number, first 60 AAs: 0

# Fusarium_sp-PT_009069-RA Total prob of N-in: 0.00048

Fusarium_sp-PT_009069-RA TMHMM2.0 outside 1 941

# Fusarium_sp-PT_009072-RA Length: 1069

# Fusarium_sp-PT_009072-RA Number of predicted TMHs: 0

# Fusarium_sp-PT_009072-RA Exp number of AAs in TMHs: 0.60406

# Fusarium_sp-PT_009072-RA Exp number, first 60 AAs: 0.0002

# Fusarium_sp-PT_009072-RA Total prob of N-in: 0.02536

Fusarium_sp-PT_009072-RA TMHMM2.0 outside 1 1069

# Fusarium_sp-PT_009073-RA Length: 776

# Fusarium_sp-PT_009073-RA Number of predicted TMHs: 0

# Fusarium_sp-PT_009073-RA Exp number of AAs in TMHs: 0.0489900000000002

# Fusarium_sp-PT_009073-RA Exp number, first 60 AAs: 0.00205

# Fusarium_sp-PT_009073-RA Total prob of N-in: 0.00231

Fusarium_sp-PT_009073-RA TMHMM2.0 outside 1 776

# Fusarium_sp-PT_009049-RA Length: 235

# Fusarium_sp-PT_009049-RA Number of predicted TMHs: 1

# Fusarium_sp-PT_009049-RA Exp number of AAs in TMHs: 23.93435

# Fusarium_sp-PT_009049-RA Exp number, first 60 AAs: 15.78845

# Fusarium_sp-PT_009049-RA Total prob of N-in: 0.10769

# Fusarium_sp-PT_009049-RA POSSIBLE N-term signal sequence

Fusarium_sp-PT_009049-RA TMHMM2.0 outside 1 45

Fusarium_sp-PT_009049-RA TMHMM2.0 TMhelix 46 68

Fusarium_sp-PT_009049-RA TMHMM2.0 inside 69 235

# Fusarium_sp-PT_009050-RA Length: 836

# Fusarium_sp-PT_009050-RA Number of predicted TMHs: 0

# Fusarium_sp-PT_009050-RA Exp number of AAs in TMHs: 0.0824

# Fusarium_sp-PT_009050-RA Exp number, first 60 AAs: 0.00018

# Fusarium_sp-PT_009050-RA Total prob of N-in: 0.00414

Fusarium_sp-PT_009050-RA TMHMM2.0 outside 1 836

# Fusarium_sp-PT_009053-RA Length: 659

# Fusarium_sp-PT_009053-RA Number of predicted TMHs: 0

# Fusarium_sp-PT_009053-RA Exp number of AAs in TMHs: 0.02866

# Fusarium_sp-PT_009053-RA Exp number, first 60 AAs: 0.00847

# Fusarium_sp-PT_009053-RA Total prob of N-in: 0.00216

Fusarium_sp-PT_009053-RA TMHMM2.0 outside 1 659

# Fusarium_sp-PT_009056-RA Length: 623

# Fusarium_sp-PT_009056-RA Number of predicted TMHs: 0

# Fusarium_sp-PT_009056-RA Exp number of AAs in TMHs: 0.00693

# Fusarium_sp-PT_009056-RA Exp number, first 60 AAs: 0

# Fusarium_sp-PT_009056-RA Total prob of N-in: 0.00035

Fusarium_sp-PT_009056-RA TMHMM2.0 outside 1 623

# Fusarium_sp-PT_009058-RA Length: 923

# Fusarium_sp-PT_009058-RA Number of predicted TMHs: 0

# Fusarium_sp-PT_009058-RA Exp number of AAs in TMHs: 0.06483

# Fusarium_sp-PT_009058-RA Exp number, first 60 AAs: 0

# Fusarium_sp-PT_009058-RA Total prob of N-in: 0.00005

Fusarium_sp-PT_009058-RA TMHMM2.0 outside 1 923

# Fusarium_sp-PT_009060-RA Length: 907

# Fusarium_sp-PT_009060-RA Number of predicted TMHs: 0

# Fusarium_sp-PT_009060-RA Exp number of AAs in TMHs: 2.86026

# Fusarium_sp-PT_009060-RA Exp number, first 60 AAs: 0

# Fusarium_sp-PT_009060-RA Total prob of N-in: 0.00245

Fusarium_sp-PT_009060-RA TMHMM2.0 outside 1 907

# Fusarium_sp-PT_009062-RA Length: 415

# Fusarium_sp-PT_009062-RA Number of predicted TMHs: 0

# Fusarium_sp-PT_009062-RA Exp number of AAs in TMHs: 0.00722

# Fusarium_sp-PT_009062-RA Exp number, first 60 AAs: 0

# Fusarium_sp-PT_009062-RA Total prob of N-in: 0.00536

Fusarium_sp-PT_009062-RA TMHMM2.0 outside 1 415

# Fusarium_sp-PT_009064-RA Length: 234

# Fusarium_sp-PT_009064-RA Number of predicted TMHs: 0

# Fusarium_sp-PT_009064-RA Exp number of AAs in TMHs: 2.02554

# Fusarium_sp-PT_009064-RA Exp number, first 60 AAs: 1.90511

# Fusarium_sp-PT_009064-RA Total prob of N-in: 0.15999

Fusarium_sp-PT_009064-RA TMHMM2.0 outside 1 234

# Fusarium_sp-PT_009066-RA Length: 464

# Fusarium_sp-PT_009066-RA Number of predicted TMHs: 0

# Fusarium_sp-PT_009066-RA Exp number of AAs in TMHs: 0.64766

# Fusarium_sp-PT_009066-RA Exp number, first 60 AAs: 0.62207

# Fusarium_sp-PT_009066-RA Total prob of N-in: 0.01878

Fusarium_sp-PT_009066-RA TMHMM2.0 outside 1 464

# Fusarium_sp-PT_009067-RA Length: 255

# Fusarium_sp-PT_009067-RA Number of predicted TMHs: 0

# Fusarium_sp-PT_009067-RA Exp number of AAs in TMHs: 0.00126

# Fusarium_sp-PT_009067-RA Exp number, first 60 AAs: 0.00126

# Fusarium_sp-PT_009067-RA Total prob of N-in: 0.08647

Fusarium_sp-PT_009067-RA TMHMM2.0 outside 1 255

# Fusarium_sp-PT_009070-RA Length: 68

# Fusarium_sp-PT_009070-RA Number of predicted TMHs: 1

# Fusarium_sp-PT_009070-RA Exp number of AAs in TMHs: 17.99787

# Fusarium_sp-PT_009070-RA Exp number, first 60 AAs: 17.99787

# Fusarium_sp-PT_009070-RA Total prob of N-in: 0.93422

# Fusarium_sp-PT_009070-RA POSSIBLE N-term signal sequence

Fusarium_sp-PT_009070-RA TMHMM2.0 inside 1 12

Fusarium_sp-PT_009070-RA TMHMM2.0 TMhelix 13 30

Fusarium_sp-PT_009070-RA TMHMM2.0 outside 31 68

# Fusarium_sp-PT_009071-RA Length: 409

# Fusarium_sp-PT_009071-RA Number of predicted TMHs: 0

# Fusarium_sp-PT_009071-RA Exp number of AAs in TMHs: 0.0394500000000001

# Fusarium_sp-PT_009071-RA Exp number, first 60 AAs: 0.03521

# Fusarium_sp-PT_009071-RA Total prob of N-in: 0.00697

Fusarium_sp-PT_009071-RA TMHMM2.0 outside 1 409

# Fusarium_sp-PT_009055-RA Length: 185

# Fusarium_sp-PT_009055-RA Number of predicted TMHs: 1

# Fusarium_sp-PT_009055-RA Exp number of AAs in TMHs: 21.41518

# Fusarium_sp-PT_009055-RA Exp number, first 60 AAs: 21.41467

# Fusarium_sp-PT_009055-RA Total prob of N-in: 0.09427

# Fusarium_sp-PT_009055-RA POSSIBLE N-term signal sequence

Fusarium_sp-PT_009055-RA TMHMM2.0 outside 1 26

Fusarium_sp-PT_009055-RA TMHMM2.0 TMhelix 27 46

Fusarium_sp-PT_009055-RA TMHMM2.0 inside 47 185

# Fusarium_sp-PT_009068-RA Length: 77

# Fusarium_sp-PT_009068-RA Number of predicted TMHs: 0

# Fusarium_sp-PT_009068-RA Exp number of AAs in TMHs: 0.00144

# Fusarium_sp-PT_009068-RA Exp number, first 60 AAs: 0.00144

# Fusarium_sp-PT_009068-RA Total prob of N-in: 0.70615

Fusarium_sp-PT_009068-RA TMHMM2.0 inside 1 77

# Fusarium_sp-PT_009046-RA Length: 60

# Fusarium_sp-PT_009046-RA Number of predicted TMHs: 0

# Fusarium_sp-PT_009046-RA Exp number of AAs in TMHs: 0

# Fusarium_sp-PT_009046-RA Exp number, first 60 AAs: 0

# Fusarium_sp-PT_009046-RA Total prob of N-in: 0.38933

Fusarium_sp-PT_009046-RA TMHMM2.0 outside 1 60

# Fusarium_sp-PT_009076-RA Length: 638

# Fusarium_sp-PT_009076-RA Number of predicted TMHs: 1

# Fusarium_sp-PT_009076-RA Exp number of AAs in TMHs: 22.17576

# Fusarium_sp-PT_009076-RA Exp number, first 60 AAs: 0.00086

# Fusarium_sp-PT_009076-RA Total prob of N-in: 0.00008

Fusarium_sp-PT_009076-RA TMHMM2.0 outside 1 581

Fusarium_sp-PT_009076-RA TMHMM2.0 TMhelix 582 604

Fusarium_sp-PT_009076-RA TMHMM2.0 inside 605 638

# Fusarium_sp-PT_009078-RA Length: 188

# Fusarium_sp-PT_009078-RA Number of predicted TMHs: 0

# Fusarium_sp-PT_009078-RA Exp number of AAs in TMHs: 0.0528

# Fusarium_sp-PT_009078-RA Exp number, first 60 AAs: 0.05222

# Fusarium_sp-PT_009078-RA Total prob of N-in: 0.03020

Fusarium_sp-PT_009078-RA TMHMM2.0 outside 1 188

# Fusarium_sp-PT_009080-RA Length: 136

# Fusarium_sp-PT_009080-RA Number of predicted TMHs: 0

# Fusarium_sp-PT_009080-RA Exp number of AAs in TMHs: 0.00011

# Fusarium_sp-PT_009080-RA Exp number, first 60 AAs: 0.00011

# Fusarium_sp-PT_009080-RA Total prob of N-in: 0.23318

Fusarium_sp-PT_009080-RA TMHMM2.0 outside 1 136

# Fusarium_sp-PT_009082-RA Length: 845

# Fusarium_sp-PT_009082-RA Number of predicted TMHs: 0

# Fusarium_sp-PT_009082-RA Exp number of AAs in TMHs: 0.00082

# Fusarium_sp-PT_009082-RA Exp number, first 60 AAs: 0.00082

# Fusarium_sp-PT_009082-RA Total prob of N-in: 0.00009

Fusarium_sp-PT_009082-RA TMHMM2.0 outside 1 845

# Fusarium_sp-PT_009083-RA Length: 976

# Fusarium_sp-PT_009083-RA Number of predicted TMHs: 0

# Fusarium_sp-PT_009083-RA Exp number of AAs in TMHs: 0.01825

# Fusarium_sp-PT_009083-RA Exp number, first 60 AAs: 0.00062

# Fusarium_sp-PT_009083-RA Total prob of N-in: 0.00090

Fusarium_sp-PT_009083-RA TMHMM2.0 outside 1 976

# Fusarium_sp-PT_009085-RA Length: 1116

# Fusarium_sp-PT_009085-RA Number of predicted TMHs: 0

# Fusarium_sp-PT_009085-RA Exp number of AAs in TMHs: 0.01625

# Fusarium_sp-PT_009085-RA Exp number, first 60 AAs: 0.00024

# Fusarium_sp-PT_009085-RA Total prob of N-in: 0.00007

Fusarium_sp-PT_009085-RA TMHMM2.0 outside 1 1116

# Fusarium_sp-PT_009086-RA Length: 996

# Fusarium_sp-PT_009086-RA Number of predicted TMHs: 0

# Fusarium_sp-PT_009086-RA Exp number of AAs in TMHs: 0.00021

# Fusarium_sp-PT_009086-RA Exp number, first 60 AAs: 0.00021

# Fusarium_sp-PT_009086-RA Total prob of N-in: 0.00001

Fusarium_sp-PT_009086-RA TMHMM2.0 outside 1 996

# Fusarium_sp-PT_009087-RA Length: 401

# Fusarium_sp-PT_009087-RA Number of predicted TMHs: 3

# Fusarium_sp-PT_009087-RA Exp number of AAs in TMHs: 64.4538500000001

# Fusarium_sp-PT_009087-RA Exp number, first 60 AAs: 8.06391

# Fusarium_sp-PT_009087-RA Total prob of N-in: 0.92103

Fusarium_sp-PT_009087-RA TMHMM2.0 inside 1 51

Fusarium_sp-PT_009087-RA TMHMM2.0 TMhelix 52 74

Fusarium_sp-PT_009087-RA TMHMM2.0 outside 75 83

Fusarium_sp-PT_009087-RA TMHMM2.0 TMhelix 84 103

Fusarium_sp-PT_009087-RA TMHMM2.0 inside 104 123

Fusarium_sp-PT_009087-RA TMHMM2.0 TMhelix 124 146

Fusarium_sp-PT_009087-RA TMHMM2.0 outside 147 401

# Fusarium_sp-PT_009089-RA Length: 305

# Fusarium_sp-PT_009089-RA Number of predicted TMHs: 0

# Fusarium_sp-PT_009089-RA Exp number of AAs in TMHs: 0.01073

# Fusarium_sp-PT_009089-RA Exp number, first 60 AAs: 0.0006

# Fusarium_sp-PT_009089-RA Total prob of N-in: 0.00933

Fusarium_sp-PT_009089-RA TMHMM2.0 outside 1 305

# Fusarium_sp-PT_009090-RA Length: 67

# Fusarium_sp-PT_009090-RA Number of predicted TMHs: 0

# Fusarium_sp-PT_009090-RA Exp number of AAs in TMHs: 0

# Fusarium_sp-PT_009090-RA Exp number, first 60 AAs: 0

# Fusarium_sp-PT_009090-RA Total prob of N-in: 0.18932

Fusarium_sp-PT_009090-RA TMHMM2.0 outside 1 67

# Fusarium_sp-PT_009091-RA Length: 373

# Fusarium_sp-PT_009091-RA Number of predicted TMHs: 1

# Fusarium_sp-PT_009091-RA Exp number of AAs in TMHs: 21.15435

# Fusarium_sp-PT_009091-RA Exp number, first 60 AAs: 21.15435

# Fusarium_sp-PT_009091-RA Total prob of N-in: 0.78691

# Fusarium_sp-PT_009091-RA POSSIBLE N-term signal sequence

Fusarium_sp-PT_009091-RA TMHMM2.0 inside 1 4

Fusarium_sp-PT_009091-RA TMHMM2.0 TMhelix 5 27

Fusarium_sp-PT_009091-RA TMHMM2.0 outside 28 373

# Fusarium_sp-PT_009093-RA Length: 1165

# Fusarium_sp-PT_009093-RA Number of predicted TMHs: 0

# Fusarium_sp-PT_009093-RA Exp number of AAs in TMHs: 0.01791

# Fusarium_sp-PT_009093-RA Exp number, first 60 AAs: 0

# Fusarium_sp-PT_009093-RA Total prob of N-in: 0.00090

Fusarium_sp-PT_009093-RA TMHMM2.0 outside 1 1165

# Fusarium_sp-PT_009096-RA Length: 780

# Fusarium_sp-PT_009096-RA Number of predicted TMHs: 0

# Fusarium_sp-PT_009096-RA Exp number of AAs in TMHs: 0.00136

# Fusarium_sp-PT_009096-RA Exp number, first 60 AAs: 0

# Fusarium_sp-PT_009096-RA Total prob of N-in: 0.00003

Fusarium_sp-PT_009096-RA TMHMM2.0 outside 1 780

# Fusarium_sp-PT_009099-RA Length: 783

# Fusarium_sp-PT_009099-RA Number of predicted TMHs: 0

# Fusarium_sp-PT_009099-RA Exp number of AAs in TMHs: 0.01101

# Fusarium_sp-PT_009099-RA Exp number, first 60 AAs: 0

# Fusarium_sp-PT_009099-RA Total prob of N-in: 0.00052

Fusarium_sp-PT_009099-RA TMHMM2.0 outside 1 783

# Fusarium_sp-PT_009074-RA Length: 640

# Fusarium_sp-PT_009074-RA Number of predicted TMHs: 12

# Fusarium_sp-PT_009074-RA Exp number of AAs in TMHs: 243.98017

# Fusarium_sp-PT_009074-RA Exp number, first 60 AAs: 0.00432

# Fusarium_sp-PT_009074-RA Total prob of N-in: 0.68532

Fusarium_sp-PT_009074-RA TMHMM2.0 inside 1 203

Fusarium_sp-PT_009074-RA TMHMM2.0 TMhelix 204 226

Fusarium_sp-PT_009074-RA TMHMM2.0 outside 227 235

Fusarium_sp-PT_009074-RA TMHMM2.0 TMhelix 236 258

Fusarium_sp-PT_009074-RA TMHMM2.0 inside 259 287

Fusarium_sp-PT_009074-RA TMHMM2.0 TMhelix 288 310

Fusarium_sp-PT_009074-RA TMHMM2.0 outside 311 319

Fusarium_sp-PT_009074-RA TMHMM2.0 TMhelix 320 339

Fusarium_sp-PT_009074-RA TMHMM2.0 inside 340 351

Fusarium_sp-PT_009074-RA TMHMM2.0 TMhelix 352 371

Fusarium_sp-PT_009074-RA TMHMM2.0 outside 372 380

Fusarium_sp-PT_009074-RA TMHMM2.0 TMhelix 381 403

Fusarium_sp-PT_009074-RA TMHMM2.0 inside 404 423

Fusarium_sp-PT_009074-RA TMHMM2.0 TMhelix 424 446

Fusarium_sp-PT_009074-RA TMHMM2.0 outside 447 455

Fusarium_sp-PT_009074-RA TMHMM2.0 TMhelix 456 478

Fusarium_sp-PT_009074-RA TMHMM2.0 inside 479 498

Fusarium_sp-PT_009074-RA TMHMM2.0 TMhelix 499 521

Fusarium_sp-PT_009074-RA TMHMM2.0 outside 522 540

Fusarium_sp-PT_009074-RA TMHMM2.0 TMhelix 541 563

Fusarium_sp-PT_009074-RA TMHMM2.0 inside 564 575

Fusarium_sp-PT_009074-RA TMHMM2.0 TMhelix 576 595

Fusarium_sp-PT_009074-RA TMHMM2.0 outside 596 599

Fusarium_sp-PT_009074-RA TMHMM2.0 TMhelix 600 622

Fusarium_sp-PT_009074-RA TMHMM2.0 inside 623 640

# Fusarium_sp-PT_009075-RA Length: 514

# Fusarium_sp-PT_009075-RA Number of predicted TMHs: 0

# Fusarium_sp-PT_009075-RA Exp number of AAs in TMHs: 0.00748999999999999

# Fusarium_sp-PT_009075-RA Exp number, first 60 AAs: 0

# Fusarium_sp-PT_009075-RA Total prob of N-in: 0.01971

Fusarium_sp-PT_009075-RA TMHMM2.0 outside 1 514

# Fusarium_sp-PT_009077-RA Length: 1195

# Fusarium_sp-PT_009077-RA Number of predicted TMHs: 0

# Fusarium_sp-PT_009077-RA Exp number of AAs in TMHs: 0.00018

# Fusarium_sp-PT_009077-RA Exp number, first 60 AAs: 0

# Fusarium_sp-PT_009077-RA Total prob of N-in: 0.00001

Fusarium_sp-PT_009077-RA TMHMM2.0 outside 1 1195

# Fusarium_sp-PT_009079-RA Length: 382

# Fusarium_sp-PT_009079-RA Number of predicted TMHs: 2

# Fusarium_sp-PT_009079-RA Exp number of AAs in TMHs: 46.38456

# Fusarium_sp-PT_009079-RA Exp number, first 60 AAs: 0

# Fusarium_sp-PT_009079-RA Total prob of N-in: 0.11386

Fusarium_sp-PT_009079-RA TMHMM2.0 outside 1 231

Fusarium_sp-PT_009079-RA TMHMM2.0 TMhelix 232 254

Fusarium_sp-PT_009079-RA TMHMM2.0 inside 255 260

Fusarium_sp-PT_009079-RA TMHMM2.0 TMhelix 261 283

Fusarium_sp-PT_009079-RA TMHMM2.0 outside 284 382

# Fusarium_sp-PT_009081-RA Length: 290

# Fusarium_sp-PT_009081-RA Number of predicted TMHs: 0

# Fusarium_sp-PT_009081-RA Exp number of AAs in TMHs: 0.000450000000000001

# Fusarium_sp-PT_009081-RA Exp number, first 60 AAs: 0.00039

# Fusarium_sp-PT_009081-RA Total prob of N-in: 0.05049

Fusarium_sp-PT_009081-RA TMHMM2.0 outside 1 290

# Fusarium_sp-PT_009084-RA Length: 168

# Fusarium_sp-PT_009084-RA Number of predicted TMHs: 0

# Fusarium_sp-PT_009084-RA Exp number of AAs in TMHs: 0.0002

# Fusarium_sp-PT_009084-RA Exp number, first 60 AAs: 0

# Fusarium_sp-PT_009084-RA Total prob of N-in: 0.08208

Fusarium_sp-PT_009084-RA TMHMM2.0 outside 1 168

# Fusarium_sp-PT_009088-RA Length: 443

# Fusarium_sp-PT_009088-RA Number of predicted TMHs: 0

# Fusarium_sp-PT_009088-RA Exp number of AAs in TMHs: 0.0034

# Fusarium_sp-PT_009088-RA Exp number, first 60 AAs: 0

# Fusarium_sp-PT_009088-RA Total prob of N-in: 0.00081

Fusarium_sp-PT_009088-RA TMHMM2.0 outside 1 443

# Fusarium_sp-PT_009092-RA Length: 241

# Fusarium_sp-PT_009092-RA Number of predicted TMHs: 0

# Fusarium_sp-PT_009092-RA Exp number of AAs in TMHs: 0.002

# Fusarium_sp-PT_009092-RA Exp number, first 60 AAs: 0.00091

# Fusarium_sp-PT_009092-RA Total prob of N-in: 0.03703

Fusarium_sp-PT_009092-RA TMHMM2.0 outside 1 241

# Fusarium_sp-PT_009094-RA Length: 1174

# Fusarium_sp-PT_009094-RA Number of predicted TMHs: 0

# Fusarium_sp-PT_009094-RA Exp number of AAs in TMHs: 0.00301

# Fusarium_sp-PT_009094-RA Exp number, first 60 AAs: 0

# Fusarium_sp-PT_009094-RA Total prob of N-in: 0.00016

Fusarium_sp-PT_009094-RA TMHMM2.0 outside 1 1174

# Fusarium_sp-PT_009095-RA Length: 209

# Fusarium_sp-PT_009095-RA Number of predicted TMHs: 0

# Fusarium_sp-PT_009095-RA Exp number of AAs in TMHs: 0

# Fusarium_sp-PT_009095-RA Exp number, first 60 AAs: 0

# Fusarium_sp-PT_009095-RA Total prob of N-in: 0.28072

Fusarium_sp-PT_009095-RA TMHMM2.0 outside 1 209

# Fusarium_sp-PT_009097-RA Length: 919

# Fusarium_sp-PT_009097-RA Number of predicted TMHs: 0

# Fusarium_sp-PT_009097-RA Exp number of AAs in TMHs: 0.0626700000000001

# Fusarium_sp-PT_009097-RA Exp number, first 60 AAs: 0.01402

# Fusarium_sp-PT_009097-RA Total prob of N-in: 0.00313

Fusarium_sp-PT_009097-RA TMHMM2.0 outside 1 919

# Fusarium_sp-PT_009098-RA Length: 169

# Fusarium_sp-PT_009098-RA Number of predicted TMHs: 0

# Fusarium_sp-PT_009098-RA Exp number of AAs in TMHs: 0

# Fusarium_sp-PT_009098-RA Exp number, first 60 AAs: 0

# Fusarium_sp-PT_009098-RA Total prob of N-in: 0.12318

Fusarium_sp-PT_009098-RA TMHMM2.0 outside 1 169

# Fusarium_sp-PT_009100-RA Length: 235

# Fusarium_sp-PT_009100-RA Number of predicted TMHs: 0

# Fusarium_sp-PT_009100-RA Exp number of AAs in TMHs: 0.29756

# Fusarium_sp-PT_009100-RA Exp number, first 60 AAs: 0.00384

# Fusarium_sp-PT_009100-RA Total prob of N-in: 0.06545

Fusarium_sp-PT_009100-RA TMHMM2.0 outside 1 235

# Fusarium_sp-PT_009101-RA Length: 196

# Fusarium_sp-PT_009101-RA Number of predicted TMHs: 0

# Fusarium_sp-PT_009101-RA Exp number of AAs in TMHs: 0.00039

# Fusarium_sp-PT_009101-RA Exp number, first 60 AAs: 0

# Fusarium_sp-PT_009101-RA Total prob of N-in: 0.06719

Fusarium_sp-PT_009101-RA TMHMM2.0 outside 1 196

# Fusarium_sp-PT_009102-RA Length: 133

# Fusarium_sp-PT_009102-RA Number of predicted TMHs: 0

# Fusarium_sp-PT_009102-RA Exp number of AAs in TMHs: 0.03861

# Fusarium_sp-PT_009102-RA Exp number, first 60 AAs: 0.03828

# Fusarium_sp-PT_009102-RA Total prob of N-in: 0.31065

Fusarium_sp-PT_009102-RA TMHMM2.0 outside 1 133

# Fusarium_sp-PT_009104-RA Length: 364

# Fusarium_sp-PT_009104-RA Number of predicted TMHs: 0

# Fusarium_sp-PT_009104-RA Exp number of AAs in TMHs: 5.3206

# Fusarium_sp-PT_009104-RA Exp number, first 60 AAs: 0.1838

# Fusarium_sp-PT_009104-RA Total prob of N-in: 0.13331

Fusarium_sp-PT_009104-RA TMHMM2.0 outside 1 364

# Fusarium_sp-PT_009106-RA Length: 156

# Fusarium_sp-PT_009106-RA Number of predicted TMHs: 0

# Fusarium_sp-PT_009106-RA Exp number of AAs in TMHs: 0.0260399999999999

# Fusarium_sp-PT_009106-RA Exp number, first 60 AAs: 0.02413

# Fusarium_sp-PT_009106-RA Total prob of N-in: 0.10946

Fusarium_sp-PT_009106-RA TMHMM2.0 outside 1 156

# Fusarium_sp-PT_009107-RA Length: 749

# Fusarium_sp-PT_009107-RA Number of predicted TMHs: 0

# Fusarium_sp-PT_009107-RA Exp number of AAs in TMHs: 0.186

# Fusarium_sp-PT_009107-RA Exp number, first 60 AAs: 0

# Fusarium_sp-PT_009107-RA Total prob of N-in: 0.00011

Fusarium_sp-PT_009107-RA TMHMM2.0 outside 1 749

# Fusarium_sp-PT_009108-RA Length: 104

# Fusarium_sp-PT_009108-RA Number of predicted TMHs: 0

# Fusarium_sp-PT_009108-RA Exp number of AAs in TMHs: 0.00054

# Fusarium_sp-PT_009108-RA Exp number, first 60 AAs: 0

# Fusarium_sp-PT_009108-RA Total prob of N-in: 0.48163

Fusarium_sp-PT_009108-RA TMHMM2.0 outside 1 104

# Fusarium_sp-PT_009109-RA Length: 497

# Fusarium_sp-PT_009109-RA Number of predicted TMHs: 0

# Fusarium_sp-PT_009109-RA Exp number of AAs in TMHs: 0.77505

# Fusarium_sp-PT_009109-RA Exp number, first 60 AAs: 0.75939

# Fusarium_sp-PT_009109-RA Total prob of N-in: 0.03676

Fusarium_sp-PT_009109-RA TMHMM2.0 outside 1 497

# Fusarium_sp-PT_009110-RA Length: 203

# Fusarium_sp-PT_009110-RA Number of predicted TMHs: 0

# Fusarium_sp-PT_009110-RA Exp number of AAs in TMHs: 0.11373

# Fusarium_sp-PT_009110-RA Exp number, first 60 AAs: 0.04364

# Fusarium_sp-PT_009110-RA Total prob of N-in: 0.10279

Fusarium_sp-PT_009110-RA TMHMM2.0 outside 1 203

# Fusarium_sp-PT_009112-RA Length: 737

# Fusarium_sp-PT_009112-RA Number of predicted TMHs: 0

# Fusarium_sp-PT_009112-RA Exp number of AAs in TMHs: 0.00369

# Fusarium_sp-PT_009112-RA Exp number, first 60 AAs: 0.00203

# Fusarium_sp-PT_009112-RA Total prob of N-in: 0.00034

Fusarium_sp-PT_009112-RA TMHMM2.0 outside 1 737

# Fusarium_sp-PT_009114-RA Length: 176

# Fusarium_sp-PT_009114-RA Number of predicted TMHs: 0

# Fusarium_sp-PT_009114-RA Exp number of AAs in TMHs: 0.0003

# Fusarium_sp-PT_009114-RA Exp number, first 60 AAs: 0

# Fusarium_sp-PT_009114-RA Total prob of N-in: 0.15591

Fusarium_sp-PT_009114-RA TMHMM2.0 outside 1 176

# Fusarium_sp-PT_009115-RA Length: 414

# Fusarium_sp-PT_009115-RA Number of predicted TMHs: 1

# Fusarium_sp-PT_009115-RA Exp number of AAs in TMHs: 24.40385

# Fusarium_sp-PT_009115-RA Exp number, first 60 AAs: 22.85008

# Fusarium_sp-PT_009115-RA Total prob of N-in: 0.76680

# Fusarium_sp-PT_009115-RA POSSIBLE N-term signal sequence

Fusarium_sp-PT_009115-RA TMHMM2.0 inside 1 25

Fusarium_sp-PT_009115-RA TMHMM2.0 TMhelix 26 48

Fusarium_sp-PT_009115-RA TMHMM2.0 outside 49 414

# Fusarium_sp-PT_009118-RA Length: 384

# Fusarium_sp-PT_009118-RA Number of predicted TMHs: 0

# Fusarium_sp-PT_009118-RA Exp number of AAs in TMHs: 0.36897

# Fusarium_sp-PT_009118-RA Exp number, first 60 AAs: 0.06143

# Fusarium_sp-PT_009118-RA Total prob of N-in: 0.00239

Fusarium_sp-PT_009118-RA TMHMM2.0 outside 1 384

# Fusarium_sp-PT_009120-RA Length: 310

# Fusarium_sp-PT_009120-RA Number of predicted TMHs: 0

# Fusarium_sp-PT_009120-RA Exp number of AAs in TMHs: 0.00769

# Fusarium_sp-PT_009120-RA Exp number, first 60 AAs: 0.00296

# Fusarium_sp-PT_009120-RA Total prob of N-in: 0.06932

Fusarium_sp-PT_009120-RA TMHMM2.0 outside 1 310

# Fusarium_sp-PT_009121-RA Length: 186

# Fusarium_sp-PT_009121-RA Number of predicted TMHs: 1

# Fusarium_sp-PT_009121-RA Exp number of AAs in TMHs: 17.72873

# Fusarium_sp-PT_009121-RA Exp number, first 60 AAs: 17.72558

# Fusarium_sp-PT_009121-RA Total prob of N-in: 0.61115

# Fusarium_sp-PT_009121-RA POSSIBLE N-term signal sequence

Fusarium_sp-PT_009121-RA TMHMM2.0 inside 1 20

Fusarium_sp-PT_009121-RA TMHMM2.0 TMhelix 21 43

Fusarium_sp-PT_009121-RA TMHMM2.0 outside 44 186

# Fusarium_sp-PT_009122-RA Length: 401

# Fusarium_sp-PT_009122-RA Number of predicted TMHs: 2

# Fusarium_sp-PT_009122-RA Exp number of AAs in TMHs: 56.38286

# Fusarium_sp-PT_009122-RA Exp number, first 60 AAs: 0

# Fusarium_sp-PT_009122-RA Total prob of N-in: 0.88691

Fusarium_sp-PT_009122-RA TMHMM2.0 inside 1 128

Fusarium_sp-PT_009122-RA TMHMM2.0 TMhelix 129 151

Fusarium_sp-PT_009122-RA TMHMM2.0 outside 152 165

Fusarium_sp-PT_009122-RA TMHMM2.0 TMhelix 166 185

Fusarium_sp-PT_009122-RA TMHMM2.0 inside 186 401

# Fusarium_sp-PT_009124-RA Length: 201

# Fusarium_sp-PT_009124-RA Number of predicted TMHs: 0

# Fusarium_sp-PT_009124-RA Exp number of AAs in TMHs: 0.00056

# Fusarium_sp-PT_009124-RA Exp number, first 60 AAs: 0.00056

# Fusarium_sp-PT_009124-RA Total prob of N-in: 0.26271

Fusarium_sp-PT_009124-RA TMHMM2.0 outside 1 201

# Fusarium_sp-PT_009126-RA Length: 396

# Fusarium_sp-PT_009126-RA Number of predicted TMHs: 1

# Fusarium_sp-PT_009126-RA Exp number of AAs in TMHs: 36.4195

# Fusarium_sp-PT_009126-RA Exp number, first 60 AAs: 0.02853

# Fusarium_sp-PT_009126-RA Total prob of N-in: 0.21043

Fusarium_sp-PT_009126-RA TMHMM2.0 outside 1 364

Fusarium_sp-PT_009126-RA TMHMM2.0 TMhelix 365 387

Fusarium_sp-PT_009126-RA TMHMM2.0 inside 388 396

# Fusarium_sp-PT_009127-RA Length: 410

# Fusarium_sp-PT_009127-RA Number of predicted TMHs: 0

# Fusarium_sp-PT_009127-RA Exp number of AAs in TMHs: 0.00187

# Fusarium_sp-PT_009127-RA Exp number, first 60 AAs: 0.00018

# Fusarium_sp-PT_009127-RA Total prob of N-in: 0.00222

Fusarium_sp-PT_009127-RA TMHMM2.0 outside 1 410

# Fusarium_sp-PT_009128-RA Length: 717

# Fusarium_sp-PT_009128-RA Number of predicted TMHs: 0

# Fusarium_sp-PT_009128-RA Exp number of AAs in TMHs: 0.01726

# Fusarium_sp-PT_009128-RA Exp number, first 60 AAs: 0

# Fusarium_sp-PT_009128-RA Total prob of N-in: 0.00051

Fusarium_sp-PT_009128-RA TMHMM2.0 outside 1 717

# Fusarium_sp-PT_009129-RA Length: 233

# Fusarium_sp-PT_009129-RA Number of predicted TMHs: 1

# Fusarium_sp-PT_009129-RA Exp number of AAs in TMHs: 21.85238

# Fusarium_sp-PT_009129-RA Exp number, first 60 AAs: 3.37184

# Fusarium_sp-PT_009129-RA Total prob of N-in: 0.25434

Fusarium_sp-PT_009129-RA TMHMM2.0 outside 1 209

Fusarium_sp-PT_009129-RA TMHMM2.0 TMhelix 210 232

Fusarium_sp-PT_009129-RA TMHMM2.0 inside 233 233

# Fusarium_sp-PT_009132-RA Length: 633

# Fusarium_sp-PT_009132-RA Number of predicted TMHs: 11

# Fusarium_sp-PT_009132-RA Exp number of AAs in TMHs: 230.25161

# Fusarium_sp-PT_009132-RA Exp number, first 60 AAs: 0.00265

# Fusarium_sp-PT_009132-RA Total prob of N-in: 0.80124

Fusarium_sp-PT_009132-RA TMHMM2.0 inside 1 95

Fusarium_sp-PT_009132-RA TMHMM2.0 TMhelix 96 118

Fusarium_sp-PT_009132-RA TMHMM2.0 outside 119 127

Fusarium_sp-PT_009132-RA TMHMM2.0 TMhelix 128 146

Fusarium_sp-PT_009132-RA TMHMM2.0 inside 147 158

Fusarium_sp-PT_009132-RA TMHMM2.0 TMhelix 159 181

Fusarium_sp-PT_009132-RA TMHMM2.0 outside 182 190

Fusarium_sp-PT_009132-RA TMHMM2.0 TMhelix 191 210

Fusarium_sp-PT_009132-RA TMHMM2.0 inside 211 221

Fusarium_sp-PT_009132-RA TMHMM2.0 TMhelix 222 244

Fusarium_sp-PT_009132-RA TMHMM2.0 outside 245 253

Fusarium_sp-PT_009132-RA TMHMM2.0 TMhelix 254 276

Fusarium_sp-PT_009132-RA TMHMM2.0 inside 277 287

Fusarium_sp-PT_009132-RA TMHMM2.0 TMhelix 288 310

Fusarium_sp-PT_009132-RA TMHMM2.0 outside 311 324

Fusarium_sp-PT_009132-RA TMHMM2.0 TMhelix 325 347

Fusarium_sp-PT_009132-RA TMHMM2.0 inside 348 499

Fusarium_sp-PT_009132-RA TMHMM2.0 TMhelix 500 522

Fusarium_sp-PT_009132-RA TMHMM2.0 outside 523 531

Fusarium_sp-PT_009132-RA TMHMM2.0 TMhelix 532 551

Fusarium_sp-PT_009132-RA TMHMM2.0 inside 552 563

Fusarium_sp-PT_009132-RA TMHMM2.0 TMhelix 564 586

Fusarium_sp-PT_009132-RA TMHMM2.0 outside 587 633

# Fusarium_sp-PT_009135-RA Length: 373

# Fusarium_sp-PT_009135-RA Number of predicted TMHs: 0

# Fusarium_sp-PT_009135-RA Exp number of AAs in TMHs: 0.000750000000000001

# Fusarium_sp-PT_009135-RA Exp number, first 60 AAs: 0.000660000000000001

# Fusarium_sp-PT_009135-RA Total prob of N-in: 0.00146

Fusarium_sp-PT_009135-RA TMHMM2.0 outside 1 373

# Fusarium_sp-PT_009103-RA Length: 324

# Fusarium_sp-PT_009103-RA Number of predicted TMHs: 5

# Fusarium_sp-PT_009103-RA Exp number of AAs in TMHs: 165.84974

# Fusarium_sp-PT_009103-RA Exp number, first 60 AAs: 33.92995

# Fusarium_sp-PT_009103-RA Total prob of N-in: 0.79105

# Fusarium_sp-PT_009103-RA POSSIBLE N-term signal sequence

Fusarium_sp-PT_009103-RA TMHMM2.0 outside 1 3

Fusarium_sp-PT_009103-RA TMHMM2.0 TMhelix 4 26

Fusarium_sp-PT_009103-RA TMHMM2.0 inside 27 79

Fusarium_sp-PT_009103-RA TMHMM2.0 TMhelix 80 102

Fusarium_sp-PT_009103-RA TMHMM2.0 outside 103 106

Fusarium_sp-PT_009103-RA TMHMM2.0 TMhelix 107 129

Fusarium_sp-PT_009103-RA TMHMM2.0 inside 130 160

Fusarium_sp-PT_009103-RA TMHMM2.0 TMhelix 161 180

Fusarium_sp-PT_009103-RA TMHMM2.0 outside 181 183

Fusarium_sp-PT_009103-RA TMHMM2.0 TMhelix 184 206

Fusarium_sp-PT_009103-RA TMHMM2.0 inside 207 324

# Fusarium_sp-PT_009105-RA Length: 326

# Fusarium_sp-PT_009105-RA Number of predicted TMHs: 0

# Fusarium_sp-PT_009105-RA Exp number of AAs in TMHs: 0.38778

# Fusarium_sp-PT_009105-RA Exp number, first 60 AAs: 0

# Fusarium_sp-PT_009105-RA Total prob of N-in: 0.04974

Fusarium_sp-PT_009105-RA TMHMM2.0 outside 1 326

# Fusarium_sp-PT_009111-RA Length: 262

# Fusarium_sp-PT_009111-RA Number of predicted TMHs: 0

# Fusarium_sp-PT_009111-RA Exp number of AAs in TMHs: 0.00556

# Fusarium_sp-PT_009111-RA Exp number, first 60 AAs: 0.00438

# Fusarium_sp-PT_009111-RA Total prob of N-in: 0.01089

Fusarium_sp-PT_009111-RA TMHMM2.0 outside 1 262

# Fusarium_sp-PT_009113-RA Length: 860

# Fusarium_sp-PT_009113-RA Number of predicted TMHs: 0

# Fusarium_sp-PT_009113-RA Exp number of AAs in TMHs: 0.07202

# Fusarium_sp-PT_009113-RA Exp number, first 60 AAs: 0

# Fusarium_sp-PT_009113-RA Total prob of N-in: 0.00011

Fusarium_sp-PT_009113-RA TMHMM2.0 outside 1 860

# Fusarium_sp-PT_009116-RA Length: 500

# Fusarium_sp-PT_009116-RA Number of predicted TMHs: 3

# Fusarium_sp-PT_009116-RA Exp number of AAs in TMHs: 69.68536

# Fusarium_sp-PT_009116-RA Exp number, first 60 AAs: 18.59967

# Fusarium_sp-PT_009116-RA Total prob of N-in: 0.90513

# Fusarium_sp-PT_009116-RA POSSIBLE N-term signal sequence

Fusarium_sp-PT_009116-RA TMHMM2.0 inside 1 24

Fusarium_sp-PT_009116-RA TMHMM2.0 TMhelix 25 42

Fusarium_sp-PT_009116-RA TMHMM2.0 outside 43 443

Fusarium_sp-PT_009116-RA TMHMM2.0 TMhelix 444 466

Fusarium_sp-PT_009116-RA TMHMM2.0 inside 467 472

Fusarium_sp-PT_009116-RA TMHMM2.0 TMhelix 473 495

Fusarium_sp-PT_009116-RA TMHMM2.0 outside 496 500

# Fusarium_sp-PT_009117-RA Length: 971

# Fusarium_sp-PT_009117-RA Number of predicted TMHs: 0

# Fusarium_sp-PT_009117-RA Exp number of AAs in TMHs: 3.34636

# Fusarium_sp-PT_009117-RA Exp number, first 60 AAs: 3.34352

# Fusarium_sp-PT_009117-RA Total prob of N-in: 0.17473

Fusarium_sp-PT_009117-RA TMHMM2.0 outside 1 971

# Fusarium_sp-PT_009119-RA Length: 368

# Fusarium_sp-PT_009119-RA Number of predicted TMHs: 0

# Fusarium_sp-PT_009119-RA Exp number of AAs in TMHs: 0.0650900000000001

# Fusarium_sp-PT_009119-RA Exp number, first 60 AAs: 0.04965

# Fusarium_sp-PT_009119-RA Total prob of N-in: 0.00958

Fusarium_sp-PT_009119-RA TMHMM2.0 outside 1 368

# Fusarium_sp-PT_009123-RA Length: 419

# Fusarium_sp-PT_009123-RA Number of predicted TMHs: 0

# Fusarium_sp-PT_009123-RA Exp number of AAs in TMHs: 0.38277

# Fusarium_sp-PT_009123-RA Exp number, first 60 AAs: 0

# Fusarium_sp-PT_009123-RA Total prob of N-in: 0.02837

Fusarium_sp-PT_009123-RA TMHMM2.0 outside 1 419

# Fusarium_sp-PT_009125-RA Length: 331

# Fusarium_sp-PT_009125-RA Number of predicted TMHs: 0

# Fusarium_sp-PT_009125-RA Exp number of AAs in TMHs: 0.02361

# Fusarium_sp-PT_009125-RA Exp number, first 60 AAs: 0

# Fusarium_sp-PT_009125-RA Total prob of N-in: 0.00402

Fusarium_sp-PT_009125-RA TMHMM2.0 outside 1 331

# Fusarium_sp-PT_009130-RA Length: 300

# Fusarium_sp-PT_009130-RA Number of predicted TMHs: 0

# Fusarium_sp-PT_009130-RA Exp number of AAs in TMHs: 0.00539

# Fusarium_sp-PT_009130-RA Exp number, first 60 AAs: 0.00536

# Fusarium_sp-PT_009130-RA Total prob of N-in: 0.00525

Fusarium_sp-PT_009130-RA TMHMM2.0 outside 1 300

# Fusarium_sp-PT_009131-RA Length: 768

# Fusarium_sp-PT_009131-RA Number of predicted TMHs: 0

# Fusarium_sp-PT_009131-RA Exp number of AAs in TMHs: 0.3332

# Fusarium_sp-PT_009131-RA Exp number, first 60 AAs: 0

# Fusarium_sp-PT_009131-RA Total prob of N-in: 0.00006

Fusarium_sp-PT_009131-RA TMHMM2.0 outside 1 768

# Fusarium_sp-PT_009133-RA Length: 328

# Fusarium_sp-PT_009133-RA Number of predicted TMHs: 0

# Fusarium_sp-PT_009133-RA Exp number of AAs in TMHs: 0.00037

# Fusarium_sp-PT_009133-RA Exp number, first 60 AAs: 0.00021

# Fusarium_sp-PT_009133-RA Total prob of N-in: 0.00256

Fusarium_sp-PT_009133-RA TMHMM2.0 outside 1 328

# Fusarium_sp-PT_009134-RA Length: 486

# Fusarium_sp-PT_009134-RA Number of predicted TMHs: 0

# Fusarium_sp-PT_009134-RA Exp number of AAs in TMHs: 0.04928

# Fusarium_sp-PT_009134-RA Exp number, first 60 AAs: 0

# Fusarium_sp-PT_009134-RA Total prob of N-in: 0.00503

Fusarium_sp-PT_009134-RA TMHMM2.0 outside 1 486

# Fusarium_sp-PT_009136-RA Length: 790

# Fusarium_sp-PT_009136-RA Number of predicted TMHs: 1

# Fusarium_sp-PT_009136-RA Exp number of AAs in TMHs: 22.90502

# Fusarium_sp-PT_009136-RA Exp number, first 60 AAs: 0.00662

# Fusarium_sp-PT_009136-RA Total prob of N-in: 0.00315

Fusarium_sp-PT_009136-RA TMHMM2.0 outside 1 485

Fusarium_sp-PT_009136-RA TMHMM2.0 TMhelix 486 508

Fusarium_sp-PT_009136-RA TMHMM2.0 inside 509 790

# Fusarium_sp-PT_009137-RA Length: 1262

# Fusarium_sp-PT_009137-RA Number of predicted TMHs: 0

# Fusarium_sp-PT_009137-RA Exp number of AAs in TMHs: 5.67972

# Fusarium_sp-PT_009137-RA Exp number, first 60 AAs: 0.03224

# Fusarium_sp-PT_009137-RA Total prob of N-in: 0.00185

Fusarium_sp-PT_009137-RA TMHMM2.0 outside 1 1262

# Fusarium_sp-PT_009140-RA Length: 147

# Fusarium_sp-PT_009140-RA Number of predicted TMHs: 0

# Fusarium_sp-PT_009140-RA Exp number of AAs in TMHs: 0.00253

# Fusarium_sp-PT_009140-RA Exp number, first 60 AAs: 0

# Fusarium_sp-PT_009140-RA Total prob of N-in: 0.22295

Fusarium_sp-PT_009140-RA TMHMM2.0 outside 1 147

# Fusarium_sp-PT_009141-RA Length: 704

# Fusarium_sp-PT_009141-RA Number of predicted TMHs: 0

# Fusarium_sp-PT_009141-RA Exp number of AAs in TMHs: 0.1209

# Fusarium_sp-PT_009141-RA Exp number, first 60 AAs: 0.0001

# Fusarium_sp-PT_009141-RA Total prob of N-in: 0.00028

Fusarium_sp-PT_009141-RA TMHMM2.0 outside 1 704

# Fusarium_sp-PT_009142-RA Length: 497

# Fusarium_sp-PT_009142-RA Number of predicted TMHs: 12

# Fusarium_sp-PT_009142-RA Exp number of AAs in TMHs: 255.81378

# Fusarium_sp-PT_009142-RA Exp number, first 60 AAs: 10.69031

# Fusarium_sp-PT_009142-RA Total prob of N-in: 0.88226

# Fusarium_sp-PT_009142-RA POSSIBLE N-term signal sequence

Fusarium_sp-PT_009142-RA TMHMM2.0 inside 1 48

Fusarium_sp-PT_009142-RA TMHMM2.0 TMhelix 49 66

Fusarium_sp-PT_009142-RA TMHMM2.0 outside 67 85

Fusarium_sp-PT_009142-RA TMHMM2.0 TMhelix 86 108

Fusarium_sp-PT_009142-RA TMHMM2.0 inside 109 114

Fusarium_sp-PT_009142-RA TMHMM2.0 TMhelix 115 134

Fusarium_sp-PT_009142-RA TMHMM2.0 outside 135 139

Fusarium_sp-PT_009142-RA TMHMM2.0 TMhelix 140 162

Fusarium_sp-PT_009142-RA TMHMM2.0 inside 163 174

Fusarium_sp-PT_009142-RA TMHMM2.0 TMhelix 175 197

Fusarium_sp-PT_009142-RA TMHMM2.0 outside 198 206

Fusarium_sp-PT_009142-RA TMHMM2.0 TMhelix 207 229

Fusarium_sp-PT_009142-RA TMHMM2.0 inside 230 281

Fusarium_sp-PT_009142-RA TMHMM2.0 TMhelix 282 304

Fusarium_sp-PT_009142-RA TMHMM2.0 outside 305 318

Fusarium_sp-PT_009142-RA TMHMM2.0 TMhelix 319 340

Fusarium_sp-PT_009142-RA TMHMM2.0 inside 341 346

Fusarium_sp-PT_009142-RA TMHMM2.0 TMhelix 347 366

Fusarium_sp-PT_009142-RA TMHMM2.0 outside 367 370

Fusarium_sp-PT_009142-RA TMHMM2.0 TMhelix 371 393

Fusarium_sp-PT_009142-RA TMHMM2.0 inside 394 405

Fusarium_sp-PT_009142-RA TMHMM2.0 TMhelix 406 428

Fusarium_sp-PT_009142-RA TMHMM2.0 outside 429 437

Fusarium_sp-PT_009142-RA TMHMM2.0 TMhelix 438 460

Fusarium_sp-PT_009142-RA TMHMM2.0 inside 461 497

# Fusarium_sp-PT_009143-RA Length: 466

# Fusarium_sp-PT_009143-RA Number of predicted TMHs: 7

# Fusarium_sp-PT_009143-RA Exp number of AAs in TMHs: 149.66738

# Fusarium_sp-PT_009143-RA Exp number, first 60 AAs: 26.18177

# Fusarium_sp-PT_009143-RA Total prob of N-in: 0.01086

# Fusarium_sp-PT_009143-RA POSSIBLE N-term signal sequence

Fusarium_sp-PT_009143-RA TMHMM2.0 outside 1 27

Fusarium_sp-PT_009143-RA TMHMM2.0 TMhelix 28 50

Fusarium_sp-PT_009143-RA TMHMM2.0 inside 51 56

Fusarium_sp-PT_009143-RA TMHMM2.0 TMhelix 57 76

Fusarium_sp-PT_009143-RA TMHMM2.0 outside 77 102

Fusarium_sp-PT_009143-RA TMHMM2.0 TMhelix 103 120

Fusarium_sp-PT_009143-RA TMHMM2.0 inside 121 128

Fusarium_sp-PT_009143-RA TMHMM2.0 TMhelix 129 151

Fusarium_sp-PT_009143-RA TMHMM2.0 outside 152 181

Fusarium_sp-PT_009143-RA TMHMM2.0 TMhelix 182 204

Fusarium_sp-PT_009143-RA TMHMM2.0 inside 205 339

Fusarium_sp-PT_009143-RA TMHMM2.0 TMhelix 340 362

Fusarium_sp-PT_009143-RA TMHMM2.0 outside 363 381

Fusarium_sp-PT_009143-RA TMHMM2.0 TMhelix 382 404

Fusarium_sp-PT_009143-RA TMHMM2.0 inside 405 466

# Fusarium_sp-PT_009145-RA Length: 737

# Fusarium_sp-PT_009145-RA Number of predicted TMHs: 0

# Fusarium_sp-PT_009145-RA Exp number of AAs in TMHs: 0.04813

# Fusarium_sp-PT_009145-RA Exp number, first 60 AAs: 0

# Fusarium_sp-PT_009145-RA Total prob of N-in: 0.00057

Fusarium_sp-PT_009145-RA TMHMM2.0 outside 1 737

# Fusarium_sp-PT_009147-RA Length: 559

# Fusarium_sp-PT_009147-RA Number of predicted TMHs: 0

# Fusarium_sp-PT_009147-RA Exp number of AAs in TMHs: 0.00199

# Fusarium_sp-PT_009147-RA Exp number, first 60 AAs: 0

# Fusarium_sp-PT_009147-RA Total prob of N-in: 0.00009

Fusarium_sp-PT_009147-RA TMHMM2.0 outside 1 559

# Fusarium_sp-PT_009150-RA Length: 380

# Fusarium_sp-PT_009150-RA Number of predicted TMHs: 0

# Fusarium_sp-PT_009150-RA Exp number of AAs in TMHs: 0.00018

# Fusarium_sp-PT_009150-RA Exp number, first 60 AAs: 0

# Fusarium_sp-PT_009150-RA Total prob of N-in: 0.00487

Fusarium_sp-PT_009150-RA TMHMM2.0 outside 1 380

# Fusarium_sp-PT_009152-RA Length: 565

# Fusarium_sp-PT_009152-RA Number of predicted TMHs: 0

# Fusarium_sp-PT_009152-RA Exp number of AAs in TMHs: 0.002

# Fusarium_sp-PT_009152-RA Exp number, first 60 AAs: 0.00039

# Fusarium_sp-PT_009152-RA Total prob of N-in: 0.00099

Fusarium_sp-PT_009152-RA TMHMM2.0 outside 1 565

# Fusarium_sp-PT_009157-RA Length: 440

# Fusarium_sp-PT_009157-RA Number of predicted TMHs: 0

# Fusarium_sp-PT_009157-RA Exp number of AAs in TMHs: 0.00572999999999999

# Fusarium_sp-PT_009157-RA Exp number, first 60 AAs: 0

# Fusarium_sp-PT_009157-RA Total prob of N-in: 0.00120

Fusarium_sp-PT_009157-RA TMHMM2.0 outside 1 440

# Fusarium_sp-PT_009160-RA Length: 697

# Fusarium_sp-PT_009160-RA Number of predicted TMHs: 5

# Fusarium_sp-PT_009160-RA Exp number of AAs in TMHs: 145.74554

# Fusarium_sp-PT_009160-RA Exp number, first 60 AAs: 0.00058

# Fusarium_sp-PT_009160-RA Total prob of N-in: 0.16981

Fusarium_sp-PT_009160-RA TMHMM2.0 outside 1 290

Fusarium_sp-PT_009160-RA TMHMM2.0 TMhelix 291 310

Fusarium_sp-PT_009160-RA TMHMM2.0 inside 311 350

Fusarium_sp-PT_009160-RA TMHMM2.0 TMhelix 351 373

Fusarium_sp-PT_009160-RA TMHMM2.0 outside 374 387

Fusarium_sp-PT_009160-RA TMHMM2.0 TMhelix 388 410

Fusarium_sp-PT_009160-RA TMHMM2.0 inside 411 422

Fusarium_sp-PT_009160-RA TMHMM2.0 TMhelix 423 445

Fusarium_sp-PT_009160-RA TMHMM2.0 outside 446 483

Fusarium_sp-PT_009160-RA TMHMM2.0 TMhelix 484 506

Fusarium_sp-PT_009160-RA TMHMM2.0 inside 507 697

# Fusarium_sp-PT_009161-RA Length: 297

# Fusarium_sp-PT_009161-RA Number of predicted TMHs: 0

# Fusarium_sp-PT_009161-RA Exp number of AAs in TMHs: 92.00759

# Fusarium_sp-PT_009161-RA Exp number, first 60 AAs: 15.99497

# Fusarium_sp-PT_009161-RA Total prob of N-in: 0.28353

# Fusarium_sp-PT_009161-RA POSSIBLE N-term signal sequence

Fusarium_sp-PT_009161-RA TMHMM2.0 outside 1 297

# Fusarium_sp-PT_009164-RA Length: 645

# Fusarium_sp-PT_009164-RA Number of predicted TMHs: 0

# Fusarium_sp-PT_009164-RA Exp number of AAs in TMHs: 0.00037

# Fusarium_sp-PT_009164-RA Exp number, first 60 AAs: 0

# Fusarium_sp-PT_009164-RA Total prob of N-in: 0.00022

Fusarium_sp-PT_009164-RA TMHMM2.0 outside 1 645

# Fusarium_sp-PT_009138-RA Length: 900

# Fusarium_sp-PT_009138-RA Number of predicted TMHs: 0

# Fusarium_sp-PT_009138-RA Exp number of AAs in TMHs: 0.00018

# Fusarium_sp-PT_009138-RA Exp number, first 60 AAs: 0.00018

# Fusarium_sp-PT_009138-RA Total prob of N-in: 0.00002

Fusarium_sp-PT_009138-RA TMHMM2.0 outside 1 900

# Fusarium_sp-PT_009139-RA Length: 153

# Fusarium_sp-PT_009139-RA Number of predicted TMHs: 0

# Fusarium_sp-PT_009139-RA Exp number of AAs in TMHs: 0.0491900000000001

# Fusarium_sp-PT_009139-RA Exp number, first 60 AAs: 0.04672

# Fusarium_sp-PT_009139-RA Total prob of N-in: 0.27763

Fusarium_sp-PT_009139-RA TMHMM2.0 outside 1 153

# Fusarium_sp-PT_009144-RA Length: 284

# Fusarium_sp-PT_009144-RA Number of predicted TMHs: 0

# Fusarium_sp-PT_009144-RA Exp number of AAs in TMHs: 0.16639

# Fusarium_sp-PT_009144-RA Exp number, first 60 AAs: 0.15873

# Fusarium_sp-PT_009144-RA Total prob of N-in: 0.06485

Fusarium_sp-PT_009144-RA TMHMM2.0 outside 1 284

# Fusarium_sp-PT_009146-RA Length: 476

# Fusarium_sp-PT_009146-RA Number of predicted TMHs: 8

# Fusarium_sp-PT_009146-RA Exp number of AAs in TMHs: 169.61747

# Fusarium_sp-PT_009146-RA Exp number, first 60 AAs: 39.48253

# Fusarium_sp-PT_009146-RA Total prob of N-in: 0.93734

# Fusarium_sp-PT_009146-RA POSSIBLE N-term signal sequence

Fusarium_sp-PT_009146-RA TMHMM2.0 inside 1 6

Fusarium_sp-PT_009146-RA TMHMM2.0 TMhelix 7 24

Fusarium_sp-PT_009146-RA TMHMM2.0 outside 25 27

Fusarium_sp-PT_009146-RA TMHMM2.0 TMhelix 28 50

Fusarium_sp-PT_009146-RA TMHMM2.0 inside 51 62

Fusarium_sp-PT_009146-RA TMHMM2.0 TMhelix 63 85

Fusarium_sp-PT_009146-RA TMHMM2.0 outside 86 104

Fusarium_sp-PT_009146-RA TMHMM2.0 TMhelix 105 127

Fusarium_sp-PT_009146-RA TMHMM2.0 inside 128 164

Fusarium_sp-PT_009146-RA TMHMM2.0 TMhelix 165 184

Fusarium_sp-PT_009146-RA TMHMM2.0 outside 185 198

Fusarium_sp-PT_009146-RA TMHMM2.0 TMhelix 199 218

Fusarium_sp-PT_009146-RA TMHMM2.0 inside 219 229

Fusarium_sp-PT_009146-RA TMHMM2.0 TMhelix 230 252

Fusarium_sp-PT_009146-RA TMHMM2.0 outside 253 266

Fusarium_sp-PT_009146-RA TMHMM2.0 TMhelix 267 289

Fusarium_sp-PT_009146-RA TMHMM2.0 inside 290 476

# Fusarium_sp-PT_009148-RA Length: 394

# Fusarium_sp-PT_009148-RA Number of predicted TMHs: 0

# Fusarium_sp-PT_009148-RA Exp number of AAs in TMHs: 0.00732999999999999

# Fusarium_sp-PT_009148-RA Exp number, first 60 AAs: 0.00679

# Fusarium_sp-PT_009148-RA Total prob of N-in: 0.00517

Fusarium_sp-PT_009148-RA TMHMM2.0 outside 1 394

# Fusarium_sp-PT_009149-RA Length: 252

# Fusarium_sp-PT_009149-RA Number of predicted TMHs: 0

# Fusarium_sp-PT_009149-RA Exp number of AAs in TMHs: 0.000650000000000001

# Fusarium_sp-PT_009149-RA Exp number, first 60 AAs: 0.00023

# Fusarium_sp-PT_009149-RA Total prob of N-in: 0.00327

Fusarium_sp-PT_009149-RA TMHMM2.0 outside 1 252

# Fusarium_sp-PT_009151-RA Length: 553

# Fusarium_sp-PT_009151-RA Number of predicted TMHs: 0

# Fusarium_sp-PT_009151-RA Exp number of AAs in TMHs: 0.00569999999999997

# Fusarium_sp-PT_009151-RA Exp number, first 60 AAs: 0

# Fusarium_sp-PT_009151-RA Total prob of N-in: 0.00208

Fusarium_sp-PT_009151-RA TMHMM2.0 outside 1 553

# Fusarium_sp-PT_009153-RA Length: 307

# Fusarium_sp-PT_009153-RA Number of predicted TMHs: 0

# Fusarium_sp-PT_009153-RA Exp number of AAs in TMHs: 0.21938

# Fusarium_sp-PT_009153-RA Exp number, first 60 AAs: 0

# Fusarium_sp-PT_009153-RA Total prob of N-in: 0.01847

Fusarium_sp-PT_009153-RA TMHMM2.0 outside 1 307

# Fusarium_sp-PT_009154-RA Length: 1175

# Fusarium_sp-PT_009154-RA Number of predicted TMHs: 0

# Fusarium_sp-PT_009154-RA Exp number of AAs in TMHs: 0.01729

# Fusarium_sp-PT_009154-RA Exp number, first 60 AAs: 0.00092

# Fusarium_sp-PT_009154-RA Total prob of N-in: 0.00082

Fusarium_sp-PT_009154-RA TMHMM2.0 outside 1 1175

# Fusarium_sp-PT_009156-RA Length: 359

# Fusarium_sp-PT_009156-RA Number of predicted TMHs: 7

# Fusarium_sp-PT_009156-RA Exp number of AAs in TMHs: 152.41717

# Fusarium_sp-PT_009156-RA Exp number, first 60 AAs: 29.29437

# Fusarium_sp-PT_009156-RA Total prob of N-in: 0.04029

# Fusarium_sp-PT_009156-RA POSSIBLE N-term signal sequence

Fusarium_sp-PT_009156-RA TMHMM2.0 outside 1 19

Fusarium_sp-PT_009156-RA TMHMM2.0 TMhelix 20 38

Fusarium_sp-PT_009156-RA TMHMM2.0 inside 39 50

Fusarium_sp-PT_009156-RA TMHMM2.0 TMhelix 51 73

Fusarium_sp-PT_009156-RA TMHMM2.0 outside 74 102

Fusarium_sp-PT_009156-RA TMHMM2.0 TMhelix 103 122

Fusarium_sp-PT_009156-RA TMHMM2.0 inside 123 142

Fusarium_sp-PT_009156-RA TMHMM2.0 TMhelix 143 165

Fusarium_sp-PT_009156-RA TMHMM2.0 outside 166 169

Fusarium_sp-PT_009156-RA TMHMM2.0 TMhelix 170 192

Fusarium_sp-PT_009156-RA TMHMM2.0 inside 193 204

Fusarium_sp-PT_009156-RA TMHMM2.0 TMhelix 205 227

Fusarium_sp-PT_009156-RA TMHMM2.0 outside 228 236

Fusarium_sp-PT_009156-RA TMHMM2.0 TMhelix 237 259

Fusarium_sp-PT_009156-RA TMHMM2.0 inside 260 359

# Fusarium_sp-PT_009158-RA Length: 484

# Fusarium_sp-PT_009158-RA Number of predicted TMHs: 1

# Fusarium_sp-PT_009158-RA Exp number of AAs in TMHs: 23.6922

# Fusarium_sp-PT_009158-RA Exp number, first 60 AAs: 0.86871

# Fusarium_sp-PT_009158-RA Total prob of N-in: 0.06609

Fusarium_sp-PT_009158-RA TMHMM2.0 outside 1 306

Fusarium_sp-PT_009158-RA TMHMM2.0 TMhelix 307 329

Fusarium_sp-PT_009158-RA TMHMM2.0 inside 330 484

# Fusarium_sp-PT_009159-RA Length: 348

# Fusarium_sp-PT_009159-RA Number of predicted TMHs: 7

# Fusarium_sp-PT_009159-RA Exp number of AAs in TMHs: 149.14103

# Fusarium_sp-PT_009159-RA Exp number, first 60 AAs: 14.99308

# Fusarium_sp-PT_009159-RA Total prob of N-in: 0.08086

# Fusarium_sp-PT_009159-RA POSSIBLE N-term signal sequence

Fusarium_sp-PT_009159-RA TMHMM2.0 outside 1 46

Fusarium_sp-PT_009159-RA TMHMM2.0 TMhelix 47 69

Fusarium_sp-PT_009159-RA TMHMM2.0 inside 70 73

Fusarium_sp-PT_009159-RA TMHMM2.0 TMhelix 74 91

Fusarium_sp-PT_009159-RA TMHMM2.0 outside 92 105

Fusarium_sp-PT_009159-RA TMHMM2.0 TMhelix 106 128

Fusarium_sp-PT_009159-RA TMHMM2.0 inside 129 148

Fusarium_sp-PT_009159-RA TMHMM2.0 TMhelix 149 171

Fusarium_sp-PT_009159-RA TMHMM2.0 outside 172 185

Fusarium_sp-PT_009159-RA TMHMM2.0 TMhelix 186 208

Fusarium_sp-PT_009159-RA TMHMM2.0 inside 209 220

Fusarium_sp-PT_009159-RA TMHMM2.0 TMhelix 221 243

Fusarium_sp-PT_009159-RA TMHMM2.0 outside 244 257

Fusarium_sp-PT_009159-RA TMHMM2.0 TMhelix 258 280

Fusarium_sp-PT_009159-RA TMHMM2.0 inside 281 348

# Fusarium_sp-PT_009162-RA Length: 600

# Fusarium_sp-PT_009162-RA Number of predicted TMHs: 0

# Fusarium_sp-PT_009162-RA Exp number of AAs in TMHs: 0.0358

# Fusarium_sp-PT_009162-RA Exp number, first 60 AAs: 0

# Fusarium_sp-PT_009162-RA Total prob of N-in: 0.00319

Fusarium_sp-PT_009162-RA TMHMM2.0 outside 1 600

# Fusarium_sp-PT_009163-RA Length: 510

# Fusarium_sp-PT_009163-RA Number of predicted TMHs: 0

# Fusarium_sp-PT_009163-RA Exp number of AAs in TMHs: 0.02673

# Fusarium_sp-PT_009163-RA Exp number, first 60 AAs: 0.0109

# Fusarium_sp-PT_009163-RA Total prob of N-in: 0.00276

Fusarium_sp-PT_009163-RA TMHMM2.0 outside 1 510

# Fusarium_sp-PT_009165-RA Length: 192

# Fusarium_sp-PT_009165-RA Number of predicted TMHs: 0

# Fusarium_sp-PT_009165-RA Exp number of AAs in TMHs: 0.000940000000000001

# Fusarium_sp-PT_009165-RA Exp number, first 60 AAs: 0.00073

# Fusarium_sp-PT_009165-RA Total prob of N-in: 0.07128

Fusarium_sp-PT_009165-RA TMHMM2.0 outside 1 192

# Fusarium_sp-PT_009155-RA Length: 829

# Fusarium_sp-PT_009155-RA Number of predicted TMHs: 0

# Fusarium_sp-PT_009155-RA Exp number of AAs in TMHs: 0.18572

# Fusarium_sp-PT_009155-RA Exp number, first 60 AAs: 0.00254

# Fusarium_sp-PT_009155-RA Total prob of N-in: 0.00035

Fusarium_sp-PT_009155-RA TMHMM2.0 outside 1 829

# Fusarium_sp-PT_009166-RA Length: 265

# Fusarium_sp-PT_009166-RA Number of predicted TMHs: 0

# Fusarium_sp-PT_009166-RA Exp number of AAs in TMHs: 0.06448

# Fusarium_sp-PT_009166-RA Exp number, first 60 AAs: 0.0032

# Fusarium_sp-PT_009166-RA Total prob of N-in: 0.05850

Fusarium_sp-PT_009166-RA TMHMM2.0 outside 1 265

# Fusarium_sp-PT_009168-RA Length: 281

# Fusarium_sp-PT_009168-RA Number of predicted TMHs: 0

# Fusarium_sp-PT_009168-RA Exp number of AAs in TMHs: 25.50393

# Fusarium_sp-PT_009168-RA Exp number, first 60 AAs: 0

# Fusarium_sp-PT_009168-RA Total prob of N-in: 0.28475

Fusarium_sp-PT_009168-RA TMHMM2.0 outside 1 281

# Fusarium_sp-PT_009169-RA Length: 310

# Fusarium_sp-PT_009169-RA Number of predicted TMHs: 0

# Fusarium_sp-PT_009169-RA Exp number of AAs in TMHs: 0.00075

# Fusarium_sp-PT_009169-RA Exp number, first 60 AAs: 0

# Fusarium_sp-PT_009169-RA Total prob of N-in: 0.03561

Fusarium_sp-PT_009169-RA TMHMM2.0 outside 1 310

# Fusarium_sp-PT_009173-RA Length: 220

# Fusarium_sp-PT_009173-RA Number of predicted TMHs: 0

# Fusarium_sp-PT_009173-RA Exp number of AAs in TMHs: 0.02525

# Fusarium_sp-PT_009173-RA Exp number, first 60 AAs: 0.02474

# Fusarium_sp-PT_009173-RA Total prob of N-in: 0.18431

Fusarium_sp-PT_009173-RA TMHMM2.0 outside 1 220

# Fusarium_sp-PT_009174-RA Length: 1471

# Fusarium_sp-PT_009174-RA Number of predicted TMHs: 0

# Fusarium_sp-PT_009174-RA Exp number of AAs in TMHs: 0.0411500000000001

# Fusarium_sp-PT_009174-RA Exp number, first 60 AAs: 0.02151

# Fusarium_sp-PT_009174-RA Total prob of N-in: 0.00105

Fusarium_sp-PT_009174-RA TMHMM2.0 outside 1 1471

# Fusarium_sp-PT_009176-RA Length: 259

# Fusarium_sp-PT_009176-RA Number of predicted TMHs: 0

# Fusarium_sp-PT_009176-RA Exp number of AAs in TMHs: 0.48334

# Fusarium_sp-PT_009176-RA Exp number, first 60 AAs: 0.47075

# Fusarium_sp-PT_009176-RA Total prob of N-in: 0.01794

Fusarium_sp-PT_009176-RA TMHMM2.0 outside 1 259

# Fusarium_sp-PT_009178-RA Length: 552

# Fusarium_sp-PT_009178-RA Number of predicted TMHs: 12

# Fusarium_sp-PT_009178-RA Exp number of AAs in TMHs: 260.98299

# Fusarium_sp-PT_009178-RA Exp number, first 60 AAs: 12.64253

# Fusarium_sp-PT_009178-RA Total prob of N-in: 0.97461

# Fusarium_sp-PT_009178-RA POSSIBLE N-term signal sequence

Fusarium_sp-PT_009178-RA TMHMM2.0 inside 1 48

Fusarium_sp-PT_009178-RA TMHMM2.0 TMhelix 49 71

Fusarium_sp-PT_009178-RA TMHMM2.0 outside 72 75

Fusarium_sp-PT_009178-RA TMHMM2.0 TMhelix 76 98

Fusarium_sp-PT_009178-RA TMHMM2.0 inside 99 132

Fusarium_sp-PT_009178-RA TMHMM2.0 TMhelix 133 155

Fusarium_sp-PT_009178-RA TMHMM2.0 outside 156 174

Fusarium_sp-PT_009178-RA TMHMM2.0 TMhelix 175 194

Fusarium_sp-PT_009178-RA TMHMM2.0 inside 195 206

Fusarium_sp-PT_009178-RA TMHMM2.0 TMhelix 207 226

Fusarium_sp-PT_009178-RA TMHMM2.0 outside 227 240

Fusarium_sp-PT_009178-RA TMHMM2.0 TMhelix 241 263

Fusarium_sp-PT_009178-RA TMHMM2.0 inside 264 275

Fusarium_sp-PT_009178-RA TMHMM2.0 TMhelix 276 298

Fusarium_sp-PT_009178-RA TMHMM2.0 outside 299 331

Fusarium_sp-PT_009178-RA TMHMM2.0 TMhelix 332 354

Fusarium_sp-PT_009178-RA TMHMM2.0 inside 355 366

Fusarium_sp-PT_009178-RA TMHMM2.0 TMhelix 367 389

Fusarium_sp-PT_009178-RA TMHMM2.0 outside 390 398

Fusarium_sp-PT_009178-RA TMHMM2.0 TMhelix 399 421

Fusarium_sp-PT_009178-RA TMHMM2.0 inside 422 448

Fusarium_sp-PT_009178-RA TMHMM2.0 TMhelix 449 468

Fusarium_sp-PT_009178-RA TMHMM2.0 outside 469 477

Fusarium_sp-PT_009178-RA TMHMM2.0 TMhelix 478 500

Fusarium_sp-PT_009178-RA TMHMM2.0 inside 501 552

# Fusarium_sp-PT_009181-RA Length: 552

# Fusarium_sp-PT_009181-RA Number of predicted TMHs: 0

# Fusarium_sp-PT_009181-RA Exp number of AAs in TMHs: 0.11314

# Fusarium_sp-PT_009181-RA Exp number, first 60 AAs: 0.00015

# Fusarium_sp-PT_009181-RA Total prob of N-in: 0.00468

Fusarium_sp-PT_009181-RA TMHMM2.0 outside 1 552

# Fusarium_sp-PT_009182-RA Length: 425

# Fusarium_sp-PT_009182-RA Number of predicted TMHs: 0

# Fusarium_sp-PT_009182-RA Exp number of AAs in TMHs: 4.67386

# Fusarium_sp-PT_009182-RA Exp number, first 60 AAs: 0.06872

# Fusarium_sp-PT_009182-RA Total prob of N-in: 0.02820

Fusarium_sp-PT_009182-RA TMHMM2.0 outside 1 425

# Fusarium_sp-PT_009184-RA Length: 325

# Fusarium_sp-PT_009184-RA Number of predicted TMHs: 0

# Fusarium_sp-PT_009184-RA Exp number of AAs in TMHs: 0.01924

# Fusarium_sp-PT_009184-RA Exp number, first 60 AAs: 0.00141

# Fusarium_sp-PT_009184-RA Total prob of N-in: 0.00177

Fusarium_sp-PT_009184-RA TMHMM2.0 outside 1 325

# Fusarium_sp-PT_009186-RA Length: 427

# Fusarium_sp-PT_009186-RA Number of predicted TMHs: 1

# Fusarium_sp-PT_009186-RA Exp number of AAs in TMHs: 17.15312

# Fusarium_sp-PT_009186-RA Exp number, first 60 AAs: 0.00124

# Fusarium_sp-PT_009186-RA Total prob of N-in: 0.40147

Fusarium_sp-PT_009186-RA TMHMM2.0 outside 1 406

Fusarium_sp-PT_009186-RA TMHMM2.0 TMhelix 407 426

Fusarium_sp-PT_009186-RA TMHMM2.0 inside 427 427

# Fusarium_sp-PT_009187-RA Length: 333

# Fusarium_sp-PT_009187-RA Number of predicted TMHs: 0

# Fusarium_sp-PT_009187-RA Exp number of AAs in TMHs: 0.42695

# Fusarium_sp-PT_009187-RA Exp number, first 60 AAs: 0.00254

# Fusarium_sp-PT_009187-RA Total prob of N-in: 0.01908

Fusarium_sp-PT_009187-RA TMHMM2.0 outside 1 333

# Fusarium_sp-PT_009188-RA Length: 321

# Fusarium_sp-PT_009188-RA Number of predicted TMHs: 7

# Fusarium_sp-PT_009188-RA Exp number of AAs in TMHs: 151.17713

# Fusarium_sp-PT_009188-RA Exp number, first 60 AAs: 20.96373

# Fusarium_sp-PT_009188-RA Total prob of N-in: 0.08154

# Fusarium_sp-PT_009188-RA POSSIBLE N-term signal sequence

Fusarium_sp-PT_009188-RA TMHMM2.0 outside 1 35

Fusarium_sp-PT_009188-RA TMHMM2.0 TMhelix 36 58

Fusarium_sp-PT_009188-RA TMHMM2.0 inside 59 62

Fusarium_sp-PT_009188-RA TMHMM2.0 TMhelix 63 85

Fusarium_sp-PT_009188-RA TMHMM2.0 outside 86 94

Fusarium_sp-PT_009188-RA TMHMM2.0 TMhelix 95 117

Fusarium_sp-PT_009188-RA TMHMM2.0 inside 118 138

Fusarium_sp-PT_009188-RA TMHMM2.0 TMhelix 139 161

Fusarium_sp-PT_009188-RA TMHMM2.0 outside 162 175

Fusarium_sp-PT_009188-RA TMHMM2.0 TMhelix 176 198

Fusarium_sp-PT_009188-RA TMHMM2.0 inside 199 223

Fusarium_sp-PT_009188-RA TMHMM2.0 TMhelix 224 246

Fusarium_sp-PT_009188-RA TMHMM2.0 outside 247 265

Fusarium_sp-PT_009188-RA TMHMM2.0 TMhelix 266 288

Fusarium_sp-PT_009188-RA TMHMM2.0 inside 289 321

# Fusarium_sp-PT_009192-RA Length: 805

# Fusarium_sp-PT_009192-RA Number of predicted TMHs: 0

# Fusarium_sp-PT_009192-RA Exp number of AAs in TMHs: 0

# Fusarium_sp-PT_009192-RA Exp number, first 60 AAs: 0

# Fusarium_sp-PT_009192-RA Total prob of N-in: 0.00006

Fusarium_sp-PT_009192-RA TMHMM2.0 outside 1 805

# Fusarium_sp-PT_009193-RA Length: 142

# Fusarium_sp-PT_009193-RA Number of predicted TMHs: 0

# Fusarium_sp-PT_009193-RA Exp number of AAs in TMHs: 0.00242

# Fusarium_sp-PT_009193-RA Exp number, first 60 AAs: 0.0012

# Fusarium_sp-PT_009193-RA Total prob of N-in: 0.06710

Fusarium_sp-PT_009193-RA TMHMM2.0 outside 1 142

# Fusarium_sp-PT_009194-RA Length: 361

# Fusarium_sp-PT_009194-RA Number of predicted TMHs: 0

# Fusarium_sp-PT_009194-RA Exp number of AAs in TMHs: 7.75956999999998

# Fusarium_sp-PT_009194-RA Exp number, first 60 AAs: 7.64671

# Fusarium_sp-PT_009194-RA Total prob of N-in: 0.23943

Fusarium_sp-PT_009194-RA TMHMM2.0 outside 1 361

# Fusarium_sp-PT_009195-RA Length: 441

# Fusarium_sp-PT_009195-RA Number of predicted TMHs: 8

# Fusarium_sp-PT_009195-RA Exp number of AAs in TMHs: 192.31253

# Fusarium_sp-PT_009195-RA Exp number, first 60 AAs: 20.3868

# Fusarium_sp-PT_009195-RA Total prob of N-in: 0.46858

# Fusarium_sp-PT_009195-RA POSSIBLE N-term signal sequence

Fusarium_sp-PT_009195-RA TMHMM2.0 inside 1 28

Fusarium_sp-PT_009195-RA TMHMM2.0 TMhelix 29 51

Fusarium_sp-PT_009195-RA TMHMM2.0 outside 52 106

Fusarium_sp-PT_009195-RA TMHMM2.0 TMhelix 107 129

Fusarium_sp-PT_009195-RA TMHMM2.0 inside 130 149

Fusarium_sp-PT_009195-RA TMHMM2.0 TMhelix 150 172

Fusarium_sp-PT_009195-RA TMHMM2.0 outside 173 181

Fusarium_sp-PT_009195-RA TMHMM2.0 TMhelix 182 204

Fusarium_sp-PT_009195-RA TMHMM2.0 inside 205 210

Fusarium_sp-PT_009195-RA TMHMM2.0 TMhelix 211 233

Fusarium_sp-PT_009195-RA TMHMM2.0 outside 234 265

Fusarium_sp-PT_009195-RA TMHMM2.0 TMhelix 266 288

Fusarium_sp-PT_009195-RA TMHMM2.0 inside 289 359

Fusarium_sp-PT_009195-RA TMHMM2.0 TMhelix 360 382

Fusarium_sp-PT_009195-RA TMHMM2.0 outside 383 396

Fusarium_sp-PT_009195-RA TMHMM2.0 TMhelix 397 419

Fusarium_sp-PT_009195-RA TMHMM2.0 inside 420 441

# Fusarium_sp-PT_009197-RA Length: 492

# Fusarium_sp-PT_009197-RA Number of predicted TMHs: 11

# Fusarium_sp-PT_009197-RA Exp number of AAs in TMHs: 255.92564

# Fusarium_sp-PT_009197-RA Exp number, first 60 AAs: 0.00013

# Fusarium_sp-PT_009197-RA Total prob of N-in: 0.49103

Fusarium_sp-PT_009197-RA TMHMM2.0 outside 1 132

Fusarium_sp-PT_009197-RA TMHMM2.0 TMhelix 133 155

Fusarium_sp-PT_009197-RA TMHMM2.0 inside 156 167

Fusarium_sp-PT_009197-RA TMHMM2.0 TMhelix 168 190

Fusarium_sp-PT_009197-RA TMHMM2.0 outside 191 194

Fusarium_sp-PT_009197-RA TMHMM2.0 TMhelix 195 217

Fusarium_sp-PT_009197-RA TMHMM2.0 inside 218 223

Fusarium_sp-PT_009197-RA TMHMM2.0 TMhelix 224 246

Fusarium_sp-PT_009197-RA TMHMM2.0 outside 247 255

Fusarium_sp-PT_009197-RA TMHMM2.0 TMhelix 256 278

Fusarium_sp-PT_009197-RA TMHMM2.0 inside 279 309

Fusarium_sp-PT_009197-RA TMHMM2.0 TMhelix 310 332

Fusarium_sp-PT_009197-RA TMHMM2.0 outside 333 346

Fusarium_sp-PT_009197-RA TMHMM2.0 TMhelix 347 365

Fusarium_sp-PT_009197-RA TMHMM2.0 inside 366 376

Fusarium_sp-PT_009197-RA TMHMM2.0 TMhelix 377 399

Fusarium_sp-PT_009197-RA TMHMM2.0 outside 400 402

Fusarium_sp-PT_009197-RA TMHMM2.0 TMhelix 403 425

Fusarium_sp-PT_009197-RA TMHMM2.0 inside 426 431

Fusarium_sp-PT_009197-RA TMHMM2.0 TMhelix 432 454

Fusarium_sp-PT_009197-RA TMHMM2.0 outside 455 463

Fusarium_sp-PT_009197-RA TMHMM2.0 TMhelix 464 486

Fusarium_sp-PT_009197-RA TMHMM2.0 inside 487 492

# Fusarium_sp-PT_009199-RA Length: 507

# Fusarium_sp-PT_009199-RA Number of predicted TMHs: 2

# Fusarium_sp-PT_009199-RA Exp number of AAs in TMHs: 44.14565

# Fusarium_sp-PT_009199-RA Exp number, first 60 AAs: 0.00418

# Fusarium_sp-PT_009199-RA Total prob of N-in: 0.01870

Fusarium_sp-PT_009199-RA TMHMM2.0 outside 1 315

Fusarium_sp-PT_009199-RA TMHMM2.0 TMhelix 316 338

Fusarium_sp-PT_009199-RA TMHMM2.0 inside 339 481

Fusarium_sp-PT_009199-RA TMHMM2.0 TMhelix 482 504

Fusarium_sp-PT_009199-RA TMHMM2.0 outside 505 507

# Fusarium_sp-PT_009200-RA Length: 939

# Fusarium_sp-PT_009200-RA Number of predicted TMHs: 0

# Fusarium_sp-PT_009200-RA Exp number of AAs in TMHs: 0.02102

# Fusarium_sp-PT_009200-RA Exp number, first 60 AAs: 0

# Fusarium_sp-PT_009200-RA Total prob of N-in: 0.00002

Fusarium_sp-PT_009200-RA TMHMM2.0 outside 1 939

# Fusarium_sp-PT_009167-RA Length: 72

# Fusarium_sp-PT_009167-RA Number of predicted TMHs: 1

# Fusarium_sp-PT_009167-RA Exp number of AAs in TMHs: 20.86438

# Fusarium_sp-PT_009167-RA Exp number, first 60 AAs: 20.86438

# Fusarium_sp-PT_009167-RA Total prob of N-in: 0.20350

# Fusarium_sp-PT_009167-RA POSSIBLE N-term signal sequence

Fusarium_sp-PT_009167-RA TMHMM2.0 outside 1 14

Fusarium_sp-PT_009167-RA TMHMM2.0 TMhelix 15 37

Fusarium_sp-PT_009167-RA TMHMM2.0 inside 38 72

# Fusarium_sp-PT_009170-RA Length: 666

# Fusarium_sp-PT_009170-RA Number of predicted TMHs: 0

# Fusarium_sp-PT_009170-RA Exp number of AAs in TMHs: 0.00295

# Fusarium_sp-PT_009170-RA Exp number, first 60 AAs: 0

# Fusarium_sp-PT_009170-RA Total prob of N-in: 0.00062

Fusarium_sp-PT_009170-RA TMHMM2.0 outside 1 666

# Fusarium_sp-PT_009171-RA Length: 1046

# Fusarium_sp-PT_009171-RA Number of predicted TMHs: 0

# Fusarium_sp-PT_009171-RA Exp number of AAs in TMHs: 0.00055

# Fusarium_sp-PT_009171-RA Exp number, first 60 AAs: 0

# Fusarium_sp-PT_009171-RA Total prob of N-in: 0.00003

Fusarium_sp-PT_009171-RA TMHMM2.0 outside 1 1046

# Fusarium_sp-PT_009172-RA Length: 129

# Fusarium_sp-PT_009172-RA Number of predicted TMHs: 0

# Fusarium_sp-PT_009172-RA Exp number of AAs in TMHs: 0

# Fusarium_sp-PT_009172-RA Exp number, first 60 AAs: 0

# Fusarium_sp-PT_009172-RA Total prob of N-in: 0.34600

Fusarium_sp-PT_009172-RA TMHMM2.0 outside 1 129

# Fusarium_sp-PT_009175-RA Length: 235

# Fusarium_sp-PT_009175-RA Number of predicted TMHs: 0

# Fusarium_sp-PT_009175-RA Exp number of AAs in TMHs: 5.01994999999999

# Fusarium_sp-PT_009175-RA Exp number, first 60 AAs: 5.01053

# Fusarium_sp-PT_009175-RA Total prob of N-in: 0.09384

Fusarium_sp-PT_009175-RA TMHMM2.0 outside 1 235

# Fusarium_sp-PT_009177-RA Length: 1061

# Fusarium_sp-PT_009177-RA Number of predicted TMHs: 0

# Fusarium_sp-PT_009177-RA Exp number of AAs in TMHs: 0.0471500000000002

# Fusarium_sp-PT_009177-RA Exp number, first 60 AAs: 0.00141

# Fusarium_sp-PT_009177-RA Total prob of N-in: 0.00011

Fusarium_sp-PT_009177-RA TMHMM2.0 outside 1 1061

# Fusarium_sp-PT_009183-RA Length: 575

# Fusarium_sp-PT_009183-RA Number of predicted TMHs: 0

# Fusarium_sp-PT_009183-RA Exp number of AAs in TMHs: 26.7786

# Fusarium_sp-PT_009183-RA Exp number, first 60 AAs: 0

# Fusarium_sp-PT_009183-RA Total prob of N-in: 0.26400

Fusarium_sp-PT_009183-RA TMHMM2.0 outside 1 575

# Fusarium_sp-PT_009185-RA Length: 198

# Fusarium_sp-PT_009185-RA Number of predicted TMHs: 0

# Fusarium_sp-PT_009185-RA Exp number of AAs in TMHs: 0.00033

# Fusarium_sp-PT_009185-RA Exp number, first 60 AAs: 0

# Fusarium_sp-PT_009185-RA Total prob of N-in: 0.10100

Fusarium_sp-PT_009185-RA TMHMM2.0 outside 1 198

# Fusarium_sp-PT_009189-RA Length: 778

# Fusarium_sp-PT_009189-RA Number of predicted TMHs: 0

# Fusarium_sp-PT_009189-RA Exp number of AAs in TMHs: 0.01732

# Fusarium_sp-PT_009189-RA Exp number, first 60 AAs: 0

# Fusarium_sp-PT_009189-RA Total prob of N-in: 0.00034

Fusarium_sp-PT_009189-RA TMHMM2.0 outside 1 778

# Fusarium_sp-PT_009191-RA Length: 462

# Fusarium_sp-PT_009191-RA Number of predicted TMHs: 0

# Fusarium_sp-PT_009191-RA Exp number of AAs in TMHs: 0.00252

# Fusarium_sp-PT_009191-RA Exp number, first 60 AAs: 0.00132

# Fusarium_sp-PT_009191-RA Total prob of N-in: 0.00545

Fusarium_sp-PT_009191-RA TMHMM2.0 outside 1 462

# Fusarium_sp-PT_009196-RA Length: 136

# Fusarium_sp-PT_009196-RA Number of predicted TMHs: 0

# Fusarium_sp-PT_009196-RA Exp number of AAs in TMHs: 0

# Fusarium_sp-PT_009196-RA Exp number, first 60 AAs: 0

# Fusarium_sp-PT_009196-RA Total prob of N-in: 0.25262

Fusarium_sp-PT_009196-RA TMHMM2.0 outside 1 136

# Fusarium_sp-PT_009198-RA Length: 249

# Fusarium_sp-PT_009198-RA Number of predicted TMHs: 0

# Fusarium_sp-PT_009198-RA Exp number of AAs in TMHs: 0.00661

# Fusarium_sp-PT_009198-RA Exp number, first 60 AAs: 0.00031

# Fusarium_sp-PT_009198-RA Total prob of N-in: 0.06821

Fusarium_sp-PT_009198-RA TMHMM2.0 outside 1 249

# Fusarium_sp-PT_009190-RA Length: 227

# Fusarium_sp-PT_009190-RA Number of predicted TMHs: 0

# Fusarium_sp-PT_009190-RA Exp number of AAs in TMHs: 0.10263

# Fusarium_sp-PT_009190-RA Exp number, first 60 AAs: 0.09814

# Fusarium_sp-PT_009190-RA Total prob of N-in: 0.01891

Fusarium_sp-PT_009190-RA TMHMM2.0 outside 1 227

# Fusarium_sp-PT_009202-RA Length: 183

# Fusarium_sp-PT_009202-RA Number of predicted TMHs: 0

# Fusarium_sp-PT_009202-RA Exp number of AAs in TMHs: 0.00481

# Fusarium_sp-PT_009202-RA Exp number, first 60 AAs: 0.00069

# Fusarium_sp-PT_009202-RA Total prob of N-in: 0.03277

Fusarium_sp-PT_009202-RA TMHMM2.0 outside 1 183

# Fusarium_sp-PT_009204-RA Length: 816

# Fusarium_sp-PT_009204-RA Number of predicted TMHs: 0

# Fusarium_sp-PT_009204-RA Exp number of AAs in TMHs: 0.03719

# Fusarium_sp-PT_009204-RA Exp number, first 60 AAs: 0

# Fusarium_sp-PT_009204-RA Total prob of N-in: 0.00002

Fusarium_sp-PT_009204-RA TMHMM2.0 outside 1 816

# Fusarium_sp-PT_009207-RA Length: 640

# Fusarium_sp-PT_009207-RA Number of predicted TMHs: 10

# Fusarium_sp-PT_009207-RA Exp number of AAs in TMHs: 219.6736

# Fusarium_sp-PT_009207-RA Exp number, first 60 AAs: 13.81745

# Fusarium_sp-PT_009207-RA Total prob of N-in: 0.21012

# Fusarium_sp-PT_009207-RA POSSIBLE N-term signal sequence

Fusarium_sp-PT_009207-RA TMHMM2.0 outside 1 9

Fusarium_sp-PT_009207-RA TMHMM2.0 TMhelix 10 32

Fusarium_sp-PT_009207-RA TMHMM2.0 inside 33 265

Fusarium_sp-PT_009207-RA TMHMM2.0 TMhelix 266 288

Fusarium_sp-PT_009207-RA TMHMM2.0 outside 289 344

Fusarium_sp-PT_009207-RA TMHMM2.0 TMhelix 345 367

Fusarium_sp-PT_009207-RA TMHMM2.0 inside 368 373

Fusarium_sp-PT_009207-RA TMHMM2.0 TMhelix 374 396

Fusarium_sp-PT_009207-RA TMHMM2.0 outside 397 410

Fusarium_sp-PT_009207-RA TMHMM2.0 TMhelix 411 433

Fusarium_sp-PT_009207-RA TMHMM2.0 inside 434 439

Fusarium_sp-PT_009207-RA TMHMM2.0 TMhelix 440 462

Fusarium_sp-PT_009207-RA TMHMM2.0 outside 463 497

Fusarium_sp-PT_009207-RA TMHMM2.0 TMhelix 498 520

Fusarium_sp-PT_009207-RA TMHMM2.0 inside 521 532

Fusarium_sp-PT_009207-RA TMHMM2.0 TMhelix 533 555

Fusarium_sp-PT_009207-RA TMHMM2.0 outside 556 569

Fusarium_sp-PT_009207-RA TMHMM2.0 TMhelix 570 589

Fusarium_sp-PT_009207-RA TMHMM2.0 inside 590 601

Fusarium_sp-PT_009207-RA TMHMM2.0 TMhelix 602 624

Fusarium_sp-PT_009207-RA TMHMM2.0 outside 625 640

# Fusarium_sp-PT_009209-RA Length: 720

# Fusarium_sp-PT_009209-RA Number of predicted TMHs: 9

# Fusarium_sp-PT_009209-RA Exp number of AAs in TMHs: 230.97487

# Fusarium_sp-PT_009209-RA Exp number, first 60 AAs: 39.35842

# Fusarium_sp-PT_009209-RA Total prob of N-in: 0.00916

# Fusarium_sp-PT_009209-RA POSSIBLE N-term signal sequence

Fusarium_sp-PT_009209-RA TMHMM2.0 outside 1 3

Fusarium_sp-PT_009209-RA TMHMM2.0 TMhelix 4 21

Fusarium_sp-PT_009209-RA TMHMM2.0 inside 22 32

Fusarium_sp-PT_009209-RA TMHMM2.0 TMhelix 33 52

Fusarium_sp-PT_009209-RA TMHMM2.0 outside 53 172

Fusarium_sp-PT_009209-RA TMHMM2.0 TMhelix 173 195

Fusarium_sp-PT_009209-RA TMHMM2.0 inside 196 214

Fusarium_sp-PT_009209-RA TMHMM2.0 TMhelix 215 237

Fusarium_sp-PT_009209-RA TMHMM2.0 outside 238 283

Fusarium_sp-PT_009209-RA TMHMM2.0 TMhelix 284 306

Fusarium_sp-PT_009209-RA TMHMM2.0 inside 307 312

Fusarium_sp-PT_009209-RA TMHMM2.0 TMhelix 313 335

Fusarium_sp-PT_009209-RA TMHMM2.0 outside 336 349

Fusarium_sp-PT_009209-RA TMHMM2.0 TMhelix 350 372

Fusarium_sp-PT_009209-RA TMHMM2.0 inside 373 446

Fusarium_sp-PT_009209-RA TMHMM2.0 TMhelix 447 469

Fusarium_sp-PT_009209-RA TMHMM2.0 outside 470 676

Fusarium_sp-PT_009209-RA TMHMM2.0 TMhelix 677 696

Fusarium_sp-PT_009209-RA TMHMM2.0 inside 697 720

# Fusarium_sp-PT_009211-RA Length: 301

# Fusarium_sp-PT_009211-RA Number of predicted TMHs: 0

# Fusarium_sp-PT_009211-RA Exp number of AAs in TMHs: 0.26343

# Fusarium_sp-PT_009211-RA Exp number, first 60 AAs: 0.18723

# Fusarium_sp-PT_009211-RA Total prob of N-in: 0.03879

Fusarium_sp-PT_009211-RA TMHMM2.0 outside 1 301

# Fusarium_sp-PT_009212-RA Length: 503

# Fusarium_sp-PT_009212-RA Number of predicted TMHs: 1

# Fusarium_sp-PT_009212-RA Exp number of AAs in TMHs: 25.2643

# Fusarium_sp-PT_009212-RA Exp number, first 60 AAs: 25.10908

# Fusarium_sp-PT_009212-RA Total prob of N-in: 0.91112

# Fusarium_sp-PT_009212-RA POSSIBLE N-term signal sequence

Fusarium_sp-PT_009212-RA TMHMM2.0 inside 1 12

Fusarium_sp-PT_009212-RA TMHMM2.0 TMhelix 13 35

Fusarium_sp-PT_009212-RA TMHMM2.0 outside 36 503

# Fusarium_sp-PT_009213-RA Length: 270

# Fusarium_sp-PT_009213-RA Number of predicted TMHs: 0

# Fusarium_sp-PT_009213-RA Exp number of AAs in TMHs: 0.0026

# Fusarium_sp-PT_009213-RA Exp number, first 60 AAs: 0.00204

# Fusarium_sp-PT_009213-RA Total prob of N-in: 0.02913

Fusarium_sp-PT_009213-RA TMHMM2.0 outside 1 270

# Fusarium_sp-PT_009216-RA Length: 318

# Fusarium_sp-PT_009216-RA Number of predicted TMHs: 1

# Fusarium_sp-PT_009216-RA Exp number of AAs in TMHs: 21.07895

# Fusarium_sp-PT_009216-RA Exp number, first 60 AAs: 0

# Fusarium_sp-PT_009216-RA Total prob of N-in: 0.04751

Fusarium_sp-PT_009216-RA TMHMM2.0 outside 1 178

Fusarium_sp-PT_009216-RA TMHMM2.0 TMhelix 179 198

Fusarium_sp-PT_009216-RA TMHMM2.0 inside 199 318

# Fusarium_sp-PT_009217-RA Length: 157

# Fusarium_sp-PT_009217-RA Number of predicted TMHs: 0

# Fusarium_sp-PT_009217-RA Exp number of AAs in TMHs: 0.00018

# Fusarium_sp-PT_009217-RA Exp number, first 60 AAs: 0

# Fusarium_sp-PT_009217-RA Total prob of N-in: 0.32544

Fusarium_sp-PT_009217-RA TMHMM2.0 outside 1 157

# Fusarium_sp-PT_009218-RA Length: 567

# Fusarium_sp-PT_009218-RA Number of predicted TMHs: 0

# Fusarium_sp-PT_009218-RA Exp number of AAs in TMHs: 5.31263999999999

# Fusarium_sp-PT_009218-RA Exp number, first 60 AAs: 0

# Fusarium_sp-PT_009218-RA Total prob of N-in: 0.02795

Fusarium_sp-PT_009218-RA TMHMM2.0 outside 1 567

# Fusarium_sp-PT_009220-RA Length: 469

# Fusarium_sp-PT_009220-RA Number of predicted TMHs: 0

# Fusarium_sp-PT_009220-RA Exp number of AAs in TMHs: 13.08711

# Fusarium_sp-PT_009220-RA Exp number, first 60 AAs: 6.05891

# Fusarium_sp-PT_009220-RA Total prob of N-in: 0.43208

Fusarium_sp-PT_009220-RA TMHMM2.0 outside 1 469

# Fusarium_sp-PT_009222-RA Length: 538

# Fusarium_sp-PT_009222-RA Number of predicted TMHs: 0

# Fusarium_sp-PT_009222-RA Exp number of AAs in TMHs: 0.00113

# Fusarium_sp-PT_009222-RA Exp number, first 60 AAs: 0

# Fusarium_sp-PT_009222-RA Total prob of N-in: 0.00066

Fusarium_sp-PT_009222-RA TMHMM2.0 outside 1 538

# Fusarium_sp-PT_009224-RA Length: 231

# Fusarium_sp-PT_009224-RA Number of predicted TMHs: 0

# Fusarium_sp-PT_009224-RA Exp number of AAs in TMHs: 0

# Fusarium_sp-PT_009224-RA Exp number, first 60 AAs: 0

# Fusarium_sp-PT_009224-RA Total prob of N-in: 0.30362

Fusarium_sp-PT_009224-RA TMHMM2.0 outside 1 231

# Fusarium_sp-PT_009225-RA Length: 718

# Fusarium_sp-PT_009225-RA Number of predicted TMHs: 0

# Fusarium_sp-PT_009225-RA Exp number of AAs in TMHs: 0.00251

# Fusarium_sp-PT_009225-RA Exp number, first 60 AAs: 0

# Fusarium_sp-PT_009225-RA Total prob of N-in: 0.00023

Fusarium_sp-PT_009225-RA TMHMM2.0 outside 1 718

# Fusarium_sp-PT_009230-RA Length: 611

# Fusarium_sp-PT_009230-RA Number of predicted TMHs: 0

# Fusarium_sp-PT_009230-RA Exp number of AAs in TMHs: 0.02253

# Fusarium_sp-PT_009230-RA Exp number, first 60 AAs: 0

# Fusarium_sp-PT_009230-RA Total prob of N-in: 0.00127

Fusarium_sp-PT_009230-RA TMHMM2.0 outside 1 611

# Fusarium_sp-PT_009232-RA Length: 480

# Fusarium_sp-PT_009232-RA Number of predicted TMHs: 7

# Fusarium_sp-PT_009232-RA Exp number of AAs in TMHs: 149.67851

# Fusarium_sp-PT_009232-RA Exp number, first 60 AAs: 32.28904

# Fusarium_sp-PT_009232-RA Total prob of N-in: 0.00177

# Fusarium_sp-PT_009232-RA POSSIBLE N-term signal sequence

Fusarium_sp-PT_009232-RA TMHMM2.0 outside 1 19

Fusarium_sp-PT_009232-RA TMHMM2.0 TMhelix 20 42

Fusarium_sp-PT_009232-RA TMHMM2.0 inside 43 48

Fusarium_sp-PT_009232-RA TMHMM2.0 TMhelix 49 71

Fusarium_sp-PT_009232-RA TMHMM2.0 outside 72 98

Fusarium_sp-PT_009232-RA TMHMM2.0 TMhelix 99 116

Fusarium_sp-PT_009232-RA TMHMM2.0 inside 117 124

Fusarium_sp-PT_009232-RA TMHMM2.0 TMhelix 125 147

Fusarium_sp-PT_009232-RA TMHMM2.0 outside 148 177

Fusarium_sp-PT_009232-RA TMHMM2.0 TMhelix 178 200

Fusarium_sp-PT_009232-RA TMHMM2.0 inside 201 361

Fusarium_sp-PT_009232-RA TMHMM2.0 TMhelix 362 381

Fusarium_sp-PT_009232-RA TMHMM2.0 outside 382 395

Fusarium_sp-PT_009232-RA TMHMM2.0 TMhelix 396 418

Fusarium_sp-PT_009232-RA TMHMM2.0 inside 419 480

# Fusarium_sp-PT_009234-RA Length: 750

# Fusarium_sp-PT_009234-RA Number of predicted TMHs: 0

# Fusarium_sp-PT_009234-RA Exp number of AAs in TMHs: 0.68972

# Fusarium_sp-PT_009234-RA Exp number, first 60 AAs: 0.68863

# Fusarium_sp-PT_009234-RA Total prob of N-in: 0.03264

Fusarium_sp-PT_009234-RA TMHMM2.0 outside 1 750

# Fusarium_sp-PT_009235-RA Length: 348

# Fusarium_sp-PT_009235-RA Number of predicted TMHs: 0

# Fusarium_sp-PT_009235-RA Exp number of AAs in TMHs: 0.20144

# Fusarium_sp-PT_009235-RA Exp number, first 60 AAs: 0.000710000000000001

# Fusarium_sp-PT_009235-RA Total prob of N-in: 0.01663

Fusarium_sp-PT_009235-RA TMHMM2.0 outside 1 348

# Fusarium_sp-PT_009201-RA Length: 446

# Fusarium_sp-PT_009201-RA Number of predicted TMHs: 0

# Fusarium_sp-PT_009201-RA Exp number of AAs in TMHs: 0.0760800000000001

# Fusarium_sp-PT_009201-RA Exp number, first 60 AAs: 0

# Fusarium_sp-PT_009201-RA Total prob of N-in: 0.00882

Fusarium_sp-PT_009201-RA TMHMM2.0 outside 1 446

# Fusarium_sp-PT_009203-RA Length: 472

# Fusarium_sp-PT_009203-RA Number of predicted TMHs: 1

# Fusarium_sp-PT_009203-RA Exp number of AAs in TMHs: 18.83799

# Fusarium_sp-PT_009203-RA Exp number, first 60 AAs: 18.83054

# Fusarium_sp-PT_009203-RA Total prob of N-in: 0.98094

# Fusarium_sp-PT_009203-RA POSSIBLE N-term signal sequence

Fusarium_sp-PT_009203-RA TMHMM2.0 inside 1 6

Fusarium_sp-PT_009203-RA TMHMM2.0 TMhelix 7 26

Fusarium_sp-PT_009203-RA TMHMM2.0 outside 27 472

# Fusarium_sp-PT_009205-RA Length: 410

# Fusarium_sp-PT_009205-RA Number of predicted TMHs: 0

# Fusarium_sp-PT_009205-RA Exp number of AAs in TMHs: 0.00535

# Fusarium_sp-PT_009205-RA Exp number, first 60 AAs: 0.00066

# Fusarium_sp-PT_009205-RA Total prob of N-in: 0.00460

Fusarium_sp-PT_009205-RA TMHMM2.0 outside 1 410

# Fusarium_sp-PT_009206-RA Length: 361

# Fusarium_sp-PT_009206-RA Number of predicted TMHs: 0

# Fusarium_sp-PT_009206-RA Exp number of AAs in TMHs: 0.03012

# Fusarium_sp-PT_009206-RA Exp number, first 60 AAs: 0.02536

# Fusarium_sp-PT_009206-RA Total prob of N-in: 0.00824

Fusarium_sp-PT_009206-RA TMHMM2.0 outside 1 361

# Fusarium_sp-PT_009208-RA Length: 343

# Fusarium_sp-PT_009208-RA Number of predicted TMHs: 0

# Fusarium_sp-PT_009208-RA Exp number of AAs in TMHs: 0.01704

# Fusarium_sp-PT_009208-RA Exp number, first 60 AAs: 0

# Fusarium_sp-PT_009208-RA Total prob of N-in: 0.01800

Fusarium_sp-PT_009208-RA TMHMM2.0 outside 1 343

# Fusarium_sp-PT_009210-RA Length: 175

# Fusarium_sp-PT_009210-RA Number of predicted TMHs: 0

# Fusarium_sp-PT_009210-RA Exp number of AAs in TMHs: 0.00684

# Fusarium_sp-PT_009210-RA Exp number, first 60 AAs: 0.0061

# Fusarium_sp-PT_009210-RA Total prob of N-in: 0.03705

Fusarium_sp-PT_009210-RA TMHMM2.0 outside 1 175

# Fusarium_sp-PT_009214-RA Length: 617

# Fusarium_sp-PT_009214-RA Number of predicted TMHs: 9

# Fusarium_sp-PT_009214-RA Exp number of AAs in TMHs: 197.56379

# Fusarium_sp-PT_009214-RA Exp number, first 60 AAs: 22.03756

# Fusarium_sp-PT_009214-RA Total prob of N-in: 0.01457

# Fusarium_sp-PT_009214-RA POSSIBLE N-term signal sequence

Fusarium_sp-PT_009214-RA TMHMM2.0 outside 1 25

Fusarium_sp-PT_009214-RA TMHMM2.0 TMhelix 26 48

Fusarium_sp-PT_009214-RA TMHMM2.0 inside 49 68

Fusarium_sp-PT_009214-RA TMHMM2.0 TMhelix 69 91

Fusarium_sp-PT_009214-RA TMHMM2.0 outside 92 94

Fusarium_sp-PT_009214-RA TMHMM2.0 TMhelix 95 117

Fusarium_sp-PT_009214-RA TMHMM2.0 inside 118 123

Fusarium_sp-PT_009214-RA TMHMM2.0 TMhelix 124 143

Fusarium_sp-PT_009214-RA TMHMM2.0 outside 144 157

Fusarium_sp-PT_009214-RA TMHMM2.0 TMhelix 158 180

Fusarium_sp-PT_009214-RA TMHMM2.0 inside 181 191

Fusarium_sp-PT_009214-RA TMHMM2.0 TMhelix 192 214

Fusarium_sp-PT_009214-RA TMHMM2.0 outside 215 228

Fusarium_sp-PT_009214-RA TMHMM2.0 TMhelix 229 249

Fusarium_sp-PT_009214-RA TMHMM2.0 inside 250 253

Fusarium_sp-PT_009214-RA TMHMM2.0 TMhelix 254 276

Fusarium_sp-PT_009214-RA TMHMM2.0 outside 277 285

Fusarium_sp-PT_009214-RA TMHMM2.0 TMhelix 286 308

Fusarium_sp-PT_009214-RA TMHMM2.0 inside 309 617

# Fusarium_sp-PT_009215-RA Length: 371

# Fusarium_sp-PT_009215-RA Number of predicted TMHs: 0

# Fusarium_sp-PT_009215-RA Exp number of AAs in TMHs: 0.00017

# Fusarium_sp-PT_009215-RA Exp number, first 60 AAs: 0

# Fusarium_sp-PT_009215-RA Total prob of N-in: 0.00369

Fusarium_sp-PT_009215-RA TMHMM2.0 outside 1 371

# Fusarium_sp-PT_009219-RA Length: 304

# Fusarium_sp-PT_009219-RA Number of predicted TMHs: 0

# Fusarium_sp-PT_009219-RA Exp number of AAs in TMHs: 0.50238

# Fusarium_sp-PT_009219-RA Exp number, first 60 AAs: 0.00819999999999999

# Fusarium_sp-PT_009219-RA Total prob of N-in: 0.04845

Fusarium_sp-PT_009219-RA TMHMM2.0 outside 1 304

# Fusarium_sp-PT_009221-RA Length: 185

# Fusarium_sp-PT_009221-RA Number of predicted TMHs: 1

# Fusarium_sp-PT_009221-RA Exp number of AAs in TMHs: 21.99415

# Fusarium_sp-PT_009221-RA Exp number, first 60 AAs: 0.00772

# Fusarium_sp-PT_009221-RA Total prob of N-in: 0.98721

Fusarium_sp-PT_009221-RA TMHMM2.0 inside 1 152

Fusarium_sp-PT_009221-RA TMHMM2.0 TMhelix 153 175

Fusarium_sp-PT_009221-RA TMHMM2.0 outside 176 185

# Fusarium_sp-PT_009223-RA Length: 415

# Fusarium_sp-PT_009223-RA Number of predicted TMHs: 0

# Fusarium_sp-PT_009223-RA Exp number of AAs in TMHs: 0.01174

# Fusarium_sp-PT_009223-RA Exp number, first 60 AAs: 0.00651

# Fusarium_sp-PT_009223-RA Total prob of N-in: 0.01167

Fusarium_sp-PT_009223-RA TMHMM2.0 outside 1 415

# Fusarium_sp-PT_009226-RA Length: 782

# Fusarium_sp-PT_009226-RA Number of predicted TMHs: 0

# Fusarium_sp-PT_009226-RA Exp number of AAs in TMHs: 0.00042

# Fusarium_sp-PT_009226-RA Exp number, first 60 AAs: 0

# Fusarium_sp-PT_009226-RA Total prob of N-in: 0.00005

Fusarium_sp-PT_009226-RA TMHMM2.0 outside 1 782

# Fusarium_sp-PT_009227-RA Length: 391

# Fusarium_sp-PT_009227-RA Number of predicted TMHs: 0

# Fusarium_sp-PT_009227-RA Exp number of AAs in TMHs: 0.24329

# Fusarium_sp-PT_009227-RA Exp number, first 60 AAs: 0.00364

# Fusarium_sp-PT_009227-RA Total prob of N-in: 0.05276

Fusarium_sp-PT_009227-RA TMHMM2.0 outside 1 391

# Fusarium_sp-PT_009228-RA Length: 1034

# Fusarium_sp-PT_009228-RA Number of predicted TMHs: 0

# Fusarium_sp-PT_009228-RA Exp number of AAs in TMHs: 19.56624

# Fusarium_sp-PT_009228-RA Exp number, first 60 AAs: 0.34005

# Fusarium_sp-PT_009228-RA Total prob of N-in: 0.04873

Fusarium_sp-PT_009228-RA TMHMM2.0 outside 1 1034

# Fusarium_sp-PT_009229-RA Length: 148

# Fusarium_sp-PT_009229-RA Number of predicted TMHs: 0

# Fusarium_sp-PT_009229-RA Exp number of AAs in TMHs: 0.00273

# Fusarium_sp-PT_009229-RA Exp number, first 60 AAs: 0.00017

# Fusarium_sp-PT_009229-RA Total prob of N-in: 0.03294

Fusarium_sp-PT_009229-RA TMHMM2.0 outside 1 148

# Fusarium_sp-PT_009231-RA Length: 270

# Fusarium_sp-PT_009231-RA Number of predicted TMHs: 0

# Fusarium_sp-PT_009231-RA Exp number of AAs in TMHs: 0.15649

# Fusarium_sp-PT_009231-RA Exp number, first 60 AAs: 0.01886

# Fusarium_sp-PT_009231-RA Total prob of N-in: 0.03820

Fusarium_sp-PT_009231-RA TMHMM2.0 outside 1 270

# Fusarium_sp-PT_009233-RA Length: 265

# Fusarium_sp-PT_009233-RA Number of predicted TMHs: 0

# Fusarium_sp-PT_009233-RA Exp number of AAs in TMHs: 1.02545

# Fusarium_sp-PT_009233-RA Exp number, first 60 AAs: 0.20849

# Fusarium_sp-PT_009233-RA Total prob of N-in: 0.01933

Fusarium_sp-PT_009233-RA TMHMM2.0 outside 1 265

# Fusarium_sp-PT_009236-RA Length: 606

# Fusarium_sp-PT_009236-RA Number of predicted TMHs: 12

# Fusarium_sp-PT_009236-RA Exp number of AAs in TMHs: 252.04278

# Fusarium_sp-PT_009236-RA Exp number, first 60 AAs: 0

# Fusarium_sp-PT_009236-RA Total prob of N-in: 0.97875

Fusarium_sp-PT_009236-RA TMHMM2.0 inside 1 127

Fusarium_sp-PT_009236-RA TMHMM2.0 TMhelix 128 150

Fusarium_sp-PT_009236-RA TMHMM2.0 outside 151 164

Fusarium_sp-PT_009236-RA TMHMM2.0 TMhelix 165 187

Fusarium_sp-PT_009236-RA TMHMM2.0 inside 188 193

Fusarium_sp-PT_009236-RA TMHMM2.0 TMhelix 194 216

Fusarium_sp-PT_009236-RA TMHMM2.0 outside 217 219

Fusarium_sp-PT_009236-RA TMHMM2.0 TMhelix 220 242

Fusarium_sp-PT_009236-RA TMHMM2.0 inside 243 254

Fusarium_sp-PT_009236-RA TMHMM2.0 TMhelix 255 277

Fusarium_sp-PT_009236-RA TMHMM2.0 outside 278 286

Fusarium_sp-PT_009236-RA TMHMM2.0 TMhelix 287 309

Fusarium_sp-PT_009236-RA TMHMM2.0 inside 310 359

Fusarium_sp-PT_009236-RA TMHMM2.0 TMhelix 360 382

Fusarium_sp-PT_009236-RA TMHMM2.0 outside 383 396

Fusarium_sp-PT_009236-RA TMHMM2.0 TMhelix 397 419

Fusarium_sp-PT_009236-RA TMHMM2.0 inside 420 438

Fusarium_sp-PT_009236-RA TMHMM2.0 TMhelix 439 461

Fusarium_sp-PT_009236-RA TMHMM2.0 outside 462 464

Fusarium_sp-PT_009236-RA TMHMM2.0 TMhelix 465 487

Fusarium_sp-PT_009236-RA TMHMM2.0 inside 488 498

Fusarium_sp-PT_009236-RA TMHMM2.0 TMhelix 499 521

Fusarium_sp-PT_009236-RA TMHMM2.0 outside 522 535

Fusarium_sp-PT_009236-RA TMHMM2.0 TMhelix 536 555

Fusarium_sp-PT_009236-RA TMHMM2.0 inside 556 606

# Fusarium_sp-PT_009238-RA Length: 860

# Fusarium_sp-PT_009238-RA Number of predicted TMHs: 10

# Fusarium_sp-PT_009238-RA Exp number of AAs in TMHs: 232.87845

# Fusarium_sp-PT_009238-RA Exp number, first 60 AAs: 21.62352

# Fusarium_sp-PT_009238-RA Total prob of N-in: 0.02880

# Fusarium_sp-PT_009238-RA POSSIBLE N-term signal sequence

Fusarium_sp-PT_009238-RA TMHMM2.0 outside 1 21

Fusarium_sp-PT_009238-RA TMHMM2.0 TMhelix 22 44

Fusarium_sp-PT_009238-RA TMHMM2.0 inside 45 104

Fusarium_sp-PT_009238-RA TMHMM2.0 TMhelix 105 127

Fusarium_sp-PT_009238-RA TMHMM2.0 outside 128 174

Fusarium_sp-PT_009238-RA TMHMM2.0 TMhelix 175 197

Fusarium_sp-PT_009238-RA TMHMM2.0 inside 198 436

Fusarium_sp-PT_009238-RA TMHMM2.0 TMhelix 437 459

Fusarium_sp-PT_009238-RA TMHMM2.0 outside 460 531

Fusarium_sp-PT_009238-RA TMHMM2.0 TMhelix 532 554

Fusarium_sp-PT_009238-RA TMHMM2.0 inside 555 574

Fusarium_sp-PT_009238-RA TMHMM2.0 TMhelix 575 597

Fusarium_sp-PT_009238-RA TMHMM2.0 outside 598 627

Fusarium_sp-PT_009238-RA TMHMM2.0 TMhelix 628 650

Fusarium_sp-PT_009238-RA TMHMM2.0 inside 651 654

Fusarium_sp-PT_009238-RA TMHMM2.0 TMhelix 655 677

Fusarium_sp-PT_009238-RA TMHMM2.0 outside 678 686

Fusarium_sp-PT_009238-RA TMHMM2.0 TMhelix 687 709

Fusarium_sp-PT_009238-RA TMHMM2.0 inside 710 715

Fusarium_sp-PT_009238-RA TMHMM2.0 TMhelix 716 738

Fusarium_sp-PT_009238-RA TMHMM2.0 outside 739 860

# Fusarium_sp-PT_009237-RA Length: 343

# Fusarium_sp-PT_009237-RA Number of predicted TMHs: 0

# Fusarium_sp-PT_009237-RA Exp number of AAs in TMHs: 0.0364800000000001

# Fusarium_sp-PT_009237-RA Exp number, first 60 AAs: 0.000500000000000001

# Fusarium_sp-PT_009237-RA Total prob of N-in: 0.06218

Fusarium_sp-PT_009237-RA TMHMM2.0 outside 1 343

# Fusarium_sp-PT_009239-RA Length: 581

# Fusarium_sp-PT_009239-RA Number of predicted TMHs: 14

# Fusarium_sp-PT_009239-RA Exp number of AAs in TMHs: 296.8127

# Fusarium_sp-PT_009239-RA Exp number, first 60 AAs: 1.88392

# Fusarium_sp-PT_009239-RA Total prob of N-in: 0.99546

Fusarium_sp-PT_009239-RA TMHMM2.0 inside 1 62

Fusarium_sp-PT_009239-RA TMHMM2.0 TMhelix 63 85

Fusarium_sp-PT_009239-RA TMHMM2.0 outside 86 99

Fusarium_sp-PT_009239-RA TMHMM2.0 TMhelix 100 119

Fusarium_sp-PT_009239-RA TMHMM2.0 inside 120 130

Fusarium_sp-PT_009239-RA TMHMM2.0 TMhelix 131 145

Fusarium_sp-PT_009239-RA TMHMM2.0 outside 146 154

Fusarium_sp-PT_009239-RA TMHMM2.0 TMhelix 155 177

Fusarium_sp-PT_009239-RA TMHMM2.0 inside 178 189

Fusarium_sp-PT_009239-RA TMHMM2.0 TMhelix 190 212

Fusarium_sp-PT_009239-RA TMHMM2.0 outside 213 216

Fusarium_sp-PT_009239-RA TMHMM2.0 TMhelix 217 239

Fusarium_sp-PT_009239-RA TMHMM2.0 inside 240 266

Fusarium_sp-PT_009239-RA TMHMM2.0 TMhelix 267 289

Fusarium_sp-PT_009239-RA TMHMM2.0 outside 290 303

Fusarium_sp-PT_009239-RA TMHMM2.0 TMhelix 304 321

Fusarium_sp-PT_009239-RA TMHMM2.0 inside 322 340

Fusarium_sp-PT_009239-RA TMHMM2.0 TMhelix 341 363

Fusarium_sp-PT_009239-RA TMHMM2.0 outside 364 377

Fusarium_sp-PT_009239-RA TMHMM2.0 TMhelix 378 400

Fusarium_sp-PT_009239-RA TMHMM2.0 inside 401 404

Fusarium_sp-PT_009239-RA TMHMM2.0 TMhelix 405 427

Fusarium_sp-PT_009239-RA TMHMM2.0 outside 428 436

Fusarium_sp-PT_009239-RA TMHMM2.0 TMhelix 437 459

Fusarium_sp-PT_009239-RA TMHMM2.0 inside 460 471

Fusarium_sp-PT_009239-RA TMHMM2.0 TMhelix 472 494

Fusarium_sp-PT_009239-RA TMHMM2.0 outside 495 547

Fusarium_sp-PT_009239-RA TMHMM2.0 TMhelix 548 566

Fusarium_sp-PT_009239-RA TMHMM2.0 inside 567 581

# Fusarium_sp-PT_009240-RA Length: 253

# Fusarium_sp-PT_009240-RA Number of predicted TMHs: 1

# Fusarium_sp-PT_009240-RA Exp number of AAs in TMHs: 22.74551

# Fusarium_sp-PT_009240-RA Exp number, first 60 AAs: 0.02525

# Fusarium_sp-PT_009240-RA Total prob of N-in: 0.00301

Fusarium_sp-PT_009240-RA TMHMM2.0 outside 1 148

Fusarium_sp-PT_009240-RA TMHMM2.0 TMhelix 149 171

Fusarium_sp-PT_009240-RA TMHMM2.0 inside 172 253

# Fusarium_sp-PT_009242-RA Length: 647

# Fusarium_sp-PT_009242-RA Number of predicted TMHs: 0

# Fusarium_sp-PT_009242-RA Exp number of AAs in TMHs: 0.0416700000000001

# Fusarium_sp-PT_009242-RA Exp number, first 60 AAs: 0

# Fusarium_sp-PT_009242-RA Total prob of N-in: 0.00396

Fusarium_sp-PT_009242-RA TMHMM2.0 outside 1 647

# Fusarium_sp-PT_009243-RA Length: 491

# Fusarium_sp-PT_009243-RA Number of predicted TMHs: 0

# Fusarium_sp-PT_009243-RA Exp number of AAs in TMHs: 0.00481999999999999

# Fusarium_sp-PT_009243-RA Exp number, first 60 AAs: 0

# Fusarium_sp-PT_009243-RA Total prob of N-in: 0.00076

Fusarium_sp-PT_009243-RA TMHMM2.0 outside 1 491

# Fusarium_sp-PT_009245-RA Length: 1467

# Fusarium_sp-PT_009245-RA Number of predicted TMHs: 16

# Fusarium_sp-PT_009245-RA Exp number of AAs in TMHs: 348.52948

# Fusarium_sp-PT_009245-RA Exp number, first 60 AAs: 21.26075

# Fusarium_sp-PT_009245-RA Total prob of N-in: 0.00510

# Fusarium_sp-PT_009245-RA POSSIBLE N-term signal sequence

Fusarium_sp-PT_009245-RA TMHMM2.0 outside 1 31

Fusarium_sp-PT_009245-RA TMHMM2.0 TMhelix 32 54

Fusarium_sp-PT_009245-RA TMHMM2.0 inside 55 74

Fusarium_sp-PT_009245-RA TMHMM2.0 TMhelix 75 94

Fusarium_sp-PT_009245-RA TMHMM2.0 outside 95 103

Fusarium_sp-PT_009245-RA TMHMM2.0 TMhelix 104 123

Fusarium_sp-PT_009245-RA TMHMM2.0 inside 124 135

Fusarium_sp-PT_009245-RA TMHMM2.0 TMhelix 136 158

Fusarium_sp-PT_009245-RA TMHMM2.0 outside 159 161

Fusarium_sp-PT_009245-RA TMHMM2.0 TMhelix 162 184

Fusarium_sp-PT_009245-RA TMHMM2.0 inside 185 273

Fusarium_sp-PT_009245-RA TMHMM2.0 TMhelix 274 296

Fusarium_sp-PT_009245-RA TMHMM2.0 outside 297 310

Fusarium_sp-PT_009245-RA TMHMM2.0 TMhelix 311 333

Fusarium_sp-PT_009245-RA TMHMM2.0 inside 334 390

Fusarium_sp-PT_009245-RA TMHMM2.0 TMhelix 391 413

Fusarium_sp-PT_009245-RA TMHMM2.0 outside 414 416

Fusarium_sp-PT_009245-RA TMHMM2.0 TMhelix 417 434

Fusarium_sp-PT_009245-RA TMHMM2.0 inside 435 493

Fusarium_sp-PT_009245-RA TMHMM2.0 TMhelix 494 516

Fusarium_sp-PT_009245-RA TMHMM2.0 outside 517 530

Fusarium_sp-PT_009245-RA TMHMM2.0 TMhelix 531 553

Fusarium_sp-PT_009245-RA TMHMM2.0 inside 554 897

Fusarium_sp-PT_009245-RA TMHMM2.0 TMhelix 898 920

Fusarium_sp-PT_009245-RA TMHMM2.0 outside 921 950

Fusarium_sp-PT_009245-RA TMHMM2.0 TMhelix 951 973

Fusarium_sp-PT_009245-RA TMHMM2.0 inside 974 1017

Fusarium_sp-PT_009245-RA TMHMM2.0 TMhelix 1018 1035

Fusarium_sp-PT_009245-RA TMHMM2.0 outside 1036 1038

Fusarium_sp-PT_009245-RA TMHMM2.0 TMhelix 1039 1061

Fusarium_sp-PT_009245-RA TMHMM2.0 inside 1062 1128

Fusarium_sp-PT_009245-RA TMHMM2.0 TMhelix 1129 1151

Fusarium_sp-PT_009245-RA TMHMM2.0 outside 1152 1467

# Fusarium_sp-PT_009251-RA Length: 337

# Fusarium_sp-PT_009251-RA Number of predicted TMHs: 0

# Fusarium_sp-PT_009251-RA Exp number of AAs in TMHs: 5.35138000000001

# Fusarium_sp-PT_009251-RA Exp number, first 60 AAs: 4.06717

# Fusarium_sp-PT_009251-RA Total prob of N-in: 0.18462

Fusarium_sp-PT_009251-RA TMHMM2.0 outside 1 337

# Fusarium_sp-PT_009253-RA Length: 231

# Fusarium_sp-PT_009253-RA Number of predicted TMHs: 1

# Fusarium_sp-PT_009253-RA Exp number of AAs in TMHs: 22.17421

# Fusarium_sp-PT_009253-RA Exp number, first 60 AAs: 0.01973

# Fusarium_sp-PT_009253-RA Total prob of N-in: 0.97856

Fusarium_sp-PT_009253-RA TMHMM2.0 inside 1 75

Fusarium_sp-PT_009253-RA TMHMM2.0 TMhelix 76 98

Fusarium_sp-PT_009253-RA TMHMM2.0 outside 99 231

# Fusarium_sp-PT_009255-RA Length: 405

# Fusarium_sp-PT_009255-RA Number of predicted TMHs: 0

# Fusarium_sp-PT_009255-RA Exp number of AAs in TMHs: 0.0783999999999999

# Fusarium_sp-PT_009255-RA Exp number, first 60 AAs: 0

# Fusarium_sp-PT_009255-RA Total prob of N-in: 0.01375

Fusarium_sp-PT_009255-RA TMHMM2.0 outside 1 405

# Fusarium_sp-PT_009256-RA Length: 412

# Fusarium_sp-PT_009256-RA Number of predicted TMHs: 0

# Fusarium_sp-PT_009256-RA Exp number of AAs in TMHs: 0.00022

# Fusarium_sp-PT_009256-RA Exp number, first 60 AAs: 0.00022

# Fusarium_sp-PT_009256-RA Total prob of N-in: 0.00153

Fusarium_sp-PT_009256-RA TMHMM2.0 outside 1 412

# Fusarium_sp-PT_009257-RA Length: 284

# Fusarium_sp-PT_009257-RA Number of predicted TMHs: 0

# Fusarium_sp-PT_009257-RA Exp number of AAs in TMHs: 5.86182

# Fusarium_sp-PT_009257-RA Exp number, first 60 AAs: 0.03587

# Fusarium_sp-PT_009257-RA Total prob of N-in: 0.03972

Fusarium_sp-PT_009257-RA TMHMM2.0 outside 1 284

# Fusarium_sp-PT_009259-RA Length: 373

# Fusarium_sp-PT_009259-RA Number of predicted TMHs: 0

# Fusarium_sp-PT_009259-RA Exp number of AAs in TMHs: 2.32986

# Fusarium_sp-PT_009259-RA Exp number, first 60 AAs: 2.28676

# Fusarium_sp-PT_009259-RA Total prob of N-in: 0.13336

Fusarium_sp-PT_009259-RA TMHMM2.0 outside 1 373

# Fusarium_sp-PT_009262-RA Length: 659

# Fusarium_sp-PT_009262-RA Number of predicted TMHs: 0

# Fusarium_sp-PT_009262-RA Exp number of AAs in TMHs: 3.63884

# Fusarium_sp-PT_009262-RA Exp number, first 60 AAs: 0

# Fusarium_sp-PT_009262-RA Total prob of N-in: 0.04428

Fusarium_sp-PT_009262-RA TMHMM2.0 outside 1 659

# Fusarium_sp-PT_009263-RA Length: 595

# Fusarium_sp-PT_009263-RA Number of predicted TMHs: 1

# Fusarium_sp-PT_009263-RA Exp number of AAs in TMHs: 22.58781

# Fusarium_sp-PT_009263-RA Exp number, first 60 AAs: 0.06515

# Fusarium_sp-PT_009263-RA Total prob of N-in: 0.02817

Fusarium_sp-PT_009263-RA TMHMM2.0 outside 1 396

Fusarium_sp-PT_009263-RA TMHMM2.0 TMhelix 397 419

Fusarium_sp-PT_009263-RA TMHMM2.0 inside 420 595

# Fusarium_sp-PT_009241-RA Length: 1556

# Fusarium_sp-PT_009241-RA Number of predicted TMHs: 0

# Fusarium_sp-PT_009241-RA Exp number of AAs in TMHs: 0.01777

# Fusarium_sp-PT_009241-RA Exp number, first 60 AAs: 0

# Fusarium_sp-PT_009241-RA Total prob of N-in: 0.00058

Fusarium_sp-PT_009241-RA TMHMM2.0 outside 1 1556

# Fusarium_sp-PT_009244-RA Length: 719

# Fusarium_sp-PT_009244-RA Number of predicted TMHs: 0

# Fusarium_sp-PT_009244-RA Exp number of AAs in TMHs: 0.00066

# Fusarium_sp-PT_009244-RA Exp number, first 60 AAs: 0

# Fusarium_sp-PT_009244-RA Total prob of N-in: 0.00019

Fusarium_sp-PT_009244-RA TMHMM2.0 outside 1 719

# Fusarium_sp-PT_009246-RA Length: 465

# Fusarium_sp-PT_009246-RA Number of predicted TMHs: 0

# Fusarium_sp-PT_009246-RA Exp number of AAs in TMHs: 0.03964

# Fusarium_sp-PT_009246-RA Exp number, first 60 AAs: 0

# Fusarium_sp-PT_009246-RA Total prob of N-in: 0.01986

Fusarium_sp-PT_009246-RA TMHMM2.0 outside 1 465

# Fusarium_sp-PT_009250-RA Length: 530

# Fusarium_sp-PT_009250-RA Number of predicted TMHs: 0

# Fusarium_sp-PT_009250-RA Exp number of AAs in TMHs: 0.01112

# Fusarium_sp-PT_009250-RA Exp number, first 60 AAs: 0

# Fusarium_sp-PT_009250-RA Total prob of N-in: 0.00077

Fusarium_sp-PT_009250-RA TMHMM2.0 outside 1 530

# Fusarium_sp-PT_009252-RA Length: 482

# Fusarium_sp-PT_009252-RA Number of predicted TMHs: 0

# Fusarium_sp-PT_009252-RA Exp number of AAs in TMHs: 0.000640000000000001

# Fusarium_sp-PT_009252-RA Exp number, first 60 AAs: 0.00043

# Fusarium_sp-PT_009252-RA Total prob of N-in: 0.00517

Fusarium_sp-PT_009252-RA TMHMM2.0 outside 1 482

# Fusarium_sp-PT_009254-RA Length: 418

# Fusarium_sp-PT_009254-RA Number of predicted TMHs: 0

# Fusarium_sp-PT_009254-RA Exp number of AAs in TMHs: 0.00023

# Fusarium_sp-PT_009254-RA Exp number, first 60 AAs: 0

# Fusarium_sp-PT_009254-RA Total prob of N-in: 0.01577

Fusarium_sp-PT_009254-RA TMHMM2.0 outside 1 418

# Fusarium_sp-PT_009258-RA Length: 86

# Fusarium_sp-PT_009258-RA Number of predicted TMHs: 0

# Fusarium_sp-PT_009258-RA Exp number of AAs in TMHs: 0

# Fusarium_sp-PT_009258-RA Exp number, first 60 AAs: 0

# Fusarium_sp-PT_009258-RA Total prob of N-in: 0.14320

Fusarium_sp-PT_009258-RA TMHMM2.0 outside 1 86

# Fusarium_sp-PT_009260-RA Length: 397

# Fusarium_sp-PT_009260-RA Number of predicted TMHs: 0

# Fusarium_sp-PT_009260-RA Exp number of AAs in TMHs: 0.00109

# Fusarium_sp-PT_009260-RA Exp number, first 60 AAs: 0.00109

# Fusarium_sp-PT_009260-RA Total prob of N-in: 0.00780

Fusarium_sp-PT_009260-RA TMHMM2.0 outside 1 397

# Fusarium_sp-PT_009261-RA Length: 255

# Fusarium_sp-PT_009261-RA Number of predicted TMHs: 0

# Fusarium_sp-PT_009261-RA Exp number of AAs in TMHs: 0.00031

# Fusarium_sp-PT_009261-RA Exp number, first 60 AAs: 0.00012

# Fusarium_sp-PT_009261-RA Total prob of N-in: 0.01970

Fusarium_sp-PT_009261-RA TMHMM2.0 outside 1 255

# Fusarium_sp-PT_009249-RA Length: 568

# Fusarium_sp-PT_009249-RA Number of predicted TMHs: 0

# Fusarium_sp-PT_009249-RA Exp number of AAs in TMHs: 1.27964

# Fusarium_sp-PT_009249-RA Exp number, first 60 AAs: 1.27584

# Fusarium_sp-PT_009249-RA Total prob of N-in: 0.05956

Fusarium_sp-PT_009249-RA TMHMM2.0 outside 1 568

# Fusarium_sp-PT_009247-RA Length: 55

# Fusarium_sp-PT_009247-RA Number of predicted TMHs: 0

# Fusarium_sp-PT_009247-RA Exp number of AAs in TMHs: 0

# Fusarium_sp-PT_009247-RA Exp number, first 60 AAs: 0

# Fusarium_sp-PT_009247-RA Total prob of N-in: 0.13339

Fusarium_sp-PT_009247-RA TMHMM2.0 outside 1 55

# Fusarium_sp-PT_009248-RA Length: 27

# Fusarium_sp-PT_009248-RA Number of predicted TMHs: 0

# Fusarium_sp-PT_009248-RA Exp number of AAs in TMHs: 0

# Fusarium_sp-PT_009248-RA Exp number, first 60 AAs: 0

# Fusarium_sp-PT_009248-RA Total prob of N-in: 0.72411

Fusarium_sp-PT_009248-RA TMHMM2.0 inside 1 27

# Fusarium_sp-PT_009265-RA Length: 255

# Fusarium_sp-PT_009265-RA Number of predicted TMHs: 2

# Fusarium_sp-PT_009265-RA Exp number of AAs in TMHs: 42.50576

# Fusarium_sp-PT_009265-RA Exp number, first 60 AAs: 42.47246

# Fusarium_sp-PT_009265-RA Total prob of N-in: 0.61468

# Fusarium_sp-PT_009265-RA POSSIBLE N-term signal sequence

Fusarium_sp-PT_009265-RA TMHMM2.0 outside 1 9

Fusarium_sp-PT_009265-RA TMHMM2.0 TMhelix 10 29

Fusarium_sp-PT_009265-RA TMHMM2.0 inside 30 35

Fusarium_sp-PT_009265-RA TMHMM2.0 TMhelix 36 58

Fusarium_sp-PT_009265-RA TMHMM2.0 outside 59 255

# Fusarium_sp-PT_009268-RA Length: 153

# Fusarium_sp-PT_009268-RA Number of predicted TMHs: 1

# Fusarium_sp-PT_009268-RA Exp number of AAs in TMHs: 22.57102

# Fusarium_sp-PT_009268-RA Exp number, first 60 AAs: 0.00015

# Fusarium_sp-PT_009268-RA Total prob of N-in: 0.02702

Fusarium_sp-PT_009268-RA TMHMM2.0 outside 1 124

Fusarium_sp-PT_009268-RA TMHMM2.0 TMhelix 125 147

Fusarium_sp-PT_009268-RA TMHMM2.0 inside 148 153

# Fusarium_sp-PT_009271-RA Length: 644

# Fusarium_sp-PT_009271-RA Number of predicted TMHs: 0

# Fusarium_sp-PT_009271-RA Exp number of AAs in TMHs: 0.00666999999999999

# Fusarium_sp-PT_009271-RA Exp number, first 60 AAs: 2e-05

# Fusarium_sp-PT_009271-RA Total prob of N-in: 0.00303

Fusarium_sp-PT_009271-RA TMHMM2.0 outside 1 644

# Fusarium_sp-PT_009272-RA Length: 1156

# Fusarium_sp-PT_009272-RA Number of predicted TMHs: 0

# Fusarium_sp-PT_009272-RA Exp number of AAs in TMHs: 0.00064

# Fusarium_sp-PT_009272-RA Exp number, first 60 AAs: 0.00021

# Fusarium_sp-PT_009272-RA Total prob of N-in: 0.00003

Fusarium_sp-PT_009272-RA TMHMM2.0 outside 1 1156

# Fusarium_sp-PT_009275-RA Length: 123

# Fusarium_sp-PT_009275-RA Number of predicted TMHs: 0

# Fusarium_sp-PT_009275-RA Exp number of AAs in TMHs: 0

# Fusarium_sp-PT_009275-RA Exp number, first 60 AAs: 0

# Fusarium_sp-PT_009275-RA Total prob of N-in: 0.02252

Fusarium_sp-PT_009275-RA TMHMM2.0 outside 1 123

# Fusarium_sp-PT_009277-RA Length: 2104

# Fusarium_sp-PT_009277-RA Number of predicted TMHs: 0

# Fusarium_sp-PT_009277-RA Exp number of AAs in TMHs: 0.10316

# Fusarium_sp-PT_009277-RA Exp number, first 60 AAs: 0.00324

# Fusarium_sp-PT_009277-RA Total prob of N-in: 0.00098

Fusarium_sp-PT_009277-RA TMHMM2.0 outside 1 2104

# Fusarium_sp-PT_009278-RA Length: 465

# Fusarium_sp-PT_009278-RA Number of predicted TMHs: 0

# Fusarium_sp-PT_009278-RA Exp number of AAs in TMHs: 0.05108

# Fusarium_sp-PT_009278-RA Exp number, first 60 AAs: 0

# Fusarium_sp-PT_009278-RA Total prob of N-in: 0.00856

Fusarium_sp-PT_009278-RA TMHMM2.0 outside 1 465

# Fusarium_sp-PT_009282-RA Length: 963

# Fusarium_sp-PT_009282-RA Number of predicted TMHs: 0

# Fusarium_sp-PT_009282-RA Exp number of AAs in TMHs: 0.00494

# Fusarium_sp-PT_009282-RA Exp number, first 60 AAs: 0

# Fusarium_sp-PT_009282-RA Total prob of N-in: 0.00001

Fusarium_sp-PT_009282-RA TMHMM2.0 outside 1 963

# Fusarium_sp-PT_009285-RA Length: 421

# Fusarium_sp-PT_009285-RA Number of predicted TMHs: 0

# Fusarium_sp-PT_009285-RA Exp number of AAs in TMHs: 0.05197

# Fusarium_sp-PT_009285-RA Exp number, first 60 AAs: 0.00058

# Fusarium_sp-PT_009285-RA Total prob of N-in: 0.00654

Fusarium_sp-PT_009285-RA TMHMM2.0 outside 1 421

# Fusarium_sp-PT_009286-RA Length: 1108

# Fusarium_sp-PT_009286-RA Number of predicted TMHs: 0

# Fusarium_sp-PT_009286-RA Exp number of AAs in TMHs: 1.7999

# Fusarium_sp-PT_009286-RA Exp number, first 60 AAs: 1.68084

# Fusarium_sp-PT_009286-RA Total prob of N-in: 0.09168

Fusarium_sp-PT_009286-RA TMHMM2.0 outside 1 1108

# Fusarium_sp-PT_009290-RA Length: 312

# Fusarium_sp-PT_009290-RA Number of predicted TMHs: 0

# Fusarium_sp-PT_009290-RA Exp number of AAs in TMHs: 0.0002

# Fusarium_sp-PT_009290-RA Exp number, first 60 AAs: 0.0002

# Fusarium_sp-PT_009290-RA Total prob of N-in: 0.02507

Fusarium_sp-PT_009290-RA TMHMM2.0 outside 1 312

# Fusarium_sp-PT_009264-RA Length: 376

# Fusarium_sp-PT_009264-RA Number of predicted TMHs: 0

# Fusarium_sp-PT_009264-RA Exp number of AAs in TMHs: 0.00857

# Fusarium_sp-PT_009264-RA Exp number, first 60 AAs: 0.000650000000000001

# Fusarium_sp-PT_009264-RA Total prob of N-in: 0.01192

Fusarium_sp-PT_009264-RA TMHMM2.0 outside 1 376

# Fusarium_sp-PT_009267-RA Length: 201

# Fusarium_sp-PT_009267-RA Number of predicted TMHs: 0

# Fusarium_sp-PT_009267-RA Exp number of AAs in TMHs: 0

# Fusarium_sp-PT_009267-RA Exp number, first 60 AAs: 0

# Fusarium_sp-PT_009267-RA Total prob of N-in: 0.01189

Fusarium_sp-PT_009267-RA TMHMM2.0 outside 1 201

# Fusarium_sp-PT_009269-RA Length: 577

# Fusarium_sp-PT_009269-RA Number of predicted TMHs: 0

# Fusarium_sp-PT_009269-RA Exp number of AAs in TMHs: 0.01958

# Fusarium_sp-PT_009269-RA Exp number, first 60 AAs: 0.0004

# Fusarium_sp-PT_009269-RA Total prob of N-in: 0.00187

Fusarium_sp-PT_009269-RA TMHMM2.0 outside 1 577

# Fusarium_sp-PT_009270-RA Length: 420

# Fusarium_sp-PT_009270-RA Number of predicted TMHs: 0

# Fusarium_sp-PT_009270-RA Exp number of AAs in TMHs: 0.00391

# Fusarium_sp-PT_009270-RA Exp number, first 60 AAs: 0.0007

# Fusarium_sp-PT_009270-RA Total prob of N-in: 0.01053

Fusarium_sp-PT_009270-RA TMHMM2.0 outside 1 420

# Fusarium_sp-PT_009273-RA Length: 609

# Fusarium_sp-PT_009273-RA Number of predicted TMHs: 12

# Fusarium_sp-PT_009273-RA Exp number of AAs in TMHs: 260.24119

# Fusarium_sp-PT_009273-RA Exp number, first 60 AAs: 0.00035

# Fusarium_sp-PT_009273-RA Total prob of N-in: 0.97821

Fusarium_sp-PT_009273-RA TMHMM2.0 inside 1 162

Fusarium_sp-PT_009273-RA TMHMM2.0 TMhelix 163 185

Fusarium_sp-PT_009273-RA TMHMM2.0 outside 186 199

Fusarium_sp-PT_009273-RA TMHMM2.0 TMhelix 200 222

Fusarium_sp-PT_009273-RA TMHMM2.0 inside 223 230

Fusarium_sp-PT_009273-RA TMHMM2.0 TMhelix 231 250

Fusarium_sp-PT_009273-RA TMHMM2.0 outside 251 255

Fusarium_sp-PT_009273-RA TMHMM2.0 TMhelix 256 278

Fusarium_sp-PT_009273-RA TMHMM2.0 inside 279 290

Fusarium_sp-PT_009273-RA TMHMM2.0 TMhelix 291 313

Fusarium_sp-PT_009273-RA TMHMM2.0 outside 314 327

Fusarium_sp-PT_009273-RA TMHMM2.0 TMhelix 328 350

Fusarium_sp-PT_009273-RA TMHMM2.0 inside 351 397

Fusarium_sp-PT_009273-RA TMHMM2.0 TMhelix 398 420

Fusarium_sp-PT_009273-RA TMHMM2.0 outside 421 434

Fusarium_sp-PT_009273-RA TMHMM2.0 TMhelix 435 457

Fusarium_sp-PT_009273-RA TMHMM2.0 inside 458 476

Fusarium_sp-PT_009273-RA TMHMM2.0 TMhelix 477 496

Fusarium_sp-PT_009273-RA TMHMM2.0 outside 497 510

Fusarium_sp-PT_009273-RA TMHMM2.0 TMhelix 511 533

Fusarium_sp-PT_009273-RA TMHMM2.0 inside 534 539

Fusarium_sp-PT_009273-RA TMHMM2.0 TMhelix 540 562

Fusarium_sp-PT_009273-RA TMHMM2.0 outside 563 571

Fusarium_sp-PT_009273-RA TMHMM2.0 TMhelix 572 594

Fusarium_sp-PT_009273-RA TMHMM2.0 inside 595 609

# Fusarium_sp-PT_009274-RA Length: 667

# Fusarium_sp-PT_009274-RA Number of predicted TMHs: 0

# Fusarium_sp-PT_009274-RA Exp number of AAs in TMHs: 0.02573

# Fusarium_sp-PT_009274-RA Exp number, first 60 AAs: 0.00016

# Fusarium_sp-PT_009274-RA Total prob of N-in: 0.00110

Fusarium_sp-PT_009274-RA TMHMM2.0 outside 1 667

# Fusarium_sp-PT_009276-RA Length: 1855

# Fusarium_sp-PT_009276-RA Number of predicted TMHs: 0

# Fusarium_sp-PT_009276-RA Exp number of AAs in TMHs: 0.0390000000000003

# Fusarium_sp-PT_009276-RA Exp number, first 60 AAs: 0.03211

# Fusarium_sp-PT_009276-RA Total prob of N-in: 0.00180

Fusarium_sp-PT_009276-RA TMHMM2.0 outside 1 1855

# Fusarium_sp-PT_009279-RA Length: 937

# Fusarium_sp-PT_009279-RA Number of predicted TMHs: 0

# Fusarium_sp-PT_009279-RA Exp number of AAs in TMHs: 14.48753

# Fusarium_sp-PT_009279-RA Exp number, first 60 AAs: 0

# Fusarium_sp-PT_009279-RA Total prob of N-in: 0.00018

Fusarium_sp-PT_009279-RA TMHMM2.0 outside 1 937

# Fusarium_sp-PT_009280-RA Length: 893

# Fusarium_sp-PT_009280-RA Number of predicted TMHs: 0

# Fusarium_sp-PT_009280-RA Exp number of AAs in TMHs: 0.536069999999999

# Fusarium_sp-PT_009280-RA Exp number, first 60 AAs: 0.00031

# Fusarium_sp-PT_009280-RA Total prob of N-in: 0.01423

Fusarium_sp-PT_009280-RA TMHMM2.0 outside 1 893

# Fusarium_sp-PT_009281-RA Length: 533

# Fusarium_sp-PT_009281-RA Number of predicted TMHs: 0

# Fusarium_sp-PT_009281-RA Exp number of AAs in TMHs: 0.0609

# Fusarium_sp-PT_009281-RA Exp number, first 60 AAs: 0.00018

# Fusarium_sp-PT_009281-RA Total prob of N-in: 0.00523

Fusarium_sp-PT_009281-RA TMHMM2.0 outside 1 533

# Fusarium_sp-PT_009283-RA Length: 502

# Fusarium_sp-PT_009283-RA Number of predicted TMHs: 0

# Fusarium_sp-PT_009283-RA Exp number of AAs in TMHs: 0.02082

# Fusarium_sp-PT_009283-RA Exp number, first 60 AAs: 0

# Fusarium_sp-PT_009283-RA Total prob of N-in: 0.00094

Fusarium_sp-PT_009283-RA TMHMM2.0 outside 1 502

# Fusarium_sp-PT_009284-RA Length: 989

# Fusarium_sp-PT_009284-RA Number of predicted TMHs: 0

# Fusarium_sp-PT_009284-RA Exp number of AAs in TMHs: 0.00735999999999999

# Fusarium_sp-PT_009284-RA Exp number, first 60 AAs: 0

# Fusarium_sp-PT_009284-RA Total prob of N-in: 0.00035

Fusarium_sp-PT_009284-RA TMHMM2.0 outside 1 989

# Fusarium_sp-PT_009287-RA Length: 183

# Fusarium_sp-PT_009287-RA Number of predicted TMHs: 0

# Fusarium_sp-PT_009287-RA Exp number of AAs in TMHs: 1e-05

# Fusarium_sp-PT_009287-RA Exp number, first 60 AAs: 0

# Fusarium_sp-PT_009287-RA Total prob of N-in: 0.09041

Fusarium_sp-PT_009287-RA TMHMM2.0 outside 1 183

# Fusarium_sp-PT_009289-RA Length: 246

# Fusarium_sp-PT_009289-RA Number of predicted TMHs: 0

# Fusarium_sp-PT_009289-RA Exp number of AAs in TMHs: 0.00327

# Fusarium_sp-PT_009289-RA Exp number, first 60 AAs: 0

# Fusarium_sp-PT_009289-RA Total prob of N-in: 0.09648

Fusarium_sp-PT_009289-RA TMHMM2.0 outside 1 246

# Fusarium_sp-PT_009291-RA Length: 253

# Fusarium_sp-PT_009291-RA Number of predicted TMHs: 0

# Fusarium_sp-PT_009291-RA Exp number of AAs in TMHs: 0.00111

# Fusarium_sp-PT_009291-RA Exp number, first 60 AAs: 1e-05

# Fusarium_sp-PT_009291-RA Total prob of N-in: 0.08550

Fusarium_sp-PT_009291-RA TMHMM2.0 outside 1 253

# Fusarium_sp-PT_009292-RA Length: 167

# Fusarium_sp-PT_009292-RA Number of predicted TMHs: 0

# Fusarium_sp-PT_009292-RA Exp number of AAs in TMHs: 0.00102

# Fusarium_sp-PT_009292-RA Exp number, first 60 AAs: 0.00102

# Fusarium_sp-PT_009292-RA Total prob of N-in: 0.01300

Fusarium_sp-PT_009292-RA TMHMM2.0 outside 1 167

# Fusarium_sp-PT_009288-RA Length: 55

# Fusarium_sp-PT_009288-RA Number of predicted TMHs: 0

# Fusarium_sp-PT_009288-RA Exp number of AAs in TMHs: 0.00015

# Fusarium_sp-PT_009288-RA Exp number, first 60 AAs: 0.00015

# Fusarium_sp-PT_009288-RA Total prob of N-in: 0.35143

Fusarium_sp-PT_009288-RA TMHMM2.0 outside 1 55

# Fusarium_sp-PT_009295-RA Length: 505

# Fusarium_sp-PT_009295-RA Number of predicted TMHs: 0

# Fusarium_sp-PT_009295-RA Exp number of AAs in TMHs: 0.02018

# Fusarium_sp-PT_009295-RA Exp number, first 60 AAs: 0.01849

# Fusarium_sp-PT_009295-RA Total prob of N-in: 0.00200

Fusarium_sp-PT_009295-RA TMHMM2.0 outside 1 505

# Fusarium_sp-PT_009296-RA Length: 445

# Fusarium_sp-PT_009296-RA Number of predicted TMHs: 1

# Fusarium_sp-PT_009296-RA Exp number of AAs in TMHs: 17.68348

# Fusarium_sp-PT_009296-RA Exp number, first 60 AAs: 17.6479

# Fusarium_sp-PT_009296-RA Total prob of N-in: 0.82740

# Fusarium_sp-PT_009296-RA POSSIBLE N-term signal sequence

Fusarium_sp-PT_009296-RA TMHMM2.0 inside 1 22

Fusarium_sp-PT_009296-RA TMHMM2.0 TMhelix 23 41

Fusarium_sp-PT_009296-RA TMHMM2.0 outside 42 445

# Fusarium_sp-PT_009299-RA Length: 800

# Fusarium_sp-PT_009299-RA Number of predicted TMHs: 0

# Fusarium_sp-PT_009299-RA Exp number of AAs in TMHs: 2.16916

# Fusarium_sp-PT_009299-RA Exp number, first 60 AAs: 0.0042

# Fusarium_sp-PT_009299-RA Total prob of N-in: 0.04975

Fusarium_sp-PT_009299-RA TMHMM2.0 outside 1 800

# Fusarium_sp-PT_009300-RA Length: 933

# Fusarium_sp-PT_009300-RA Number of predicted TMHs: 0

# Fusarium_sp-PT_009300-RA Exp number of AAs in TMHs: 0.08023

# Fusarium_sp-PT_009300-RA Exp number, first 60 AAs: 0.00039

# Fusarium_sp-PT_009300-RA Total prob of N-in: 0.00092

Fusarium_sp-PT_009300-RA TMHMM2.0 outside 1 933

# Fusarium_sp-PT_009303-RA Length: 446

# Fusarium_sp-PT_009303-RA Number of predicted TMHs: 0

# Fusarium_sp-PT_009303-RA Exp number of AAs in TMHs: 0.10368

# Fusarium_sp-PT_009303-RA Exp number, first 60 AAs: 0.00051

# Fusarium_sp-PT_009303-RA Total prob of N-in: 0.00736

Fusarium_sp-PT_009303-RA TMHMM2.0 outside 1 446

# Fusarium_sp-PT_009307-RA Length: 418

# Fusarium_sp-PT_009307-RA Number of predicted TMHs: 0

# Fusarium_sp-PT_009307-RA Exp number of AAs in TMHs: 0.0421699999999999

# Fusarium_sp-PT_009307-RA Exp number, first 60 AAs: 0.00874999999999999

# Fusarium_sp-PT_009307-RA Total prob of N-in: 0.00563

Fusarium_sp-PT_009307-RA TMHMM2.0 outside 1 418

# Fusarium_sp-PT_009309-RA Length: 190

# Fusarium_sp-PT_009309-RA Number of predicted TMHs: 0

# Fusarium_sp-PT_009309-RA Exp number of AAs in TMHs: 9.53343999999998

# Fusarium_sp-PT_009309-RA Exp number, first 60 AAs: 9.53257999999999

# Fusarium_sp-PT_009309-RA Total prob of N-in: 0.43776

Fusarium_sp-PT_009309-RA TMHMM2.0 outside 1 190

# Fusarium_sp-PT_009311-RA Length: 239

# Fusarium_sp-PT_009311-RA Number of predicted TMHs: 0

# Fusarium_sp-PT_009311-RA Exp number of AAs in TMHs: 0

# Fusarium_sp-PT_009311-RA Exp number, first 60 AAs: 0

# Fusarium_sp-PT_009311-RA Total prob of N-in: 0.05785

Fusarium_sp-PT_009311-RA TMHMM2.0 outside 1 239

# Fusarium_sp-PT_009312-RA Length: 2075

# Fusarium_sp-PT_009312-RA Number of predicted TMHs: 0

# Fusarium_sp-PT_009312-RA Exp number of AAs in TMHs: 0.0047

# Fusarium_sp-PT_009312-RA Exp number, first 60 AAs: 0.0047

# Fusarium_sp-PT_009312-RA Total prob of N-in: 0.00022

Fusarium_sp-PT_009312-RA TMHMM2.0 outside 1 2075

# Fusarium_sp-PT_009313-RA Length: 782

# Fusarium_sp-PT_009313-RA Number of predicted TMHs: 0

# Fusarium_sp-PT_009313-RA Exp number of AAs in TMHs: 0.00176

# Fusarium_sp-PT_009313-RA Exp number, first 60 AAs: 0.00034

# Fusarium_sp-PT_009313-RA Total prob of N-in: 0.00014

Fusarium_sp-PT_009313-RA TMHMM2.0 outside 1 782

# Fusarium_sp-PT_009314-RA Length: 389

# Fusarium_sp-PT_009314-RA Number of predicted TMHs: 0

# Fusarium_sp-PT_009314-RA Exp number of AAs in TMHs: 0.07968

# Fusarium_sp-PT_009314-RA Exp number, first 60 AAs: 0

# Fusarium_sp-PT_009314-RA Total prob of N-in: 0.00194

Fusarium_sp-PT_009314-RA TMHMM2.0 outside 1 389

# Fusarium_sp-PT_009316-RA Length: 341

# Fusarium_sp-PT_009316-RA Number of predicted TMHs: 1

# Fusarium_sp-PT_009316-RA Exp number of AAs in TMHs: 30.26481

# Fusarium_sp-PT_009316-RA Exp number, first 60 AAs: 0.04751

# Fusarium_sp-PT_009316-RA Total prob of N-in: 0.44946

Fusarium_sp-PT_009316-RA TMHMM2.0 inside 1 117

Fusarium_sp-PT_009316-RA TMHMM2.0 TMhelix 118 137

Fusarium_sp-PT_009316-RA TMHMM2.0 outside 138 341

# Fusarium_sp-PT_009319-RA Length: 251

# Fusarium_sp-PT_009319-RA Number of predicted TMHs: 0

# Fusarium_sp-PT_009319-RA Exp number of AAs in TMHs: 1.01922

# Fusarium_sp-PT_009319-RA Exp number, first 60 AAs: 0.31424

# Fusarium_sp-PT_009319-RA Total prob of N-in: 0.38041

Fusarium_sp-PT_009319-RA TMHMM2.0 outside 1 251

# Fusarium_sp-PT_009321-RA Length: 565

# Fusarium_sp-PT_009321-RA Number of predicted TMHs: 0

# Fusarium_sp-PT_009321-RA Exp number of AAs in TMHs: 0.00016

# Fusarium_sp-PT_009321-RA Exp number, first 60 AAs: 0.00016

# Fusarium_sp-PT_009321-RA Total prob of N-in: 0.00108

Fusarium_sp-PT_009321-RA TMHMM2.0 outside 1 565

# Fusarium_sp-PT_009325-RA Length: 4860

# Fusarium_sp-PT_009325-RA Number of predicted TMHs: 0

# Fusarium_sp-PT_009325-RA Exp number of AAs in TMHs: 5.69788999999993

# Fusarium_sp-PT_009325-RA Exp number, first 60 AAs: 0.0007

# Fusarium_sp-PT_009325-RA Total prob of N-in: 0.24813

Fusarium_sp-PT_009325-RA TMHMM2.0 outside 1 4860

# Fusarium_sp-PT_009328-RA Length: 509

# Fusarium_sp-PT_009328-RA Number of predicted TMHs: 0

# Fusarium_sp-PT_009328-RA Exp number of AAs in TMHs: 0.03362

# Fusarium_sp-PT_009328-RA Exp number, first 60 AAs: 0

# Fusarium_sp-PT_009328-RA Total prob of N-in: 0.00218

Fusarium_sp-PT_009328-RA TMHMM2.0 outside 1 509

# Fusarium_sp-PT_009294-RA Length: 163

# Fusarium_sp-PT_009294-RA Number of predicted TMHs: 0

# Fusarium_sp-PT_009294-RA Exp number of AAs in TMHs: 0.00018

# Fusarium_sp-PT_009294-RA Exp number, first 60 AAs: 0

# Fusarium_sp-PT_009294-RA Total prob of N-in: 0.13331

Fusarium_sp-PT_009294-RA TMHMM2.0 outside 1 163

# Fusarium_sp-PT_009297-RA Length: 551

# Fusarium_sp-PT_009297-RA Number of predicted TMHs: 8

# Fusarium_sp-PT_009297-RA Exp number of AAs in TMHs: 212.3979

# Fusarium_sp-PT_009297-RA Exp number, first 60 AAs: 14.13515

# Fusarium_sp-PT_009297-RA Total prob of N-in: 0.86894

# Fusarium_sp-PT_009297-RA POSSIBLE N-term signal sequence

Fusarium_sp-PT_009297-RA TMHMM2.0 inside 1 45

Fusarium_sp-PT_009297-RA TMHMM2.0 TMhelix 46 68

Fusarium_sp-PT_009297-RA TMHMM2.0 outside 69 89

Fusarium_sp-PT_009297-RA TMHMM2.0 TMhelix 90 112

Fusarium_sp-PT_009297-RA TMHMM2.0 inside 113 124

Fusarium_sp-PT_009297-RA TMHMM2.0 TMhelix 125 147

Fusarium_sp-PT_009297-RA TMHMM2.0 outside 148 184

Fusarium_sp-PT_009297-RA TMHMM2.0 TMhelix 185 207

Fusarium_sp-PT_009297-RA TMHMM2.0 inside 208 365

Fusarium_sp-PT_009297-RA TMHMM2.0 TMhelix 366 388

Fusarium_sp-PT_009297-RA TMHMM2.0 outside 389 417

Fusarium_sp-PT_009297-RA TMHMM2.0 TMhelix 418 440

Fusarium_sp-PT_009297-RA TMHMM2.0 inside 441 444

Fusarium_sp-PT_009297-RA TMHMM2.0 TMhelix 445 467

Fusarium_sp-PT_009297-RA TMHMM2.0 outside 468 481

Fusarium_sp-PT_009297-RA TMHMM2.0 TMhelix 482 504

Fusarium_sp-PT_009297-RA TMHMM2.0 inside 505 551

# Fusarium_sp-PT_009298-RA Length: 959

# Fusarium_sp-PT_009298-RA Number of predicted TMHs: 0

# Fusarium_sp-PT_009298-RA Exp number of AAs in TMHs: 0.00641

# Fusarium_sp-PT_009298-RA Exp number, first 60 AAs: 0.00015

# Fusarium_sp-PT_009298-RA Total prob of N-in: 0.00006

Fusarium_sp-PT_009298-RA TMHMM2.0 outside 1 959

# Fusarium_sp-PT_009302-RA Length: 89

# Fusarium_sp-PT_009302-RA Number of predicted TMHs: 1

# Fusarium_sp-PT_009302-RA Exp number of AAs in TMHs: 18.60022

# Fusarium_sp-PT_009302-RA Exp number, first 60 AAs: 10.20877

# Fusarium_sp-PT_009302-RA Total prob of N-in: 0.85702

# Fusarium_sp-PT_009302-RA POSSIBLE N-term signal sequence

Fusarium_sp-PT_009302-RA TMHMM2.0 inside 1 50

Fusarium_sp-PT_009302-RA TMHMM2.0 TMhelix 51 68

Fusarium_sp-PT_009302-RA TMHMM2.0 outside 69 89

# Fusarium_sp-PT_009304-RA Length: 456

# Fusarium_sp-PT_009304-RA Number of predicted TMHs: 7

# Fusarium_sp-PT_009304-RA Exp number of AAs in TMHs: 186.81252

# Fusarium_sp-PT_009304-RA Exp number, first 60 AAs: 13.58391

# Fusarium_sp-PT_009304-RA Total prob of N-in: 0.22935

# Fusarium_sp-PT_009304-RA POSSIBLE N-term signal sequence

Fusarium_sp-PT_009304-RA TMHMM2.0 outside 1 132

Fusarium_sp-PT_009304-RA TMHMM2.0 TMhelix 133 155

Fusarium_sp-PT_009304-RA TMHMM2.0 inside 156 163

Fusarium_sp-PT_009304-RA TMHMM2.0 TMhelix 164 183

Fusarium_sp-PT_009304-RA TMHMM2.0 outside 184 242

Fusarium_sp-PT_009304-RA TMHMM2.0 TMhelix 243 265

Fusarium_sp-PT_009304-RA TMHMM2.0 inside 266 277

Fusarium_sp-PT_009304-RA TMHMM2.0 TMhelix 278 300

Fusarium_sp-PT_009304-RA TMHMM2.0 outside 301 314

Fusarium_sp-PT_009304-RA TMHMM2.0 TMhelix 315 337

Fusarium_sp-PT_009304-RA TMHMM2.0 inside 338 341

Fusarium_sp-PT_009304-RA TMHMM2.0 TMhelix 342 364

Fusarium_sp-PT_009304-RA TMHMM2.0 outside 365 367

Fusarium_sp-PT_009304-RA TMHMM2.0 TMhelix 368 385

Fusarium_sp-PT_009304-RA TMHMM2.0 inside 386 456

# Fusarium_sp-PT_009305-RA Length: 458

# Fusarium_sp-PT_009305-RA Number of predicted TMHs: 1

# Fusarium_sp-PT_009305-RA Exp number of AAs in TMHs: 22.05749

# Fusarium_sp-PT_009305-RA Exp number, first 60 AAs: 22.05538

# Fusarium_sp-PT_009305-RA Total prob of N-in: 0.95059

# Fusarium_sp-PT_009305-RA POSSIBLE N-term signal sequence

Fusarium_sp-PT_009305-RA TMHMM2.0 inside 1 8

Fusarium_sp-PT_009305-RA TMHMM2.0 TMhelix 9 31

Fusarium_sp-PT_009305-RA TMHMM2.0 outside 32 458

# Fusarium_sp-PT_009306-RA Length: 1051

# Fusarium_sp-PT_009306-RA Number of predicted TMHs: 0

# Fusarium_sp-PT_009306-RA Exp number of AAs in TMHs: 0.000880000000000001

# Fusarium_sp-PT_009306-RA Exp number, first 60 AAs: 0

# Fusarium_sp-PT_009306-RA Total prob of N-in: 0.00002

Fusarium_sp-PT_009306-RA TMHMM2.0 outside 1 1051

# Fusarium_sp-PT_009308-RA Length: 346

# Fusarium_sp-PT_009308-RA Number of predicted TMHs: 0

# Fusarium_sp-PT_009308-RA Exp number of AAs in TMHs: 0.04619

# Fusarium_sp-PT_009308-RA Exp number, first 60 AAs: 0

# Fusarium_sp-PT_009308-RA Total prob of N-in: 0.01490

Fusarium_sp-PT_009308-RA TMHMM2.0 outside 1 346

# Fusarium_sp-PT_009310-RA Length: 544

# Fusarium_sp-PT_009310-RA Number of predicted TMHs: 3

# Fusarium_sp-PT_009310-RA Exp number of AAs in TMHs: 85.12601

# Fusarium_sp-PT_009310-RA Exp number, first 60 AAs: 20.93156

# Fusarium_sp-PT_009310-RA Total prob of N-in: 0.50532

# Fusarium_sp-PT_009310-RA POSSIBLE N-term signal sequence

Fusarium_sp-PT_009310-RA TMHMM2.0 inside 1 37

Fusarium_sp-PT_009310-RA TMHMM2.0 TMhelix 38 60

Fusarium_sp-PT_009310-RA TMHMM2.0 outside 61 138

Fusarium_sp-PT_009310-RA TMHMM2.0 TMhelix 139 161

Fusarium_sp-PT_009310-RA TMHMM2.0 inside 162 167

Fusarium_sp-PT_009310-RA TMHMM2.0 TMhelix 168 190

Fusarium_sp-PT_009310-RA TMHMM2.0 outside 191 544

# Fusarium_sp-PT_009315-RA Length: 446

# Fusarium_sp-PT_009315-RA Number of predicted TMHs: 0

# Fusarium_sp-PT_009315-RA Exp number of AAs in TMHs: 0.03737

# Fusarium_sp-PT_009315-RA Exp number, first 60 AAs: 0.0001

# Fusarium_sp-PT_009315-RA Total prob of N-in: 0.00509

Fusarium_sp-PT_009315-RA TMHMM2.0 outside 1 446

# Fusarium_sp-PT_009317-RA Length: 668

# Fusarium_sp-PT_009317-RA Number of predicted TMHs: 0

# Fusarium_sp-PT_009317-RA Exp number of AAs in TMHs: 0.00018

# Fusarium_sp-PT_009317-RA Exp number, first 60 AAs: 0

# Fusarium_sp-PT_009317-RA Total prob of N-in: 0.00015

Fusarium_sp-PT_009317-RA TMHMM2.0 outside 1 668

# Fusarium_sp-PT_009318-RA Length: 862

# Fusarium_sp-PT_009318-RA Number of predicted TMHs: 0

# Fusarium_sp-PT_009318-RA Exp number of AAs in TMHs: 0.03808

# Fusarium_sp-PT_009318-RA Exp number, first 60 AAs: 0

# Fusarium_sp-PT_009318-RA Total prob of N-in: 0.00003

Fusarium_sp-PT_009318-RA TMHMM2.0 outside 1 862

# Fusarium_sp-PT_009320-RA Length: 304

# Fusarium_sp-PT_009320-RA Number of predicted TMHs: 6

# Fusarium_sp-PT_009320-RA Exp number of AAs in TMHs: 113.5588

# Fusarium_sp-PT_009320-RA Exp number, first 60 AAs: 21.03319

# Fusarium_sp-PT_009320-RA Total prob of N-in: 0.93227

# Fusarium_sp-PT_009320-RA POSSIBLE N-term signal sequence

Fusarium_sp-PT_009320-RA TMHMM2.0 inside 1 28

Fusarium_sp-PT_009320-RA TMHMM2.0 TMhelix 29 51

Fusarium_sp-PT_009320-RA TMHMM2.0 outside 52 70

Fusarium_sp-PT_009320-RA TMHMM2.0 TMhelix 71 93

Fusarium_sp-PT_009320-RA TMHMM2.0 inside 94 104

Fusarium_sp-PT_009320-RA TMHMM2.0 TMhelix 105 122

Fusarium_sp-PT_009320-RA TMHMM2.0 outside 123 174

Fusarium_sp-PT_009320-RA TMHMM2.0 TMhelix 175 197

Fusarium_sp-PT_009320-RA TMHMM2.0 inside 198 203

Fusarium_sp-PT_009320-RA TMHMM2.0 TMhelix 204 222

Fusarium_sp-PT_009320-RA TMHMM2.0 outside 223 231

Fusarium_sp-PT_009320-RA TMHMM2.0 TMhelix 232 251

Fusarium_sp-PT_009320-RA TMHMM2.0 inside 252 304

# Fusarium_sp-PT_009322-RA Length: 495

# Fusarium_sp-PT_009322-RA Number of predicted TMHs: 10

# Fusarium_sp-PT_009322-RA Exp number of AAs in TMHs: 236.8037

# Fusarium_sp-PT_009322-RA Exp number, first 60 AAs: 4.72552

# Fusarium_sp-PT_009322-RA Total prob of N-in: 0.87589

Fusarium_sp-PT_009322-RA TMHMM2.0 inside 1 107

Fusarium_sp-PT_009322-RA TMHMM2.0 TMhelix 108 127

Fusarium_sp-PT_009322-RA TMHMM2.0 outside 128 131

Fusarium_sp-PT_009322-RA TMHMM2.0 TMhelix 132 154

Fusarium_sp-PT_009322-RA TMHMM2.0 inside 155 166

Fusarium_sp-PT_009322-RA TMHMM2.0 TMhelix 167 189

Fusarium_sp-PT_009322-RA TMHMM2.0 outside 190 198

Fusarium_sp-PT_009322-RA TMHMM2.0 TMhelix 199 221

Fusarium_sp-PT_009322-RA TMHMM2.0 inside 222 281

Fusarium_sp-PT_009322-RA TMHMM2.0 TMhelix 282 304

Fusarium_sp-PT_009322-RA TMHMM2.0 outside 305 313

Fusarium_sp-PT_009322-RA TMHMM2.0 TMhelix 314 336

Fusarium_sp-PT_009322-RA TMHMM2.0 inside 337 342

Fusarium_sp-PT_009322-RA TMHMM2.0 TMhelix 343 365

Fusarium_sp-PT_009322-RA TMHMM2.0 outside 366 368

Fusarium_sp-PT_009322-RA TMHMM2.0 TMhelix 369 391

Fusarium_sp-PT_009322-RA TMHMM2.0 inside 392 402

Fusarium_sp-PT_009322-RA TMHMM2.0 TMhelix 403 422

Fusarium_sp-PT_009322-RA TMHMM2.0 outside 423 431

Fusarium_sp-PT_009322-RA TMHMM2.0 TMhelix 432 454

Fusarium_sp-PT_009322-RA TMHMM2.0 inside 455 495

# Fusarium_sp-PT_009323-RA Length: 812

# Fusarium_sp-PT_009323-RA Number of predicted TMHs: 0

# Fusarium_sp-PT_009323-RA Exp number of AAs in TMHs: 0.08722

# Fusarium_sp-PT_009323-RA Exp number, first 60 AAs: 0

# Fusarium_sp-PT_009323-RA Total prob of N-in: 0.00016

Fusarium_sp-PT_009323-RA TMHMM2.0 outside 1 812

# Fusarium_sp-PT_009324-RA Length: 534

# Fusarium_sp-PT_009324-RA Number of predicted TMHs: 0

# Fusarium_sp-PT_009324-RA Exp number of AAs in TMHs: 0.0529200000000001

# Fusarium_sp-PT_009324-RA Exp number, first 60 AAs: 0

# Fusarium_sp-PT_009324-RA Total prob of N-in: 0.00301

Fusarium_sp-PT_009324-RA TMHMM2.0 outside 1 534

# Fusarium_sp-PT_009326-RA Length: 632

# Fusarium_sp-PT_009326-RA Number of predicted TMHs: 0

# Fusarium_sp-PT_009326-RA Exp number of AAs in TMHs: 0.56329

# Fusarium_sp-PT_009326-RA Exp number, first 60 AAs: 0.199

# Fusarium_sp-PT_009326-RA Total prob of N-in: 0.02106

Fusarium_sp-PT_009326-RA TMHMM2.0 outside 1 632

# Fusarium_sp-PT_009327-RA Length: 543

# Fusarium_sp-PT_009327-RA Number of predicted TMHs: 0

# Fusarium_sp-PT_009327-RA Exp number of AAs in TMHs: 7.65744999999999

# Fusarium_sp-PT_009327-RA Exp number, first 60 AAs: 7.64587

# Fusarium_sp-PT_009327-RA Total prob of N-in: 0.33168

Fusarium_sp-PT_009327-RA TMHMM2.0 outside 1 543

# Fusarium_sp-PT_009293-RA Length: 129

# Fusarium_sp-PT_009293-RA Number of predicted TMHs: 0

# Fusarium_sp-PT_009293-RA Exp number of AAs in TMHs: 0.02169

# Fusarium_sp-PT_009293-RA Exp number, first 60 AAs: 0

# Fusarium_sp-PT_009293-RA Total prob of N-in: 0.43002

Fusarium_sp-PT_009293-RA TMHMM2.0 outside 1 129

# Fusarium_sp-PT_000001-RA Length: 278

# Fusarium_sp-PT_000001-RA Number of predicted TMHs: 0

# Fusarium_sp-PT_000001-RA Exp number of AAs in TMHs: 0.15401

# Fusarium_sp-PT_000001-RA Exp number, first 60 AAs: 0.14125

# Fusarium_sp-PT_000001-RA Total prob of N-in: 0.04118

Fusarium_sp-PT_000001-RA TMHMM2.0 outside 1 278

# Fusarium_sp-PT_000002-RA Length: 454

# Fusarium_sp-PT_000002-RA Number of predicted TMHs: 9

# Fusarium_sp-PT_000002-RA Exp number of AAs in TMHs: 215.30813

# Fusarium_sp-PT_000002-RA Exp number, first 60 AAs: 3.85611

# Fusarium_sp-PT_000002-RA Total prob of N-in: 0.38435

Fusarium_sp-PT_000002-RA TMHMM2.0 outside 1 83

Fusarium_sp-PT_000002-RA TMHMM2.0 TMhelix 84 106

Fusarium_sp-PT_000002-RA TMHMM2.0 inside 107 118

Fusarium_sp-PT_000002-RA TMHMM2.0 TMhelix 119 141

Fusarium_sp-PT_000002-RA TMHMM2.0 outside 142 173

Fusarium_sp-PT_000002-RA TMHMM2.0 TMhelix 174 196

Fusarium_sp-PT_000002-RA TMHMM2.0 inside 197 207

Fusarium_sp-PT_000002-RA TMHMM2.0 TMhelix 208 230

Fusarium_sp-PT_000002-RA TMHMM2.0 outside 231 287

Fusarium_sp-PT_000002-RA TMHMM2.0 TMhelix 288 310

Fusarium_sp-PT_000002-RA TMHMM2.0 inside 311 316

Fusarium_sp-PT_000002-RA TMHMM2.0 TMhelix 317 339

Fusarium_sp-PT_000002-RA TMHMM2.0 outside 340 343

Fusarium_sp-PT_000002-RA TMHMM2.0 TMhelix 344 363

Fusarium_sp-PT_000002-RA TMHMM2.0 inside 364 375

Fusarium_sp-PT_000002-RA TMHMM2.0 TMhelix 376 398

Fusarium_sp-PT_000002-RA TMHMM2.0 outside 399 407

Fusarium_sp-PT_000002-RA TMHMM2.0 TMhelix 408 430

Fusarium_sp-PT_000002-RA TMHMM2.0 inside 431 454

# Fusarium_sp-PT_000005-RA Length: 556

# Fusarium_sp-PT_000005-RA Number of predicted TMHs: 0

# Fusarium_sp-PT_000005-RA Exp number of AAs in TMHs: 0.00134

# Fusarium_sp-PT_000005-RA Exp number, first 60 AAs: 0

# Fusarium_sp-PT_000005-RA Total prob of N-in: 0.00251

Fusarium_sp-PT_000005-RA TMHMM2.0 outside 1 556

# Fusarium_sp-PT_000007-RA Length: 363

# Fusarium_sp-PT_000007-RA Number of predicted TMHs: 0

# Fusarium_sp-PT_000007-RA Exp number of AAs in TMHs: 0.00802

# Fusarium_sp-PT_000007-RA Exp number, first 60 AAs: 0.00108

# Fusarium_sp-PT_000007-RA Total prob of N-in: 0.04836

Fusarium_sp-PT_000007-RA TMHMM2.0 outside 1 363

# Fusarium_sp-PT_000009-RA Length: 3155

# Fusarium_sp-PT_000009-RA Number of predicted TMHs: 0

# Fusarium_sp-PT_000009-RA Exp number of AAs in TMHs: 0.0035

# Fusarium_sp-PT_000009-RA Exp number, first 60 AAs: 0.00166

# Fusarium_sp-PT_000009-RA Total prob of N-in: 0.00010

Fusarium_sp-PT_000009-RA TMHMM2.0 outside 1 3155

# Fusarium_sp-PT_000013-RA Length: 524

# Fusarium_sp-PT_000013-RA Number of predicted TMHs: 12

# Fusarium_sp-PT_000013-RA Exp number of AAs in TMHs: 258.65302

# Fusarium_sp-PT_000013-RA Exp number, first 60 AAs: 11.91368

# Fusarium_sp-PT_000013-RA Total prob of N-in: 0.96141

# Fusarium_sp-PT_000013-RA POSSIBLE N-term signal sequence

Fusarium_sp-PT_000013-RA TMHMM2.0 inside 1 48

Fusarium_sp-PT_000013-RA TMHMM2.0 TMhelix 49 66

Fusarium_sp-PT_000013-RA TMHMM2.0 outside 67 80

Fusarium_sp-PT_000013-RA TMHMM2.0 TMhelix 81 114

Fusarium_sp-PT_000013-RA TMHMM2.0 inside 115 126

Fusarium_sp-PT_000013-RA TMHMM2.0 TMhelix 127 149

Fusarium_sp-PT_000013-RA TMHMM2.0 outside 150 152

Fusarium_sp-PT_000013-RA TMHMM2.0 TMhelix 153 172

Fusarium_sp-PT_000013-RA TMHMM2.0 inside 173 178

Fusarium_sp-PT_000013-RA TMHMM2.0 TMhelix 179 196

Fusarium_sp-PT_000013-RA TMHMM2.0 outside 197 205

Fusarium_sp-PT_000013-RA TMHMM2.0 TMhelix 206 225

Fusarium_sp-PT_000013-RA TMHMM2.0 inside 226 286

Fusarium_sp-PT_000013-RA TMHMM2.0 TMhelix 287 309

Fusarium_sp-PT_000013-RA TMHMM2.0 outside 310 336

Fusarium_sp-PT_000013-RA TMHMM2.0 TMhelix 337 359

Fusarium_sp-PT_000013-RA TMHMM2.0 inside 360 387

Fusarium_sp-PT_000013-RA TMHMM2.0 TMhelix 388 410

Fusarium_sp-PT_000013-RA TMHMM2.0 outside 411 415

Fusarium_sp-PT_000013-RA TMHMM2.0 TMhelix 416 438

Fusarium_sp-PT_000013-RA TMHMM2.0 inside 439 450

Fusarium_sp-PT_000013-RA TMHMM2.0 TMhelix 451 473

Fusarium_sp-PT_000013-RA TMHMM2.0 outside 474 487

Fusarium_sp-PT_000013-RA TMHMM2.0 TMhelix 488 507

Fusarium_sp-PT_000013-RA TMHMM2.0 inside 508 524

# Fusarium_sp-PT_000014-RA Length: 763

# Fusarium_sp-PT_000014-RA Number of predicted TMHs: 0

# Fusarium_sp-PT_000014-RA Exp number of AAs in TMHs: 3.74763

# Fusarium_sp-PT_000014-RA Exp number, first 60 AAs: 0.00233

# Fusarium_sp-PT_000014-RA Total prob of N-in: 0.00904

Fusarium_sp-PT_000014-RA TMHMM2.0 outside 1 763

# Fusarium_sp-PT_000015-RA Length: 1265

# Fusarium_sp-PT_000015-RA Number of predicted TMHs: 11

# Fusarium_sp-PT_000015-RA Exp number of AAs in TMHs: 254.090460000001

# Fusarium_sp-PT_000015-RA Exp number, first 60 AAs: 14.94681

# Fusarium_sp-PT_000015-RA Total prob of N-in: 0.92543

# Fusarium_sp-PT_000015-RA POSSIBLE N-term signal sequence

Fusarium_sp-PT_000015-RA TMHMM2.0 inside 1 46

Fusarium_sp-PT_000015-RA TMHMM2.0 TMhelix 47 69

Fusarium_sp-PT_000015-RA TMHMM2.0 outside 70 88

Fusarium_sp-PT_000015-RA TMHMM2.0 TMhelix 89 111

Fusarium_sp-PT_000015-RA TMHMM2.0 inside 112 164

Fusarium_sp-PT_000015-RA TMHMM2.0 TMhelix 165 184

Fusarium_sp-PT_000015-RA TMHMM2.0 outside 185 187

Fusarium_sp-PT_000015-RA TMHMM2.0 TMhelix 188 210

Fusarium_sp-PT_000015-RA TMHMM2.0 inside 211 269

Fusarium_sp-PT_000015-RA TMHMM2.0 TMhelix 270 292

Fusarium_sp-PT_000015-RA TMHMM2.0 outside 293 306

Fusarium_sp-PT_000015-RA TMHMM2.0 TMhelix 307 329

Fusarium_sp-PT_000015-RA TMHMM2.0 inside 330 691

Fusarium_sp-PT_000015-RA TMHMM2.0 TMhelix 692 714

Fusarium_sp-PT_000015-RA TMHMM2.0 outside 715 733

Fusarium_sp-PT_000015-RA TMHMM2.0 TMhelix 734 756

Fusarium_sp-PT_000015-RA TMHMM2.0 inside 757 809

Fusarium_sp-PT_000015-RA TMHMM2.0 TMhelix 810 832

Fusarium_sp-PT_000015-RA TMHMM2.0 outside 833 835

Fusarium_sp-PT_000015-RA TMHMM2.0 TMhelix 836 858

Fusarium_sp-PT_000015-RA TMHMM2.0 inside 859 953

Fusarium_sp-PT_000015-RA TMHMM2.0 TMhelix 954 976

Fusarium_sp-PT_000015-RA TMHMM2.0 outside 977 1265

# Fusarium_sp-PT_000016-RA Length: 336

# Fusarium_sp-PT_000016-RA Number of predicted TMHs: 6

# Fusarium_sp-PT_000016-RA Exp number of AAs in TMHs: 137.05494

# Fusarium_sp-PT_000016-RA Exp number, first 60 AAs: 39.10404

# Fusarium_sp-PT_000016-RA Total prob of N-in: 0.15391

# Fusarium_sp-PT_000016-RA POSSIBLE N-term signal sequence

Fusarium_sp-PT_000016-RA TMHMM2.0 outside 1 3

Fusarium_sp-PT_000016-RA TMHMM2.0 TMhelix 4 20

Fusarium_sp-PT_000016-RA TMHMM2.0 inside 21 32

Fusarium_sp-PT_000016-RA TMHMM2.0 TMhelix 33 55

Fusarium_sp-PT_000016-RA TMHMM2.0 outside 56 74

Fusarium_sp-PT_000016-RA TMHMM2.0 TMhelix 75 97

Fusarium_sp-PT_000016-RA TMHMM2.0 inside 98 109

Fusarium_sp-PT_000016-RA TMHMM2.0 TMhelix 110 132

Fusarium_sp-PT_000016-RA TMHMM2.0 outside 133 155

Fusarium_sp-PT_000016-RA TMHMM2.0 TMhelix 156 178

Fusarium_sp-PT_000016-RA TMHMM2.0 inside 179 190

Fusarium_sp-PT_000016-RA TMHMM2.0 TMhelix 191 213

Fusarium_sp-PT_000016-RA TMHMM2.0 outside 214 336

# Fusarium_sp-PT_000018-RA Length: 1251

# Fusarium_sp-PT_000018-RA Number of predicted TMHs: 0

# Fusarium_sp-PT_000018-RA Exp number of AAs in TMHs: 0.17554

# Fusarium_sp-PT_000018-RA Exp number, first 60 AAs: 0.08964

# Fusarium_sp-PT_000018-RA Total prob of N-in: 0.00439

Fusarium_sp-PT_000018-RA TMHMM2.0 outside 1 1251

# Fusarium_sp-PT_000021-RA Length: 1446

# Fusarium_sp-PT_000021-RA Number of predicted TMHs: 0

# Fusarium_sp-PT_000021-RA Exp number of AAs in TMHs: 0.368190000000001

# Fusarium_sp-PT_000021-RA Exp number, first 60 AAs: 0.27918

# Fusarium_sp-PT_000021-RA Total prob of N-in: 0.01595

Fusarium_sp-PT_000021-RA TMHMM2.0 outside 1 1446

# Fusarium_sp-PT_000003-RA Length: 312

# Fusarium_sp-PT_000003-RA Number of predicted TMHs: 3

# Fusarium_sp-PT_000003-RA Exp number of AAs in TMHs: 62.1179500000001

# Fusarium_sp-PT_000003-RA Exp number, first 60 AAs: 36.16575

# Fusarium_sp-PT_000003-RA Total prob of N-in: 0.99768

# Fusarium_sp-PT_000003-RA POSSIBLE N-term signal sequence

Fusarium_sp-PT_000003-RA TMHMM2.0 inside 1 11

Fusarium_sp-PT_000003-RA TMHMM2.0 TMhelix 12 34

Fusarium_sp-PT_000003-RA TMHMM2.0 outside 35 48

Fusarium_sp-PT_000003-RA TMHMM2.0 TMhelix 49 66

Fusarium_sp-PT_000003-RA TMHMM2.0 inside 67 86

Fusarium_sp-PT_000003-RA TMHMM2.0 TMhelix 87 104

Fusarium_sp-PT_000003-RA TMHMM2.0 outside 105 312

# Fusarium_sp-PT_000004-RA Length: 121

# Fusarium_sp-PT_000004-RA Number of predicted TMHs: 0

# Fusarium_sp-PT_000004-RA Exp number of AAs in TMHs: 1.76301

# Fusarium_sp-PT_000004-RA Exp number, first 60 AAs: 0.00671

# Fusarium_sp-PT_000004-RA Total prob of N-in: 0.30606

Fusarium_sp-PT_000004-RA TMHMM2.0 outside 1 121

# Fusarium_sp-PT_000006-RA Length: 557

# Fusarium_sp-PT_000006-RA Number of predicted TMHs: 10

# Fusarium_sp-PT_000006-RA Exp number of AAs in TMHs: 255.36604

# Fusarium_sp-PT_000006-RA Exp number, first 60 AAs: 10.41861

# Fusarium_sp-PT_000006-RA Total prob of N-in: 0.71445

# Fusarium_sp-PT_000006-RA POSSIBLE N-term signal sequence

Fusarium_sp-PT_000006-RA TMHMM2.0 inside 1 132

Fusarium_sp-PT_000006-RA TMHMM2.0 TMhelix 133 155

Fusarium_sp-PT_000006-RA TMHMM2.0 outside 156 169

Fusarium_sp-PT_000006-RA TMHMM2.0 TMhelix 170 192

Fusarium_sp-PT_000006-RA TMHMM2.0 inside 193 204

Fusarium_sp-PT_000006-RA TMHMM2.0 TMhelix 205 227

Fusarium_sp-PT_000006-RA TMHMM2.0 outside 228 241

Fusarium_sp-PT_000006-RA TMHMM2.0 TMhelix 242 264

Fusarium_sp-PT_000006-RA TMHMM2.0 inside 265 275

Fusarium_sp-PT_000006-RA TMHMM2.0 TMhelix 276 298

Fusarium_sp-PT_000006-RA TMHMM2.0 outside 299 328

Fusarium_sp-PT_000006-RA TMHMM2.0 TMhelix 329 351

Fusarium_sp-PT_000006-RA TMHMM2.0 inside 352 373

Fusarium_sp-PT_000006-RA TMHMM2.0 TMhelix 374 396

Fusarium_sp-PT_000006-RA TMHMM2.0 outside 397 399

Fusarium_sp-PT_000006-RA TMHMM2.0 TMhelix 400 422

Fusarium_sp-PT_000006-RA TMHMM2.0 inside 423 450

Fusarium_sp-PT_000006-RA TMHMM2.0 TMhelix 451 473

Fusarium_sp-PT_000006-RA TMHMM2.0 outside 474 482

Fusarium_sp-PT_000006-RA TMHMM2.0 TMhelix 483 505

Fusarium_sp-PT_000006-RA TMHMM2.0 inside 506 557

# Fusarium_sp-PT_000011-RA Length: 91

# Fusarium_sp-PT_000011-RA Number of predicted TMHs: 0

# Fusarium_sp-PT_000011-RA Exp number of AAs in TMHs: 0

# Fusarium_sp-PT_000011-RA Exp number, first 60 AAs: 0

# Fusarium_sp-PT_000011-RA Total prob of N-in: 0.48992

Fusarium_sp-PT_000011-RA TMHMM2.0 outside 1 91

# Fusarium_sp-PT_000012-RA Length: 534

# Fusarium_sp-PT_000012-RA Number of predicted TMHs: 1

# Fusarium_sp-PT_000012-RA Exp number of AAs in TMHs: 21.0238

# Fusarium_sp-PT_000012-RA Exp number, first 60 AAs: 20.99697

# Fusarium_sp-PT_000012-RA Total prob of N-in: 0.91373

# Fusarium_sp-PT_000012-RA POSSIBLE N-term signal sequence

Fusarium_sp-PT_000012-RA TMHMM2.0 inside 1 18

Fusarium_sp-PT_000012-RA TMHMM2.0 TMhelix 19 41

Fusarium_sp-PT_000012-RA TMHMM2.0 outside 42 534

# Fusarium_sp-PT_000017-RA Length: 578

# Fusarium_sp-PT_000017-RA Number of predicted TMHs: 1

# Fusarium_sp-PT_000017-RA Exp number of AAs in TMHs: 22.06498

# Fusarium_sp-PT_000017-RA Exp number, first 60 AAs: 21.72788

# Fusarium_sp-PT_000017-RA Total prob of N-in: 0.98111

# Fusarium_sp-PT_000017-RA POSSIBLE N-term signal sequence

Fusarium_sp-PT_000017-RA TMHMM2.0 inside 1 12

Fusarium_sp-PT_000017-RA TMHMM2.0 TMhelix 13 35

Fusarium_sp-PT_000017-RA TMHMM2.0 outside 36 578

# Fusarium_sp-PT_000019-RA Length: 539

# Fusarium_sp-PT_000019-RA Number of predicted TMHs: 0

# Fusarium_sp-PT_000019-RA Exp number of AAs in TMHs: 9.10249999999997

# Fusarium_sp-PT_000019-RA Exp number, first 60 AAs: 9.07014

# Fusarium_sp-PT_000019-RA Total prob of N-in: 0.41873

Fusarium_sp-PT_000019-RA TMHMM2.0 outside 1 539

# Fusarium_sp-PT_000020-RA Length: 390

# Fusarium_sp-PT_000020-RA Number of predicted TMHs: 0

# Fusarium_sp-PT_000020-RA Exp number of AAs in TMHs: 0.588659999999998

# Fusarium_sp-PT_000020-RA Exp number, first 60 AAs: 0.57493

# Fusarium_sp-PT_000020-RA Total prob of N-in: 0.03224

Fusarium_sp-PT_000020-RA TMHMM2.0 outside 1 390

# Fusarium_sp-PT_000008-RA Length: 404

# Fusarium_sp-PT_000008-RA Number of predicted TMHs: 0

# Fusarium_sp-PT_000008-RA Exp number of AAs in TMHs: 0.18438

# Fusarium_sp-PT_000008-RA Exp number, first 60 AAs: 0

# Fusarium_sp-PT_000008-RA Total prob of N-in: 0.00485

Fusarium_sp-PT_000008-RA TMHMM2.0 outside 1 404

# Fusarium_sp-PT_000010-RA Length: 47

# Fusarium_sp-PT_000010-RA Number of predicted TMHs: 0

# Fusarium_sp-PT_000010-RA Exp number of AAs in TMHs: 0

# Fusarium_sp-PT_000010-RA Exp number, first 60 AAs: 0

# Fusarium_sp-PT_000010-RA Total prob of N-in: 0.45869

Fusarium_sp-PT_000010-RA TMHMM2.0 inside 1 47

# Fusarium_sp-PT_000023-RA Length: 81

# Fusarium_sp-PT_000023-RA Number of predicted TMHs: 0

# Fusarium_sp-PT_000023-RA Exp number of AAs in TMHs: 0.00245

# Fusarium_sp-PT_000023-RA Exp number, first 60 AAs: 0.00112

# Fusarium_sp-PT_000023-RA Total prob of N-in: 0.25160

Fusarium_sp-PT_000023-RA TMHMM2.0 outside 1 81

# Fusarium_sp-PT_000024-RA Length: 2499

# Fusarium_sp-PT_000024-RA Number of predicted TMHs: 0

# Fusarium_sp-PT_000024-RA Exp number of AAs in TMHs: 0.50335

# Fusarium_sp-PT_000024-RA Exp number, first 60 AAs: 0.00116

# Fusarium_sp-PT_000024-RA Total prob of N-in: 0.00049

Fusarium_sp-PT_000024-RA TMHMM2.0 outside 1 2499

# Fusarium_sp-PT_000027-RA Length: 116

# Fusarium_sp-PT_000027-RA Number of predicted TMHs: 0

# Fusarium_sp-PT_000027-RA Exp number of AAs in TMHs: 0.29591

# Fusarium_sp-PT_000027-RA Exp number, first 60 AAs: 0.29591

# Fusarium_sp-PT_000027-RA Total prob of N-in: 0.10272

Fusarium_sp-PT_000027-RA TMHMM2.0 outside 1 116

# Fusarium_sp-PT_000029-RA Length: 394

# Fusarium_sp-PT_000029-RA Number of predicted TMHs: 0

# Fusarium_sp-PT_000029-RA Exp number of AAs in TMHs: 0.07616

# Fusarium_sp-PT_000029-RA Exp number, first 60 AAs: 0.00509

# Fusarium_sp-PT_000029-RA Total prob of N-in: 0.00532

Fusarium_sp-PT_000029-RA TMHMM2.0 outside 1 394

# Fusarium_sp-PT_000034-RA Length: 415

# Fusarium_sp-PT_000034-RA Number of predicted TMHs: 0

# Fusarium_sp-PT_000034-RA Exp number of AAs in TMHs: 0.16808

# Fusarium_sp-PT_000034-RA Exp number, first 60 AAs: 0

# Fusarium_sp-PT_000034-RA Total prob of N-in: 0.00364

Fusarium_sp-PT_000034-RA TMHMM2.0 outside 1 415

# Fusarium_sp-PT_000036-RA Length: 449

# Fusarium_sp-PT_000036-RA Number of predicted TMHs: 0

# Fusarium_sp-PT_000036-RA Exp number of AAs in TMHs: 4.05285999999999

# Fusarium_sp-PT_000036-RA Exp number, first 60 AAs: 0.00013

# Fusarium_sp-PT_000036-RA Total prob of N-in: 0.13210

Fusarium_sp-PT_000036-RA TMHMM2.0 outside 1 449

# Fusarium_sp-PT_000037-RA Length: 831

# Fusarium_sp-PT_000037-RA Number of predicted TMHs: 0

# Fusarium_sp-PT_000037-RA Exp number of AAs in TMHs: 0.01144

# Fusarium_sp-PT_000037-RA Exp number, first 60 AAs: 0

# Fusarium_sp-PT_000037-RA Total prob of N-in: 0.00022

Fusarium_sp-PT_000037-RA TMHMM2.0 outside 1 831

# Fusarium_sp-PT_000038-RA Length: 516

# Fusarium_sp-PT_000038-RA Number of predicted TMHs: 10

# Fusarium_sp-PT_000038-RA Exp number of AAs in TMHs: 231.6089

# Fusarium_sp-PT_000038-RA Exp number, first 60 AAs: 19.25758

# Fusarium_sp-PT_000038-RA Total prob of N-in: 0.98699

# Fusarium_sp-PT_000038-RA POSSIBLE N-term signal sequence

Fusarium_sp-PT_000038-RA TMHMM2.0 inside 1 32

Fusarium_sp-PT_000038-RA TMHMM2.0 TMhelix 33 55

Fusarium_sp-PT_000038-RA TMHMM2.0 outside 56 74

Fusarium_sp-PT_000038-RA TMHMM2.0 TMhelix 75 97

Fusarium_sp-PT_000038-RA TMHMM2.0 inside 98 129

Fusarium_sp-PT_000038-RA TMHMM2.0 TMhelix 130 152

Fusarium_sp-PT_000038-RA TMHMM2.0 outside 153 161

Fusarium_sp-PT_000038-RA TMHMM2.0 TMhelix 162 184

Fusarium_sp-PT_000038-RA TMHMM2.0 inside 185 288

Fusarium_sp-PT_000038-RA TMHMM2.0 TMhelix 289 311

Fusarium_sp-PT_000038-RA TMHMM2.0 outside 312 325

Fusarium_sp-PT_000038-RA TMHMM2.0 TMhelix 326 348

Fusarium_sp-PT_000038-RA TMHMM2.0 inside 349 354

Fusarium_sp-PT_000038-RA TMHMM2.0 TMhelix 355 377

Fusarium_sp-PT_000038-RA TMHMM2.0 outside 378 391

Fusarium_sp-PT_000038-RA TMHMM2.0 TMhelix 392 414

Fusarium_sp-PT_000038-RA TMHMM2.0 inside 415 426

Fusarium_sp-PT_000038-RA TMHMM2.0 TMhelix 427 449

Fusarium_sp-PT_000038-RA TMHMM2.0 outside 450 452

Fusarium_sp-PT_000038-RA TMHMM2.0 TMhelix 453 475

Fusarium_sp-PT_000038-RA TMHMM2.0 inside 476 516

# Fusarium_sp-PT_000040-RA Length: 331

# Fusarium_sp-PT_000040-RA Number of predicted TMHs: 0

# Fusarium_sp-PT_000040-RA Exp number of AAs in TMHs: 0.49516

# Fusarium_sp-PT_000040-RA Exp number, first 60 AAs: 0.30324

# Fusarium_sp-PT_000040-RA Total prob of N-in: 0.04362

Fusarium_sp-PT_000040-RA TMHMM2.0 outside 1 331

# Fusarium_sp-PT_000041-RA Length: 427

# Fusarium_sp-PT_000041-RA Number of predicted TMHs: 0

# Fusarium_sp-PT_000041-RA Exp number of AAs in TMHs: 0.0345

# Fusarium_sp-PT_000041-RA Exp number, first 60 AAs: 0.00094

# Fusarium_sp-PT_000041-RA Total prob of N-in: 0.00363

Fusarium_sp-PT_000041-RA TMHMM2.0 outside 1 427

# Fusarium_sp-PT_000043-RA Length: 316

# Fusarium_sp-PT_000043-RA Number of predicted TMHs: 0

# Fusarium_sp-PT_000043-RA Exp number of AAs in TMHs: 0.24876

# Fusarium_sp-PT_000043-RA Exp number, first 60 AAs: 0.0164

# Fusarium_sp-PT_000043-RA Total prob of N-in: 0.02333

Fusarium_sp-PT_000043-RA TMHMM2.0 outside 1 316

# Fusarium_sp-PT_000044-RA Length: 110

# Fusarium_sp-PT_000044-RA Number of predicted TMHs: 0

# Fusarium_sp-PT_000044-RA Exp number of AAs in TMHs: 0.00019

# Fusarium_sp-PT_000044-RA Exp number, first 60 AAs: 0.00019

# Fusarium_sp-PT_000044-RA Total prob of N-in: 0.30343

Fusarium_sp-PT_000044-RA TMHMM2.0 outside 1 110

# Fusarium_sp-PT_000047-RA Length: 321

# Fusarium_sp-PT_000047-RA Number of predicted TMHs: 0

# Fusarium_sp-PT_000047-RA Exp number of AAs in TMHs: 0.0014

# Fusarium_sp-PT_000047-RA Exp number, first 60 AAs: 0

# Fusarium_sp-PT_000047-RA Total prob of N-in: 0.04683

Fusarium_sp-PT_000047-RA TMHMM2.0 outside 1 321

# Fusarium_sp-PT_000051-RA Length: 185

# Fusarium_sp-PT_000051-RA Number of predicted TMHs: 0

# Fusarium_sp-PT_000051-RA Exp number of AAs in TMHs: 0.04278

# Fusarium_sp-PT_000051-RA Exp number, first 60 AAs: 0.00079

# Fusarium_sp-PT_000051-RA Total prob of N-in: 0.11115

Fusarium_sp-PT_000051-RA TMHMM2.0 outside 1 185

# Fusarium_sp-PT_000022-RA Length: 882

# Fusarium_sp-PT_000022-RA Number of predicted TMHs: 2

# Fusarium_sp-PT_000022-RA Exp number of AAs in TMHs: 42.9709200000001

# Fusarium_sp-PT_000022-RA Exp number, first 60 AAs: 6.41902

# Fusarium_sp-PT_000022-RA Total prob of N-in: 0.00760

Fusarium_sp-PT_000022-RA TMHMM2.0 outside 1 52

Fusarium_sp-PT_000022-RA TMHMM2.0 TMhelix 53 75

Fusarium_sp-PT_000022-RA TMHMM2.0 inside 76 81

Fusarium_sp-PT_000022-RA TMHMM2.0 TMhelix 82 104

Fusarium_sp-PT_000022-RA TMHMM2.0 outside 105 882

# Fusarium_sp-PT_000025-RA Length: 530

# Fusarium_sp-PT_000025-RA Number of predicted TMHs: 8

# Fusarium_sp-PT_000025-RA Exp number of AAs in TMHs: 200.43038

# Fusarium_sp-PT_000025-RA Exp number, first 60 AAs: 0.03607

# Fusarium_sp-PT_000025-RA Total prob of N-in: 0.83110

Fusarium_sp-PT_000025-RA TMHMM2.0 inside 1 190

Fusarium_sp-PT_000025-RA TMHMM2.0 TMhelix 191 213

Fusarium_sp-PT_000025-RA TMHMM2.0 outside 214 217

Fusarium_sp-PT_000025-RA TMHMM2.0 TMhelix 218 240

Fusarium_sp-PT_000025-RA TMHMM2.0 inside 241 295

Fusarium_sp-PT_000025-RA TMHMM2.0 TMhelix 296 318

Fusarium_sp-PT_000025-RA TMHMM2.0 outside 319 332

Fusarium_sp-PT_000025-RA TMHMM2.0 TMhelix 333 355

Fusarium_sp-PT_000025-RA TMHMM2.0 inside 356 374

Fusarium_sp-PT_000025-RA TMHMM2.0 TMhelix 375 397

Fusarium_sp-PT_000025-RA TMHMM2.0 outside 398 401

Fusarium_sp-PT_000025-RA TMHMM2.0 TMhelix 402 424

Fusarium_sp-PT_000025-RA TMHMM2.0 inside 425 430

Fusarium_sp-PT_000025-RA TMHMM2.0 TMhelix 431 453

Fusarium_sp-PT_000025-RA TMHMM2.0 outside 454 472

Fusarium_sp-PT_000025-RA TMHMM2.0 TMhelix 473 495

Fusarium_sp-PT_000025-RA TMHMM2.0 inside 496 530

# Fusarium_sp-PT_000026-RA Length: 300

# Fusarium_sp-PT_000026-RA Number of predicted TMHs: 1

# Fusarium_sp-PT_000026-RA Exp number of AAs in TMHs: 22.59748

# Fusarium_sp-PT_000026-RA Exp number, first 60 AAs: 21.74844

# Fusarium_sp-PT_000026-RA Total prob of N-in: 0.93284

# Fusarium_sp-PT_000026-RA POSSIBLE N-term signal sequence

Fusarium_sp-PT_000026-RA TMHMM2.0 inside 1 38

Fusarium_sp-PT_000026-RA TMHMM2.0 TMhelix 39 61

Fusarium_sp-PT_000026-RA TMHMM2.0 outside 62 300

# Fusarium_sp-PT_000030-RA Length: 591

# Fusarium_sp-PT_000030-RA Number of predicted TMHs: 0

# Fusarium_sp-PT_000030-RA Exp number of AAs in TMHs: 0.00017

# Fusarium_sp-PT_000030-RA Exp number, first 60 AAs: 0

# Fusarium_sp-PT_000030-RA Total prob of N-in: 0.00030

Fusarium_sp-PT_000030-RA TMHMM2.0 outside 1 591

# Fusarium_sp-PT_000031-RA Length: 390

# Fusarium_sp-PT_000031-RA Number of predicted TMHs: 0

# Fusarium_sp-PT_000031-RA Exp number of AAs in TMHs: 0.000760000000000001

# Fusarium_sp-PT_000031-RA Exp number, first 60 AAs: 0

# Fusarium_sp-PT_000031-RA Total prob of N-in: 0.01558

Fusarium_sp-PT_000031-RA TMHMM2.0 outside 1 390

# Fusarium_sp-PT_000033-RA Length: 337

# Fusarium_sp-PT_000033-RA Number of predicted TMHs: 0

# Fusarium_sp-PT_000033-RA Exp number of AAs in TMHs: 0.39949

# Fusarium_sp-PT_000033-RA Exp number, first 60 AAs: 0.00308

# Fusarium_sp-PT_000033-RA Total prob of N-in: 0.11057

Fusarium_sp-PT_000033-RA TMHMM2.0 outside 1 337

# Fusarium_sp-PT_000035-RA Length: 851

# Fusarium_sp-PT_000035-RA Number of predicted TMHs: 0

# Fusarium_sp-PT_000035-RA Exp number of AAs in TMHs: 0.20362

# Fusarium_sp-PT_000035-RA Exp number, first 60 AAs: 0.00018

# Fusarium_sp-PT_000035-RA Total prob of N-in: 0.00919

Fusarium_sp-PT_000035-RA TMHMM2.0 outside 1 851

# Fusarium_sp-PT_000039-RA Length: 256

# Fusarium_sp-PT_000039-RA Number of predicted TMHs: 0

# Fusarium_sp-PT_000039-RA Exp number of AAs in TMHs: 0.0759399999999999

# Fusarium_sp-PT_000039-RA Exp number, first 60 AAs: 0.01559

# Fusarium_sp-PT_000039-RA Total prob of N-in: 0.03400

Fusarium_sp-PT_000039-RA TMHMM2.0 outside 1 256

# Fusarium_sp-PT_000042-RA Length: 311

# Fusarium_sp-PT_000042-RA Number of predicted TMHs: 0

# Fusarium_sp-PT_000042-RA Exp number of AAs in TMHs: 0.0702299999999999

# Fusarium_sp-PT_000042-RA Exp number, first 60 AAs: 0.01752

# Fusarium_sp-PT_000042-RA Total prob of N-in: 0.02454

Fusarium_sp-PT_000042-RA TMHMM2.0 outside 1 311

# Fusarium_sp-PT_000046-RA Length: 513

# Fusarium_sp-PT_000046-RA Number of predicted TMHs: 0

# Fusarium_sp-PT_000046-RA Exp number of AAs in TMHs: 0.00391

# Fusarium_sp-PT_000046-RA Exp number, first 60 AAs: 0

# Fusarium_sp-PT_000046-RA Total prob of N-in: 0.01329

Fusarium_sp-PT_000046-RA TMHMM2.0 outside 1 513

# Fusarium_sp-PT_000048-RA Length: 431

# Fusarium_sp-PT_000048-RA Number of predicted TMHs: 0

# Fusarium_sp-PT_000048-RA Exp number of AAs in TMHs: 2.65689000000001

# Fusarium_sp-PT_000048-RA Exp number, first 60 AAs: 2e-05

# Fusarium_sp-PT_000048-RA Total prob of N-in: 0.14148

Fusarium_sp-PT_000048-RA TMHMM2.0 outside 1 431

# Fusarium_sp-PT_000049-RA Length: 432

# Fusarium_sp-PT_000049-RA Number of predicted TMHs: 0

# Fusarium_sp-PT_000049-RA Exp number of AAs in TMHs: 0.330730000000001

# Fusarium_sp-PT_000049-RA Exp number, first 60 AAs: 0.00037

# Fusarium_sp-PT_000049-RA Total prob of N-in: 0.02651

Fusarium_sp-PT_000049-RA TMHMM2.0 outside 1 432

# Fusarium_sp-PT_000050-RA Length: 516

# Fusarium_sp-PT_000050-RA Number of predicted TMHs: 0

# Fusarium_sp-PT_000050-RA Exp number of AAs in TMHs: 0.04874

# Fusarium_sp-PT_000050-RA Exp number, first 60 AAs: 0

# Fusarium_sp-PT_000050-RA Total prob of N-in: 0.01363

Fusarium_sp-PT_000050-RA TMHMM2.0 outside 1 516

# Fusarium_sp-PT_000053-RA Length: 433

# Fusarium_sp-PT_000053-RA Number of predicted TMHs: 0

# Fusarium_sp-PT_000053-RA Exp number of AAs in TMHs: 4.01841

# Fusarium_sp-PT_000053-RA Exp number, first 60 AAs: 0.0087

# Fusarium_sp-PT_000053-RA Total prob of N-in: 0.07412

Fusarium_sp-PT_000053-RA TMHMM2.0 outside 1 433

# Fusarium_sp-PT_000028-RA Length: 462

# Fusarium_sp-PT_000028-RA Number of predicted TMHs: 10

# Fusarium_sp-PT_000028-RA Exp number of AAs in TMHs: 227.90067

# Fusarium_sp-PT_000028-RA Exp number, first 60 AAs: 21.44644

# Fusarium_sp-PT_000028-RA Total prob of N-in: 0.86788

# Fusarium_sp-PT_000028-RA POSSIBLE N-term signal sequence

Fusarium_sp-PT_000028-RA TMHMM2.0 inside 1 33

Fusarium_sp-PT_000028-RA TMHMM2.0 TMhelix 34 56

Fusarium_sp-PT_000028-RA TMHMM2.0 outside 57 70

Fusarium_sp-PT_000028-RA TMHMM2.0 TMhelix 71 93

Fusarium_sp-PT_000028-RA TMHMM2.0 inside 94 99

Fusarium_sp-PT_000028-RA TMHMM2.0 TMhelix 100 122

Fusarium_sp-PT_000028-RA TMHMM2.0 outside 123 125

Fusarium_sp-PT_000028-RA TMHMM2.0 TMhelix 126 148

Fusarium_sp-PT_000028-RA TMHMM2.0 inside 149 159

Fusarium_sp-PT_000028-RA TMHMM2.0 TMhelix 160 182

Fusarium_sp-PT_000028-RA TMHMM2.0 outside 183 186

Fusarium_sp-PT_000028-RA TMHMM2.0 TMhelix 187 209

Fusarium_sp-PT_000028-RA TMHMM2.0 inside 210 253

Fusarium_sp-PT_000028-RA TMHMM2.0 TMhelix 254 276

Fusarium_sp-PT_000028-RA TMHMM2.0 outside 277 290

Fusarium_sp-PT_000028-RA TMHMM2.0 TMhelix 291 313

Fusarium_sp-PT_000028-RA TMHMM2.0 inside 314 378

Fusarium_sp-PT_000028-RA TMHMM2.0 TMhelix 379 401

Fusarium_sp-PT_000028-RA TMHMM2.0 outside 402 438

Fusarium_sp-PT_000028-RA TMHMM2.0 TMhelix 439 461

Fusarium_sp-PT_000028-RA TMHMM2.0 inside 462 462

# Fusarium_sp-PT_000032-RA Length: 374

# Fusarium_sp-PT_000032-RA Number of predicted TMHs: 0

# Fusarium_sp-PT_000032-RA Exp number of AAs in TMHs: 0.657649999999999

# Fusarium_sp-PT_000032-RA Exp number, first 60 AAs: 0.61517

# Fusarium_sp-PT_000032-RA Total prob of N-in: 0.06078

Fusarium_sp-PT_000032-RA TMHMM2.0 outside 1 374

# Fusarium_sp-PT_000045-RA Length: 256

# Fusarium_sp-PT_000045-RA Number of predicted TMHs: 0

# Fusarium_sp-PT_000045-RA Exp number of AAs in TMHs: 0

# Fusarium_sp-PT_000045-RA Exp number, first 60 AAs: 0

# Fusarium_sp-PT_000045-RA Total prob of N-in: 0.03303

Fusarium_sp-PT_000045-RA TMHMM2.0 outside 1 256

# Fusarium_sp-PT_000052-RA Length: 110

# Fusarium_sp-PT_000052-RA Number of predicted TMHs: 0

# Fusarium_sp-PT_000052-RA Exp number of AAs in TMHs: 0.00314

# Fusarium_sp-PT_000052-RA Exp number, first 60 AAs: 0.00312

# Fusarium_sp-PT_000052-RA Total prob of N-in: 0.08298

Fusarium_sp-PT_000052-RA TMHMM2.0 outside 1 110

# Fusarium_sp-PT_000054-RA Length: 659

# Fusarium_sp-PT_000054-RA Number of predicted TMHs: 0

# Fusarium_sp-PT_000054-RA Exp number of AAs in TMHs: 1.71666

# Fusarium_sp-PT_000054-RA Exp number, first 60 AAs: 0

# Fusarium_sp-PT_000054-RA Total prob of N-in: 0.00010

Fusarium_sp-PT_000054-RA TMHMM2.0 outside 1 659

# Fusarium_sp-PT_000058-RA Length: 467

# Fusarium_sp-PT_000058-RA Number of predicted TMHs: 0

# Fusarium_sp-PT_000058-RA Exp number of AAs in TMHs: 0.0754800000000002

# Fusarium_sp-PT_000058-RA Exp number, first 60 AAs: 0.06338

# Fusarium_sp-PT_000058-RA Total prob of N-in: 0.00377

Fusarium_sp-PT_000058-RA TMHMM2.0 outside 1 467

# Fusarium_sp-PT_000066-RA Length: 428

# Fusarium_sp-PT_000066-RA Number of predicted TMHs: 0

# Fusarium_sp-PT_000066-RA Exp number of AAs in TMHs: 39.67856

# Fusarium_sp-PT_000066-RA Exp number, first 60 AAs: 3.00837

# Fusarium_sp-PT_000066-RA Total prob of N-in: 0.73352

Fusarium_sp-PT_000066-RA TMHMM2.0 outside 1 428

# Fusarium_sp-PT_000068-RA Length: 685

# Fusarium_sp-PT_000068-RA Number of predicted TMHs: 0

# Fusarium_sp-PT_000068-RA Exp number of AAs in TMHs: 0.12017

# Fusarium_sp-PT_000068-RA Exp number, first 60 AAs: 0

# Fusarium_sp-PT_000068-RA Total prob of N-in: 0.00033

Fusarium_sp-PT_000068-RA TMHMM2.0 outside 1 685

# Fusarium_sp-PT_000071-RA Length: 689

# Fusarium_sp-PT_000071-RA Number of predicted TMHs: 0

# Fusarium_sp-PT_000071-RA Exp number of AAs in TMHs: 12.23481

# Fusarium_sp-PT_000071-RA Exp number, first 60 AAs: 5.16112

# Fusarium_sp-PT_000071-RA Total prob of N-in: 0.19131

Fusarium_sp-PT_000071-RA TMHMM2.0 outside 1 689

# Fusarium_sp-PT_000072-RA Length: 167

# Fusarium_sp-PT_000072-RA Number of predicted TMHs: 0

# Fusarium_sp-PT_000072-RA Exp number of AAs in TMHs: 0.00931

# Fusarium_sp-PT_000072-RA Exp number, first 60 AAs: 0.00164

# Fusarium_sp-PT_000072-RA Total prob of N-in: 0.37851

Fusarium_sp-PT_000072-RA TMHMM2.0 outside 1 167

# Fusarium_sp-PT_000056-RA Length: 619

# Fusarium_sp-PT_000056-RA Number of predicted TMHs: 0

# Fusarium_sp-PT_000056-RA Exp number of AAs in TMHs: 0.0208

# Fusarium_sp-PT_000056-RA Exp number, first 60 AAs: 0

# Fusarium_sp-PT_000056-RA Total prob of N-in: 0.00051

Fusarium_sp-PT_000056-RA TMHMM2.0 outside 1 619

# Fusarium_sp-PT_000059-RA Length: 524

# Fusarium_sp-PT_000059-RA Number of predicted TMHs: 12

# Fusarium_sp-PT_000059-RA Exp number of AAs in TMHs: 250.52362

# Fusarium_sp-PT_000059-RA Exp number, first 60 AAs: 2.61728

# Fusarium_sp-PT_000059-RA Total prob of N-in: 0.99297

Fusarium_sp-PT_000059-RA TMHMM2.0 inside 1 57

Fusarium_sp-PT_000059-RA TMHMM2.0 TMhelix 58 75

Fusarium_sp-PT_000059-RA TMHMM2.0 outside 76 105

Fusarium_sp-PT_000059-RA TMHMM2.0 TMhelix 106 128

Fusarium_sp-PT_000059-RA TMHMM2.0 inside 129 139

Fusarium_sp-PT_000059-RA TMHMM2.0 TMhelix 140 162

Fusarium_sp-PT_000059-RA TMHMM2.0 outside 163 165

Fusarium_sp-PT_000059-RA TMHMM2.0 TMhelix 166 183

Fusarium_sp-PT_000059-RA TMHMM2.0 inside 184 189

Fusarium_sp-PT_000059-RA TMHMM2.0 TMhelix 190 209

Fusarium_sp-PT_000059-RA TMHMM2.0 outside 210 223

Fusarium_sp-PT_000059-RA TMHMM2.0 TMhelix 224 246

Fusarium_sp-PT_000059-RA TMHMM2.0 inside 247 310

Fusarium_sp-PT_000059-RA TMHMM2.0 TMhelix 311 333

Fusarium_sp-PT_000059-RA TMHMM2.0 outside 334 347

Fusarium_sp-PT_000059-RA TMHMM2.0 TMhelix 348 370

Fusarium_sp-PT_000059-RA TMHMM2.0 inside 371 376

Fusarium_sp-PT_000059-RA TMHMM2.0 TMhelix 377 399

Fusarium_sp-PT_000059-RA TMHMM2.0 outside 400 408

Fusarium_sp-PT_000059-RA TMHMM2.0 TMhelix 409 431

Fusarium_sp-PT_000059-RA TMHMM2.0 inside 432 443

Fusarium_sp-PT_000059-RA TMHMM2.0 TMhelix 444 466

Fusarium_sp-PT_000059-RA TMHMM2.0 outside 467 475

Fusarium_sp-PT_000059-RA TMHMM2.0 TMhelix 476 498

Fusarium_sp-PT_000059-RA TMHMM2.0 inside 499 524

# Fusarium_sp-PT_000061-RA Length: 742

# Fusarium_sp-PT_000061-RA Number of predicted TMHs: 0

# Fusarium_sp-PT_000061-RA Exp number of AAs in TMHs: 0.92927

# Fusarium_sp-PT_000061-RA Exp number, first 60 AAs: 0

# Fusarium_sp-PT_000061-RA Total prob of N-in: 0.00222

Fusarium_sp-PT_000061-RA TMHMM2.0 outside 1 742

# Fusarium_sp-PT_000065-RA Length: 574

# Fusarium_sp-PT_000065-RA Number of predicted TMHs: 12

# Fusarium_sp-PT_000065-RA Exp number of AAs in TMHs: 261.23555

# Fusarium_sp-PT_000065-RA Exp number, first 60 AAs: 0.00012

# Fusarium_sp-PT_000065-RA Total prob of N-in: 0.64021

Fusarium_sp-PT_000065-RA TMHMM2.0 inside 1 93

Fusarium_sp-PT_000065-RA TMHMM2.0 TMhelix 94 116

Fusarium_sp-PT_000065-RA TMHMM2.0 outside 117 130

Fusarium_sp-PT_000065-RA TMHMM2.0 TMhelix 131 148

Fusarium_sp-PT_000065-RA TMHMM2.0 inside 149 154

Fusarium_sp-PT_000065-RA TMHMM2.0 TMhelix 155 177

Fusarium_sp-PT_000065-RA TMHMM2.0 outside 178 191

Fusarium_sp-PT_000065-RA TMHMM2.0 TMhelix 192 214

Fusarium_sp-PT_000065-RA TMHMM2.0 inside 215 220

Fusarium_sp-PT_000065-RA TMHMM2.0 TMhelix 221 240

Fusarium_sp-PT_000065-RA TMHMM2.0 outside 241 259

Fusarium_sp-PT_000065-RA TMHMM2.0 TMhelix 260 282

Fusarium_sp-PT_000065-RA TMHMM2.0 inside 283 302

Fusarium_sp-PT_000065-RA TMHMM2.0 TMhelix 303 325

Fusarium_sp-PT_000065-RA TMHMM2.0 outside 326 354

Fusarium_sp-PT_000065-RA TMHMM2.0 TMhelix 355 377

Fusarium_sp-PT_000065-RA TMHMM2.0 inside 378 397

Fusarium_sp-PT_000065-RA TMHMM2.0 TMhelix 398 415

Fusarium_sp-PT_000065-RA TMHMM2.0 outside 416 419

Fusarium_sp-PT_000065-RA TMHMM2.0 TMhelix 420 439

Fusarium_sp-PT_000065-RA TMHMM2.0 inside 440 473

Fusarium_sp-PT_000065-RA TMHMM2.0 TMhelix 474 496

Fusarium_sp-PT_000065-RA TMHMM2.0 outside 497 510

Fusarium_sp-PT_000065-RA TMHMM2.0 TMhelix 511 533

Fusarium_sp-PT_000065-RA TMHMM2.0 inside 534 574

# Fusarium_sp-PT_000067-RA Length: 333

# Fusarium_sp-PT_000067-RA Number of predicted TMHs: 0

# Fusarium_sp-PT_000067-RA Exp number of AAs in TMHs: 2.07381

# Fusarium_sp-PT_000067-RA Exp number, first 60 AAs: 0.01976

# Fusarium_sp-PT_000067-RA Total prob of N-in: 0.12027

Fusarium_sp-PT_000067-RA TMHMM2.0 outside 1 333

# Fusarium_sp-PT_000069-RA Length: 563

# Fusarium_sp-PT_000069-RA Number of predicted TMHs: 10

# Fusarium_sp-PT_000069-RA Exp number of AAs in TMHs: 239.86447

# Fusarium_sp-PT_000069-RA Exp number, first 60 AAs: 4.56257

# Fusarium_sp-PT_000069-RA Total prob of N-in: 0.98354

Fusarium_sp-PT_000069-RA TMHMM2.0 inside 1 54

Fusarium_sp-PT_000069-RA TMHMM2.0 TMhelix 55 77

Fusarium_sp-PT_000069-RA TMHMM2.0 outside 78 111

Fusarium_sp-PT_000069-RA TMHMM2.0 TMhelix 112 131

Fusarium_sp-PT_000069-RA TMHMM2.0 inside 132 137

Fusarium_sp-PT_000069-RA TMHMM2.0 TMhelix 138 156

Fusarium_sp-PT_000069-RA TMHMM2.0 outside 157 165

Fusarium_sp-PT_000069-RA TMHMM2.0 TMhelix 166 188

Fusarium_sp-PT_000069-RA TMHMM2.0 inside 189 194

Fusarium_sp-PT_000069-RA TMHMM2.0 TMhelix 195 217

Fusarium_sp-PT_000069-RA TMHMM2.0 outside 218 231

Fusarium_sp-PT_000069-RA TMHMM2.0 TMhelix 232 251

Fusarium_sp-PT_000069-RA TMHMM2.0 inside 252 336

Fusarium_sp-PT_000069-RA TMHMM2.0 TMhelix 337 359

Fusarium_sp-PT_000069-RA TMHMM2.0 outside 360 400

Fusarium_sp-PT_000069-RA TMHMM2.0 TMhelix 401 420

Fusarium_sp-PT_000069-RA TMHMM2.0 inside 421 432

Fusarium_sp-PT_000069-RA TMHMM2.0 TMhelix 433 455

Fusarium_sp-PT_000069-RA TMHMM2.0 outside 456 497

Fusarium_sp-PT_000069-RA TMHMM2.0 TMhelix 498 520

Fusarium_sp-PT_000069-RA TMHMM2.0 inside 521 563

# Fusarium_sp-PT_000070-RA Length: 310

# Fusarium_sp-PT_000070-RA Number of predicted TMHs: 0

# Fusarium_sp-PT_000070-RA Exp number of AAs in TMHs: 13.6048

# Fusarium_sp-PT_000070-RA Exp number, first 60 AAs: 10.12714

# Fusarium_sp-PT_000070-RA Total prob of N-in: 0.58740

# Fusarium_sp-PT_000070-RA POSSIBLE N-term signal sequence

Fusarium_sp-PT_000070-RA TMHMM2.0 outside 1 310

# Fusarium_sp-PT_000073-RA Length: 552

# Fusarium_sp-PT_000073-RA Number of predicted TMHs: 11

# Fusarium_sp-PT_000073-RA Exp number of AAs in TMHs: 242.79181

# Fusarium_sp-PT_000073-RA Exp number, first 60 AAs: 22.45359

# Fusarium_sp-PT_000073-RA Total prob of N-in: 0.95964

# Fusarium_sp-PT_000073-RA POSSIBLE N-term signal sequence

Fusarium_sp-PT_000073-RA TMHMM2.0 inside 1 20

Fusarium_sp-PT_000073-RA TMHMM2.0 TMhelix 21 43

Fusarium_sp-PT_000073-RA TMHMM2.0 outside 44 76

Fusarium_sp-PT_000073-RA TMHMM2.0 TMhelix 77 99

Fusarium_sp-PT_000073-RA TMHMM2.0 inside 100 103

Fusarium_sp-PT_000073-RA TMHMM2.0 TMhelix 104 121

Fusarium_sp-PT_000073-RA TMHMM2.0 outside 122 130

Fusarium_sp-PT_000073-RA TMHMM2.0 TMhelix 131 153

Fusarium_sp-PT_000073-RA TMHMM2.0 inside 154 164

Fusarium_sp-PT_000073-RA TMHMM2.0 TMhelix 165 187

Fusarium_sp-PT_000073-RA TMHMM2.0 outside 188 196

Fusarium_sp-PT_000073-RA TMHMM2.0 TMhelix 197 219

Fusarium_sp-PT_000073-RA TMHMM2.0 inside 220 289

Fusarium_sp-PT_000073-RA TMHMM2.0 TMhelix 290 312

Fusarium_sp-PT_000073-RA TMHMM2.0 outside 313 326

Fusarium_sp-PT_000073-RA TMHMM2.0 TMhelix 327 349

Fusarium_sp-PT_000073-RA TMHMM2.0 inside 350 357

Fusarium_sp-PT_000073-RA TMHMM2.0 TMhelix 358 380

Fusarium_sp-PT_000073-RA TMHMM2.0 outside 381 394

Fusarium_sp-PT_000073-RA TMHMM2.0 TMhelix 395 417

Fusarium_sp-PT_000073-RA TMHMM2.0 inside 418 467

Fusarium_sp-PT_000073-RA TMHMM2.0 TMhelix 468 490

Fusarium_sp-PT_000073-RA TMHMM2.0 outside 491 552

# Fusarium_sp-PT_000075-RA Length: 263

# Fusarium_sp-PT_000075-RA Number of predicted TMHs: 0

# Fusarium_sp-PT_000075-RA Exp number of AAs in TMHs: 0.46387

# Fusarium_sp-PT_000075-RA Exp number, first 60 AAs: 0.05487

# Fusarium_sp-PT_000075-RA Total prob of N-in: 0.04404

Fusarium_sp-PT_000075-RA TMHMM2.0 outside 1 263

# Fusarium_sp-PT_000060-RA Length: 457

# Fusarium_sp-PT_000060-RA Number of predicted TMHs: 0

# Fusarium_sp-PT_000060-RA Exp number of AAs in TMHs: 2.36001

# Fusarium_sp-PT_000060-RA Exp number, first 60 AAs: 0.00027

# Fusarium_sp-PT_000060-RA Total prob of N-in: 0.09505

Fusarium_sp-PT_000060-RA TMHMM2.0 outside 1 457

# Fusarium_sp-PT_000062-RA Length: 408

# Fusarium_sp-PT_000062-RA Number of predicted TMHs: 0

# Fusarium_sp-PT_000062-RA Exp number of AAs in TMHs: 7.12336999999998

# Fusarium_sp-PT_000062-RA Exp number, first 60 AAs: 7.06303

# Fusarium_sp-PT_000062-RA Total prob of N-in: 0.31154

Fusarium_sp-PT_000062-RA TMHMM2.0 outside 1 408

# Fusarium_sp-PT_000063-RA Length: 296

# Fusarium_sp-PT_000063-RA Number of predicted TMHs: 0

# Fusarium_sp-PT_000063-RA Exp number of AAs in TMHs: 0.15226

# Fusarium_sp-PT_000063-RA Exp number, first 60 AAs: 0.0054

# Fusarium_sp-PT_000063-RA Total prob of N-in: 0.03225

Fusarium_sp-PT_000063-RA TMHMM2.0 outside 1 296

# Fusarium_sp-PT_000064-RA Length: 212

# Fusarium_sp-PT_000064-RA Number of predicted TMHs: 0

# Fusarium_sp-PT_000064-RA Exp number of AAs in TMHs: 0.41294

# Fusarium_sp-PT_000064-RA Exp number, first 60 AAs: 0.01136

# Fusarium_sp-PT_000064-RA Total prob of N-in: 0.02662

Fusarium_sp-PT_000064-RA TMHMM2.0 outside 1 212

# Fusarium_sp-PT_000055-RA Length: 178

# Fusarium_sp-PT_000055-RA Number of predicted TMHs: 0

# Fusarium_sp-PT_000055-RA Exp number of AAs in TMHs: 1.54194

# Fusarium_sp-PT_000055-RA Exp number, first 60 AAs: 0.24

# Fusarium_sp-PT_000055-RA Total prob of N-in: 0.05601

Fusarium_sp-PT_000055-RA TMHMM2.0 outside 1 178

# Fusarium_sp-PT_000074-RA Length: 129

# Fusarium_sp-PT_000074-RA Number of predicted TMHs: 0

# Fusarium_sp-PT_000074-RA Exp number of AAs in TMHs: 0.03914

# Fusarium_sp-PT_000074-RA Exp number, first 60 AAs: 0

# Fusarium_sp-PT_000074-RA Total prob of N-in: 0.18959

Fusarium_sp-PT_000074-RA TMHMM2.0 outside 1 129

# Fusarium_sp-PT_000057-RA Length: 52

# Fusarium_sp-PT_000057-RA Number of predicted TMHs: 0

# Fusarium_sp-PT_000057-RA Exp number of AAs in TMHs: 0

# Fusarium_sp-PT_000057-RA Exp number, first 60 AAs: 0

# Fusarium_sp-PT_000057-RA Total prob of N-in: 0.32293

Fusarium_sp-PT_000057-RA TMHMM2.0 outside 1 52

# Fusarium_sp-PT_000076-RA Length: 251

# Fusarium_sp-PT_000076-RA Number of predicted TMHs: 6

# Fusarium_sp-PT_000076-RA Exp number of AAs in TMHs: 130.08032

# Fusarium_sp-PT_000076-RA Exp number, first 60 AAs: 7.35681

# Fusarium_sp-PT_000076-RA Total prob of N-in: 0.07088

Fusarium_sp-PT_000076-RA TMHMM2.0 outside 1 52

Fusarium_sp-PT_000076-RA TMHMM2.0 TMhelix 53 75

Fusarium_sp-PT_000076-RA TMHMM2.0 inside 76 81

Fusarium_sp-PT_000076-RA TMHMM2.0 TMhelix 82 104

Fusarium_sp-PT_000076-RA TMHMM2.0 outside 105 113

Fusarium_sp-PT_000076-RA TMHMM2.0 TMhelix 114 136

Fusarium_sp-PT_000076-RA TMHMM2.0 inside 137 148

Fusarium_sp-PT_000076-RA TMHMM2.0 TMhelix 149 168

Fusarium_sp-PT_000076-RA TMHMM2.0 outside 169 171

Fusarium_sp-PT_000076-RA TMHMM2.0 TMhelix 172 194

Fusarium_sp-PT_000076-RA TMHMM2.0 inside 195 202

Fusarium_sp-PT_000076-RA TMHMM2.0 TMhelix 203 225

Fusarium_sp-PT_000076-RA TMHMM2.0 outside 226 251

# Fusarium_sp-PT_000079-RA Length: 417

# Fusarium_sp-PT_000079-RA Number of predicted TMHs: 8

# Fusarium_sp-PT_000079-RA Exp number of AAs in TMHs: 169.10708

# Fusarium_sp-PT_000079-RA Exp number, first 60 AAs: 4.59782

# Fusarium_sp-PT_000079-RA Total prob of N-in: 0.74152

Fusarium_sp-PT_000079-RA TMHMM2.0 inside 1 84

Fusarium_sp-PT_000079-RA TMHMM2.0 TMhelix 85 102

Fusarium_sp-PT_000079-RA TMHMM2.0 outside 103 111

Fusarium_sp-PT_000079-RA TMHMM2.0 TMhelix 112 134

Fusarium_sp-PT_000079-RA TMHMM2.0 inside 135 145

Fusarium_sp-PT_000079-RA TMHMM2.0 TMhelix 146 165

Fusarium_sp-PT_000079-RA TMHMM2.0 outside 166 168

Fusarium_sp-PT_000079-RA TMHMM2.0 TMhelix 169 191

Fusarium_sp-PT_000079-RA TMHMM2.0 inside 192 247

Fusarium_sp-PT_000079-RA TMHMM2.0 TMhelix 248 266

Fusarium_sp-PT_000079-RA TMHMM2.0 outside 267 270

Fusarium_sp-PT_000079-RA TMHMM2.0 TMhelix 271 293

Fusarium_sp-PT_000079-RA TMHMM2.0 inside 294 333

Fusarium_sp-PT_000079-RA TMHMM2.0 TMhelix 334 351

Fusarium_sp-PT_000079-RA TMHMM2.0 outside 352 365

Fusarium_sp-PT_000079-RA TMHMM2.0 TMhelix 366 388

Fusarium_sp-PT_000079-RA TMHMM2.0 inside 389 417

# Fusarium_sp-PT_000080-RA Length: 370

# Fusarium_sp-PT_000080-RA Number of predicted TMHs: 0

# Fusarium_sp-PT_000080-RA Exp number of AAs in TMHs: 2.25114

# Fusarium_sp-PT_000080-RA Exp number, first 60 AAs: 0

# Fusarium_sp-PT_000080-RA Total prob of N-in: 0.01593

Fusarium_sp-PT_000080-RA TMHMM2.0 outside 1 370

# Fusarium_sp-PT_000081-RA Length: 514

# Fusarium_sp-PT_000081-RA Number of predicted TMHs: 11

# Fusarium_sp-PT_000081-RA Exp number of AAs in TMHs: 242.49817

# Fusarium_sp-PT_000081-RA Exp number, first 60 AAs: 13.45509

# Fusarium_sp-PT_000081-RA Total prob of N-in: 0.37879

# Fusarium_sp-PT_000081-RA POSSIBLE N-term signal sequence

Fusarium_sp-PT_000081-RA TMHMM2.0 outside 1 46

Fusarium_sp-PT_000081-RA TMHMM2.0 TMhelix 47 69

Fusarium_sp-PT_000081-RA TMHMM2.0 inside 70 75

Fusarium_sp-PT_000081-RA TMHMM2.0 TMhelix 76 98

Fusarium_sp-PT_000081-RA TMHMM2.0 outside 99 123

Fusarium_sp-PT_000081-RA TMHMM2.0 TMhelix 124 146

Fusarium_sp-PT_000081-RA TMHMM2.0 inside 147 152

Fusarium_sp-PT_000081-RA TMHMM2.0 TMhelix 153 175

Fusarium_sp-PT_000081-RA TMHMM2.0 outside 176 178

Fusarium_sp-PT_000081-RA TMHMM2.0 TMhelix 179 196

Fusarium_sp-PT_000081-RA TMHMM2.0 inside 197 266

Fusarium_sp-PT_000081-RA TMHMM2.0 TMhelix 267 289

Fusarium_sp-PT_000081-RA TMHMM2.0 outside 290 308

Fusarium_sp-PT_000081-RA TMHMM2.0 TMhelix 309 331

Fusarium_sp-PT_000081-RA TMHMM2.0 inside 332 364

Fusarium_sp-PT_000081-RA TMHMM2.0 TMhelix 365 384

Fusarium_sp-PT_000081-RA TMHMM2.0 outside 385 393

Fusarium_sp-PT_000081-RA TMHMM2.0 TMhelix 394 416

Fusarium_sp-PT_000081-RA TMHMM2.0 inside 417 435

Fusarium_sp-PT_000081-RA TMHMM2.0 TMhelix 436 458

Fusarium_sp-PT_000081-RA TMHMM2.0 outside 459 472

Fusarium_sp-PT_000081-RA TMHMM2.0 TMhelix 473 490

Fusarium_sp-PT_000081-RA TMHMM2.0 inside 491 514

# Fusarium_sp-PT_000082-RA Length: 337

# Fusarium_sp-PT_000082-RA Number of predicted TMHs: 0

# Fusarium_sp-PT_000082-RA Exp number of AAs in TMHs: 0.00761999999999999

# Fusarium_sp-PT_000082-RA Exp number, first 60 AAs: 0.00075

# Fusarium_sp-PT_000082-RA Total prob of N-in: 0.02842

Fusarium_sp-PT_000082-RA TMHMM2.0 outside 1 337

# Fusarium_sp-PT_000084-RA Length: 162

# Fusarium_sp-PT_000084-RA Number of predicted TMHs: 0

# Fusarium_sp-PT_000084-RA Exp number of AAs in TMHs: 0.01396

# Fusarium_sp-PT_000084-RA Exp number, first 60 AAs: 0.0131

# Fusarium_sp-PT_000084-RA Total prob of N-in: 0.07880

Fusarium_sp-PT_000084-RA TMHMM2.0 outside 1 162

# Fusarium_sp-PT_000086-RA Length: 595

# Fusarium_sp-PT_000086-RA Number of predicted TMHs: 0

# Fusarium_sp-PT_000086-RA Exp number of AAs in TMHs: 0.96873

# Fusarium_sp-PT_000086-RA Exp number, first 60 AAs: 0.0002

# Fusarium_sp-PT_000086-RA Total prob of N-in: 0.00028

Fusarium_sp-PT_000086-RA TMHMM2.0 outside 1 595

# Fusarium_sp-PT_000087-RA Length: 163

# Fusarium_sp-PT_000087-RA Number of predicted TMHs: 0

# Fusarium_sp-PT_000087-RA Exp number of AAs in TMHs: 0.0145

# Fusarium_sp-PT_000087-RA Exp number, first 60 AAs: 0

# Fusarium_sp-PT_000087-RA Total prob of N-in: 0.11335

Fusarium_sp-PT_000087-RA TMHMM2.0 outside 1 163

# Fusarium_sp-PT_000088-RA Length: 471

# Fusarium_sp-PT_000088-RA Number of predicted TMHs: 0

# Fusarium_sp-PT_000088-RA Exp number of AAs in TMHs: 0.49127

# Fusarium_sp-PT_000088-RA Exp number, first 60 AAs: 0.00313

# Fusarium_sp-PT_000088-RA Total prob of N-in: 0.00358

Fusarium_sp-PT_000088-RA TMHMM2.0 outside 1 471

# Fusarium_sp-PT_000093-RA Length: 629

# Fusarium_sp-PT_000093-RA Number of predicted TMHs: 0

# Fusarium_sp-PT_000093-RA Exp number of AAs in TMHs: 0.582519999999999

# Fusarium_sp-PT_000093-RA Exp number, first 60 AAs: 0

# Fusarium_sp-PT_000093-RA Total prob of N-in: 0.02002

Fusarium_sp-PT_000093-RA TMHMM2.0 outside 1 629

# Fusarium_sp-PT_000097-RA Length: 704

# Fusarium_sp-PT_000097-RA Number of predicted TMHs: 0

# Fusarium_sp-PT_000097-RA Exp number of AAs in TMHs: 0.277850000000001

# Fusarium_sp-PT_000097-RA Exp number, first 60 AAs: 0.09162

# Fusarium_sp-PT_000097-RA Total prob of N-in: 0.00928

Fusarium_sp-PT_000097-RA TMHMM2.0 outside 1 704

# Fusarium_sp-PT_000077-RA Length: 569

# Fusarium_sp-PT_000077-RA Number of predicted TMHs: 0

# Fusarium_sp-PT_000077-RA Exp number of AAs in TMHs: 4.17716

# Fusarium_sp-PT_000077-RA Exp number, first 60 AAs: 3.65629

# Fusarium_sp-PT_000077-RA Total prob of N-in: 0.15567

Fusarium_sp-PT_000077-RA TMHMM2.0 outside 1 569

# Fusarium_sp-PT_000083-RA Length: 104

# Fusarium_sp-PT_000083-RA Number of predicted TMHs: 1

# Fusarium_sp-PT_000083-RA Exp number of AAs in TMHs: 20.74377

# Fusarium_sp-PT_000083-RA Exp number, first 60 AAs: 20.74377

# Fusarium_sp-PT_000083-RA Total prob of N-in: 0.94183

# Fusarium_sp-PT_000083-RA POSSIBLE N-term signal sequence

Fusarium_sp-PT_000083-RA TMHMM2.0 inside 1 4

Fusarium_sp-PT_000083-RA TMHMM2.0 TMhelix 5 27

Fusarium_sp-PT_000083-RA TMHMM2.0 outside 28 104

# Fusarium_sp-PT_000085-RA Length: 228

# Fusarium_sp-PT_000085-RA Number of predicted TMHs: 0

# Fusarium_sp-PT_000085-RA Exp number of AAs in TMHs: 0.25588

# Fusarium_sp-PT_000085-RA Exp number, first 60 AAs: 0.23778

# Fusarium_sp-PT_000085-RA Total prob of N-in: 0.04843

Fusarium_sp-PT_000085-RA TMHMM2.0 outside 1 228

# Fusarium_sp-PT_000089-RA Length: 866

# Fusarium_sp-PT_000089-RA Number of predicted TMHs: 0

# Fusarium_sp-PT_000089-RA Exp number of AAs in TMHs: 1.01233

# Fusarium_sp-PT_000089-RA Exp number, first 60 AAs: 0

# Fusarium_sp-PT_000089-RA Total prob of N-in: 0.01533

Fusarium_sp-PT_000089-RA TMHMM2.0 outside 1 866

# Fusarium_sp-PT_000090-RA Length: 471

# Fusarium_sp-PT_000090-RA Number of predicted TMHs: 0

# Fusarium_sp-PT_000090-RA Exp number of AAs in TMHs: 0.00124

# Fusarium_sp-PT_000090-RA Exp number, first 60 AAs: 0.00052

# Fusarium_sp-PT_000090-RA Total prob of N-in: 0.00121

Fusarium_sp-PT_000090-RA TMHMM2.0 outside 1 471

# Fusarium_sp-PT_000091-RA Length: 92

# Fusarium_sp-PT_000091-RA Number of predicted TMHs: 0

# Fusarium_sp-PT_000091-RA Exp number of AAs in TMHs: 0.51818

# Fusarium_sp-PT_000091-RA Exp number, first 60 AAs: 0.51818

# Fusarium_sp-PT_000091-RA Total prob of N-in: 0.15985

Fusarium_sp-PT_000091-RA TMHMM2.0 outside 1 92

# Fusarium_sp-PT_000095-RA Length: 327

# Fusarium_sp-PT_000095-RA Number of predicted TMHs: 0

# Fusarium_sp-PT_000095-RA Exp number of AAs in TMHs: 0.09537

# Fusarium_sp-PT_000095-RA Exp number, first 60 AAs: 0.00916

# Fusarium_sp-PT_000095-RA Total prob of N-in: 0.01073

Fusarium_sp-PT_000095-RA TMHMM2.0 outside 1 327

# Fusarium_sp-PT_000078-RA Length: 508

# Fusarium_sp-PT_000078-RA Number of predicted TMHs: 0

# Fusarium_sp-PT_000078-RA Exp number of AAs in TMHs: 3.0881

# Fusarium_sp-PT_000078-RA Exp number, first 60 AAs: 0.00458

# Fusarium_sp-PT_000078-RA Total prob of N-in: 0.15379

Fusarium_sp-PT_000078-RA TMHMM2.0 outside 1 508

# Fusarium_sp-PT_000092-RA Length: 249

# Fusarium_sp-PT_000092-RA Number of predicted TMHs: 0

# Fusarium_sp-PT_000092-RA Exp number of AAs in TMHs: 48.46166

# Fusarium_sp-PT_000092-RA Exp number, first 60 AAs: 20.88275

# Fusarium_sp-PT_000092-RA Total prob of N-in: 0.70806

# Fusarium_sp-PT_000092-RA POSSIBLE N-term signal sequence

Fusarium_sp-PT_000092-RA TMHMM2.0 outside 1 249

# Fusarium_sp-PT_000096-RA Length: 231

# Fusarium_sp-PT_000096-RA Number of predicted TMHs: 1

# Fusarium_sp-PT_000096-RA Exp number of AAs in TMHs: 18.27149

# Fusarium_sp-PT_000096-RA Exp number, first 60 AAs: 17.58298

# Fusarium_sp-PT_000096-RA Total prob of N-in: 0.78947

# Fusarium_sp-PT_000096-RA POSSIBLE N-term signal sequence

Fusarium_sp-PT_000096-RA TMHMM2.0 inside 1 11

Fusarium_sp-PT_000096-RA TMHMM2.0 TMhelix 12 34

Fusarium_sp-PT_000096-RA TMHMM2.0 outside 35 231

# Fusarium_sp-PT_000094-RA Length: 223

# Fusarium_sp-PT_000094-RA Number of predicted TMHs: 0

# Fusarium_sp-PT_000094-RA Exp number of AAs in TMHs: 7.59343999999999

# Fusarium_sp-PT_000094-RA Exp number, first 60 AAs: 0.13254

# Fusarium_sp-PT_000094-RA Total prob of N-in: 0.34499

Fusarium_sp-PT_000094-RA TMHMM2.0 outside 1 223

# Fusarium_sp-PT_000100-RA Length: 352

# Fusarium_sp-PT_000100-RA Number of predicted TMHs: 0

# Fusarium_sp-PT_000100-RA Exp number of AAs in TMHs: 6.48165

# Fusarium_sp-PT_000100-RA Exp number, first 60 AAs: 6.45479

# Fusarium_sp-PT_000100-RA Total prob of N-in: 0.29669

Fusarium_sp-PT_000100-RA TMHMM2.0 outside 1 352

# Fusarium_sp-PT_000103-RA Length: 486

# Fusarium_sp-PT_000103-RA Number of predicted TMHs: 0

# Fusarium_sp-PT_000103-RA Exp number of AAs in TMHs: 0.08689

# Fusarium_sp-PT_000103-RA Exp number, first 60 AAs: 0.00011

# Fusarium_sp-PT_000103-RA Total prob of N-in: 0.01243

Fusarium_sp-PT_000103-RA TMHMM2.0 outside 1 486

# Fusarium_sp-PT_000108-RA Length: 607

# Fusarium_sp-PT_000108-RA Number of predicted TMHs: 0

# Fusarium_sp-PT_000108-RA Exp number of AAs in TMHs: 0.00417

# Fusarium_sp-PT_000108-RA Exp number, first 60 AAs: 0

# Fusarium_sp-PT_000108-RA Total prob of N-in: 0.00045

Fusarium_sp-PT_000108-RA TMHMM2.0 outside 1 607

# Fusarium_sp-PT_000111-RA Length: 180

# Fusarium_sp-PT_000111-RA Number of predicted TMHs: 1

# Fusarium_sp-PT_000111-RA Exp number of AAs in TMHs: 34.92165

# Fusarium_sp-PT_000111-RA Exp number, first 60 AAs: 15.35284

# Fusarium_sp-PT_000111-RA Total prob of N-in: 0.35688

# Fusarium_sp-PT_000111-RA POSSIBLE N-term signal sequence

Fusarium_sp-PT_000111-RA TMHMM2.0 outside 1 42

Fusarium_sp-PT_000111-RA TMHMM2.0 TMhelix 43 65

Fusarium_sp-PT_000111-RA TMHMM2.0 inside 66 180

# Fusarium_sp-PT_000115-RA Length: 342

# Fusarium_sp-PT_000115-RA Number of predicted TMHs: 0

# Fusarium_sp-PT_000115-RA Exp number of AAs in TMHs: 0.00354

# Fusarium_sp-PT_000115-RA Exp number, first 60 AAs: 0.00208

# Fusarium_sp-PT_000115-RA Total prob of N-in: 0.00698

Fusarium_sp-PT_000115-RA TMHMM2.0 outside 1 342

# Fusarium_sp-PT_000117-RA Length: 494

# Fusarium_sp-PT_000117-RA Number of predicted TMHs: 12

# Fusarium_sp-PT_000117-RA Exp number of AAs in TMHs: 252.11458

# Fusarium_sp-PT_000117-RA Exp number, first 60 AAs: 30.06557

# Fusarium_sp-PT_000117-RA Total prob of N-in: 0.84825

# Fusarium_sp-PT_000117-RA POSSIBLE N-term signal sequence

Fusarium_sp-PT_000117-RA TMHMM2.0 inside 1 12

Fusarium_sp-PT_000117-RA TMHMM2.0 TMhelix 13 35

Fusarium_sp-PT_000117-RA TMHMM2.0 outside 36 49

Fusarium_sp-PT_000117-RA TMHMM2.0 TMhelix 50 72

Fusarium_sp-PT_000117-RA TMHMM2.0 inside 73 84

Fusarium_sp-PT_000117-RA TMHMM2.0 TMhelix 85 107

Fusarium_sp-PT_000117-RA TMHMM2.0 outside 108 111

Fusarium_sp-PT_000117-RA TMHMM2.0 TMhelix 112 129

Fusarium_sp-PT_000117-RA TMHMM2.0 inside 130 141

Fusarium_sp-PT_000117-RA TMHMM2.0 TMhelix 142 161

Fusarium_sp-PT_000117-RA TMHMM2.0 outside 162 170

Fusarium_sp-PT_000117-RA TMHMM2.0 TMhelix 171 193

Fusarium_sp-PT_000117-RA TMHMM2.0 inside 194 260

Fusarium_sp-PT_000117-RA TMHMM2.0 TMhelix 261 283

Fusarium_sp-PT_000117-RA TMHMM2.0 outside 284 297

Fusarium_sp-PT_000117-RA TMHMM2.0 TMhelix 298 320

Fusarium_sp-PT_000117-RA TMHMM2.0 inside 321 326

Fusarium_sp-PT_000117-RA TMHMM2.0 TMhelix 327 349

Fusarium_sp-PT_000117-RA TMHMM2.0 outside 350 358

Fusarium_sp-PT_000117-RA TMHMM2.0 TMhelix 359 381

Fusarium_sp-PT_000117-RA TMHMM2.0 inside 382 401

Fusarium_sp-PT_000117-RA TMHMM2.0 TMhelix 402 424

Fusarium_sp-PT_000117-RA TMHMM2.0 outside 425 429

Fusarium_sp-PT_000117-RA TMHMM2.0 TMhelix 430 449

Fusarium_sp-PT_000117-RA TMHMM2.0 inside 450 494

# Fusarium_sp-PT_000121-RA Length: 624

# Fusarium_sp-PT_000121-RA Number of predicted TMHs: 0

# Fusarium_sp-PT_000121-RA Exp number of AAs in TMHs: 1.03738

# Fusarium_sp-PT_000121-RA Exp number, first 60 AAs: 0.00472

# Fusarium_sp-PT_000121-RA Total prob of N-in: 0.04987

Fusarium_sp-PT_000121-RA TMHMM2.0 outside 1 624

# Fusarium_sp-PT_000122-RA Length: 447

# Fusarium_sp-PT_000122-RA Number of predicted TMHs: 0

# Fusarium_sp-PT_000122-RA Exp number of AAs in TMHs: 0.17019

# Fusarium_sp-PT_000122-RA Exp number, first 60 AAs: 0.0262

# Fusarium_sp-PT_000122-RA Total prob of N-in: 0.00450

Fusarium_sp-PT_000122-RA TMHMM2.0 outside 1 447

# Fusarium_sp-PT_000125-RA Length: 674

# Fusarium_sp-PT_000125-RA Number of predicted TMHs: 4

# Fusarium_sp-PT_000125-RA Exp number of AAs in TMHs: 82.68866

# Fusarium_sp-PT_000125-RA Exp number, first 60 AAs: 0

# Fusarium_sp-PT_000125-RA Total prob of N-in: 0.55237

Fusarium_sp-PT_000125-RA TMHMM2.0 inside 1 356

Fusarium_sp-PT_000125-RA TMHMM2.0 TMhelix 357 379

Fusarium_sp-PT_000125-RA TMHMM2.0 outside 380 388

Fusarium_sp-PT_000125-RA TMHMM2.0 TMhelix 389 411

Fusarium_sp-PT_000125-RA TMHMM2.0 inside 412 549

Fusarium_sp-PT_000125-RA TMHMM2.0 TMhelix 550 572

Fusarium_sp-PT_000125-RA TMHMM2.0 outside 573 581

Fusarium_sp-PT_000125-RA TMHMM2.0 TMhelix 582 604

Fusarium_sp-PT_000125-RA TMHMM2.0 inside 605 674

# Fusarium_sp-PT_000128-RA Length: 537

# Fusarium_sp-PT_000128-RA Number of predicted TMHs: 4

# Fusarium_sp-PT_000128-RA Exp number of AAs in TMHs: 85.68052

# Fusarium_sp-PT_000128-RA Exp number, first 60 AAs: 0

# Fusarium_sp-PT_000128-RA Total prob of N-in: 0.89211

Fusarium_sp-PT_000128-RA TMHMM2.0 inside 1 122

Fusarium_sp-PT_000128-RA TMHMM2.0 TMhelix 123 145

Fusarium_sp-PT_000128-RA TMHMM2.0 outside 146 177

Fusarium_sp-PT_000128-RA TMHMM2.0 TMhelix 178 200

Fusarium_sp-PT_000128-RA TMHMM2.0 inside 201 219

Fusarium_sp-PT_000128-RA TMHMM2.0 TMhelix 220 242

Fusarium_sp-PT_000128-RA TMHMM2.0 outside 243 261

Fusarium_sp-PT_000128-RA TMHMM2.0 TMhelix 262 284

Fusarium_sp-PT_000128-RA TMHMM2.0 inside 285 537

# Fusarium_sp-PT_000130-RA Length: 790

# Fusarium_sp-PT_000130-RA Number of predicted TMHs: 0

# Fusarium_sp-PT_000130-RA Exp number of AAs in TMHs: 0.000870000000000001

# Fusarium_sp-PT_000130-RA Exp number, first 60 AAs: 0

# Fusarium_sp-PT_000130-RA Total prob of N-in: 0.00005

Fusarium_sp-PT_000130-RA TMHMM2.0 outside 1 790

# Fusarium_sp-PT_000099-RA Length: 860

# Fusarium_sp-PT_000099-RA Number of predicted TMHs: 0

# Fusarium_sp-PT_000099-RA Exp number of AAs in TMHs: 5.23784999999999

# Fusarium_sp-PT_000099-RA Exp number, first 60 AAs: 5.2346

# Fusarium_sp-PT_000099-RA Total prob of N-in: 0.25571

Fusarium_sp-PT_000099-RA TMHMM2.0 outside 1 860

# Fusarium_sp-PT_000101-RA Length: 160

# Fusarium_sp-PT_000101-RA Number of predicted TMHs: 0

# Fusarium_sp-PT_000101-RA Exp number of AAs in TMHs: 0.00663

# Fusarium_sp-PT_000101-RA Exp number, first 60 AAs: 0

# Fusarium_sp-PT_000101-RA Total prob of N-in: 0.36045

Fusarium_sp-PT_000101-RA TMHMM2.0 outside 1 160

# Fusarium_sp-PT_000102-RA Length: 367

# Fusarium_sp-PT_000102-RA Number of predicted TMHs: 0

# Fusarium_sp-PT_000102-RA Exp number of AAs in TMHs: 0.788590000000001

# Fusarium_sp-PT_000102-RA Exp number, first 60 AAs: 0.78493

# Fusarium_sp-PT_000102-RA Total prob of N-in: 0.03880

Fusarium_sp-PT_000102-RA TMHMM2.0 outside 1 367

# Fusarium_sp-PT_000104-RA Length: 498

# Fusarium_sp-PT_000104-RA Number of predicted TMHs: 12

# Fusarium_sp-PT_000104-RA Exp number of AAs in TMHs: 252.57704

# Fusarium_sp-PT_000104-RA Exp number, first 60 AAs: 22.78434

# Fusarium_sp-PT_000104-RA Total prob of N-in: 0.96656

# Fusarium_sp-PT_000104-RA POSSIBLE N-term signal sequence

Fusarium_sp-PT_000104-RA TMHMM2.0 inside 1 33

Fusarium_sp-PT_000104-RA TMHMM2.0 TMhelix 34 56

Fusarium_sp-PT_000104-RA TMHMM2.0 outside 57 70

Fusarium_sp-PT_000104-RA TMHMM2.0 TMhelix 71 93

Fusarium_sp-PT_000104-RA TMHMM2.0 inside 94 99

Fusarium_sp-PT_000104-RA TMHMM2.0 TMhelix 100 119

Fusarium_sp-PT_000104-RA TMHMM2.0 outside 120 124

Fusarium_sp-PT_000104-RA TMHMM2.0 TMhelix 125 147

Fusarium_sp-PT_000104-RA TMHMM2.0 inside 148 158

Fusarium_sp-PT_000104-RA TMHMM2.0 TMhelix 159 181

Fusarium_sp-PT_000104-RA TMHMM2.0 outside 182 190

Fusarium_sp-PT_000104-RA TMHMM2.0 TMhelix 191 213

Fusarium_sp-PT_000104-RA TMHMM2.0 inside 214 271

Fusarium_sp-PT_000104-RA TMHMM2.0 TMhelix 272 294

Fusarium_sp-PT_000104-RA TMHMM2.0 outside 295 308

Fusarium_sp-PT_000104-RA TMHMM2.0 TMhelix 309 331

Fusarium_sp-PT_000104-RA TMHMM2.0 inside 332 369

Fusarium_sp-PT_000104-RA TMHMM2.0 TMhelix 370 392

Fusarium_sp-PT_000104-RA TMHMM2.0 outside 393 396

Fusarium_sp-PT_000104-RA TMHMM2.0 TMhelix 397 419

Fusarium_sp-PT_000104-RA TMHMM2.0 inside 420 430

Fusarium_sp-PT_000104-RA TMHMM2.0 TMhelix 431 453

Fusarium_sp-PT_000104-RA TMHMM2.0 outside 454 457

Fusarium_sp-PT_000104-RA TMHMM2.0 TMhelix 458 480

Fusarium_sp-PT_000104-RA TMHMM2.0 inside 481 498

# Fusarium_sp-PT_000105-RA Length: 382

# Fusarium_sp-PT_000105-RA Number of predicted TMHs: 6

# Fusarium_sp-PT_000105-RA Exp number of AAs in TMHs: 132.42614

# Fusarium_sp-PT_000105-RA Exp number, first 60 AAs: 22.55678

# Fusarium_sp-PT_000105-RA Total prob of N-in: 0.93086

# Fusarium_sp-PT_000105-RA POSSIBLE N-term signal sequence

Fusarium_sp-PT_000105-RA TMHMM2.0 inside 1 6

Fusarium_sp-PT_000105-RA TMHMM2.0 TMhelix 7 29

Fusarium_sp-PT_000105-RA TMHMM2.0 outside 30 84

Fusarium_sp-PT_000105-RA TMHMM2.0 TMhelix 85 107

Fusarium_sp-PT_000105-RA TMHMM2.0 inside 108 118

Fusarium_sp-PT_000105-RA TMHMM2.0 TMhelix 119 141

Fusarium_sp-PT_000105-RA TMHMM2.0 outside 142 167

Fusarium_sp-PT_000105-RA TMHMM2.0 TMhelix 168 190

Fusarium_sp-PT_000105-RA TMHMM2.0 inside 191 202

Fusarium_sp-PT_000105-RA TMHMM2.0 TMhelix 203 225

Fusarium_sp-PT_000105-RA TMHMM2.0 outside 226 234

Fusarium_sp-PT_000105-RA TMHMM2.0 TMhelix 235 257

Fusarium_sp-PT_000105-RA TMHMM2.0 inside 258 382

# Fusarium_sp-PT_000106-RA Length: 422

# Fusarium_sp-PT_000106-RA Number of predicted TMHs: 7

# Fusarium_sp-PT_000106-RA Exp number of AAs in TMHs: 146.54514

# Fusarium_sp-PT_000106-RA Exp number, first 60 AAs: 31.01413

# Fusarium_sp-PT_000106-RA Total prob of N-in: 0.24506

# Fusarium_sp-PT_000106-RA POSSIBLE N-term signal sequence

Fusarium_sp-PT_000106-RA TMHMM2.0 outside 1 19

Fusarium_sp-PT_000106-RA TMHMM2.0 TMhelix 20 39

Fusarium_sp-PT_000106-RA TMHMM2.0 inside 40 45

Fusarium_sp-PT_000106-RA TMHMM2.0 TMhelix 46 68

Fusarium_sp-PT_000106-RA TMHMM2.0 outside 69 82

Fusarium_sp-PT_000106-RA TMHMM2.0 TMhelix 83 105

Fusarium_sp-PT_000106-RA TMHMM2.0 inside 106 116

Fusarium_sp-PT_000106-RA TMHMM2.0 TMhelix 117 139

Fusarium_sp-PT_000106-RA TMHMM2.0 outside 140 165

Fusarium_sp-PT_000106-RA TMHMM2.0 TMhelix 166 188

Fusarium_sp-PT_000106-RA TMHMM2.0 inside 189 290

Fusarium_sp-PT_000106-RA TMHMM2.0 TMhelix 291 313

Fusarium_sp-PT_000106-RA TMHMM2.0 outside 314 327

Fusarium_sp-PT_000106-RA TMHMM2.0 TMhelix 328 350

Fusarium_sp-PT_000106-RA TMHMM2.0 inside 351 422

# Fusarium_sp-PT_000107-RA Length: 543

# Fusarium_sp-PT_000107-RA Number of predicted TMHs: 10

# Fusarium_sp-PT_000107-RA Exp number of AAs in TMHs: 203.30195

# Fusarium_sp-PT_000107-RA Exp number, first 60 AAs: 3.69803

# Fusarium_sp-PT_000107-RA Total prob of N-in: 0.80257

Fusarium_sp-PT_000107-RA TMHMM2.0 inside 1 134

Fusarium_sp-PT_000107-RA TMHMM2.0 TMhelix 135 154

Fusarium_sp-PT_000107-RA TMHMM2.0 outside 155 157

Fusarium_sp-PT_000107-RA TMHMM2.0 TMhelix 158 180

Fusarium_sp-PT_000107-RA TMHMM2.0 inside 181 186

Fusarium_sp-PT_000107-RA TMHMM2.0 TMhelix 187 209

Fusarium_sp-PT_000107-RA TMHMM2.0 outside 210 228

Fusarium_sp-PT_000107-RA TMHMM2.0 TMhelix 229 251

Fusarium_sp-PT_000107-RA TMHMM2.0 inside 252 313

Fusarium_sp-PT_000107-RA TMHMM2.0 TMhelix 314 336

Fusarium_sp-PT_000107-RA TMHMM2.0 outside 337 350

Fusarium_sp-PT_000107-RA TMHMM2.0 TMhelix 351 368

Fusarium_sp-PT_000107-RA TMHMM2.0 inside 369 374

Fusarium_sp-PT_000107-RA TMHMM2.0 TMhelix 375 397

Fusarium_sp-PT_000107-RA TMHMM2.0 outside 398 406

Fusarium_sp-PT_000107-RA TMHMM2.0 TMhelix 407 429

Fusarium_sp-PT_000107-RA TMHMM2.0 inside 430 441

Fusarium_sp-PT_000107-RA TMHMM2.0 TMhelix 442 460

Fusarium_sp-PT_000107-RA TMHMM2.0 outside 461 474

Fusarium_sp-PT_000107-RA TMHMM2.0 TMhelix 475 492

Fusarium_sp-PT_000107-RA TMHMM2.0 inside 493 543

# Fusarium_sp-PT_000109-RA Length: 569

# Fusarium_sp-PT_000109-RA Number of predicted TMHs: 0

# Fusarium_sp-PT_000109-RA Exp number of AAs in TMHs: 0.29351

# Fusarium_sp-PT_000109-RA Exp number, first 60 AAs: 0.00545

# Fusarium_sp-PT_000109-RA Total prob of N-in: 0.01528

Fusarium_sp-PT_000109-RA TMHMM2.0 outside 1 569

# Fusarium_sp-PT_000110-RA Length: 312

# Fusarium_sp-PT_000110-RA Number of predicted TMHs: 0

# Fusarium_sp-PT_000110-RA Exp number of AAs in TMHs: 0.12666

# Fusarium_sp-PT_000110-RA Exp number, first 60 AAs: 0.00332

# Fusarium_sp-PT_000110-RA Total prob of N-in: 0.01521

Fusarium_sp-PT_000110-RA TMHMM2.0 outside 1 312

# Fusarium_sp-PT_000112-RA Length: 1337

# Fusarium_sp-PT_000112-RA Number of predicted TMHs: 0

# Fusarium_sp-PT_000112-RA Exp number of AAs in TMHs: 1.11410000000001

# Fusarium_sp-PT_000112-RA Exp number, first 60 AAs: 0.00146

# Fusarium_sp-PT_000112-RA Total prob of N-in: 0.05066

Fusarium_sp-PT_000112-RA TMHMM2.0 outside 1 1337

# Fusarium_sp-PT_000113-RA Length: 108

# Fusarium_sp-PT_000113-RA Number of predicted TMHs: 0

# Fusarium_sp-PT_000113-RA Exp number of AAs in TMHs: 0.00033

# Fusarium_sp-PT_000113-RA Exp number, first 60 AAs: 0.00033

# Fusarium_sp-PT_000113-RA Total prob of N-in: 0.27757

Fusarium_sp-PT_000113-RA TMHMM2.0 outside 1 108

# Fusarium_sp-PT_000114-RA Length: 689

# Fusarium_sp-PT_000114-RA Number of predicted TMHs: 3

# Fusarium_sp-PT_000114-RA Exp number of AAs in TMHs: 74.3051700000001

# Fusarium_sp-PT_000114-RA Exp number, first 60 AAs: 20.19208

# Fusarium_sp-PT_000114-RA Total prob of N-in: 0.29793

# Fusarium_sp-PT_000114-RA POSSIBLE N-term signal sequence

Fusarium_sp-PT_000114-RA TMHMM2.0 outside 1 33

Fusarium_sp-PT_000114-RA TMHMM2.0 TMhelix 34 53

Fusarium_sp-PT_000114-RA TMHMM2.0 inside 54 65

Fusarium_sp-PT_000114-RA TMHMM2.0 TMhelix 66 88

Fusarium_sp-PT_000114-RA TMHMM2.0 outside 89 598

Fusarium_sp-PT_000114-RA TMHMM2.0 TMhelix 599 621

Fusarium_sp-PT_000114-RA TMHMM2.0 inside 622 689

# Fusarium_sp-PT_000116-RA Length: 523

# Fusarium_sp-PT_000116-RA Number of predicted TMHs: 1

# Fusarium_sp-PT_000116-RA Exp number of AAs in TMHs: 21.28793

# Fusarium_sp-PT_000116-RA Exp number, first 60 AAs: 19.1552

# Fusarium_sp-PT_000116-RA Total prob of N-in: 0.89558

# Fusarium_sp-PT_000116-RA POSSIBLE N-term signal sequence

Fusarium_sp-PT_000116-RA TMHMM2.0 inside 1 4

Fusarium_sp-PT_000116-RA TMHMM2.0 TMhelix 5 22

Fusarium_sp-PT_000116-RA TMHMM2.0 outside 23 523

# Fusarium_sp-PT_000118-RA Length: 237

# Fusarium_sp-PT_000118-RA Number of predicted TMHs: 0

# Fusarium_sp-PT_000118-RA Exp number of AAs in TMHs: 0.3708

# Fusarium_sp-PT_000118-RA Exp number, first 60 AAs: 0.00041

# Fusarium_sp-PT_000118-RA Total prob of N-in: 0.05496

Fusarium_sp-PT_000118-RA TMHMM2.0 outside 1 237

# Fusarium_sp-PT_000120-RA Length: 392

# Fusarium_sp-PT_000120-RA Number of predicted TMHs: 0

# Fusarium_sp-PT_000120-RA Exp number of AAs in TMHs: 0.00603999999999998

# Fusarium_sp-PT_000120-RA Exp number, first 60 AAs: 0.00513

# Fusarium_sp-PT_000120-RA Total prob of N-in: 0.00876

Fusarium_sp-PT_000120-RA TMHMM2.0 outside 1 392

# Fusarium_sp-PT_000126-RA Length: 653

# Fusarium_sp-PT_000126-RA Number of predicted TMHs: 12

# Fusarium_sp-PT_000126-RA Exp number of AAs in TMHs: 257.58594

# Fusarium_sp-PT_000126-RA Exp number, first 60 AAs: 0

# Fusarium_sp-PT_000126-RA Total prob of N-in: 0.83288

Fusarium_sp-PT_000126-RA TMHMM2.0 inside 1 122

Fusarium_sp-PT_000126-RA TMHMM2.0 TMhelix 123 140

Fusarium_sp-PT_000126-RA TMHMM2.0 outside 141 149

Fusarium_sp-PT_000126-RA TMHMM2.0 TMhelix 150 167

Fusarium_sp-PT_000126-RA TMHMM2.0 inside 168 199

Fusarium_sp-PT_000126-RA TMHMM2.0 TMhelix 200 217

Fusarium_sp-PT_000126-RA TMHMM2.0 outside 218 226

Fusarium_sp-PT_000126-RA TMHMM2.0 TMhelix 227 244

Fusarium_sp-PT_000126-RA TMHMM2.0 inside 245 250

Fusarium_sp-PT_000126-RA TMHMM2.0 TMhelix 251 273

Fusarium_sp-PT_000126-RA TMHMM2.0 outside 274 312

Fusarium_sp-PT_000126-RA TMHMM2.0 TMhelix 313 335

Fusarium_sp-PT_000126-RA TMHMM2.0 inside 336 346

Fusarium_sp-PT_000126-RA TMHMM2.0 TMhelix 347 369

Fusarium_sp-PT_000126-RA TMHMM2.0 outside 370 388

Fusarium_sp-PT_000126-RA TMHMM2.0 TMhelix 389 411

Fusarium_sp-PT_000126-RA TMHMM2.0 inside 412 479

Fusarium_sp-PT_000126-RA TMHMM2.0 TMhelix 480 502

Fusarium_sp-PT_000126-RA TMHMM2.0 outside 503 511

Fusarium_sp-PT_000126-RA TMHMM2.0 TMhelix 512 531

Fusarium_sp-PT_000126-RA TMHMM2.0 inside 532 582

Fusarium_sp-PT_000126-RA TMHMM2.0 TMhelix 583 605

Fusarium_sp-PT_000126-RA TMHMM2.0 outside 606 619

Fusarium_sp-PT_000126-RA TMHMM2.0 TMhelix 620 642

Fusarium_sp-PT_000126-RA TMHMM2.0 inside 643 653

# Fusarium_sp-PT_000127-RA Length: 3023

# Fusarium_sp-PT_000127-RA Number of predicted TMHs: 0

# Fusarium_sp-PT_000127-RA Exp number of AAs in TMHs: 0.26847

# Fusarium_sp-PT_000127-RA Exp number, first 60 AAs: 0.00061

# Fusarium_sp-PT_000127-RA Total prob of N-in: 0.00004

Fusarium_sp-PT_000127-RA TMHMM2.0 outside 1 3023

# Fusarium_sp-PT_000129-RA Length: 873

# Fusarium_sp-PT_000129-RA Number of predicted TMHs: 0

# Fusarium_sp-PT_000129-RA Exp number of AAs in TMHs: 0.00173

# Fusarium_sp-PT_000129-RA Exp number, first 60 AAs: 0

# Fusarium_sp-PT_000129-RA Total prob of N-in: 0.00009

Fusarium_sp-PT_000129-RA TMHMM2.0 outside 1 873

# Fusarium_sp-PT_000098-RA Length: 411

# Fusarium_sp-PT_000098-RA Number of predicted TMHs: 1

# Fusarium_sp-PT_000098-RA Exp number of AAs in TMHs: 22.44116

# Fusarium_sp-PT_000098-RA Exp number, first 60 AAs: 0.00754

# Fusarium_sp-PT_000098-RA Total prob of N-in: 0.34510

Fusarium_sp-PT_000098-RA TMHMM2.0 outside 1 296

Fusarium_sp-PT_000098-RA TMHMM2.0 TMhelix 297 319

Fusarium_sp-PT_000098-RA TMHMM2.0 inside 320 411

# Fusarium_sp-PT_000119-RA Length: 139

# Fusarium_sp-PT_000119-RA Number of predicted TMHs: 3

# Fusarium_sp-PT_000119-RA Exp number of AAs in TMHs: 75.24413

# Fusarium_sp-PT_000119-RA Exp number, first 60 AAs: 28.10816

# Fusarium_sp-PT_000119-RA Total prob of N-in: 0.40702

# Fusarium_sp-PT_000119-RA POSSIBLE N-term signal sequence

Fusarium_sp-PT_000119-RA TMHMM2.0 outside 1 40

Fusarium_sp-PT_000119-RA TMHMM2.0 TMhelix 41 63

Fusarium_sp-PT_000119-RA TMHMM2.0 inside 64 83

Fusarium_sp-PT_000119-RA TMHMM2.0 TMhelix 84 106

Fusarium_sp-PT_000119-RA TMHMM2.0 outside 107 110

Fusarium_sp-PT_000119-RA TMHMM2.0 TMhelix 111 133

Fusarium_sp-PT_000119-RA TMHMM2.0 inside 134 139

# Fusarium_sp-PT_000131-RA Length: 400

# Fusarium_sp-PT_000131-RA Number of predicted TMHs: 0

# Fusarium_sp-PT_000131-RA Exp number of AAs in TMHs: 0.12661

# Fusarium_sp-PT_000131-RA Exp number, first 60 AAs: 0.09225

# Fusarium_sp-PT_000131-RA Total prob of N-in: 0.00519

Fusarium_sp-PT_000131-RA TMHMM2.0 outside 1 400

# Fusarium_sp-PT_000132-RA Length: 1226

# Fusarium_sp-PT_000132-RA Number of predicted TMHs: 0

# Fusarium_sp-PT_000132-RA Exp number of AAs in TMHs: 0.0677

# Fusarium_sp-PT_000132-RA Exp number, first 60 AAs: 0.00018

# Fusarium_sp-PT_000132-RA Total prob of N-in: 0.00236

Fusarium_sp-PT_000132-RA TMHMM2.0 outside 1 1226

# Fusarium_sp-PT_000133-RA Length: 2571

# Fusarium_sp-PT_000133-RA Number of predicted TMHs: 0

# Fusarium_sp-PT_000133-RA Exp number of AAs in TMHs: 0.43174

# Fusarium_sp-PT_000133-RA Exp number, first 60 AAs: 0

# Fusarium_sp-PT_000133-RA Total prob of N-in: 0.00001

Fusarium_sp-PT_000133-RA TMHMM2.0 outside 1 2571

# Fusarium_sp-PT_000134-RA Length: 617

# Fusarium_sp-PT_000134-RA Number of predicted TMHs: 0

# Fusarium_sp-PT_000134-RA Exp number of AAs in TMHs: 0.00034

# Fusarium_sp-PT_000134-RA Exp number, first 60 AAs: 0.00016

# Fusarium_sp-PT_000134-RA Total prob of N-in: 0.00021

Fusarium_sp-PT_000134-RA TMHMM2.0 outside 1 617

# Fusarium_sp-PT_000135-RA Length: 393

# Fusarium_sp-PT_000135-RA Number of predicted TMHs: 0

# Fusarium_sp-PT_000135-RA Exp number of AAs in TMHs: 0.20285

# Fusarium_sp-PT_000135-RA Exp number, first 60 AAs: 0.00015

# Fusarium_sp-PT_000135-RA Total prob of N-in: 0.01753

Fusarium_sp-PT_000135-RA TMHMM2.0 outside 1 393

# Fusarium_sp-PT_000137-RA Length: 338

# Fusarium_sp-PT_000137-RA Number of predicted TMHs: 0

# Fusarium_sp-PT_000137-RA Exp number of AAs in TMHs: 0.855079999999997

# Fusarium_sp-PT_000137-RA Exp number, first 60 AAs: 0.82941

# Fusarium_sp-PT_000137-RA Total prob of N-in: 0.01789

Fusarium_sp-PT_000137-RA TMHMM2.0 outside 1 338

# Fusarium_sp-PT_000138-RA Length: 873

# Fusarium_sp-PT_000138-RA Number of predicted TMHs: 14

# Fusarium_sp-PT_000138-RA Exp number of AAs in TMHs: 309.357289999999

# Fusarium_sp-PT_000138-RA Exp number, first 60 AAs: 0

# Fusarium_sp-PT_000138-RA Total prob of N-in: 0.65430

Fusarium_sp-PT_000138-RA TMHMM2.0 inside 1 231

Fusarium_sp-PT_000138-RA TMHMM2.0 TMhelix 232 254

Fusarium_sp-PT_000138-RA TMHMM2.0 outside 255 268

Fusarium_sp-PT_000138-RA TMHMM2.0 TMhelix 269 288

Fusarium_sp-PT_000138-RA TMHMM2.0 inside 289 292

Fusarium_sp-PT_000138-RA TMHMM2.0 TMhelix 293 315

Fusarium_sp-PT_000138-RA TMHMM2.0 outside 316 329

Fusarium_sp-PT_000138-RA TMHMM2.0 TMhelix 330 352

Fusarium_sp-PT_000138-RA TMHMM2.0 inside 353 417

Fusarium_sp-PT_000138-RA TMHMM2.0 TMhelix 418 437

Fusarium_sp-PT_000138-RA TMHMM2.0 outside 438 484

Fusarium_sp-PT_000138-RA TMHMM2.0 TMhelix 485 507

Fusarium_sp-PT_000138-RA TMHMM2.0 inside 508 551

Fusarium_sp-PT_000138-RA TMHMM2.0 TMhelix 552 574

Fusarium_sp-PT_000138-RA TMHMM2.0 outside 575 578

Fusarium_sp-PT_000138-RA TMHMM2.0 TMhelix 579 601

Fusarium_sp-PT_000138-RA TMHMM2.0 inside 602 607

Fusarium_sp-PT_000138-RA TMHMM2.0 TMhelix 608 630

Fusarium_sp-PT_000138-RA TMHMM2.0 outside 631 660

Fusarium_sp-PT_000138-RA TMHMM2.0 TMhelix 661 680

Fusarium_sp-PT_000138-RA TMHMM2.0 inside 681 700

Fusarium_sp-PT_000138-RA TMHMM2.0 TMhelix 701 718

Fusarium_sp-PT_000138-RA TMHMM2.0 outside 719 727

Fusarium_sp-PT_000138-RA TMHMM2.0 TMhelix 728 750

Fusarium_sp-PT_000138-RA TMHMM2.0 inside 751 770

Fusarium_sp-PT_000138-RA TMHMM2.0 TMhelix 771 793

Fusarium_sp-PT_000138-RA TMHMM2.0 outside 794 807

Fusarium_sp-PT_000138-RA TMHMM2.0 TMhelix 808 830

Fusarium_sp-PT_000138-RA TMHMM2.0 inside 831 873

# Fusarium_sp-PT_000140-RA Length: 536

# Fusarium_sp-PT_000140-RA Number of predicted TMHs: 0

# Fusarium_sp-PT_000140-RA Exp number of AAs in TMHs: 0.25602

# Fusarium_sp-PT_000140-RA Exp number, first 60 AAs: 0.00047

# Fusarium_sp-PT_000140-RA Total prob of N-in: 0.02294

Fusarium_sp-PT_000140-RA TMHMM2.0 outside 1 536

# Fusarium_sp-PT_000141-RA Length: 92

# Fusarium_sp-PT_000141-RA Number of predicted TMHs: 0

# Fusarium_sp-PT_000141-RA Exp number of AAs in TMHs: 1.19149

# Fusarium_sp-PT_000141-RA Exp number, first 60 AAs: 1.19043

# Fusarium_sp-PT_000141-RA Total prob of N-in: 0.56727

Fusarium_sp-PT_000141-RA TMHMM2.0 inside 1 92

# Fusarium_sp-PT_000144-RA Length: 168

# Fusarium_sp-PT_000144-RA Number of predicted TMHs: 0

# Fusarium_sp-PT_000144-RA Exp number of AAs in TMHs: 0.00036

# Fusarium_sp-PT_000144-RA Exp number, first 60 AAs: 0

# Fusarium_sp-PT_000144-RA Total prob of N-in: 0.06891

Fusarium_sp-PT_000144-RA TMHMM2.0 outside 1 168

# Fusarium_sp-PT_000148-RA Length: 619

# Fusarium_sp-PT_000148-RA Number of predicted TMHs: 0

# Fusarium_sp-PT_000148-RA Exp number of AAs in TMHs: 0.00601999999999999

# Fusarium_sp-PT_000148-RA Exp number, first 60 AAs: 0

# Fusarium_sp-PT_000148-RA Total prob of N-in: 0.00044

Fusarium_sp-PT_000148-RA TMHMM2.0 outside 1 619

# Fusarium_sp-PT_000150-RA Length: 547

# Fusarium_sp-PT_000150-RA Number of predicted TMHs: 0

# Fusarium_sp-PT_000150-RA Exp number of AAs in TMHs: 0.000920000000000001

# Fusarium_sp-PT_000150-RA Exp number, first 60 AAs: 0

# Fusarium_sp-PT_000150-RA Total prob of N-in: 0.00868

Fusarium_sp-PT_000150-RA TMHMM2.0 outside 1 547

# Fusarium_sp-PT_000151-RA Length: 973

# Fusarium_sp-PT_000151-RA Number of predicted TMHs: 0

# Fusarium_sp-PT_000151-RA Exp number of AAs in TMHs: 0.01101

# Fusarium_sp-PT_000151-RA Exp number, first 60 AAs: 0.00031

# Fusarium_sp-PT_000151-RA Total prob of N-in: 0.00049

Fusarium_sp-PT_000151-RA TMHMM2.0 outside 1 973

# Fusarium_sp-PT_000153-RA Length: 1359

# Fusarium_sp-PT_000153-RA Number of predicted TMHs: 0

# Fusarium_sp-PT_000153-RA Exp number of AAs in TMHs: 0.01464

# Fusarium_sp-PT_000153-RA Exp number, first 60 AAs: 0.00816

# Fusarium_sp-PT_000153-RA Total prob of N-in: 0.00079

Fusarium_sp-PT_000153-RA TMHMM2.0 outside 1 1359

# Fusarium_sp-PT_000136-RA Length: 533

# Fusarium_sp-PT_000136-RA Number of predicted TMHs: 0

# Fusarium_sp-PT_000136-RA Exp number of AAs in TMHs: 0.08426

# Fusarium_sp-PT_000136-RA Exp number, first 60 AAs: 0

# Fusarium_sp-PT_000136-RA Total prob of N-in: 0.00051

Fusarium_sp-PT_000136-RA TMHMM2.0 outside 1 533

# Fusarium_sp-PT_000139-RA Length: 185

# Fusarium_sp-PT_000139-RA Number of predicted TMHs: 0

# Fusarium_sp-PT_000139-RA Exp number of AAs in TMHs: 0.00021

# Fusarium_sp-PT_000139-RA Exp number, first 60 AAs: 0.00021

# Fusarium_sp-PT_000139-RA Total prob of N-in: 0.21638

Fusarium_sp-PT_000139-RA TMHMM2.0 outside 1 185

# Fusarium_sp-PT_000142-RA Length: 1011

# Fusarium_sp-PT_000142-RA Number of predicted TMHs: 0

# Fusarium_sp-PT_000142-RA Exp number of AAs in TMHs: 0.04066

# Fusarium_sp-PT_000142-RA Exp number, first 60 AAs: 0.01068

# Fusarium_sp-PT_000142-RA Total prob of N-in: 0.00053

Fusarium_sp-PT_000142-RA TMHMM2.0 outside 1 1011

# Fusarium_sp-PT_000143-RA Length: 207

# Fusarium_sp-PT_000143-RA Number of predicted TMHs: 1

# Fusarium_sp-PT_000143-RA Exp number of AAs in TMHs: 25.28271

# Fusarium_sp-PT_000143-RA Exp number, first 60 AAs: 0.0076

# Fusarium_sp-PT_000143-RA Total prob of N-in: 0.90298

Fusarium_sp-PT_000143-RA TMHMM2.0 inside 1 179

Fusarium_sp-PT_000143-RA TMHMM2.0 TMhelix 180 197

Fusarium_sp-PT_000143-RA TMHMM2.0 outside 198 207

# Fusarium_sp-PT_000145-RA Length: 184

# Fusarium_sp-PT_000145-RA Number of predicted TMHs: 0

# Fusarium_sp-PT_000145-RA Exp number of AAs in TMHs: 0.07099

# Fusarium_sp-PT_000145-RA Exp number, first 60 AAs: 0.07099

# Fusarium_sp-PT_000145-RA Total prob of N-in: 0.10218

Fusarium_sp-PT_000145-RA TMHMM2.0 outside 1 184

# Fusarium_sp-PT_000146-RA Length: 285

# Fusarium_sp-PT_000146-RA Number of predicted TMHs: 0

# Fusarium_sp-PT_000146-RA Exp number of AAs in TMHs: 10.55962

# Fusarium_sp-PT_000146-RA Exp number, first 60 AAs: 0.0051

# Fusarium_sp-PT_000146-RA Total prob of N-in: 0.14070

Fusarium_sp-PT_000146-RA TMHMM2.0 outside 1 285

# Fusarium_sp-PT_000147-RA Length: 447

# Fusarium_sp-PT_000147-RA Number of predicted TMHs: 12

# Fusarium_sp-PT_000147-RA Exp number of AAs in TMHs: 259.20417

# Fusarium_sp-PT_000147-RA Exp number, first 60 AAs: 6.74878

# Fusarium_sp-PT_000147-RA Total prob of N-in: 0.92741

Fusarium_sp-PT_000147-RA TMHMM2.0 inside 1 51

Fusarium_sp-PT_000147-RA TMHMM2.0 TMhelix 52 74

Fusarium_sp-PT_000147-RA TMHMM2.0 outside 75 96

Fusarium_sp-PT_000147-RA TMHMM2.0 TMhelix 97 119

Fusarium_sp-PT_000147-RA TMHMM2.0 inside 120 125

Fusarium_sp-PT_000147-RA TMHMM2.0 TMhelix 126 148

Fusarium_sp-PT_000147-RA TMHMM2.0 outside 149 151

Fusarium_sp-PT_000147-RA TMHMM2.0 TMhelix 152 174

Fusarium_sp-PT_000147-RA TMHMM2.0 inside 175 185

Fusarium_sp-PT_000147-RA TMHMM2.0 TMhelix 186 208

Fusarium_sp-PT_000147-RA TMHMM2.0 outside 209 212

Fusarium_sp-PT_000147-RA TMHMM2.0 TMhelix 213 235

Fusarium_sp-PT_000147-RA TMHMM2.0 inside 236 259

Fusarium_sp-PT_000147-RA TMHMM2.0 TMhelix 260 279

Fusarium_sp-PT_000147-RA TMHMM2.0 outside 280 293

Fusarium_sp-PT_000147-RA TMHMM2.0 TMhelix 294 316

Fusarium_sp-PT_000147-RA TMHMM2.0 inside 317 322

Fusarium_sp-PT_000147-RA TMHMM2.0 TMhelix 323 340

Fusarium_sp-PT_000147-RA TMHMM2.0 outside 341 349

Fusarium_sp-PT_000147-RA TMHMM2.0 TMhelix 350 372

Fusarium_sp-PT_000147-RA TMHMM2.0 inside 373 384

Fusarium_sp-PT_000147-RA TMHMM2.0 TMhelix 385 407

Fusarium_sp-PT_000147-RA TMHMM2.0 outside 408 411

Fusarium_sp-PT_000147-RA TMHMM2.0 TMhelix 412 434

Fusarium_sp-PT_000147-RA TMHMM2.0 inside 435 447

# Fusarium_sp-PT_000152-RA Length: 212

# Fusarium_sp-PT_000152-RA Number of predicted TMHs: 0

# Fusarium_sp-PT_000152-RA Exp number of AAs in TMHs: 0

# Fusarium_sp-PT_000152-RA Exp number, first 60 AAs: 0

# Fusarium_sp-PT_000152-RA Total prob of N-in: 0.05614

Fusarium_sp-PT_000152-RA TMHMM2.0 outside 1 212

# Fusarium_sp-PT_000149-RA Length: 65

# Fusarium_sp-PT_000149-RA Number of predicted TMHs: 1

# Fusarium_sp-PT_000149-RA Exp number of AAs in TMHs: 22.42011

# Fusarium_sp-PT_000149-RA Exp number, first 60 AAs: 22.41897

# Fusarium_sp-PT_000149-RA Total prob of N-in: 0.05787

# Fusarium_sp-PT_000149-RA POSSIBLE N-term signal sequence

Fusarium_sp-PT_000149-RA TMHMM2.0 outside 1 9

Fusarium_sp-PT_000149-RA TMHMM2.0 TMhelix 10 30

Fusarium_sp-PT_000149-RA TMHMM2.0 inside 31 65

# Fusarium_sp-PT_000155-RA Length: 661

# Fusarium_sp-PT_000155-RA Number of predicted TMHs: 2

# Fusarium_sp-PT_000155-RA Exp number of AAs in TMHs: 51.9949500000001

# Fusarium_sp-PT_000155-RA Exp number, first 60 AAs: 0

# Fusarium_sp-PT_000155-RA Total prob of N-in: 0.22547

Fusarium_sp-PT_000155-RA TMHMM2.0 outside 1 165

Fusarium_sp-PT_000155-RA TMHMM2.0 TMhelix 166 188

Fusarium_sp-PT_000155-RA TMHMM2.0 inside 189 350

Fusarium_sp-PT_000155-RA TMHMM2.0 TMhelix 351 373

Fusarium_sp-PT_000155-RA TMHMM2.0 outside 374 661

# Fusarium_sp-PT_000156-RA Length: 306

# Fusarium_sp-PT_000156-RA Number of predicted TMHs: 0

# Fusarium_sp-PT_000156-RA Exp number of AAs in TMHs: 0.0368

# Fusarium_sp-PT_000156-RA Exp number, first 60 AAs: 0

# Fusarium_sp-PT_000156-RA Total prob of N-in: 0.03068

Fusarium_sp-PT_000156-RA TMHMM2.0 outside 1 306

# Fusarium_sp-PT_000159-RA Length: 454

# Fusarium_sp-PT_000159-RA Number of predicted TMHs: 0

# Fusarium_sp-PT_000159-RA Exp number of AAs in TMHs: 0.735509999999999

# Fusarium_sp-PT_000159-RA Exp number, first 60 AAs: 0

# Fusarium_sp-PT_000159-RA Total prob of N-in: 0.02321

Fusarium_sp-PT_000159-RA TMHMM2.0 outside 1 454

# Fusarium_sp-PT_000160-RA Length: 349

# Fusarium_sp-PT_000160-RA Number of predicted TMHs: 2

# Fusarium_sp-PT_000160-RA Exp number of AAs in TMHs: 44.79012

# Fusarium_sp-PT_000160-RA Exp number, first 60 AAs: 0.00295

# Fusarium_sp-PT_000160-RA Total prob of N-in: 0.99583

Fusarium_sp-PT_000160-RA TMHMM2.0 inside 1 251

Fusarium_sp-PT_000160-RA TMHMM2.0 TMhelix 252 274

Fusarium_sp-PT_000160-RA TMHMM2.0 outside 275 288

Fusarium_sp-PT_000160-RA TMHMM2.0 TMhelix 289 310

Fusarium_sp-PT_000160-RA TMHMM2.0 inside 311 349

# Fusarium_sp-PT_000162-RA Length: 227

# Fusarium_sp-PT_000162-RA Number of predicted TMHs: 0

# Fusarium_sp-PT_000162-RA Exp number of AAs in TMHs: 0.00038

# Fusarium_sp-PT_000162-RA Exp number, first 60 AAs: 0.00038

# Fusarium_sp-PT_000162-RA Total prob of N-in: 0.07865

Fusarium_sp-PT_000162-RA TMHMM2.0 outside 1 227

# Fusarium_sp-PT_000164-RA Length: 461

# Fusarium_sp-PT_000164-RA Number of predicted TMHs: 0

# Fusarium_sp-PT_000164-RA Exp number of AAs in TMHs: 0.13521

# Fusarium_sp-PT_000164-RA Exp number, first 60 AAs: 0

# Fusarium_sp-PT_000164-RA Total prob of N-in: 0.02961

Fusarium_sp-PT_000164-RA TMHMM2.0 outside 1 461

# Fusarium_sp-PT_000165-RA Length: 559

# Fusarium_sp-PT_000165-RA Number of predicted TMHs: 11

# Fusarium_sp-PT_000165-RA Exp number of AAs in TMHs: 229.29056

# Fusarium_sp-PT_000165-RA Exp number, first 60 AAs: 18.42328

# Fusarium_sp-PT_000165-RA Total prob of N-in: 0.86025

# Fusarium_sp-PT_000165-RA POSSIBLE N-term signal sequence

Fusarium_sp-PT_000165-RA TMHMM2.0 inside 1 20

Fusarium_sp-PT_000165-RA TMHMM2.0 TMhelix 21 43

Fusarium_sp-PT_000165-RA TMHMM2.0 outside 44 79

Fusarium_sp-PT_000165-RA TMHMM2.0 TMhelix 80 97

Fusarium_sp-PT_000165-RA TMHMM2.0 inside 98 103

Fusarium_sp-PT_000165-RA TMHMM2.0 TMhelix 104 123

Fusarium_sp-PT_000165-RA TMHMM2.0 outside 124 132

Fusarium_sp-PT_000165-RA TMHMM2.0 TMhelix 133 155

Fusarium_sp-PT_000165-RA TMHMM2.0 inside 156 161

Fusarium_sp-PT_000165-RA TMHMM2.0 TMhelix 162 184

Fusarium_sp-PT_000165-RA TMHMM2.0 outside 185 198

Fusarium_sp-PT_000165-RA TMHMM2.0 TMhelix 199 221

Fusarium_sp-PT_000165-RA TMHMM2.0 inside 222 294

Fusarium_sp-PT_000165-RA TMHMM2.0 TMhelix 295 317

Fusarium_sp-PT_000165-RA TMHMM2.0 outside 318 331

Fusarium_sp-PT_000165-RA TMHMM2.0 TMhelix 332 354

Fusarium_sp-PT_000165-RA TMHMM2.0 inside 355 362

Fusarium_sp-PT_000165-RA TMHMM2.0 TMhelix 363 385

Fusarium_sp-PT_000165-RA TMHMM2.0 outside 386 406

Fusarium_sp-PT_000165-RA TMHMM2.0 TMhelix 407 429

Fusarium_sp-PT_000165-RA TMHMM2.0 inside 430 478

Fusarium_sp-PT_000165-RA TMHMM2.0 TMhelix 479 498

Fusarium_sp-PT_000165-RA TMHMM2.0 outside 499 559

# Fusarium_sp-PT_000167-RA Length: 441

# Fusarium_sp-PT_000167-RA Number of predicted TMHs: 0

# Fusarium_sp-PT_000167-RA Exp number of AAs in TMHs: 0.00412

# Fusarium_sp-PT_000167-RA Exp number, first 60 AAs: 0.00352

# Fusarium_sp-PT_000167-RA Total prob of N-in: 0.00298

Fusarium_sp-PT_000167-RA TMHMM2.0 outside 1 441

# Fusarium_sp-PT_000168-RA Length: 368

# Fusarium_sp-PT_000168-RA Number of predicted TMHs: 3

# Fusarium_sp-PT_000168-RA Exp number of AAs in TMHs: 88.6103500000001

# Fusarium_sp-PT_000168-RA Exp number, first 60 AAs: 0

# Fusarium_sp-PT_000168-RA Total prob of N-in: 0.80459

Fusarium_sp-PT_000168-RA TMHMM2.0 outside 1 197

Fusarium_sp-PT_000168-RA TMHMM2.0 TMhelix 198 220

Fusarium_sp-PT_000168-RA TMHMM2.0 inside 221 262

Fusarium_sp-PT_000168-RA TMHMM2.0 TMhelix 263 285

Fusarium_sp-PT_000168-RA TMHMM2.0 outside 286 289

Fusarium_sp-PT_000168-RA TMHMM2.0 TMhelix 290 312

Fusarium_sp-PT_000168-RA TMHMM2.0 inside 313 368

# Fusarium_sp-PT_000169-RA Length: 286

# Fusarium_sp-PT_000169-RA Number of predicted TMHs: 1

# Fusarium_sp-PT_000169-RA Exp number of AAs in TMHs: 22.99399

# Fusarium_sp-PT_000169-RA Exp number, first 60 AAs: 0.03458

# Fusarium_sp-PT_000169-RA Total prob of N-in: 0.01777

Fusarium_sp-PT_000169-RA TMHMM2.0 outside 1 191

Fusarium_sp-PT_000169-RA TMHMM2.0 TMhelix 192 214

Fusarium_sp-PT_000169-RA TMHMM2.0 inside 215 286

# Fusarium_sp-PT_000171-RA Length: 953

# Fusarium_sp-PT_000171-RA Number of predicted TMHs: 0

# Fusarium_sp-PT_000171-RA Exp number of AAs in TMHs: 0.02675

# Fusarium_sp-PT_000171-RA Exp number, first 60 AAs: 0.00034

# Fusarium_sp-PT_000171-RA Total prob of N-in: 0.00125

Fusarium_sp-PT_000171-RA TMHMM2.0 outside 1 953

# Fusarium_sp-PT_000173-RA Length: 472

# Fusarium_sp-PT_000173-RA Number of predicted TMHs: 0

# Fusarium_sp-PT_000173-RA Exp number of AAs in TMHs: 12.36681

# Fusarium_sp-PT_000173-RA Exp number, first 60 AAs: 0.00018

# Fusarium_sp-PT_000173-RA Total prob of N-in: 0.56748

Fusarium_sp-PT_000173-RA TMHMM2.0 outside 1 472

# Fusarium_sp-PT_000154-RA Length: 570

# Fusarium_sp-PT_000154-RA Number of predicted TMHs: 0

# Fusarium_sp-PT_000154-RA Exp number of AAs in TMHs: 0

# Fusarium_sp-PT_000154-RA Exp number, first 60 AAs: 0

# Fusarium_sp-PT_000154-RA Total prob of N-in: 0.00009

Fusarium_sp-PT_000154-RA TMHMM2.0 outside 1 570

# Fusarium_sp-PT_000157-RA Length: 139

# Fusarium_sp-PT_000157-RA Number of predicted TMHs: 0

# Fusarium_sp-PT_000157-RA Exp number of AAs in TMHs: 0

# Fusarium_sp-PT_000157-RA Exp number, first 60 AAs: 0

# Fusarium_sp-PT_000157-RA Total prob of N-in: 0.34204

Fusarium_sp-PT_000157-RA TMHMM2.0 outside 1 139

# Fusarium_sp-PT_000158-RA Length: 89

# Fusarium_sp-PT_000158-RA Number of predicted TMHs: 0

# Fusarium_sp-PT_000158-RA Exp number of AAs in TMHs: 0

# Fusarium_sp-PT_000158-RA Exp number, first 60 AAs: 0

# Fusarium_sp-PT_000158-RA Total prob of N-in: 0.72050

Fusarium_sp-PT_000158-RA TMHMM2.0 inside 1 89

# Fusarium_sp-PT_000161-RA Length: 632

# Fusarium_sp-PT_000161-RA Number of predicted TMHs: 0

# Fusarium_sp-PT_000161-RA Exp number of AAs in TMHs: 4.26593

# Fusarium_sp-PT_000161-RA Exp number, first 60 AAs: 4.25553

# Fusarium_sp-PT_000161-RA Total prob of N-in: 0.18826

Fusarium_sp-PT_000161-RA TMHMM2.0 outside 1 632

# Fusarium_sp-PT_000163-RA Length: 279

# Fusarium_sp-PT_000163-RA Number of predicted TMHs: 0

# Fusarium_sp-PT_000163-RA Exp number of AAs in TMHs: 0

# Fusarium_sp-PT_000163-RA Exp number, first 60 AAs: 0

# Fusarium_sp-PT_000163-RA Total prob of N-in: 0.00828

Fusarium_sp-PT_000163-RA TMHMM2.0 outside 1 279

# Fusarium_sp-PT_000166-RA Length: 392

# Fusarium_sp-PT_000166-RA Number of predicted TMHs: 0

# Fusarium_sp-PT_000166-RA Exp number of AAs in TMHs: 12.57344

# Fusarium_sp-PT_000166-RA Exp number, first 60 AAs: 1.73474

# Fusarium_sp-PT_000166-RA Total prob of N-in: 0.56449

Fusarium_sp-PT_000166-RA TMHMM2.0 outside 1 392

# Fusarium_sp-PT_000170-RA Length: 1020

# Fusarium_sp-PT_000170-RA Number of predicted TMHs: 0

# Fusarium_sp-PT_000170-RA Exp number of AAs in TMHs: 0.21475

# Fusarium_sp-PT_000170-RA Exp number, first 60 AAs: 0

# Fusarium_sp-PT_000170-RA Total prob of N-in: 0.00001

Fusarium_sp-PT_000170-RA TMHMM2.0 outside 1 1020

# Fusarium_sp-PT_000172-RA Length: 627

# Fusarium_sp-PT_000172-RA Number of predicted TMHs: 0

# Fusarium_sp-PT_000172-RA Exp number of AAs in TMHs: 0.0596900000000001

# Fusarium_sp-PT_000172-RA Exp number, first 60 AAs: 0.00381

# Fusarium_sp-PT_000172-RA Total prob of N-in: 0.00385

Fusarium_sp-PT_000172-RA TMHMM2.0 outside 1 627

# Fusarium_sp-PT_000174-RA Length: 113

# Fusarium_sp-PT_000174-RA Number of predicted TMHs: 0

# Fusarium_sp-PT_000174-RA Exp number of AAs in TMHs: 5.66102

# Fusarium_sp-PT_000174-RA Exp number, first 60 AAs: 5.66102

# Fusarium_sp-PT_000174-RA Total prob of N-in: 0.35422

Fusarium_sp-PT_000174-RA TMHMM2.0 outside 1 113

# Fusarium_sp-PT_000175-RA Length: 294

# Fusarium_sp-PT_000175-RA Number of predicted TMHs: 0

# Fusarium_sp-PT_000175-RA Exp number of AAs in TMHs: 8.02549

# Fusarium_sp-PT_000175-RA Exp number, first 60 AAs: 0.96214

# Fusarium_sp-PT_000175-RA Total prob of N-in: 0.12999

Fusarium_sp-PT_000175-RA TMHMM2.0 outside 1 294

# Fusarium_sp-PT_000178-RA Length: 513

# Fusarium_sp-PT_000178-RA Number of predicted TMHs: 1

# Fusarium_sp-PT_000178-RA Exp number of AAs in TMHs: 24.94557

# Fusarium_sp-PT_000178-RA Exp number, first 60 AAs: 22.77855

# Fusarium_sp-PT_000178-RA Total prob of N-in: 0.90667

# Fusarium_sp-PT_000178-RA POSSIBLE N-term signal sequence

Fusarium_sp-PT_000178-RA TMHMM2.0 inside 1 6

Fusarium_sp-PT_000178-RA TMHMM2.0 TMhelix 7 29

Fusarium_sp-PT_000178-RA TMHMM2.0 outside 30 513

# Fusarium_sp-PT_000180-RA Length: 1750

# Fusarium_sp-PT_000180-RA Number of predicted TMHs: 0

# Fusarium_sp-PT_000180-RA Exp number of AAs in TMHs: 0.0848599999999999

# Fusarium_sp-PT_000180-RA Exp number, first 60 AAs: 0.05809

# Fusarium_sp-PT_000180-RA Total prob of N-in: 0.00268

Fusarium_sp-PT_000180-RA TMHMM2.0 outside 1 1750

# Fusarium_sp-PT_000183-RA Length: 299

# Fusarium_sp-PT_000183-RA Number of predicted TMHs: 0

# Fusarium_sp-PT_000183-RA Exp number of AAs in TMHs: 0.20556

# Fusarium_sp-PT_000183-RA Exp number, first 60 AAs: 0.187

# Fusarium_sp-PT_000183-RA Total prob of N-in: 0.02822

Fusarium_sp-PT_000183-RA TMHMM2.0 outside 1 299

# Fusarium_sp-PT_000184-RA Length: 457

# Fusarium_sp-PT_000184-RA Number of predicted TMHs: 0

# Fusarium_sp-PT_000184-RA Exp number of AAs in TMHs: 4.02355

# Fusarium_sp-PT_000184-RA Exp number, first 60 AAs: 0

# Fusarium_sp-PT_000184-RA Total prob of N-in: 0.10576

Fusarium_sp-PT_000184-RA TMHMM2.0 outside 1 457

# Fusarium_sp-PT_000189-RA Length: 391

# Fusarium_sp-PT_000189-RA Number of predicted TMHs: 0

# Fusarium_sp-PT_000189-RA Exp number of AAs in TMHs: 2.88542000000001

# Fusarium_sp-PT_000189-RA Exp number, first 60 AAs: 0.01591

# Fusarium_sp-PT_000189-RA Total prob of N-in: 0.16899

Fusarium_sp-PT_000189-RA TMHMM2.0 outside 1 391

# Fusarium_sp-PT_000190-RA Length: 448

# Fusarium_sp-PT_000190-RA Number of predicted TMHs: 0

# Fusarium_sp-PT_000190-RA Exp number of AAs in TMHs: 28.40875

# Fusarium_sp-PT_000190-RA Exp number, first 60 AAs: 0.02463

# Fusarium_sp-PT_000190-RA Total prob of N-in: 0.26183

Fusarium_sp-PT_000190-RA TMHMM2.0 outside 1 448

# Fusarium_sp-PT_000192-RA Length: 186

# Fusarium_sp-PT_000192-RA Number of predicted TMHs: 0

# Fusarium_sp-PT_000192-RA Exp number of AAs in TMHs: 0

# Fusarium_sp-PT_000192-RA Exp number, first 60 AAs: 0

# Fusarium_sp-PT_000192-RA Total prob of N-in: 0.30759

Fusarium_sp-PT_000192-RA TMHMM2.0 outside 1 186

# Fusarium_sp-PT_000193-RA Length: 455

# Fusarium_sp-PT_000193-RA Number of predicted TMHs: 10

# Fusarium_sp-PT_000193-RA Exp number of AAs in TMHs: 216.21052

# Fusarium_sp-PT_000193-RA Exp number, first 60 AAs: 23.67396

# Fusarium_sp-PT_000193-RA Total prob of N-in: 0.49207

# Fusarium_sp-PT_000193-RA POSSIBLE N-term signal sequence

Fusarium_sp-PT_000193-RA TMHMM2.0 inside 1 39

Fusarium_sp-PT_000193-RA TMHMM2.0 TMhelix 40 62

Fusarium_sp-PT_000193-RA TMHMM2.0 outside 63 71

Fusarium_sp-PT_000193-RA TMHMM2.0 TMhelix 72 94

Fusarium_sp-PT_000193-RA TMHMM2.0 inside 95 100

Fusarium_sp-PT_000193-RA TMHMM2.0 TMhelix 101 119

Fusarium_sp-PT_000193-RA TMHMM2.0 outside 120 128

Fusarium_sp-PT_000193-RA TMHMM2.0 TMhelix 129 151

Fusarium_sp-PT_000193-RA TMHMM2.0 inside 152 157

Fusarium_sp-PT_000193-RA TMHMM2.0 TMhelix 158 180

Fusarium_sp-PT_000193-RA TMHMM2.0 outside 181 189

Fusarium_sp-PT_000193-RA TMHMM2.0 TMhelix 190 212

Fusarium_sp-PT_000193-RA TMHMM2.0 inside 213 276

Fusarium_sp-PT_000193-RA TMHMM2.0 TMhelix 277 296

Fusarium_sp-PT_000193-RA TMHMM2.0 outside 297 315

Fusarium_sp-PT_000193-RA TMHMM2.0 TMhelix 316 333

Fusarium_sp-PT_000193-RA TMHMM2.0 inside 334 345

Fusarium_sp-PT_000193-RA TMHMM2.0 TMhelix 346 368

Fusarium_sp-PT_000193-RA TMHMM2.0 outside 369 371

Fusarium_sp-PT_000193-RA TMHMM2.0 TMhelix 372 394

Fusarium_sp-PT_000193-RA TMHMM2.0 inside 395 455

# Fusarium_sp-PT_000194-RA Length: 401

# Fusarium_sp-PT_000194-RA Number of predicted TMHs: 1

# Fusarium_sp-PT_000194-RA Exp number of AAs in TMHs: 19.27526

# Fusarium_sp-PT_000194-RA Exp number, first 60 AAs: 19.27082

# Fusarium_sp-PT_000194-RA Total prob of N-in: 0.96096

# Fusarium_sp-PT_000194-RA POSSIBLE N-term signal sequence

Fusarium_sp-PT_000194-RA TMHMM2.0 inside 1 11

Fusarium_sp-PT_000194-RA TMHMM2.0 TMhelix 12 29

Fusarium_sp-PT_000194-RA TMHMM2.0 outside 30 401

# Fusarium_sp-PT_000195-RA Length: 494

# Fusarium_sp-PT_000195-RA Number of predicted TMHs: 13

# Fusarium_sp-PT_000195-RA Exp number of AAs in TMHs: 268.83875

# Fusarium_sp-PT_000195-RA Exp number, first 60 AAs: 3.33255

# Fusarium_sp-PT_000195-RA Total prob of N-in: 0.36828

Fusarium_sp-PT_000195-RA TMHMM2.0 outside 1 55

Fusarium_sp-PT_000195-RA TMHMM2.0 TMhelix 56 75

Fusarium_sp-PT_000195-RA TMHMM2.0 inside 76 86

Fusarium_sp-PT_000195-RA TMHMM2.0 TMhelix 87 109

Fusarium_sp-PT_000195-RA TMHMM2.0 outside 110 118

Fusarium_sp-PT_000195-RA TMHMM2.0 TMhelix 119 141

Fusarium_sp-PT_000195-RA TMHMM2.0 inside 142 147

Fusarium_sp-PT_000195-RA TMHMM2.0 TMhelix 148 170

Fusarium_sp-PT_000195-RA TMHMM2.0 outside 171 182

Fusarium_sp-PT_000195-RA TMHMM2.0 TMhelix 183 205

Fusarium_sp-PT_000195-RA TMHMM2.0 inside 206 216

Fusarium_sp-PT_000195-RA TMHMM2.0 TMhelix 217 239

Fusarium_sp-PT_000195-RA TMHMM2.0 outside 240 258

Fusarium_sp-PT_000195-RA TMHMM2.0 TMhelix 259 278

Fusarium_sp-PT_000195-RA TMHMM2.0 inside 279 284

Fusarium_sp-PT_000195-RA TMHMM2.0 TMhelix 285 307

Fusarium_sp-PT_000195-RA TMHMM2.0 outside 308 321

Fusarium_sp-PT_000195-RA TMHMM2.0 TMhelix 322 344

Fusarium_sp-PT_000195-RA TMHMM2.0 inside 345 364

Fusarium_sp-PT_000195-RA TMHMM2.0 TMhelix 365 382

Fusarium_sp-PT_000195-RA TMHMM2.0 outside 383 386

Fusarium_sp-PT_000195-RA TMHMM2.0 TMhelix 387 409

Fusarium_sp-PT_000195-RA TMHMM2.0 inside 410 421

Fusarium_sp-PT_000195-RA TMHMM2.0 TMhelix 422 444

Fusarium_sp-PT_000195-RA TMHMM2.0 outside 445 453

Fusarium_sp-PT_000195-RA TMHMM2.0 TMhelix 454 476

Fusarium_sp-PT_000195-RA TMHMM2.0 inside 477 494

# Fusarium_sp-PT_000198-RA Length: 213

# Fusarium_sp-PT_000198-RA Number of predicted TMHs: 0

# Fusarium_sp-PT_000198-RA Exp number of AAs in TMHs: 0.16683

# Fusarium_sp-PT_000198-RA Exp number, first 60 AAs: 0

# Fusarium_sp-PT_000198-RA Total prob of N-in: 0.04978

Fusarium_sp-PT_000198-RA TMHMM2.0 outside 1 213

# Fusarium_sp-PT_000200-RA Length: 567

# Fusarium_sp-PT_000200-RA Number of predicted TMHs: 0

# Fusarium_sp-PT_000200-RA Exp number of AAs in TMHs: 0.04051

# Fusarium_sp-PT_000200-RA Exp number, first 60 AAs: 0.03368

# Fusarium_sp-PT_000200-RA Total prob of N-in: 0.00204

Fusarium_sp-PT_000200-RA TMHMM2.0 outside 1 567

# Fusarium_sp-PT_000202-RA Length: 133

# Fusarium_sp-PT_000202-RA Number of predicted TMHs: 0

# Fusarium_sp-PT_000202-RA Exp number of AAs in TMHs: 0

# Fusarium_sp-PT_000202-RA Exp number, first 60 AAs: 0

# Fusarium_sp-PT_000202-RA Total prob of N-in: 0.21565

Fusarium_sp-PT_000202-RA TMHMM2.0 outside 1 133

# Fusarium_sp-PT_000204-RA Length: 351

# Fusarium_sp-PT_000204-RA Number of predicted TMHs: 0

# Fusarium_sp-PT_000204-RA Exp number of AAs in TMHs: 0.00333

# Fusarium_sp-PT_000204-RA Exp number, first 60 AAs: 0.00049

# Fusarium_sp-PT_000204-RA Total prob of N-in: 0.03130

Fusarium_sp-PT_000204-RA TMHMM2.0 outside 1 351

# Fusarium_sp-PT_000176-RA Length: 190

# Fusarium_sp-PT_000176-RA Number of predicted TMHs: 0

# Fusarium_sp-PT_000176-RA Exp number of AAs in TMHs: 0.01264

# Fusarium_sp-PT_000176-RA Exp number, first 60 AAs: 0.01115

# Fusarium_sp-PT_000176-RA Total prob of N-in: 0.03345

Fusarium_sp-PT_000176-RA TMHMM2.0 outside 1 190

# Fusarium_sp-PT_000177-RA Length: 178

# Fusarium_sp-PT_000177-RA Number of predicted TMHs: 1

# Fusarium_sp-PT_000177-RA Exp number of AAs in TMHs: 24.04503

# Fusarium_sp-PT_000177-RA Exp number, first 60 AAs: 1.54442

# Fusarium_sp-PT_000177-RA Total prob of N-in: 0.07299

Fusarium_sp-PT_000177-RA TMHMM2.0 outside 1 68

Fusarium_sp-PT_000177-RA TMHMM2.0 TMhelix 69 91

Fusarium_sp-PT_000177-RA TMHMM2.0 inside 92 178

# Fusarium_sp-PT_000179-RA Length: 409

# Fusarium_sp-PT_000179-RA Number of predicted TMHs: 0

# Fusarium_sp-PT_000179-RA Exp number of AAs in TMHs: 0.00669999999999998

# Fusarium_sp-PT_000179-RA Exp number, first 60 AAs: 0.0006

# Fusarium_sp-PT_000179-RA Total prob of N-in: 0.00105

Fusarium_sp-PT_000179-RA TMHMM2.0 outside 1 409

# Fusarium_sp-PT_000181-RA Length: 230

# Fusarium_sp-PT_000181-RA Number of predicted TMHs: 0

# Fusarium_sp-PT_000181-RA Exp number of AAs in TMHs: 9.25173000000001

# Fusarium_sp-PT_000181-RA Exp number, first 60 AAs: 0.84719

# Fusarium_sp-PT_000181-RA Total prob of N-in: 0.28399

Fusarium_sp-PT_000181-RA TMHMM2.0 outside 1 230

# Fusarium_sp-PT_000182-RA Length: 729

# Fusarium_sp-PT_000182-RA Number of predicted TMHs: 0

# Fusarium_sp-PT_000182-RA Exp number of AAs in TMHs: 0.13541

# Fusarium_sp-PT_000182-RA Exp number, first 60 AAs: 0

# Fusarium_sp-PT_000182-RA Total prob of N-in: 0.00228

Fusarium_sp-PT_000182-RA TMHMM2.0 outside 1 729

# Fusarium_sp-PT_000186-RA Length: 410

# Fusarium_sp-PT_000186-RA Number of predicted TMHs: 1

# Fusarium_sp-PT_000186-RA Exp number of AAs in TMHs: 17.03101

# Fusarium_sp-PT_000186-RA Exp number, first 60 AAs: 0.0439

# Fusarium_sp-PT_000186-RA Total prob of N-in: 0.63992

Fusarium_sp-PT_000186-RA TMHMM2.0 inside 1 128

Fusarium_sp-PT_000186-RA TMHMM2.0 TMhelix 129 151

Fusarium_sp-PT_000186-RA TMHMM2.0 outside 152 410

# Fusarium_sp-PT_000187-RA Length: 533

# Fusarium_sp-PT_000187-RA Number of predicted TMHs: 0

# Fusarium_sp-PT_000187-RA Exp number of AAs in TMHs: 0.01724

# Fusarium_sp-PT_000187-RA Exp number, first 60 AAs: 0.0027

# Fusarium_sp-PT_000187-RA Total prob of N-in: 0.00091

Fusarium_sp-PT_000187-RA TMHMM2.0 outside 1 533

# Fusarium_sp-PT_000188-RA Length: 482

# Fusarium_sp-PT_000188-RA Number of predicted TMHs: 2

# Fusarium_sp-PT_000188-RA Exp number of AAs in TMHs: 40.63691

# Fusarium_sp-PT_000188-RA Exp number, first 60 AAs: 0

# Fusarium_sp-PT_000188-RA Total prob of N-in: 0.91474

Fusarium_sp-PT_000188-RA TMHMM2.0 inside 1 326

Fusarium_sp-PT_000188-RA TMHMM2.0 TMhelix 327 349

Fusarium_sp-PT_000188-RA TMHMM2.0 outside 350 358

Fusarium_sp-PT_000188-RA TMHMM2.0 TMhelix 359 378

Fusarium_sp-PT_000188-RA TMHMM2.0 inside 379 482

# Fusarium_sp-PT_000191-RA Length: 264

# Fusarium_sp-PT_000191-RA Number of predicted TMHs: 0

# Fusarium_sp-PT_000191-RA Exp number of AAs in TMHs: 0

# Fusarium_sp-PT_000191-RA Exp number, first 60 AAs: 0

# Fusarium_sp-PT_000191-RA Total prob of N-in: 0.08764

Fusarium_sp-PT_000191-RA TMHMM2.0 outside 1 264

# Fusarium_sp-PT_000196-RA Length: 330

# Fusarium_sp-PT_000196-RA Number of predicted TMHs: 0

# Fusarium_sp-PT_000196-RA Exp number of AAs in TMHs: 2.11282

# Fusarium_sp-PT_000196-RA Exp number, first 60 AAs: 2.11282

# Fusarium_sp-PT_000196-RA Total prob of N-in: 0.11152

Fusarium_sp-PT_000196-RA TMHMM2.0 outside 1 330

# Fusarium_sp-PT_000199-RA Length: 514

# Fusarium_sp-PT_000199-RA Number of predicted TMHs: 0

# Fusarium_sp-PT_000199-RA Exp number of AAs in TMHs: 0.0648100000000001

# Fusarium_sp-PT_000199-RA Exp number, first 60 AAs: 0

# Fusarium_sp-PT_000199-RA Total prob of N-in: 0.00344

Fusarium_sp-PT_000199-RA TMHMM2.0 outside 1 514

# Fusarium_sp-PT_000201-RA Length: 575

# Fusarium_sp-PT_000201-RA Number of predicted TMHs: 9

# Fusarium_sp-PT_000201-RA Exp number of AAs in TMHs: 207.3371

# Fusarium_sp-PT_000201-RA Exp number, first 60 AAs: 21.93786

# Fusarium_sp-PT_000201-RA Total prob of N-in: 0.76448

# Fusarium_sp-PT_000201-RA POSSIBLE N-term signal sequence

Fusarium_sp-PT_000201-RA TMHMM2.0 inside 1 8

Fusarium_sp-PT_000201-RA TMHMM2.0 TMhelix 9 27

Fusarium_sp-PT_000201-RA TMHMM2.0 outside 28 83

Fusarium_sp-PT_000201-RA TMHMM2.0 TMhelix 84 106

Fusarium_sp-PT_000201-RA TMHMM2.0 inside 107 118

Fusarium_sp-PT_000201-RA TMHMM2.0 TMhelix 119 141

Fusarium_sp-PT_000201-RA TMHMM2.0 outside 142 145

Fusarium_sp-PT_000201-RA TMHMM2.0 TMhelix 146 168

Fusarium_sp-PT_000201-RA TMHMM2.0 inside 169 180

Fusarium_sp-PT_000201-RA TMHMM2.0 TMhelix 181 203

Fusarium_sp-PT_000201-RA TMHMM2.0 outside 204 217

Fusarium_sp-PT_000201-RA TMHMM2.0 TMhelix 218 240

Fusarium_sp-PT_000201-RA TMHMM2.0 inside 241 406

Fusarium_sp-PT_000201-RA TMHMM2.0 TMhelix 407 426

Fusarium_sp-PT_000201-RA TMHMM2.0 outside 427 452

Fusarium_sp-PT_000201-RA TMHMM2.0 TMhelix 453 475

Fusarium_sp-PT_000201-RA TMHMM2.0 inside 476 544

Fusarium_sp-PT_000201-RA TMHMM2.0 TMhelix 545 567

Fusarium_sp-PT_000201-RA TMHMM2.0 outside 568 575

# Fusarium_sp-PT_000203-RA Length: 1150

# Fusarium_sp-PT_000203-RA Number of predicted TMHs: 0

# Fusarium_sp-PT_000203-RA Exp number of AAs in TMHs: 0.01188

# Fusarium_sp-PT_000203-RA Exp number, first 60 AAs: 0.00969

# Fusarium_sp-PT_000203-RA Total prob of N-in: 0.00049

Fusarium_sp-PT_000203-RA TMHMM2.0 outside 1 1150

# Fusarium_sp-PT_000205-RA Length: 124

# Fusarium_sp-PT_000205-RA Number of predicted TMHs: 0

# Fusarium_sp-PT_000205-RA Exp number of AAs in TMHs: 0.00019

# Fusarium_sp-PT_000205-RA Exp number, first 60 AAs: 0.00019

# Fusarium_sp-PT_000205-RA Total prob of N-in: 0.12454

Fusarium_sp-PT_000205-RA TMHMM2.0 outside 1 124

# Fusarium_sp-PT_000185-RA Length: 124

# Fusarium_sp-PT_000185-RA Number of predicted TMHs: 0

# Fusarium_sp-PT_000185-RA Exp number of AAs in TMHs: 0.34946

# Fusarium_sp-PT_000185-RA Exp number, first 60 AAs: 0.34931

# Fusarium_sp-PT_000185-RA Total prob of N-in: 0.24544

Fusarium_sp-PT_000185-RA TMHMM2.0 outside 1 124

# Fusarium_sp-PT_000206-RA Length: 1117

# Fusarium_sp-PT_000206-RA Number of predicted TMHs: 0

# Fusarium_sp-PT_000206-RA Exp number of AAs in TMHs: 0.02395

# Fusarium_sp-PT_000206-RA Exp number, first 60 AAs: 0.00024

# Fusarium_sp-PT_000206-RA Total prob of N-in: 0.00073

Fusarium_sp-PT_000206-RA TMHMM2.0 outside 1 1117

# Fusarium_sp-PT_000208-RA Length: 607

# Fusarium_sp-PT_000208-RA Number of predicted TMHs: 0

# Fusarium_sp-PT_000208-RA Exp number of AAs in TMHs: 4.20524999999997

# Fusarium_sp-PT_000208-RA Exp number, first 60 AAs: 4.1113

# Fusarium_sp-PT_000208-RA Total prob of N-in: 0.17746

Fusarium_sp-PT_000208-RA TMHMM2.0 outside 1 607

# Fusarium_sp-PT_000210-RA Length: 309

# Fusarium_sp-PT_000210-RA Number of predicted TMHs: 0

# Fusarium_sp-PT_000210-RA Exp number of AAs in TMHs: 0.00244

# Fusarium_sp-PT_000210-RA Exp number, first 60 AAs: 0

# Fusarium_sp-PT_000210-RA Total prob of N-in: 0.00993

Fusarium_sp-PT_000210-RA TMHMM2.0 outside 1 309

# Fusarium_sp-PT_000212-RA Length: 337

# Fusarium_sp-PT_000212-RA Number of predicted TMHs: 0

# Fusarium_sp-PT_000212-RA Exp number of AAs in TMHs: 5.36014

# Fusarium_sp-PT_000212-RA Exp number, first 60 AAs: 5.36014

# Fusarium_sp-PT_000212-RA Total prob of N-in: 0.24599

Fusarium_sp-PT_000212-RA TMHMM2.0 outside 1 337

# Fusarium_sp-PT_000217-RA Length: 461

# Fusarium_sp-PT_000217-RA Number of predicted TMHs: 1

# Fusarium_sp-PT_000217-RA Exp number of AAs in TMHs: 23.52381

# Fusarium_sp-PT_000217-RA Exp number, first 60 AAs: 1.73279

# Fusarium_sp-PT_000217-RA Total prob of N-in: 0.14180

Fusarium_sp-PT_000217-RA TMHMM2.0 outside 1 436

Fusarium_sp-PT_000217-RA TMHMM2.0 TMhelix 437 459

Fusarium_sp-PT_000217-RA TMHMM2.0 inside 460 461

# Fusarium_sp-PT_000218-RA Length: 141

# Fusarium_sp-PT_000218-RA Number of predicted TMHs: 0

# Fusarium_sp-PT_000218-RA Exp number of AAs in TMHs: 0.00312

# Fusarium_sp-PT_000218-RA Exp number, first 60 AAs: 0.00028

# Fusarium_sp-PT_000218-RA Total prob of N-in: 0.07492

Fusarium_sp-PT_000218-RA TMHMM2.0 outside 1 141

# Fusarium_sp-PT_000220-RA Length: 677

# Fusarium_sp-PT_000220-RA Number of predicted TMHs: 0

# Fusarium_sp-PT_000220-RA Exp number of AAs in TMHs: 3.3061

# Fusarium_sp-PT_000220-RA Exp number, first 60 AAs: 3.30609

# Fusarium_sp-PT_000220-RA Total prob of N-in: 0.15236

Fusarium_sp-PT_000220-RA TMHMM2.0 outside 1 677

# Fusarium_sp-PT_000221-RA Length: 301

# Fusarium_sp-PT_000221-RA Number of predicted TMHs: 1

# Fusarium_sp-PT_000221-RA Exp number of AAs in TMHs: 23.66202

# Fusarium_sp-PT_000221-RA Exp number, first 60 AAs: 22.26464

# Fusarium_sp-PT_000221-RA Total prob of N-in: 0.26470

# Fusarium_sp-PT_000221-RA POSSIBLE N-term signal sequence

Fusarium_sp-PT_000221-RA TMHMM2.0 outside 1 37

Fusarium_sp-PT_000221-RA TMHMM2.0 TMhelix 38 60

Fusarium_sp-PT_000221-RA TMHMM2.0 inside 61 301

# Fusarium_sp-PT_000222-RA Length: 376

# Fusarium_sp-PT_000222-RA Number of predicted TMHs: 0

# Fusarium_sp-PT_000222-RA Exp number of AAs in TMHs: 0.00147

# Fusarium_sp-PT_000222-RA Exp number, first 60 AAs: 0.00147

# Fusarium_sp-PT_000222-RA Total prob of N-in: 0.00655

Fusarium_sp-PT_000222-RA TMHMM2.0 outside 1 376

# Fusarium_sp-PT_000225-RA Length: 690

# Fusarium_sp-PT_000225-RA Number of predicted TMHs: 0

# Fusarium_sp-PT_000225-RA Exp number of AAs in TMHs: 0.42939

# Fusarium_sp-PT_000225-RA Exp number, first 60 AAs: 0.05512

# Fusarium_sp-PT_000225-RA Total prob of N-in: 0.01993

Fusarium_sp-PT_000225-RA TMHMM2.0 outside 1 690

# Fusarium_sp-PT_000226-RA Length: 250

# Fusarium_sp-PT_000226-RA Number of predicted TMHs: 0

# Fusarium_sp-PT_000226-RA Exp number of AAs in TMHs: 0.0682100000000001

# Fusarium_sp-PT_000226-RA Exp number, first 60 AAs: 0.01014

# Fusarium_sp-PT_000226-RA Total prob of N-in: 0.05316

Fusarium_sp-PT_000226-RA TMHMM2.0 outside 1 250

# Fusarium_sp-PT_000229-RA Length: 515

# Fusarium_sp-PT_000229-RA Number of predicted TMHs: 11

# Fusarium_sp-PT_000229-RA Exp number of AAs in TMHs: 242.76552

# Fusarium_sp-PT_000229-RA Exp number, first 60 AAs: 0.00443

# Fusarium_sp-PT_000229-RA Total prob of N-in: 0.95995

Fusarium_sp-PT_000229-RA TMHMM2.0 inside 1 90

Fusarium_sp-PT_000229-RA TMHMM2.0 TMhelix 91 113

Fusarium_sp-PT_000229-RA TMHMM2.0 outside 114 117

Fusarium_sp-PT_000229-RA TMHMM2.0 TMhelix 118 140

Fusarium_sp-PT_000229-RA TMHMM2.0 inside 141 160

Fusarium_sp-PT_000229-RA TMHMM2.0 TMhelix 161 183

Fusarium_sp-PT_000229-RA TMHMM2.0 outside 184 197

Fusarium_sp-PT_000229-RA TMHMM2.0 TMhelix 198 217

Fusarium_sp-PT_000229-RA TMHMM2.0 inside 218 228

Fusarium_sp-PT_000229-RA TMHMM2.0 TMhelix 229 251

Fusarium_sp-PT_000229-RA TMHMM2.0 outside 252 279

Fusarium_sp-PT_000229-RA TMHMM2.0 TMhelix 280 302

Fusarium_sp-PT_000229-RA TMHMM2.0 inside 303 313

Fusarium_sp-PT_000229-RA TMHMM2.0 TMhelix 314 336

Fusarium_sp-PT_000229-RA TMHMM2.0 outside 337 359

Fusarium_sp-PT_000229-RA TMHMM2.0 TMhelix 360 382

Fusarium_sp-PT_000229-RA TMHMM2.0 inside 383 394

Fusarium_sp-PT_000229-RA TMHMM2.0 TMhelix 395 417

Fusarium_sp-PT_000229-RA TMHMM2.0 outside 418 426

Fusarium_sp-PT_000229-RA TMHMM2.0 TMhelix 427 449

Fusarium_sp-PT_000229-RA TMHMM2.0 inside 450 469

Fusarium_sp-PT_000229-RA TMHMM2.0 TMhelix 470 492

Fusarium_sp-PT_000229-RA TMHMM2.0 outside 493 515

# Fusarium_sp-PT_000230-RA Length: 375

# Fusarium_sp-PT_000230-RA Number of predicted TMHs: 5

# Fusarium_sp-PT_000230-RA Exp number of AAs in TMHs: 103.88546

# Fusarium_sp-PT_000230-RA Exp number, first 60 AAs: 0.00083

# Fusarium_sp-PT_000230-RA Total prob of N-in: 0.95942

Fusarium_sp-PT_000230-RA TMHMM2.0 inside 1 69

Fusarium_sp-PT_000230-RA TMHMM2.0 TMhelix 70 87

Fusarium_sp-PT_000230-RA TMHMM2.0 outside 88 96

Fusarium_sp-PT_000230-RA TMHMM2.0 TMhelix 97 119

Fusarium_sp-PT_000230-RA TMHMM2.0 inside 120 131

Fusarium_sp-PT_000230-RA TMHMM2.0 TMhelix 132 149

Fusarium_sp-PT_000230-RA TMHMM2.0 outside 150 174

Fusarium_sp-PT_000230-RA TMHMM2.0 TMhelix 175 197

Fusarium_sp-PT_000230-RA TMHMM2.0 inside 198 222

Fusarium_sp-PT_000230-RA TMHMM2.0 TMhelix 223 245

Fusarium_sp-PT_000230-RA TMHMM2.0 outside 246 375

# Fusarium_sp-PT_000232-RA Length: 250

# Fusarium_sp-PT_000232-RA Number of predicted TMHs: 0

# Fusarium_sp-PT_000232-RA Exp number of AAs in TMHs: 0.04152

# Fusarium_sp-PT_000232-RA Exp number, first 60 AAs: 0.01559

# Fusarium_sp-PT_000232-RA Total prob of N-in: 0.09079

Fusarium_sp-PT_000232-RA TMHMM2.0 outside 1 250

# Fusarium_sp-PT_000207-RA Length: 137

# Fusarium_sp-PT_000207-RA Number of predicted TMHs: 0

# Fusarium_sp-PT_000207-RA Exp number of AAs in TMHs: 0.00536

# Fusarium_sp-PT_000207-RA Exp number, first 60 AAs: 0.00196

# Fusarium_sp-PT_000207-RA Total prob of N-in: 0.01782

Fusarium_sp-PT_000207-RA TMHMM2.0 outside 1 137

# Fusarium_sp-PT_000209-RA Length: 530

# Fusarium_sp-PT_000209-RA Number of predicted TMHs: 11

# Fusarium_sp-PT_000209-RA Exp number of AAs in TMHs: 242.76681

# Fusarium_sp-PT_000209-RA Exp number, first 60 AAs: 14.98958

# Fusarium_sp-PT_000209-RA Total prob of N-in: 0.17262

# Fusarium_sp-PT_000209-RA POSSIBLE N-term signal sequence

Fusarium_sp-PT_000209-RA TMHMM2.0 outside 1 44

Fusarium_sp-PT_000209-RA TMHMM2.0 TMhelix 45 67

Fusarium_sp-PT_000209-RA TMHMM2.0 inside 68 73

Fusarium_sp-PT_000209-RA TMHMM2.0 TMhelix 74 96

Fusarium_sp-PT_000209-RA TMHMM2.0 outside 97 149

Fusarium_sp-PT_000209-RA TMHMM2.0 TMhelix 150 172

Fusarium_sp-PT_000209-RA TMHMM2.0 inside 173 178

Fusarium_sp-PT_000209-RA TMHMM2.0 TMhelix 179 201

Fusarium_sp-PT_000209-RA TMHMM2.0 outside 202 220

Fusarium_sp-PT_000209-RA TMHMM2.0 TMhelix 221 243

Fusarium_sp-PT_000209-RA TMHMM2.0 inside 244 266

Fusarium_sp-PT_000209-RA TMHMM2.0 TMhelix 267 289

Fusarium_sp-PT_000209-RA TMHMM2.0 outside 290 314

Fusarium_sp-PT_000209-RA TMHMM2.0 TMhelix 315 337

Fusarium_sp-PT_000209-RA TMHMM2.0 inside 338 363

Fusarium_sp-PT_000209-RA TMHMM2.0 TMhelix 364 386

Fusarium_sp-PT_000209-RA TMHMM2.0 outside 387 390

Fusarium_sp-PT_000209-RA TMHMM2.0 TMhelix 391 413

Fusarium_sp-PT_000209-RA TMHMM2.0 inside 414 433

Fusarium_sp-PT_000209-RA TMHMM2.0 TMhelix 434 456

Fusarium_sp-PT_000209-RA TMHMM2.0 outside 457 465

Fusarium_sp-PT_000209-RA TMHMM2.0 TMhelix 466 488

Fusarium_sp-PT_000209-RA TMHMM2.0 inside 489 530

# Fusarium_sp-PT_000211-RA Length: 927

# Fusarium_sp-PT_000211-RA Number of predicted TMHs: 0

# Fusarium_sp-PT_000211-RA Exp number of AAs in TMHs: 0.00185

# Fusarium_sp-PT_000211-RA Exp number, first 60 AAs: 0

# Fusarium_sp-PT_000211-RA Total prob of N-in: 0.00001

Fusarium_sp-PT_000211-RA TMHMM2.0 outside 1 927

# Fusarium_sp-PT_000214-RA Length: 1055

# Fusarium_sp-PT_000214-RA Number of predicted TMHs: 0

# Fusarium_sp-PT_000214-RA Exp number of AAs in TMHs: 0.03985

# Fusarium_sp-PT_000214-RA Exp number, first 60 AAs: 0

# Fusarium_sp-PT_000214-RA Total prob of N-in: 0.00067

Fusarium_sp-PT_000214-RA TMHMM2.0 outside 1 1055

# Fusarium_sp-PT_000215-RA Length: 376

# Fusarium_sp-PT_000215-RA Number of predicted TMHs: 0

# Fusarium_sp-PT_000215-RA Exp number of AAs in TMHs: 20.34274

# Fusarium_sp-PT_000215-RA Exp number, first 60 AAs: 9.61622

# Fusarium_sp-PT_000215-RA Total prob of N-in: 0.54612

Fusarium_sp-PT_000215-RA TMHMM2.0 outside 1 376

# Fusarium_sp-PT_000219-RA Length: 380

# Fusarium_sp-PT_000219-RA Number of predicted TMHs: 0

# Fusarium_sp-PT_000219-RA Exp number of AAs in TMHs: 0.07926

# Fusarium_sp-PT_000219-RA Exp number, first 60 AAs: 0.00192

# Fusarium_sp-PT_000219-RA Total prob of N-in: 0.00677

Fusarium_sp-PT_000219-RA TMHMM2.0 outside 1 380

# Fusarium_sp-PT_000223-RA Length: 1412

# Fusarium_sp-PT_000223-RA Number of predicted TMHs: 0

# Fusarium_sp-PT_000223-RA Exp number of AAs in TMHs: 0.01287

# Fusarium_sp-PT_000223-RA Exp number, first 60 AAs: 0.00033

# Fusarium_sp-PT_000223-RA Total prob of N-in: 0.00039

Fusarium_sp-PT_000223-RA TMHMM2.0 outside 1 1412

# Fusarium_sp-PT_000224-RA Length: 478

# Fusarium_sp-PT_000224-RA Number of predicted TMHs: 0

# Fusarium_sp-PT_000224-RA Exp number of AAs in TMHs: 0

# Fusarium_sp-PT_000224-RA Exp number, first 60 AAs: 0

# Fusarium_sp-PT_000224-RA Total prob of N-in: 0.00013

Fusarium_sp-PT_000224-RA TMHMM2.0 outside 1 478

# Fusarium_sp-PT_000227-RA Length: 338

# Fusarium_sp-PT_000227-RA Number of predicted TMHs: 0

# Fusarium_sp-PT_000227-RA Exp number of AAs in TMHs: 0.03276

# Fusarium_sp-PT_000227-RA Exp number, first 60 AAs: 0.01643

# Fusarium_sp-PT_000227-RA Total prob of N-in: 0.03855

Fusarium_sp-PT_000227-RA TMHMM2.0 outside 1 338

# Fusarium_sp-PT_000228-RA Length: 432

# Fusarium_sp-PT_000228-RA Number of predicted TMHs: 10

# Fusarium_sp-PT_000228-RA Exp number of AAs in TMHs: 204.95059

# Fusarium_sp-PT_000228-RA Exp number, first 60 AAs: 42.54965

# Fusarium_sp-PT_000228-RA Total prob of N-in: 0.99323

# Fusarium_sp-PT_000228-RA POSSIBLE N-term signal sequence

Fusarium_sp-PT_000228-RA TMHMM2.0 inside 1 1

Fusarium_sp-PT_000228-RA TMHMM2.0 TMhelix 2 24

Fusarium_sp-PT_000228-RA TMHMM2.0 outside 25 33

Fusarium_sp-PT_000228-RA TMHMM2.0 TMhelix 34 53

Fusarium_sp-PT_000228-RA TMHMM2.0 inside 54 65

Fusarium_sp-PT_000228-RA TMHMM2.0 TMhelix 66 88

Fusarium_sp-PT_000228-RA TMHMM2.0 outside 89 138

Fusarium_sp-PT_000228-RA TMHMM2.0 TMhelix 139 161

Fusarium_sp-PT_000228-RA TMHMM2.0 inside 162 173

Fusarium_sp-PT_000228-RA TMHMM2.0 TMhelix 174 196

Fusarium_sp-PT_000228-RA TMHMM2.0 outside 197 205

Fusarium_sp-PT_000228-RA TMHMM2.0 TMhelix 206 225

Fusarium_sp-PT_000228-RA TMHMM2.0 inside 226 231

Fusarium_sp-PT_000228-RA TMHMM2.0 TMhelix 232 249

Fusarium_sp-PT_000228-RA TMHMM2.0 outside 250 297

Fusarium_sp-PT_000228-RA TMHMM2.0 TMhelix 298 320

Fusarium_sp-PT_000228-RA TMHMM2.0 inside 321 360

Fusarium_sp-PT_000228-RA TMHMM2.0 TMhelix 361 383

Fusarium_sp-PT_000228-RA TMHMM2.0 outside 384 402

Fusarium_sp-PT_000228-RA TMHMM2.0 TMhelix 403 422

Fusarium_sp-PT_000228-RA TMHMM2.0 inside 423 432

# Fusarium_sp-PT_000231-RA Length: 287

# Fusarium_sp-PT_000231-RA Number of predicted TMHs: 1

# Fusarium_sp-PT_000231-RA Exp number of AAs in TMHs: 16.97303

# Fusarium_sp-PT_000231-RA Exp number, first 60 AAs: 0.00013

# Fusarium_sp-PT_000231-RA Total prob of N-in: 0.04126

Fusarium_sp-PT_000231-RA TMHMM2.0 outside 1 266

Fusarium_sp-PT_000231-RA TMHMM2.0 TMhelix 267 286

Fusarium_sp-PT_000231-RA TMHMM2.0 inside 287 287

# Fusarium_sp-PT_000213-RA Length: 78

# Fusarium_sp-PT_000213-RA Number of predicted TMHs: 0

# Fusarium_sp-PT_000213-RA Exp number of AAs in TMHs: 0.0047

# Fusarium_sp-PT_000213-RA Exp number, first 60 AAs: 0.00302

# Fusarium_sp-PT_000213-RA Total prob of N-in: 0.35963

Fusarium_sp-PT_000213-RA TMHMM2.0 outside 1 78

# Fusarium_sp-PT_000216-RA Length: 61

# Fusarium_sp-PT_000216-RA Number of predicted TMHs: 0

# Fusarium_sp-PT_000216-RA Exp number of AAs in TMHs: 0

# Fusarium_sp-PT_000216-RA Exp number, first 60 AAs: 0

# Fusarium_sp-PT_000216-RA Total prob of N-in: 0.69016

Fusarium_sp-PT_000216-RA TMHMM2.0 inside 1 61

# Fusarium_sp-PT_000233-RA Length: 317

# Fusarium_sp-PT_000233-RA Number of predicted TMHs: 0

# Fusarium_sp-PT_000233-RA Exp number of AAs in TMHs: 0.00018

# Fusarium_sp-PT_000233-RA Exp number, first 60 AAs: 0

# Fusarium_sp-PT_000233-RA Total prob of N-in: 0.00580

Fusarium_sp-PT_000233-RA TMHMM2.0 outside 1 317

# Fusarium_sp-PT_000236-RA Length: 453

# Fusarium_sp-PT_000236-RA Number of predicted TMHs: 0

# Fusarium_sp-PT_000236-RA Exp number of AAs in TMHs: 0.17946

# Fusarium_sp-PT_000236-RA Exp number, first 60 AAs: 0

# Fusarium_sp-PT_000236-RA Total prob of N-in: 0.00271

Fusarium_sp-PT_000236-RA TMHMM2.0 outside 1 453

# Fusarium_sp-PT_000238-RA Length: 382

# Fusarium_sp-PT_000238-RA Number of predicted TMHs: 0

# Fusarium_sp-PT_000238-RA Exp number of AAs in TMHs: 10.5627

# Fusarium_sp-PT_000238-RA Exp number, first 60 AAs: 0

# Fusarium_sp-PT_000238-RA Total prob of N-in: 0.28946

Fusarium_sp-PT_000238-RA TMHMM2.0 outside 1 382

# Fusarium_sp-PT_000239-RA Length: 263

# Fusarium_sp-PT_000239-RA Number of predicted TMHs: 7

# Fusarium_sp-PT_000239-RA Exp number of AAs in TMHs: 149.36417

# Fusarium_sp-PT_000239-RA Exp number, first 60 AAs: 42.56727

# Fusarium_sp-PT_000239-RA Total prob of N-in: 0.06821

# Fusarium_sp-PT_000239-RA POSSIBLE N-term signal sequence

Fusarium_sp-PT_000239-RA TMHMM2.0 outside 1 4

Fusarium_sp-PT_000239-RA TMHMM2.0 TMhelix 5 24

Fusarium_sp-PT_000239-RA TMHMM2.0 inside 25 30

Fusarium_sp-PT_000239-RA TMHMM2.0 TMhelix 31 53

Fusarium_sp-PT_000239-RA TMHMM2.0 outside 54 57

Fusarium_sp-PT_000239-RA TMHMM2.0 TMhelix 58 80

Fusarium_sp-PT_000239-RA TMHMM2.0 inside 81 100

Fusarium_sp-PT_000239-RA TMHMM2.0 TMhelix 101 118

Fusarium_sp-PT_000239-RA TMHMM2.0 outside 119 132

Fusarium_sp-PT_000239-RA TMHMM2.0 TMhelix 133 155

Fusarium_sp-PT_000239-RA TMHMM2.0 inside 156 175

Fusarium_sp-PT_000239-RA TMHMM2.0 TMhelix 176 198

Fusarium_sp-PT_000239-RA TMHMM2.0 outside 199 217

Fusarium_sp-PT_000239-RA TMHMM2.0 TMhelix 218 237

Fusarium_sp-PT_000239-RA TMHMM2.0 inside 238 263

# Fusarium_sp-PT_000240-RA Length: 96

# Fusarium_sp-PT_000240-RA Number of predicted TMHs: 0

# Fusarium_sp-PT_000240-RA Exp number of AAs in TMHs: 0.0032

# Fusarium_sp-PT_000240-RA Exp number, first 60 AAs: 0.0032

# Fusarium_sp-PT_000240-RA Total prob of N-in: 0.08694

Fusarium_sp-PT_000240-RA TMHMM2.0 outside 1 96

# Fusarium_sp-PT_000242-RA Length: 734

# Fusarium_sp-PT_000242-RA Number of predicted TMHs: 0

# Fusarium_sp-PT_000242-RA Exp number of AAs in TMHs: 0.00197

# Fusarium_sp-PT_000242-RA Exp number, first 60 AAs: 0.00018

# Fusarium_sp-PT_000242-RA Total prob of N-in: 0.00005

Fusarium_sp-PT_000242-RA TMHMM2.0 outside 1 734

# Fusarium_sp-PT_000244-RA Length: 166

# Fusarium_sp-PT_000244-RA Number of predicted TMHs: 0

# Fusarium_sp-PT_000244-RA Exp number of AAs in TMHs: 0.00048

# Fusarium_sp-PT_000244-RA Exp number, first 60 AAs: 0

# Fusarium_sp-PT_000244-RA Total prob of N-in: 0.10656

Fusarium_sp-PT_000244-RA TMHMM2.0 outside 1 166

# Fusarium_sp-PT_000245-RA Length: 542

# Fusarium_sp-PT_000245-RA Number of predicted TMHs: 12

# Fusarium_sp-PT_000245-RA Exp number of AAs in TMHs: 260.61707

# Fusarium_sp-PT_000245-RA Exp number, first 60 AAs: 1.88594

# Fusarium_sp-PT_000245-RA Total prob of N-in: 0.95629

Fusarium_sp-PT_000245-RA TMHMM2.0 inside 1 58

Fusarium_sp-PT_000245-RA TMHMM2.0 TMhelix 59 81

Fusarium_sp-PT_000245-RA TMHMM2.0 outside 82 100

Fusarium_sp-PT_000245-RA TMHMM2.0 TMhelix 101 123

Fusarium_sp-PT_000245-RA TMHMM2.0 inside 124 129

Fusarium_sp-PT_000245-RA TMHMM2.0 TMhelix 130 147

Fusarium_sp-PT_000245-RA TMHMM2.0 outside 148 156

Fusarium_sp-PT_000245-RA TMHMM2.0 TMhelix 157 179

Fusarium_sp-PT_000245-RA TMHMM2.0 inside 180 190

Fusarium_sp-PT_000245-RA TMHMM2.0 TMhelix 191 213

Fusarium_sp-PT_000245-RA TMHMM2.0 outside 214 222

Fusarium_sp-PT_000245-RA TMHMM2.0 TMhelix 223 245

Fusarium_sp-PT_000245-RA TMHMM2.0 inside 246 312

Fusarium_sp-PT_000245-RA TMHMM2.0 TMhelix 313 335

Fusarium_sp-PT_000245-RA TMHMM2.0 outside 336 339

Fusarium_sp-PT_000245-RA TMHMM2.0 TMhelix 340 362

Fusarium_sp-PT_000245-RA TMHMM2.0 inside 363 374

Fusarium_sp-PT_000245-RA TMHMM2.0 TMhelix 375 395

Fusarium_sp-PT_000245-RA TMHMM2.0 outside 396 404

Fusarium_sp-PT_000245-RA TMHMM2.0 TMhelix 405 427

Fusarium_sp-PT_000245-RA TMHMM2.0 inside 428 433

Fusarium_sp-PT_000245-RA TMHMM2.0 TMhelix 434 456

Fusarium_sp-PT_000245-RA TMHMM2.0 outside 457 475

Fusarium_sp-PT_000245-RA TMHMM2.0 TMhelix 476 498

Fusarium_sp-PT_000245-RA TMHMM2.0 inside 499 542

# Fusarium_sp-PT_000247-RA Length: 474

# Fusarium_sp-PT_000247-RA Number of predicted TMHs: 0

# Fusarium_sp-PT_000247-RA Exp number of AAs in TMHs: 0.00064

# Fusarium_sp-PT_000247-RA Exp number, first 60 AAs: 0

# Fusarium_sp-PT_000247-RA Total prob of N-in: 0.00230

Fusarium_sp-PT_000247-RA TMHMM2.0 outside 1 474

# Fusarium_sp-PT_000249-RA Length: 394

# Fusarium_sp-PT_000249-RA Number of predicted TMHs: 0

# Fusarium_sp-PT_000249-RA Exp number of AAs in TMHs: 0

# Fusarium_sp-PT_000249-RA Exp number, first 60 AAs: 0

# Fusarium_sp-PT_000249-RA Total prob of N-in: 0.00370

Fusarium_sp-PT_000249-RA TMHMM2.0 outside 1 394

# Fusarium_sp-PT_000250-RA Length: 1189

# Fusarium_sp-PT_000250-RA Number of predicted TMHs: 0

# Fusarium_sp-PT_000250-RA Exp number of AAs in TMHs: 0.1532

# Fusarium_sp-PT_000250-RA Exp number, first 60 AAs: 0

# Fusarium_sp-PT_000250-RA Total prob of N-in: 0.00022

Fusarium_sp-PT_000250-RA TMHMM2.0 outside 1 1189

# Fusarium_sp-PT_000253-RA Length: 625

# Fusarium_sp-PT_000253-RA Number of predicted TMHs: 0

# Fusarium_sp-PT_000253-RA Exp number of AAs in TMHs: 0.00039

# Fusarium_sp-PT_000253-RA Exp number, first 60 AAs: 0

# Fusarium_sp-PT_000253-RA Total prob of N-in: 0.00096

Fusarium_sp-PT_000253-RA TMHMM2.0 outside 1 625

# Fusarium_sp-PT_000254-RA Length: 234

# Fusarium_sp-PT_000254-RA Number of predicted TMHs: 0

# Fusarium_sp-PT_000254-RA Exp number of AAs in TMHs: 0

# Fusarium_sp-PT_000254-RA Exp number, first 60 AAs: 0

# Fusarium_sp-PT_000254-RA Total prob of N-in: 0.20647

Fusarium_sp-PT_000254-RA TMHMM2.0 outside 1 234

# Fusarium_sp-PT_000256-RA Length: 586

# Fusarium_sp-PT_000256-RA Number of predicted TMHs: 0

# Fusarium_sp-PT_000256-RA Exp number of AAs in TMHs: 0.00131

# Fusarium_sp-PT_000256-RA Exp number, first 60 AAs: 0

# Fusarium_sp-PT_000256-RA Total prob of N-in: 0.00348

Fusarium_sp-PT_000256-RA TMHMM2.0 outside 1 586

# Fusarium_sp-PT_000257-RA Length: 463

# Fusarium_sp-PT_000257-RA Number of predicted TMHs: 0

# Fusarium_sp-PT_000257-RA Exp number of AAs in TMHs: 0.01727

# Fusarium_sp-PT_000257-RA Exp number, first 60 AAs: 0.01307

# Fusarium_sp-PT_000257-RA Total prob of N-in: 0.00216

Fusarium_sp-PT_000257-RA TMHMM2.0 outside 1 463

# Fusarium_sp-PT_000260-RA Length: 1456

# Fusarium_sp-PT_000260-RA Number of predicted TMHs: 0

# Fusarium_sp-PT_000260-RA Exp number of AAs in TMHs: 0

# Fusarium_sp-PT_000260-RA Exp number, first 60 AAs: 0

# Fusarium_sp-PT_000260-RA Total prob of N-in: 0.00000

Fusarium_sp-PT_000260-RA TMHMM2.0 outside 1 1456

# Fusarium_sp-PT_000262-RA Length: 617

# Fusarium_sp-PT_000262-RA Number of predicted TMHs: 0

# Fusarium_sp-PT_000262-RA Exp number of AAs in TMHs: 12.09583

# Fusarium_sp-PT_000262-RA Exp number, first 60 AAs: 11.25544

# Fusarium_sp-PT_000262-RA Total prob of N-in: 0.56078

# Fusarium_sp-PT_000262-RA POSSIBLE N-term signal sequence

Fusarium_sp-PT_000262-RA TMHMM2.0 outside 1 617

# Fusarium_sp-PT_000234-RA Length: 179

# Fusarium_sp-PT_000234-RA Number of predicted TMHs: 0

# Fusarium_sp-PT_000234-RA Exp number of AAs in TMHs: 13.53645

# Fusarium_sp-PT_000234-RA Exp number, first 60 AAs: 1.8263

# Fusarium_sp-PT_000234-RA Total prob of N-in: 0.52141

Fusarium_sp-PT_000234-RA TMHMM2.0 outside 1 179

# Fusarium_sp-PT_000235-RA Length: 109

# Fusarium_sp-PT_000235-RA Number of predicted TMHs: 0

# Fusarium_sp-PT_000235-RA Exp number of AAs in TMHs: 0.27943

# Fusarium_sp-PT_000235-RA Exp number, first 60 AAs: 0.02239

# Fusarium_sp-PT_000235-RA Total prob of N-in: 0.11770

Fusarium_sp-PT_000235-RA TMHMM2.0 outside 1 109

# Fusarium_sp-PT_000237-RA Length: 140

# Fusarium_sp-PT_000237-RA Number of predicted TMHs: 0

# Fusarium_sp-PT_000237-RA Exp number of AAs in TMHs: 0.05926

# Fusarium_sp-PT_000237-RA Exp number, first 60 AAs: 0.05926

# Fusarium_sp-PT_000237-RA Total prob of N-in: 0.08764

Fusarium_sp-PT_000237-RA TMHMM2.0 outside 1 140

# Fusarium_sp-PT_000241-RA Length: 515

# Fusarium_sp-PT_000241-RA Number of predicted TMHs: 0

# Fusarium_sp-PT_000241-RA Exp number of AAs in TMHs: 17.38255

# Fusarium_sp-PT_000241-RA Exp number, first 60 AAs: 0.82625

# Fusarium_sp-PT_000241-RA Total prob of N-in: 0.50766

Fusarium_sp-PT_000241-RA TMHMM2.0 outside 1 515

# Fusarium_sp-PT_000243-RA Length: 332

# Fusarium_sp-PT_000243-RA Number of predicted TMHs: 0

# Fusarium_sp-PT_000243-RA Exp number of AAs in TMHs: 0

# Fusarium_sp-PT_000243-RA Exp number, first 60 AAs: 0

# Fusarium_sp-PT_000243-RA Total prob of N-in: 0.00800

Fusarium_sp-PT_000243-RA TMHMM2.0 outside 1 332

# Fusarium_sp-PT_000246-RA Length: 516

# Fusarium_sp-PT_000246-RA Number of predicted TMHs: 0

# Fusarium_sp-PT_000246-RA Exp number of AAs in TMHs: 0.22281

# Fusarium_sp-PT_000246-RA Exp number, first 60 AAs: 0.21927

# Fusarium_sp-PT_000246-RA Total prob of N-in: 0.01355

Fusarium_sp-PT_000246-RA TMHMM2.0 outside 1 516

# Fusarium_sp-PT_000248-RA Length: 207

# Fusarium_sp-PT_000248-RA Number of predicted TMHs: 0

# Fusarium_sp-PT_000248-RA Exp number of AAs in TMHs: 0.00108

# Fusarium_sp-PT_000248-RA Exp number, first 60 AAs: 0.00093

# Fusarium_sp-PT_000248-RA Total prob of N-in: 0.04932

Fusarium_sp-PT_000248-RA TMHMM2.0 outside 1 207

# Fusarium_sp-PT_000251-RA Length: 372

# Fusarium_sp-PT_000251-RA Number of predicted TMHs: 0

# Fusarium_sp-PT_000251-RA Exp number of AAs in TMHs: 0.00161

# Fusarium_sp-PT_000251-RA Exp number, first 60 AAs: 0.00139

# Fusarium_sp-PT_000251-RA Total prob of N-in: 0.01657

Fusarium_sp-PT_000251-RA TMHMM2.0 outside 1 372

# Fusarium_sp-PT_000252-RA Length: 153

# Fusarium_sp-PT_000252-RA Number of predicted TMHs: 0

# Fusarium_sp-PT_000252-RA Exp number of AAs in TMHs: 0.02939

# Fusarium_sp-PT_000252-RA Exp number, first 60 AAs: 0.0285

# Fusarium_sp-PT_000252-RA Total prob of N-in: 0.14160

Fusarium_sp-PT_000252-RA TMHMM2.0 outside 1 153

# Fusarium_sp-PT_000258-RA Length: 337

# Fusarium_sp-PT_000258-RA Number of predicted TMHs: 0

# Fusarium_sp-PT_000258-RA Exp number of AAs in TMHs: 0

# Fusarium_sp-PT_000258-RA Exp number, first 60 AAs: 0

# Fusarium_sp-PT_000258-RA Total prob of N-in: 0.01254

Fusarium_sp-PT_000258-RA TMHMM2.0 outside 1 337

# Fusarium_sp-PT_000259-RA Length: 705

# Fusarium_sp-PT_000259-RA Number of predicted TMHs: 0

# Fusarium_sp-PT_000259-RA Exp number of AAs in TMHs: 0.643409999999998

# Fusarium_sp-PT_000259-RA Exp number, first 60 AAs: 0

# Fusarium_sp-PT_000259-RA Total prob of N-in: 0.03458

Fusarium_sp-PT_000259-RA TMHMM2.0 outside 1 705

# Fusarium_sp-PT_000261-RA Length: 249

# Fusarium_sp-PT_000261-RA Number of predicted TMHs: 0

# Fusarium_sp-PT_000261-RA Exp number of AAs in TMHs: 0.40895

# Fusarium_sp-PT_000261-RA Exp number, first 60 AAs: 0

# Fusarium_sp-PT_000261-RA Total prob of N-in: 0.01374

Fusarium_sp-PT_000261-RA TMHMM2.0 outside 1 249

# Fusarium_sp-PT_000263-RA Length: 332

# Fusarium_sp-PT_000263-RA Number of predicted TMHs: 0

# Fusarium_sp-PT_000263-RA Exp number of AAs in TMHs: 0.13377

# Fusarium_sp-PT_000263-RA Exp number, first 60 AAs: 0.00455

# Fusarium_sp-PT_000263-RA Total prob of N-in: 0.00438

Fusarium_sp-PT_000263-RA TMHMM2.0 outside 1 332

# Fusarium_sp-PT_000264-RA Length: 514

# Fusarium_sp-PT_000264-RA Number of predicted TMHs: 1

# Fusarium_sp-PT_000264-RA Exp number of AAs in TMHs: 20.92747

# Fusarium_sp-PT_000264-RA Exp number, first 60 AAs: 20.50439

# Fusarium_sp-PT_000264-RA Total prob of N-in: 0.95955

# Fusarium_sp-PT_000264-RA POSSIBLE N-term signal sequence

Fusarium_sp-PT_000264-RA TMHMM2.0 inside 1 16

Fusarium_sp-PT_000264-RA TMHMM2.0 TMhelix 17 36

Fusarium_sp-PT_000264-RA TMHMM2.0 outside 37 514

# Fusarium_sp-PT_000255-RA Length: 575

# Fusarium_sp-PT_000255-RA Number of predicted TMHs: 0

# Fusarium_sp-PT_000255-RA Exp number of AAs in TMHs: 0

# Fusarium_sp-PT_000255-RA Exp number, first 60 AAs: 0

# Fusarium_sp-PT_000255-RA Total prob of N-in: 0.00117

Fusarium_sp-PT_000255-RA TMHMM2.0 outside 1 575

# Fusarium_sp-PT_000265-RA Length: 91

# Fusarium_sp-PT_000265-RA Number of predicted TMHs: 0

# Fusarium_sp-PT_000265-RA Exp number of AAs in TMHs: 0

# Fusarium_sp-PT_000265-RA Exp number, first 60 AAs: 0

# Fusarium_sp-PT_000265-RA Total prob of N-in: 0.35977

Fusarium_sp-PT_000265-RA TMHMM2.0 outside 1 91

# Fusarium_sp-PT_000266-RA Length: 250

# Fusarium_sp-PT_000266-RA Number of predicted TMHs: 0

# Fusarium_sp-PT_000266-RA Exp number of AAs in TMHs: 0.07655

# Fusarium_sp-PT_000266-RA Exp number, first 60 AAs: 0.01816

# Fusarium_sp-PT_000266-RA Total prob of N-in: 0.13555

Fusarium_sp-PT_000266-RA TMHMM2.0 outside 1 250

# Fusarium_sp-PT_000268-RA Length: 328

# Fusarium_sp-PT_000268-RA Number of predicted TMHs: 0

# Fusarium_sp-PT_000268-RA Exp number of AAs in TMHs: 3.31896

# Fusarium_sp-PT_000268-RA Exp number, first 60 AAs: 0.0038

# Fusarium_sp-PT_000268-RA Total prob of N-in: 0.04731

Fusarium_sp-PT_000268-RA TMHMM2.0 outside 1 328

# Fusarium_sp-PT_000269-RA Length: 819

# Fusarium_sp-PT_000269-RA Number of predicted TMHs: 0

# Fusarium_sp-PT_000269-RA Exp number of AAs in TMHs: 0.11731

# Fusarium_sp-PT_000269-RA Exp number, first 60 AAs: 0.11581

# Fusarium_sp-PT_000269-RA Total prob of N-in: 0.00560

Fusarium_sp-PT_000269-RA TMHMM2.0 outside 1 819

# Fusarium_sp-PT_000271-RA Length: 385

# Fusarium_sp-PT_000271-RA Number of predicted TMHs: 2

# Fusarium_sp-PT_000271-RA Exp number of AAs in TMHs: 38.69503

# Fusarium_sp-PT_000271-RA Exp number, first 60 AAs: 15.91407

# Fusarium_sp-PT_000271-RA Total prob of N-in: 0.72094

# Fusarium_sp-PT_000271-RA POSSIBLE N-term signal sequence

Fusarium_sp-PT_000271-RA TMHMM2.0 inside 1 6

Fusarium_sp-PT_000271-RA TMHMM2.0 TMhelix 7 29

Fusarium_sp-PT_000271-RA TMHMM2.0 outside 30 207

Fusarium_sp-PT_000271-RA TMHMM2.0 TMhelix 208 230

Fusarium_sp-PT_000271-RA TMHMM2.0 inside 231 385

# Fusarium_sp-PT_000273-RA Length: 334

# Fusarium_sp-PT_000273-RA Number of predicted TMHs: 0

# Fusarium_sp-PT_000273-RA Exp number of AAs in TMHs: 0.495820000000001

# Fusarium_sp-PT_000273-RA Exp number, first 60 AAs: 0.47691

# Fusarium_sp-PT_000273-RA Total prob of N-in: 0.01775

Fusarium_sp-PT_000273-RA TMHMM2.0 outside 1 334

# Fusarium_sp-PT_000275-RA Length: 236

# Fusarium_sp-PT_000275-RA Number of predicted TMHs: 0

# Fusarium_sp-PT_000275-RA Exp number of AAs in TMHs: 0.19717

# Fusarium_sp-PT_000275-RA Exp number, first 60 AAs: 0.19679

# Fusarium_sp-PT_000275-RA Total prob of N-in: 0.07233

Fusarium_sp-PT_000275-RA TMHMM2.0 outside 1 236

# Fusarium_sp-PT_000277-RA Length: 440

# Fusarium_sp-PT_000277-RA Number of predicted TMHs: 0

# Fusarium_sp-PT_000277-RA Exp number of AAs in TMHs: 0.0088

# Fusarium_sp-PT_000277-RA Exp number, first 60 AAs: 0

# Fusarium_sp-PT_000277-RA Total prob of N-in: 0.00322

Fusarium_sp-PT_000277-RA TMHMM2.0 outside 1 440

# Fusarium_sp-PT_000278-RA Length: 184

# Fusarium_sp-PT_000278-RA Number of predicted TMHs: 0

# Fusarium_sp-PT_000278-RA Exp number of AAs in TMHs: 0.00177

# Fusarium_sp-PT_000278-RA Exp number, first 60 AAs: 0

# Fusarium_sp-PT_000278-RA Total prob of N-in: 0.17535

Fusarium_sp-PT_000278-RA TMHMM2.0 outside 1 184

# Fusarium_sp-PT_000281-RA Length: 248

# Fusarium_sp-PT_000281-RA Number of predicted TMHs: 0

# Fusarium_sp-PT_000281-RA Exp number of AAs in TMHs: 4.94043

# Fusarium_sp-PT_000281-RA Exp number, first 60 AAs: 4.28226

# Fusarium_sp-PT_000281-RA Total prob of N-in: 0.21154

Fusarium_sp-PT_000281-RA TMHMM2.0 outside 1 248

# Fusarium_sp-PT_000283-RA Length: 546

# Fusarium_sp-PT_000283-RA Number of predicted TMHs: 0

# Fusarium_sp-PT_000283-RA Exp number of AAs in TMHs: 0.0723

# Fusarium_sp-PT_000283-RA Exp number, first 60 AAs: 0

# Fusarium_sp-PT_000283-RA Total prob of N-in: 0.00883

Fusarium_sp-PT_000283-RA TMHMM2.0 outside 1 546

# Fusarium_sp-PT_000286-RA Length: 134

# Fusarium_sp-PT_000286-RA Number of predicted TMHs: 0

# Fusarium_sp-PT_000286-RA Exp number of AAs in TMHs: 0.05571

# Fusarium_sp-PT_000286-RA Exp number, first 60 AAs: 0.05086

# Fusarium_sp-PT_000286-RA Total prob of N-in: 0.06585

Fusarium_sp-PT_000286-RA TMHMM2.0 outside 1 134

# Fusarium_sp-PT_000290-RA Length: 656

# Fusarium_sp-PT_000290-RA Number of predicted TMHs: 0

# Fusarium_sp-PT_000290-RA Exp number of AAs in TMHs: 0.00266

# Fusarium_sp-PT_000290-RA Exp number, first 60 AAs: 0

# Fusarium_sp-PT_000290-RA Total prob of N-in: 0.00011

Fusarium_sp-PT_000290-RA TMHMM2.0 outside 1 656

# Fusarium_sp-PT_000292-RA Length: 104

# Fusarium_sp-PT_000292-RA Number of predicted TMHs: 1

# Fusarium_sp-PT_000292-RA Exp number of AAs in TMHs: 18.32102

# Fusarium_sp-PT_000292-RA Exp number, first 60 AAs: 0.00233

# Fusarium_sp-PT_000292-RA Total prob of N-in: 0.94695

Fusarium_sp-PT_000292-RA TMHMM2.0 inside 1 65

Fusarium_sp-PT_000292-RA TMHMM2.0 TMhelix 66 84

Fusarium_sp-PT_000292-RA TMHMM2.0 outside 85 104

# Fusarium_sp-PT_000293-RA Length: 241

# Fusarium_sp-PT_000293-RA Number of predicted TMHs: 0

# Fusarium_sp-PT_000293-RA Exp number of AAs in TMHs: 0.00983999999999999

# Fusarium_sp-PT_000293-RA Exp number, first 60 AAs: 0

# Fusarium_sp-PT_000293-RA Total prob of N-in: 0.12357

Fusarium_sp-PT_000293-RA TMHMM2.0 outside 1 241

# Fusarium_sp-PT_000297-RA Length: 452

# Fusarium_sp-PT_000297-RA Number of predicted TMHs: 0

# Fusarium_sp-PT_000297-RA Exp number of AAs in TMHs: 0.4938

# Fusarium_sp-PT_000297-RA Exp number, first 60 AAs: 0.00105

# Fusarium_sp-PT_000297-RA Total prob of N-in: 0.00326

Fusarium_sp-PT_000297-RA TMHMM2.0 outside 1 452

# Fusarium_sp-PT_000298-RA Length: 747

# Fusarium_sp-PT_000298-RA Number of predicted TMHs: 0

# Fusarium_sp-PT_000298-RA Exp number of AAs in TMHs: 0

# Fusarium_sp-PT_000298-RA Exp number, first 60 AAs: 0

# Fusarium_sp-PT_000298-RA Total prob of N-in: 0.00002

Fusarium_sp-PT_000298-RA TMHMM2.0 outside 1 747

# Fusarium_sp-PT_000300-RA Length: 279

# Fusarium_sp-PT_000300-RA Number of predicted TMHs: 0

# Fusarium_sp-PT_000300-RA Exp number of AAs in TMHs: 0.23197

# Fusarium_sp-PT_000300-RA Exp number, first 60 AAs: 0.14627

# Fusarium_sp-PT_000300-RA Total prob of N-in: 0.01208

Fusarium_sp-PT_000300-RA TMHMM2.0 outside 1 279

# Fusarium_sp-PT_000301-RA Length: 155

# Fusarium_sp-PT_000301-RA Number of predicted TMHs: 0

# Fusarium_sp-PT_000301-RA Exp number of AAs in TMHs: 0

# Fusarium_sp-PT_000301-RA Exp number, first 60 AAs: 0

# Fusarium_sp-PT_000301-RA Total prob of N-in: 0.40051

Fusarium_sp-PT_000301-RA TMHMM2.0 outside 1 155

# Fusarium_sp-PT_000305-RA Length: 123

# Fusarium_sp-PT_000305-RA Number of predicted TMHs: 0

# Fusarium_sp-PT_000305-RA Exp number of AAs in TMHs: 0.03952

# Fusarium_sp-PT_000305-RA Exp number, first 60 AAs: 0.03952

# Fusarium_sp-PT_000305-RA Total prob of N-in: 0.05884

Fusarium_sp-PT_000305-RA TMHMM2.0 outside 1 123

# Fusarium_sp-PT_000267-RA Length: 279

# Fusarium_sp-PT_000267-RA Number of predicted TMHs: 0

# Fusarium_sp-PT_000267-RA Exp number of AAs in TMHs: 0.01256

# Fusarium_sp-PT_000267-RA Exp number, first 60 AAs: 0.000650000000000001

# Fusarium_sp-PT_000267-RA Total prob of N-in: 0.02508

Fusarium_sp-PT_000267-RA TMHMM2.0 outside 1 279

# Fusarium_sp-PT_000270-RA Length: 920

# Fusarium_sp-PT_000270-RA Number of predicted TMHs: 9

# Fusarium_sp-PT_000270-RA Exp number of AAs in TMHs: 194.3603

# Fusarium_sp-PT_000270-RA Exp number, first 60 AAs: 0

# Fusarium_sp-PT_000270-RA Total prob of N-in: 0.02866

Fusarium_sp-PT_000270-RA TMHMM2.0 outside 1 460

Fusarium_sp-PT_000270-RA TMHMM2.0 TMhelix 461 483

Fusarium_sp-PT_000270-RA TMHMM2.0 inside 484 562

Fusarium_sp-PT_000270-RA TMHMM2.0 TMhelix 563 581

Fusarium_sp-PT_000270-RA TMHMM2.0 outside 582 595

Fusarium_sp-PT_000270-RA TMHMM2.0 TMhelix 596 618

Fusarium_sp-PT_000270-RA TMHMM2.0 inside 619 637

Fusarium_sp-PT_000270-RA TMHMM2.0 TMhelix 638 660

Fusarium_sp-PT_000270-RA TMHMM2.0 outside 661 674

Fusarium_sp-PT_000270-RA TMHMM2.0 TMhelix 675 697

Fusarium_sp-PT_000270-RA TMHMM2.0 inside 698 715

Fusarium_sp-PT_000270-RA TMHMM2.0 TMhelix 716 738

Fusarium_sp-PT_000270-RA TMHMM2.0 outside 739 747

Fusarium_sp-PT_000270-RA TMHMM2.0 TMhelix 748 767

Fusarium_sp-PT_000270-RA TMHMM2.0 inside 768 846

Fusarium_sp-PT_000270-RA TMHMM2.0 TMhelix 847 869

Fusarium_sp-PT_000270-RA TMHMM2.0 outside 870 891

Fusarium_sp-PT_000270-RA TMHMM2.0 TMhelix 892 914

Fusarium_sp-PT_000270-RA TMHMM2.0 inside 915 920

# Fusarium_sp-PT_000274-RA Length: 126

# Fusarium_sp-PT_000274-RA Number of predicted TMHs: 0

# Fusarium_sp-PT_000274-RA Exp number of AAs in TMHs: 0

# Fusarium_sp-PT_000274-RA Exp number, first 60 AAs: 0

# Fusarium_sp-PT_000274-RA Total prob of N-in: 0.04708

Fusarium_sp-PT_000274-RA TMHMM2.0 outside 1 126

# Fusarium_sp-PT_000276-RA Length: 372

# Fusarium_sp-PT_000276-RA Number of predicted TMHs: 0

# Fusarium_sp-PT_000276-RA Exp number of AAs in TMHs: 0.0463200000000001

# Fusarium_sp-PT_000276-RA Exp number, first 60 AAs: 0

# Fusarium_sp-PT_000276-RA Total prob of N-in: 0.02228

Fusarium_sp-PT_000276-RA TMHMM2.0 outside 1 372

# Fusarium_sp-PT_000279-RA Length: 368

# Fusarium_sp-PT_000279-RA Number of predicted TMHs: 0

# Fusarium_sp-PT_000279-RA Exp number of AAs in TMHs: 0

# Fusarium_sp-PT_000279-RA Exp number, first 60 AAs: 0

# Fusarium_sp-PT_000279-RA Total prob of N-in: 0.00142

Fusarium_sp-PT_000279-RA TMHMM2.0 outside 1 368

# Fusarium_sp-PT_000280-RA Length: 200

# Fusarium_sp-PT_000280-RA Number of predicted TMHs: 0

# Fusarium_sp-PT_000280-RA Exp number of AAs in TMHs: 0.06727

# Fusarium_sp-PT_000280-RA Exp number, first 60 AAs: 0.02319

# Fusarium_sp-PT_000280-RA Total prob of N-in: 0.11356

Fusarium_sp-PT_000280-RA TMHMM2.0 outside 1 200

# Fusarium_sp-PT_000282-RA Length: 476

# Fusarium_sp-PT_000282-RA Number of predicted TMHs: 9

# Fusarium_sp-PT_000282-RA Exp number of AAs in TMHs: 235.18955

# Fusarium_sp-PT_000282-RA Exp number, first 60 AAs: 18.12678

# Fusarium_sp-PT_000282-RA Total prob of N-in: 0.53648

# Fusarium_sp-PT_000282-RA POSSIBLE N-term signal sequence

Fusarium_sp-PT_000282-RA TMHMM2.0 outside 1 23

Fusarium_sp-PT_000282-RA TMHMM2.0 TMhelix 24 41

Fusarium_sp-PT_000282-RA TMHMM2.0 inside 42 153

Fusarium_sp-PT_000282-RA TMHMM2.0 TMhelix 154 176

Fusarium_sp-PT_000282-RA TMHMM2.0 outside 177 190

Fusarium_sp-PT_000282-RA TMHMM2.0 TMhelix 191 210

Fusarium_sp-PT_000282-RA TMHMM2.0 inside 211 250

Fusarium_sp-PT_000282-RA TMHMM2.0 TMhelix 251 268

Fusarium_sp-PT_000282-RA TMHMM2.0 outside 269 299

Fusarium_sp-PT_000282-RA TMHMM2.0 TMhelix 300 322

Fusarium_sp-PT_000282-RA TMHMM2.0 inside 323 326

Fusarium_sp-PT_000282-RA TMHMM2.0 TMhelix 327 346

Fusarium_sp-PT_000282-RA TMHMM2.0 outside 347 355

Fusarium_sp-PT_000282-RA TMHMM2.0 TMhelix 356 378

Fusarium_sp-PT_000282-RA TMHMM2.0 inside 379 389

Fusarium_sp-PT_000282-RA TMHMM2.0 TMhelix 390 412

Fusarium_sp-PT_000282-RA TMHMM2.0 outside 413 421

Fusarium_sp-PT_000282-RA TMHMM2.0 TMhelix 422 444

Fusarium_sp-PT_000282-RA TMHMM2.0 inside 445 476

# Fusarium_sp-PT_000284-RA Length: 418

# Fusarium_sp-PT_000284-RA Number of predicted TMHs: 0

# Fusarium_sp-PT_000284-RA Exp number of AAs in TMHs: 0.03336

# Fusarium_sp-PT_000284-RA Exp number, first 60 AAs: 0.00065

# Fusarium_sp-PT_000284-RA Total prob of N-in: 0.00564

Fusarium_sp-PT_000284-RA TMHMM2.0 outside 1 418

# Fusarium_sp-PT_000285-RA Length: 164

# Fusarium_sp-PT_000285-RA Number of predicted TMHs: 0

# Fusarium_sp-PT_000285-RA Exp number of AAs in TMHs: 0.07807

# Fusarium_sp-PT_000285-RA Exp number, first 60 AAs: 0.07807

# Fusarium_sp-PT_000285-RA Total prob of N-in: 0.03555

Fusarium_sp-PT_000285-RA TMHMM2.0 outside 1 164

# Fusarium_sp-PT_000287-RA Length: 512

# Fusarium_sp-PT_000287-RA Number of predicted TMHs: 11

# Fusarium_sp-PT_000287-RA Exp number of AAs in TMHs: 231.26025

# Fusarium_sp-PT_000287-RA Exp number, first 60 AAs: 0.32963

# Fusarium_sp-PT_000287-RA Total prob of N-in: 0.25629

Fusarium_sp-PT_000287-RA TMHMM2.0 outside 1 87

Fusarium_sp-PT_000287-RA TMHMM2.0 TMhelix 88 110

Fusarium_sp-PT_000287-RA TMHMM2.0 inside 111 114

Fusarium_sp-PT_000287-RA TMHMM2.0 TMhelix 115 137

Fusarium_sp-PT_000287-RA TMHMM2.0 outside 138 146

Fusarium_sp-PT_000287-RA TMHMM2.0 TMhelix 147 169

Fusarium_sp-PT_000287-RA TMHMM2.0 inside 170 175

Fusarium_sp-PT_000287-RA TMHMM2.0 TMhelix 176 198

Fusarium_sp-PT_000287-RA TMHMM2.0 outside 199 207

Fusarium_sp-PT_000287-RA TMHMM2.0 TMhelix 208 230

Fusarium_sp-PT_000287-RA TMHMM2.0 inside 231 278

Fusarium_sp-PT_000287-RA TMHMM2.0 TMhelix 279 301

Fusarium_sp-PT_000287-RA TMHMM2.0 outside 302 310

Fusarium_sp-PT_000287-RA TMHMM2.0 TMhelix 311 333

Fusarium_sp-PT_000287-RA TMHMM2.0 inside 334 339

Fusarium_sp-PT_000287-RA TMHMM2.0 TMhelix 340 362

Fusarium_sp-PT_000287-RA TMHMM2.0 outside 363 376

Fusarium_sp-PT_000287-RA TMHMM2.0 TMhelix 377 399

Fusarium_sp-PT_000287-RA TMHMM2.0 inside 400 405

Fusarium_sp-PT_000287-RA TMHMM2.0 TMhelix 406 428

Fusarium_sp-PT_000287-RA TMHMM2.0 outside 429 442

Fusarium_sp-PT_000287-RA TMHMM2.0 TMhelix 443 460

Fusarium_sp-PT_000287-RA TMHMM2.0 inside 461 512

# Fusarium_sp-PT_000288-RA Length: 232

# Fusarium_sp-PT_000288-RA Number of predicted TMHs: 0

# Fusarium_sp-PT_000288-RA Exp number of AAs in TMHs: 0.0920999999999999

# Fusarium_sp-PT_000288-RA Exp number, first 60 AAs: 0.08599

# Fusarium_sp-PT_000288-RA Total prob of N-in: 0.21294

Fusarium_sp-PT_000288-RA TMHMM2.0 outside 1 232

# Fusarium_sp-PT_000289-RA Length: 445

# Fusarium_sp-PT_000289-RA Number of predicted TMHs: 1

# Fusarium_sp-PT_000289-RA Exp number of AAs in TMHs: 22.96718

# Fusarium_sp-PT_000289-RA Exp number, first 60 AAs: 0.10437

# Fusarium_sp-PT_000289-RA Total prob of N-in: 0.05269

Fusarium_sp-PT_000289-RA TMHMM2.0 outside 1 275

Fusarium_sp-PT_000289-RA TMHMM2.0 TMhelix 276 298

Fusarium_sp-PT_000289-RA TMHMM2.0 inside 299 445

# Fusarium_sp-PT_000291-RA Length: 280

# Fusarium_sp-PT_000291-RA Number of predicted TMHs: 0

# Fusarium_sp-PT_000291-RA Exp number of AAs in TMHs: 5.97707999999999

# Fusarium_sp-PT_000291-RA Exp number, first 60 AAs: 5.97551

# Fusarium_sp-PT_000291-RA Total prob of N-in: 0.29490

Fusarium_sp-PT_000291-RA TMHMM2.0 outside 1 280

# Fusarium_sp-PT_000294-RA Length: 150

# Fusarium_sp-PT_000294-RA Number of predicted TMHs: 4

# Fusarium_sp-PT_000294-RA Exp number of AAs in TMHs: 81.57703

# Fusarium_sp-PT_000294-RA Exp number, first 60 AAs: 22.86783

# Fusarium_sp-PT_000294-RA Total prob of N-in: 0.08824

# Fusarium_sp-PT_000294-RA POSSIBLE N-term signal sequence

Fusarium_sp-PT_000294-RA TMHMM2.0 outside 1 14

Fusarium_sp-PT_000294-RA TMHMM2.0 TMhelix 15 37

Fusarium_sp-PT_000294-RA TMHMM2.0 inside 38 57

Fusarium_sp-PT_000294-RA TMHMM2.0 TMhelix 58 80

Fusarium_sp-PT_000294-RA TMHMM2.0 outside 81 89

Fusarium_sp-PT_000294-RA TMHMM2.0 TMhelix 90 112

Fusarium_sp-PT_000294-RA TMHMM2.0 inside 113 120

Fusarium_sp-PT_000294-RA TMHMM2.0 TMhelix 121 143

Fusarium_sp-PT_000294-RA TMHMM2.0 outside 144 150

# Fusarium_sp-PT_000295-RA Length: 491

# Fusarium_sp-PT_000295-RA Number of predicted TMHs: 0

# Fusarium_sp-PT_000295-RA Exp number of AAs in TMHs: 2.20324

# Fusarium_sp-PT_000295-RA Exp number, first 60 AAs: 0

# Fusarium_sp-PT_000295-RA Total prob of N-in: 0.10277

Fusarium_sp-PT_000295-RA TMHMM2.0 outside 1 491

# Fusarium_sp-PT_000296-RA Length: 456

# Fusarium_sp-PT_000296-RA Number of predicted TMHs: 0

# Fusarium_sp-PT_000296-RA Exp number of AAs in TMHs: 0.00063

# Fusarium_sp-PT_000296-RA Exp number, first 60 AAs: 5e-05

# Fusarium_sp-PT_000296-RA Total prob of N-in: 0.01214

Fusarium_sp-PT_000296-RA TMHMM2.0 outside 1 456

# Fusarium_sp-PT_000299-RA Length: 226

# Fusarium_sp-PT_000299-RA Number of predicted TMHs: 0

# Fusarium_sp-PT_000299-RA Exp number of AAs in TMHs: 0.01576

# Fusarium_sp-PT_000299-RA Exp number, first 60 AAs: 0.01543

# Fusarium_sp-PT_000299-RA Total prob of N-in: 0.11611

Fusarium_sp-PT_000299-RA TMHMM2.0 outside 1 226

# Fusarium_sp-PT_000302-RA Length: 190

# Fusarium_sp-PT_000302-RA Number of predicted TMHs: 0

# Fusarium_sp-PT_000302-RA Exp number of AAs in TMHs: 0.00451999999999999

# Fusarium_sp-PT_000302-RA Exp number, first 60 AAs: 0.00022

# Fusarium_sp-PT_000302-RA Total prob of N-in: 0.20398

Fusarium_sp-PT_000302-RA TMHMM2.0 outside 1 190

# Fusarium_sp-PT_000303-RA Length: 237

# Fusarium_sp-PT_000303-RA Number of predicted TMHs: 0

# Fusarium_sp-PT_000303-RA Exp number of AAs in TMHs: 0.00268

# Fusarium_sp-PT_000303-RA Exp number, first 60 AAs: 0

# Fusarium_sp-PT_000303-RA Total prob of N-in: 0.13274

Fusarium_sp-PT_000303-RA TMHMM2.0 outside 1 237

# Fusarium_sp-PT_000304-RA Length: 413

# Fusarium_sp-PT_000304-RA Number of predicted TMHs: 3

# Fusarium_sp-PT_000304-RA Exp number of AAs in TMHs: 92.6453400000002

# Fusarium_sp-PT_000304-RA Exp number, first 60 AAs: 4.78849

# Fusarium_sp-PT_000304-RA Total prob of N-in: 0.45375

Fusarium_sp-PT_000304-RA TMHMM2.0 inside 1 116

Fusarium_sp-PT_000304-RA TMHMM2.0 TMhelix 117 139

Fusarium_sp-PT_000304-RA TMHMM2.0 outside 140 158

Fusarium_sp-PT_000304-RA TMHMM2.0 TMhelix 159 178

Fusarium_sp-PT_000304-RA TMHMM2.0 inside 179 233

Fusarium_sp-PT_000304-RA TMHMM2.0 TMhelix 234 256

Fusarium_sp-PT_000304-RA TMHMM2.0 outside 257 413

# Fusarium_sp-PT_000306-RA Length: 928

# Fusarium_sp-PT_000306-RA Number of predicted TMHs: 0

# Fusarium_sp-PT_000306-RA Exp number of AAs in TMHs: 0.012

# Fusarium_sp-PT_000306-RA Exp number, first 60 AAs: 0

# Fusarium_sp-PT_000306-RA Total prob of N-in: 0.00029

Fusarium_sp-PT_000306-RA TMHMM2.0 outside 1 928

# Fusarium_sp-PT_000308-RA Length: 731

# Fusarium_sp-PT_000308-RA Number of predicted TMHs: 0

# Fusarium_sp-PT_000308-RA Exp number of AAs in TMHs: 5.57246999999999

# Fusarium_sp-PT_000308-RA Exp number, first 60 AAs: 0.00941

# Fusarium_sp-PT_000308-RA Total prob of N-in: 0.25085

Fusarium_sp-PT_000308-RA TMHMM2.0 outside 1 731

# Fusarium_sp-PT_000312-RA Length: 138

# Fusarium_sp-PT_000312-RA Number of predicted TMHs: 0

# Fusarium_sp-PT_000312-RA Exp number of AAs in TMHs: 0.05676

# Fusarium_sp-PT_000312-RA Exp number, first 60 AAs: 0

# Fusarium_sp-PT_000312-RA Total prob of N-in: 0.27822

Fusarium_sp-PT_000312-RA TMHMM2.0 outside 1 138

# Fusarium_sp-PT_000314-RA Length: 538

# Fusarium_sp-PT_000314-RA Number of predicted TMHs: 0

# Fusarium_sp-PT_000314-RA Exp number of AAs in TMHs: 0.566560000000001

# Fusarium_sp-PT_000314-RA Exp number, first 60 AAs: 0.00079

# Fusarium_sp-PT_000314-RA Total prob of N-in: 0.01228

Fusarium_sp-PT_000314-RA TMHMM2.0 outside 1 538

# Fusarium_sp-PT_000315-RA Length: 1341

# Fusarium_sp-PT_000315-RA Number of predicted TMHs: 0

# Fusarium_sp-PT_000315-RA Exp number of AAs in TMHs: 0.06753

# Fusarium_sp-PT_000315-RA Exp number, first 60 AAs: 0

# Fusarium_sp-PT_000315-RA Total prob of N-in: 0.00045

Fusarium_sp-PT_000315-RA TMHMM2.0 outside 1 1341

# Fusarium_sp-PT_000317-RA Length: 204

# Fusarium_sp-PT_000317-RA Number of predicted TMHs: 0

# Fusarium_sp-PT_000317-RA Exp number of AAs in TMHs: 0.00098

# Fusarium_sp-PT_000317-RA Exp number, first 60 AAs: 0.00037

# Fusarium_sp-PT_000317-RA Total prob of N-in: 0.13299

Fusarium_sp-PT_000317-RA TMHMM2.0 outside 1 204

# Fusarium_sp-PT_000323-RA Length: 139

# Fusarium_sp-PT_000323-RA Number of predicted TMHs: 0

# Fusarium_sp-PT_000323-RA Exp number of AAs in TMHs: 2.09563

# Fusarium_sp-PT_000323-RA Exp number, first 60 AAs: 1.94938

# Fusarium_sp-PT_000323-RA Total prob of N-in: 0.10607

Fusarium_sp-PT_000323-RA TMHMM2.0 outside 1 139

# Fusarium_sp-PT_000325-RA Length: 413

# Fusarium_sp-PT_000325-RA Number of predicted TMHs: 0

# Fusarium_sp-PT_000325-RA Exp number of AAs in TMHs: 0.00751

# Fusarium_sp-PT_000325-RA Exp number, first 60 AAs: 0

# Fusarium_sp-PT_000325-RA Total prob of N-in: 0.00268

Fusarium_sp-PT_000325-RA TMHMM2.0 outside 1 413

# Fusarium_sp-PT_000328-RA Length: 791

# Fusarium_sp-PT_000328-RA Number of predicted TMHs: 0

# Fusarium_sp-PT_000328-RA Exp number of AAs in TMHs: 0.03328

# Fusarium_sp-PT_000328-RA Exp number, first 60 AAs: 0.00146

# Fusarium_sp-PT_000328-RA Total prob of N-in: 0.00064

Fusarium_sp-PT_000328-RA TMHMM2.0 outside 1 791

# Fusarium_sp-PT_000330-RA Length: 183

# Fusarium_sp-PT_000330-RA Number of predicted TMHs: 0

# Fusarium_sp-PT_000330-RA Exp number of AAs in TMHs: 0.00911

# Fusarium_sp-PT_000330-RA Exp number, first 60 AAs: 0

# Fusarium_sp-PT_000330-RA Total prob of N-in: 0.27577

Fusarium_sp-PT_000330-RA TMHMM2.0 outside 1 183

# Fusarium_sp-PT_000331-RA Length: 301

# Fusarium_sp-PT_000331-RA Number of predicted TMHs: 0

# Fusarium_sp-PT_000331-RA Exp number of AAs in TMHs: 2.13501

# Fusarium_sp-PT_000331-RA Exp number, first 60 AAs: 1.9677

# Fusarium_sp-PT_000331-RA Total prob of N-in: 0.05442

Fusarium_sp-PT_000331-RA TMHMM2.0 outside 1 301

# Fusarium_sp-PT_000335-RA Length: 86

# Fusarium_sp-PT_000335-RA Number of predicted TMHs: 1

# Fusarium_sp-PT_000335-RA Exp number of AAs in TMHs: 21.13759

# Fusarium_sp-PT_000335-RA Exp number, first 60 AAs: 1.10483

# Fusarium_sp-PT_000335-RA Total prob of N-in: 0.93819

Fusarium_sp-PT_000335-RA TMHMM2.0 inside 1 59

Fusarium_sp-PT_000335-RA TMHMM2.0 TMhelix 60 82

Fusarium_sp-PT_000335-RA TMHMM2.0 outside 83 86

# Fusarium_sp-PT_000337-RA Length: 388

# Fusarium_sp-PT_000337-RA Number of predicted TMHs: 0

# Fusarium_sp-PT_000337-RA Exp number of AAs in TMHs: 0.01358

# Fusarium_sp-PT_000337-RA Exp number, first 60 AAs: 0.00197

# Fusarium_sp-PT_000337-RA Total prob of N-in: 0.00381

Fusarium_sp-PT_000337-RA TMHMM2.0 outside 1 388

# Fusarium_sp-PT_000338-RA Length: 1087

# Fusarium_sp-PT_000338-RA Number of predicted TMHs: 0

# Fusarium_sp-PT_000338-RA Exp number of AAs in TMHs: 0.00805

# Fusarium_sp-PT_000338-RA Exp number, first 60 AAs: 0.0002

# Fusarium_sp-PT_000338-RA Total prob of N-in: 0.00001

Fusarium_sp-PT_000338-RA TMHMM2.0 outside 1 1087

# Fusarium_sp-PT_000339-RA Length: 460

# Fusarium_sp-PT_000339-RA Number of predicted TMHs: 0

# Fusarium_sp-PT_000339-RA Exp number of AAs in TMHs: 0.0923300000000001

# Fusarium_sp-PT_000339-RA Exp number, first 60 AAs: 0.07293

# Fusarium_sp-PT_000339-RA Total prob of N-in: 0.01378

Fusarium_sp-PT_000339-RA TMHMM2.0 outside 1 460

# Fusarium_sp-PT_000341-RA Length: 260

# Fusarium_sp-PT_000341-RA Number of predicted TMHs: 0

# Fusarium_sp-PT_000341-RA Exp number of AAs in TMHs: 0.00258

# Fusarium_sp-PT_000341-RA Exp number, first 60 AAs: 0

# Fusarium_sp-PT_000341-RA Total prob of N-in: 0.01964

Fusarium_sp-PT_000341-RA TMHMM2.0 outside 1 260

# Fusarium_sp-PT_000307-RA Length: 1234

# Fusarium_sp-PT_000307-RA Number of predicted TMHs: 0

# Fusarium_sp-PT_000307-RA Exp number of AAs in TMHs: 0.00508

# Fusarium_sp-PT_000307-RA Exp number, first 60 AAs: 0

# Fusarium_sp-PT_000307-RA Total prob of N-in: 0.00024

Fusarium_sp-PT_000307-RA TMHMM2.0 outside 1 1234

# Fusarium_sp-PT_000309-RA Length: 120

# Fusarium_sp-PT_000309-RA Number of predicted TMHs: 2

# Fusarium_sp-PT_000309-RA Exp number of AAs in TMHs: 42.88416

# Fusarium_sp-PT_000309-RA Exp number, first 60 AAs: 7.71856

# Fusarium_sp-PT_000309-RA Total prob of N-in: 0.32535

Fusarium_sp-PT_000309-RA TMHMM2.0 outside 1 52

Fusarium_sp-PT_000309-RA TMHMM2.0 TMhelix 53 75

Fusarium_sp-PT_000309-RA TMHMM2.0 inside 76 86

Fusarium_sp-PT_000309-RA TMHMM2.0 TMhelix 87 109

Fusarium_sp-PT_000309-RA TMHMM2.0 outside 110 120

# Fusarium_sp-PT_000310-RA Length: 678

# Fusarium_sp-PT_000310-RA Number of predicted TMHs: 0

# Fusarium_sp-PT_000310-RA Exp number of AAs in TMHs: 5.85491999999998

# Fusarium_sp-PT_000310-RA Exp number, first 60 AAs: 5.21602

# Fusarium_sp-PT_000310-RA Total prob of N-in: 0.26947

Fusarium_sp-PT_000310-RA TMHMM2.0 outside 1 678

# Fusarium_sp-PT_000313-RA Length: 514

# Fusarium_sp-PT_000313-RA Number of predicted TMHs: 0

# Fusarium_sp-PT_000313-RA Exp number of AAs in TMHs: 0.13937

# Fusarium_sp-PT_000313-RA Exp number, first 60 AAs: 0

# Fusarium_sp-PT_000313-RA Total prob of N-in: 0.00140

Fusarium_sp-PT_000313-RA TMHMM2.0 outside 1 514

# Fusarium_sp-PT_000316-RA Length: 322

# Fusarium_sp-PT_000316-RA Number of predicted TMHs: 0

# Fusarium_sp-PT_000316-RA Exp number of AAs in TMHs: 0.17153

# Fusarium_sp-PT_000316-RA Exp number, first 60 AAs: 0.17054

# Fusarium_sp-PT_000316-RA Total prob of N-in: 0.06347

Fusarium_sp-PT_000316-RA TMHMM2.0 outside 1 322

# Fusarium_sp-PT_000318-RA Length: 361

# Fusarium_sp-PT_000318-RA Number of predicted TMHs: 0

# Fusarium_sp-PT_000318-RA Exp number of AAs in TMHs: 0.04409

# Fusarium_sp-PT_000318-RA Exp number, first 60 AAs: 0.00517

# Fusarium_sp-PT_000318-RA Total prob of N-in: 0.00651

Fusarium_sp-PT_000318-RA TMHMM2.0 outside 1 361

# Fusarium_sp-PT_000319-RA Length: 344

# Fusarium_sp-PT_000319-RA Number of predicted TMHs: 2

# Fusarium_sp-PT_000319-RA Exp number of AAs in TMHs: 42.94957

# Fusarium_sp-PT_000319-RA Exp number, first 60 AAs: 29.45556

# Fusarium_sp-PT_000319-RA Total prob of N-in: 0.40563

# Fusarium_sp-PT_000319-RA POSSIBLE N-term signal sequence

Fusarium_sp-PT_000319-RA TMHMM2.0 outside 1 9

Fusarium_sp-PT_000319-RA TMHMM2.0 TMhelix 10 32

Fusarium_sp-PT_000319-RA TMHMM2.0 inside 33 52

Fusarium_sp-PT_000319-RA TMHMM2.0 TMhelix 53 75

Fusarium_sp-PT_000319-RA TMHMM2.0 outside 76 344

# Fusarium_sp-PT_000321-RA Length: 327

# Fusarium_sp-PT_000321-RA Number of predicted TMHs: 0

# Fusarium_sp-PT_000321-RA Exp number of AAs in TMHs: 0.05675

# Fusarium_sp-PT_000321-RA Exp number, first 60 AAs: 0.00314

# Fusarium_sp-PT_000321-RA Total prob of N-in: 0.00754

Fusarium_sp-PT_000321-RA TMHMM2.0 outside 1 327

# Fusarium_sp-PT_000322-RA Length: 356

# Fusarium_sp-PT_000322-RA Number of predicted TMHs: 0

# Fusarium_sp-PT_000322-RA Exp number of AAs in TMHs: 1.26584

# Fusarium_sp-PT_000322-RA Exp number, first 60 AAs: 0.88551

# Fusarium_sp-PT_000322-RA Total prob of N-in: 0.04168

Fusarium_sp-PT_000322-RA TMHMM2.0 outside 1 356

# Fusarium_sp-PT_000324-RA Length: 289

# Fusarium_sp-PT_000324-RA Number of predicted TMHs: 0

# Fusarium_sp-PT_000324-RA Exp number of AAs in TMHs: 0.000980000000000001

# Fusarium_sp-PT_000324-RA Exp number, first 60 AAs: 0.00076

# Fusarium_sp-PT_000324-RA Total prob of N-in: 0.05436

Fusarium_sp-PT_000324-RA TMHMM2.0 outside 1 289

# Fusarium_sp-PT_000326-RA Length: 555

# Fusarium_sp-PT_000326-RA Number of predicted TMHs: 10

# Fusarium_sp-PT_000326-RA Exp number of AAs in TMHs: 211.36891

# Fusarium_sp-PT_000326-RA Exp number, first 60 AAs: 0

# Fusarium_sp-PT_000326-RA Total prob of N-in: 0.99798

Fusarium_sp-PT_000326-RA TMHMM2.0 inside 1 181

Fusarium_sp-PT_000326-RA TMHMM2.0 TMhelix 182 204

Fusarium_sp-PT_000326-RA TMHMM2.0 outside 205 213

Fusarium_sp-PT_000326-RA TMHMM2.0 TMhelix 214 236

Fusarium_sp-PT_000326-RA TMHMM2.0 inside 237 283

Fusarium_sp-PT_000326-RA TMHMM2.0 TMhelix 284 306

Fusarium_sp-PT_000326-RA TMHMM2.0 outside 307 315

Fusarium_sp-PT_000326-RA TMHMM2.0 TMhelix 316 335

Fusarium_sp-PT_000326-RA TMHMM2.0 inside 336 341

Fusarium_sp-PT_000326-RA TMHMM2.0 TMhelix 342 359

Fusarium_sp-PT_000326-RA TMHMM2.0 outside 360 373

Fusarium_sp-PT_000326-RA TMHMM2.0 TMhelix 374 393

Fusarium_sp-PT_000326-RA TMHMM2.0 inside 394 412

Fusarium_sp-PT_000326-RA TMHMM2.0 TMhelix 413 435

Fusarium_sp-PT_000326-RA TMHMM2.0 outside 436 449

Fusarium_sp-PT_000326-RA TMHMM2.0 TMhelix 450 472

Fusarium_sp-PT_000326-RA TMHMM2.0 inside 473 476

Fusarium_sp-PT_000326-RA TMHMM2.0 TMhelix 477 499

Fusarium_sp-PT_000326-RA TMHMM2.0 outside 500 502

Fusarium_sp-PT_000326-RA TMHMM2.0 TMhelix 503 525

Fusarium_sp-PT_000326-RA TMHMM2.0 inside 526 555

# Fusarium_sp-PT_000327-RA Length: 353

# Fusarium_sp-PT_000327-RA Number of predicted TMHs: 0

# Fusarium_sp-PT_000327-RA Exp number of AAs in TMHs: 0.01815

# Fusarium_sp-PT_000327-RA Exp number, first 60 AAs: 0.00667

# Fusarium_sp-PT_000327-RA Total prob of N-in: 0.01080

Fusarium_sp-PT_000327-RA TMHMM2.0 outside 1 353

# Fusarium_sp-PT_000329-RA Length: 221

# Fusarium_sp-PT_000329-RA Number of predicted TMHs: 0

# Fusarium_sp-PT_000329-RA Exp number of AAs in TMHs: 0.0034

# Fusarium_sp-PT_000329-RA Exp number, first 60 AAs: 0.00058

# Fusarium_sp-PT_000329-RA Total prob of N-in: 0.01709

Fusarium_sp-PT_000329-RA TMHMM2.0 outside 1 221

# Fusarium_sp-PT_000332-RA Length: 849

# Fusarium_sp-PT_000332-RA Number of predicted TMHs: 0

# Fusarium_sp-PT_000332-RA Exp number of AAs in TMHs: 0.000940000000000001

# Fusarium_sp-PT_000332-RA Exp number, first 60 AAs: 0

# Fusarium_sp-PT_000332-RA Total prob of N-in: 0.00004

Fusarium_sp-PT_000332-RA TMHMM2.0 outside 1 849

# Fusarium_sp-PT_000333-RA Length: 52

# Fusarium_sp-PT_000333-RA Number of predicted TMHs: 1

# Fusarium_sp-PT_000333-RA Exp number of AAs in TMHs: 25.89506

# Fusarium_sp-PT_000333-RA Exp number, first 60 AAs: 25.89506

# Fusarium_sp-PT_000333-RA Total prob of N-in: 0.51203

# Fusarium_sp-PT_000333-RA POSSIBLE N-term signal sequence

Fusarium_sp-PT_000333-RA TMHMM2.0 outside 1 27

Fusarium_sp-PT_000333-RA TMHMM2.0 TMhelix 28 50

Fusarium_sp-PT_000333-RA TMHMM2.0 inside 51 52

# Fusarium_sp-PT_000334-RA Length: 242

# Fusarium_sp-PT_000334-RA Number of predicted TMHs: 4

# Fusarium_sp-PT_000334-RA Exp number of AAs in TMHs: 113.85169

# Fusarium_sp-PT_000334-RA Exp number, first 60 AAs: 12.61686

# Fusarium_sp-PT_000334-RA Total prob of N-in: 0.54303

# Fusarium_sp-PT_000334-RA POSSIBLE N-term signal sequence

Fusarium_sp-PT_000334-RA TMHMM2.0 inside 1 100

Fusarium_sp-PT_000334-RA TMHMM2.0 TMhelix 101 123

Fusarium_sp-PT_000334-RA TMHMM2.0 outside 124 132

Fusarium_sp-PT_000334-RA TMHMM2.0 TMhelix 133 155

Fusarium_sp-PT_000334-RA TMHMM2.0 inside 156 175

Fusarium_sp-PT_000334-RA TMHMM2.0 TMhelix 176 198

Fusarium_sp-PT_000334-RA TMHMM2.0 outside 199 207

Fusarium_sp-PT_000334-RA TMHMM2.0 TMhelix 208 230

Fusarium_sp-PT_000334-RA TMHMM2.0 inside 231 242

# Fusarium_sp-PT_000336-RA Length: 695

# Fusarium_sp-PT_000336-RA Number of predicted TMHs: 0

# Fusarium_sp-PT_000336-RA Exp number of AAs in TMHs: 0.0984

# Fusarium_sp-PT_000336-RA Exp number, first 60 AAs: 0.00116

# Fusarium_sp-PT_000336-RA Total prob of N-in: 0.00416

Fusarium_sp-PT_000336-RA TMHMM2.0 outside 1 695

# Fusarium_sp-PT_000340-RA Length: 568

# Fusarium_sp-PT_000340-RA Number of predicted TMHs: 0

# Fusarium_sp-PT_000340-RA Exp number of AAs in TMHs: 22.07412

# Fusarium_sp-PT_000340-RA Exp number, first 60 AAs: 0.01079

# Fusarium_sp-PT_000340-RA Total prob of N-in: 0.53171

Fusarium_sp-PT_000340-RA TMHMM2.0 outside 1 568

# Fusarium_sp-PT_000342-RA Length: 568

# Fusarium_sp-PT_000342-RA Number of predicted TMHs: 0

# Fusarium_sp-PT_000342-RA Exp number of AAs in TMHs: 0.645919999999999

# Fusarium_sp-PT_000342-RA Exp number, first 60 AAs: 5e-05

# Fusarium_sp-PT_000342-RA Total prob of N-in: 0.00149

Fusarium_sp-PT_000342-RA TMHMM2.0 outside 1 568

# Fusarium_sp-PT_000311-RA Length: 359

# Fusarium_sp-PT_000311-RA Number of predicted TMHs: 6

# Fusarium_sp-PT_000311-RA Exp number of AAs in TMHs: 127.40634

# Fusarium_sp-PT_000311-RA Exp number, first 60 AAs: 20.20718

# Fusarium_sp-PT_000311-RA Total prob of N-in: 0.86352

# Fusarium_sp-PT_000311-RA POSSIBLE N-term signal sequence

Fusarium_sp-PT_000311-RA TMHMM2.0 inside 1 8

Fusarium_sp-PT_000311-RA TMHMM2.0 TMhelix 9 31

Fusarium_sp-PT_000311-RA TMHMM2.0 outside 32 93

Fusarium_sp-PT_000311-RA TMHMM2.0 TMhelix 94 116

Fusarium_sp-PT_000311-RA TMHMM2.0 inside 117 168

Fusarium_sp-PT_000311-RA TMHMM2.0 TMhelix 169 191

Fusarium_sp-PT_000311-RA TMHMM2.0 outside 192 210

Fusarium_sp-PT_000311-RA TMHMM2.0 TMhelix 211 233

Fusarium_sp-PT_000311-RA TMHMM2.0 inside 234 279

Fusarium_sp-PT_000311-RA TMHMM2.0 TMhelix 280 302

Fusarium_sp-PT_000311-RA TMHMM2.0 outside 303 316

Fusarium_sp-PT_000311-RA TMHMM2.0 TMhelix 317 339

Fusarium_sp-PT_000311-RA TMHMM2.0 inside 340 359

# Fusarium_sp-PT_000343-RA Length: 485

# Fusarium_sp-PT_000343-RA Number of predicted TMHs: 2

# Fusarium_sp-PT_000343-RA Exp number of AAs in TMHs: 56.40755

# Fusarium_sp-PT_000343-RA Exp number, first 60 AAs: 0.00039

# Fusarium_sp-PT_000343-RA Total prob of N-in: 0.56603

Fusarium_sp-PT_000343-RA TMHMM2.0 inside 1 349

Fusarium_sp-PT_000343-RA TMHMM2.0 TMhelix 350 372

Fusarium_sp-PT_000343-RA TMHMM2.0 outside 373 391

Fusarium_sp-PT_000343-RA TMHMM2.0 TMhelix 392 414

Fusarium_sp-PT_000343-RA TMHMM2.0 inside 415 485

# Fusarium_sp-PT_000344-RA Length: 783

# Fusarium_sp-PT_000344-RA Number of predicted TMHs: 0

# Fusarium_sp-PT_000344-RA Exp number of AAs in TMHs: 0.918779999999999

# Fusarium_sp-PT_000344-RA Exp number, first 60 AAs: 0.89038

# Fusarium_sp-PT_000344-RA Total prob of N-in: 0.04336

Fusarium_sp-PT_000344-RA TMHMM2.0 outside 1 783

# Fusarium_sp-PT_000347-RA Length: 613

# Fusarium_sp-PT_000347-RA Number of predicted TMHs: 1

# Fusarium_sp-PT_000347-RA Exp number of AAs in TMHs: 23.61084

# Fusarium_sp-PT_000347-RA Exp number, first 60 AAs: 0.33027

# Fusarium_sp-PT_000347-RA Total prob of N-in: 0.01899

Fusarium_sp-PT_000347-RA TMHMM2.0 outside 1 467

Fusarium_sp-PT_000347-RA TMHMM2.0 TMhelix 468 490

Fusarium_sp-PT_000347-RA TMHMM2.0 inside 491 613

# Fusarium_sp-PT_000348-RA Length: 424

# Fusarium_sp-PT_000348-RA Number of predicted TMHs: 0

# Fusarium_sp-PT_000348-RA Exp number of AAs in TMHs: 0.00185

# Fusarium_sp-PT_000348-RA Exp number, first 60 AAs: 4e-05

# Fusarium_sp-PT_000348-RA Total prob of N-in: 0.00216

Fusarium_sp-PT_000348-RA TMHMM2.0 outside 1 424

# Fusarium_sp-PT_000352-RA Length: 352

# Fusarium_sp-PT_000352-RA Number of predicted TMHs: 0

# Fusarium_sp-PT_000352-RA Exp number of AAs in TMHs: 0.00966

# Fusarium_sp-PT_000352-RA Exp number, first 60 AAs: 0.00023

# Fusarium_sp-PT_000352-RA Total prob of N-in: 0.05711

Fusarium_sp-PT_000352-RA TMHMM2.0 outside 1 352

# Fusarium_sp-PT_000356-RA Length: 221

# Fusarium_sp-PT_000356-RA Number of predicted TMHs: 0

# Fusarium_sp-PT_000356-RA Exp number of AAs in TMHs: 0.0015

# Fusarium_sp-PT_000356-RA Exp number, first 60 AAs: 0

# Fusarium_sp-PT_000356-RA Total prob of N-in: 0.42116

Fusarium_sp-PT_000356-RA TMHMM2.0 outside 1 221

# Fusarium_sp-PT_000357-RA Length: 568

# Fusarium_sp-PT_000357-RA Number of predicted TMHs: 0

# Fusarium_sp-PT_000357-RA Exp number of AAs in TMHs: 0.00227

# Fusarium_sp-PT_000357-RA Exp number, first 60 AAs: 0

# Fusarium_sp-PT_000357-RA Total prob of N-in: 0.00139

Fusarium_sp-PT_000357-RA TMHMM2.0 outside 1 568

# Fusarium_sp-PT_000358-RA Length: 940

# Fusarium_sp-PT_000358-RA Number of predicted TMHs: 0

# Fusarium_sp-PT_000358-RA Exp number of AAs in TMHs: 0.0037

# Fusarium_sp-PT_000358-RA Exp number, first 60 AAs: 0.00157

# Fusarium_sp-PT_000358-RA Total prob of N-in: 0.00018

Fusarium_sp-PT_000358-RA TMHMM2.0 outside 1 940

# Fusarium_sp-PT_000359-RA Length: 401

# Fusarium_sp-PT_000359-RA Number of predicted TMHs: 0

# Fusarium_sp-PT_000359-RA Exp number of AAs in TMHs: 0.04282

# Fusarium_sp-PT_000359-RA Exp number, first 60 AAs: 0.0215

# Fusarium_sp-PT_000359-RA Total prob of N-in: 0.00564

Fusarium_sp-PT_000359-RA TMHMM2.0 outside 1 401

# Fusarium_sp-PT_000362-RA Length: 317

# Fusarium_sp-PT_000362-RA Number of predicted TMHs: 0

# Fusarium_sp-PT_000362-RA Exp number of AAs in TMHs: 0.00275

# Fusarium_sp-PT_000362-RA Exp number, first 60 AAs: 0.00036

# Fusarium_sp-PT_000362-RA Total prob of N-in: 0.01842

Fusarium_sp-PT_000362-RA TMHMM2.0 outside 1 317

# Fusarium_sp-PT_000364-RA Length: 149

# Fusarium_sp-PT_000364-RA Number of predicted TMHs: 4

# Fusarium_sp-PT_000364-RA Exp number of AAs in TMHs: 75.1493

# Fusarium_sp-PT_000364-RA Exp number, first 60 AAs: 21.41283

# Fusarium_sp-PT_000364-RA Total prob of N-in: 0.81413

# Fusarium_sp-PT_000364-RA POSSIBLE N-term signal sequence

Fusarium_sp-PT_000364-RA TMHMM2.0 inside 1 8

Fusarium_sp-PT_000364-RA TMHMM2.0 TMhelix 9 31

Fusarium_sp-PT_000364-RA TMHMM2.0 outside 32 74

Fusarium_sp-PT_000364-RA TMHMM2.0 TMhelix 75 94

Fusarium_sp-PT_000364-RA TMHMM2.0 inside 95 98

Fusarium_sp-PT_000364-RA TMHMM2.0 TMhelix 99 121

Fusarium_sp-PT_000364-RA TMHMM2.0 outside 122 130

Fusarium_sp-PT_000364-RA TMHMM2.0 TMhelix 131 145

Fusarium_sp-PT_000364-RA TMHMM2.0 inside 146 149

# Fusarium_sp-PT_000367-RA Length: 394

# Fusarium_sp-PT_000367-RA Number of predicted TMHs: 0

# Fusarium_sp-PT_000367-RA Exp number of AAs in TMHs: 0

# Fusarium_sp-PT_000367-RA Exp number, first 60 AAs: 0

# Fusarium_sp-PT_000367-RA Total prob of N-in: 0.01290

Fusarium_sp-PT_000367-RA TMHMM2.0 outside 1 394

# Fusarium_sp-PT_000374-RA Length: 502

# Fusarium_sp-PT_000374-RA Number of predicted TMHs: 0

# Fusarium_sp-PT_000374-RA Exp number of AAs in TMHs: 0.0144

# Fusarium_sp-PT_000374-RA Exp number, first 60 AAs: 0.01072

# Fusarium_sp-PT_000374-RA Total prob of N-in: 0.00310

Fusarium_sp-PT_000374-RA TMHMM2.0 outside 1 502

# Fusarium_sp-PT_000345-RA Length: 290

# Fusarium_sp-PT_000345-RA Number of predicted TMHs: 0

# Fusarium_sp-PT_000345-RA Exp number of AAs in TMHs: 0.04919

# Fusarium_sp-PT_000345-RA Exp number, first 60 AAs: 0.04835

# Fusarium_sp-PT_000345-RA Total prob of N-in: 0.01316

Fusarium_sp-PT_000345-RA TMHMM2.0 outside 1 290

# Fusarium_sp-PT_000346-RA Length: 249

# Fusarium_sp-PT_000346-RA Number of predicted TMHs: 0

# Fusarium_sp-PT_000346-RA Exp number of AAs in TMHs: 8.32318999999999

# Fusarium_sp-PT_000346-RA Exp number, first 60 AAs: 0.00029

# Fusarium_sp-PT_000346-RA Total prob of N-in: 0.01203

Fusarium_sp-PT_000346-RA TMHMM2.0 outside 1 249

# Fusarium_sp-PT_000349-RA Length: 796

# Fusarium_sp-PT_000349-RA Number of predicted TMHs: 0

# Fusarium_sp-PT_000349-RA Exp number of AAs in TMHs: 0.06453

# Fusarium_sp-PT_000349-RA Exp number, first 60 AAs: 0

# Fusarium_sp-PT_000349-RA Total prob of N-in: 0.00005

Fusarium_sp-PT_000349-RA TMHMM2.0 outside 1 796

# Fusarium_sp-PT_000350-RA Length: 350

# Fusarium_sp-PT_000350-RA Number of predicted TMHs: 0

# Fusarium_sp-PT_000350-RA Exp number of AAs in TMHs: 0.00037

# Fusarium_sp-PT_000350-RA Exp number, first 60 AAs: 0

# Fusarium_sp-PT_000350-RA Total prob of N-in: 0.03510

Fusarium_sp-PT_000350-RA TMHMM2.0 outside 1 350

# Fusarium_sp-PT_000351-RA Length: 930

# Fusarium_sp-PT_000351-RA Number of predicted TMHs: 0

# Fusarium_sp-PT_000351-RA Exp number of AAs in TMHs: 0.00176

# Fusarium_sp-PT_000351-RA Exp number, first 60 AAs: 0.00037

# Fusarium_sp-PT_000351-RA Total prob of N-in: 0.00007

Fusarium_sp-PT_000351-RA TMHMM2.0 outside 1 930

# Fusarium_sp-PT_000353-RA Length: 820

# Fusarium_sp-PT_000353-RA Number of predicted TMHs: 1

# Fusarium_sp-PT_000353-RA Exp number of AAs in TMHs: 22.70808

# Fusarium_sp-PT_000353-RA Exp number, first 60 AAs: 0

# Fusarium_sp-PT_000353-RA Total prob of N-in: 0.77430

Fusarium_sp-PT_000353-RA TMHMM2.0 inside 1 597

Fusarium_sp-PT_000353-RA TMHMM2.0 TMhelix 598 620

Fusarium_sp-PT_000353-RA TMHMM2.0 outside 621 820

# Fusarium_sp-PT_000354-RA Length: 625

# Fusarium_sp-PT_000354-RA Number of predicted TMHs: 0

# Fusarium_sp-PT_000354-RA Exp number of AAs in TMHs: 0

# Fusarium_sp-PT_000354-RA Exp number, first 60 AAs: 0

# Fusarium_sp-PT_000354-RA Total prob of N-in: 0.00065

Fusarium_sp-PT_000354-RA TMHMM2.0 outside 1 625

# Fusarium_sp-PT_000355-RA Length: 110

# Fusarium_sp-PT_000355-RA Number of predicted TMHs: 0

# Fusarium_sp-PT_000355-RA Exp number of AAs in TMHs: 0.01187

# Fusarium_sp-PT_000355-RA Exp number, first 60 AAs: 0.00717

# Fusarium_sp-PT_000355-RA Total prob of N-in: 0.62683

Fusarium_sp-PT_000355-RA TMHMM2.0 inside 1 110

# Fusarium_sp-PT_000360-RA Length: 301

# Fusarium_sp-PT_000360-RA Number of predicted TMHs: 0

# Fusarium_sp-PT_000360-RA Exp number of AAs in TMHs: 0.08913

# Fusarium_sp-PT_000360-RA Exp number, first 60 AAs: 0.00045

# Fusarium_sp-PT_000360-RA Total prob of N-in: 0.02778

Fusarium_sp-PT_000360-RA TMHMM2.0 outside 1 301

# Fusarium_sp-PT_000361-RA Length: 91

# Fusarium_sp-PT_000361-RA Number of predicted TMHs: 0

# Fusarium_sp-PT_000361-RA Exp number of AAs in TMHs: 0.04758

# Fusarium_sp-PT_000361-RA Exp number, first 60 AAs: 0.04742

# Fusarium_sp-PT_000361-RA Total prob of N-in: 0.07159

Fusarium_sp-PT_000361-RA TMHMM2.0 outside 1 91

# Fusarium_sp-PT_000363-RA Length: 84

# Fusarium_sp-PT_000363-RA Number of predicted TMHs: 0

# Fusarium_sp-PT_000363-RA Exp number of AAs in TMHs: 0

# Fusarium_sp-PT_000363-RA Exp number, first 60 AAs: 0

# Fusarium_sp-PT_000363-RA Total prob of N-in: 0.30921

Fusarium_sp-PT_000363-RA TMHMM2.0 outside 1 84

# Fusarium_sp-PT_000365-RA Length: 180

# Fusarium_sp-PT_000365-RA Number of predicted TMHs: 0

# Fusarium_sp-PT_000365-RA Exp number of AAs in TMHs: 0.00034

# Fusarium_sp-PT_000365-RA Exp number, first 60 AAs: 0.00034

# Fusarium_sp-PT_000365-RA Total prob of N-in: 0.09694

Fusarium_sp-PT_000365-RA TMHMM2.0 outside 1 180

# Fusarium_sp-PT_000366-RA Length: 253

# Fusarium_sp-PT_000366-RA Number of predicted TMHs: 0

# Fusarium_sp-PT_000366-RA Exp number of AAs in TMHs: 0.37281

# Fusarium_sp-PT_000366-RA Exp number, first 60 AAs: 0.10965

# Fusarium_sp-PT_000366-RA Total prob of N-in: 0.08782

Fusarium_sp-PT_000366-RA TMHMM2.0 outside 1 253

# Fusarium_sp-PT_000368-RA Length: 227

# Fusarium_sp-PT_000368-RA Number of predicted TMHs: 0

# Fusarium_sp-PT_000368-RA Exp number of AAs in TMHs: 0.013

# Fusarium_sp-PT_000368-RA Exp number, first 60 AAs: 0.01241

# Fusarium_sp-PT_000368-RA Total prob of N-in: 0.04951

Fusarium_sp-PT_000368-RA TMHMM2.0 outside 1 227

# Fusarium_sp-PT_000369-RA Length: 198

# Fusarium_sp-PT_000369-RA Number of predicted TMHs: 4

# Fusarium_sp-PT_000369-RA Exp number of AAs in TMHs: 91.0156199999999

# Fusarium_sp-PT_000369-RA Exp number, first 60 AAs: 22.81422

# Fusarium_sp-PT_000369-RA Total prob of N-in: 0.71926

# Fusarium_sp-PT_000369-RA POSSIBLE N-term signal sequence

Fusarium_sp-PT_000369-RA TMHMM2.0 inside 1 4

Fusarium_sp-PT_000369-RA TMHMM2.0 TMhelix 5 27

Fusarium_sp-PT_000369-RA TMHMM2.0 outside 28 83

Fusarium_sp-PT_000369-RA TMHMM2.0 TMhelix 84 106

Fusarium_sp-PT_000369-RA TMHMM2.0 inside 107 118

Fusarium_sp-PT_000369-RA TMHMM2.0 TMhelix 119 141

Fusarium_sp-PT_000369-RA TMHMM2.0 outside 142 160

Fusarium_sp-PT_000369-RA TMHMM2.0 TMhelix 161 183

Fusarium_sp-PT_000369-RA TMHMM2.0 inside 184 198

# Fusarium_sp-PT_000370-RA Length: 447

# Fusarium_sp-PT_000370-RA Number of predicted TMHs: 0

# Fusarium_sp-PT_000370-RA Exp number of AAs in TMHs: 0.306410000000001

# Fusarium_sp-PT_000370-RA Exp number, first 60 AAs: 0.00065

# Fusarium_sp-PT_000370-RA Total prob of N-in: 0.04632

Fusarium_sp-PT_000370-RA TMHMM2.0 outside 1 447

# Fusarium_sp-PT_000371-RA Length: 624

# Fusarium_sp-PT_000371-RA Number of predicted TMHs: 0

# Fusarium_sp-PT_000371-RA Exp number of AAs in TMHs: 0.000740000000000001

# Fusarium_sp-PT_000371-RA Exp number, first 60 AAs: 0.00055

# Fusarium_sp-PT_000371-RA Total prob of N-in: 0.00177

Fusarium_sp-PT_000371-RA TMHMM2.0 outside 1 624

# Fusarium_sp-PT_000372-RA Length: 312

# Fusarium_sp-PT_000372-RA Number of predicted TMHs: 0

# Fusarium_sp-PT_000372-RA Exp number of AAs in TMHs: 0.000580000000000001

# Fusarium_sp-PT_000372-RA Exp number, first 60 AAs: 0.0004

# Fusarium_sp-PT_000372-RA Total prob of N-in: 0.01238

Fusarium_sp-PT_000372-RA TMHMM2.0 outside 1 312

# Fusarium_sp-PT_000373-RA Length: 434

# Fusarium_sp-PT_000373-RA Number of predicted TMHs: 0

# Fusarium_sp-PT_000373-RA Exp number of AAs in TMHs: 0.01344

# Fusarium_sp-PT_000373-RA Exp number, first 60 AAs: 0

# Fusarium_sp-PT_000373-RA Total prob of N-in: 0.00207

Fusarium_sp-PT_000373-RA TMHMM2.0 outside 1 434

# Fusarium_sp-PT_000375-RA Length: 180

# Fusarium_sp-PT_000375-RA Number of predicted TMHs: 0

# Fusarium_sp-PT_000375-RA Exp number of AAs in TMHs: 0.00099

# Fusarium_sp-PT_000375-RA Exp number, first 60 AAs: 0.00091

# Fusarium_sp-PT_000375-RA Total prob of N-in: 0.12124

Fusarium_sp-PT_000375-RA TMHMM2.0 outside 1 180

# Fusarium_sp-PT_000376-RA Length: 582

# Fusarium_sp-PT_000376-RA Number of predicted TMHs: 8

# Fusarium_sp-PT_000376-RA Exp number of AAs in TMHs: 179.35544

# Fusarium_sp-PT_000376-RA Exp number, first 60 AAs: 0

# Fusarium_sp-PT_000376-RA Total prob of N-in: 0.95538

Fusarium_sp-PT_000376-RA TMHMM2.0 inside 1 149

Fusarium_sp-PT_000376-RA TMHMM2.0 TMhelix 150 172

Fusarium_sp-PT_000376-RA TMHMM2.0 outside 173 211

Fusarium_sp-PT_000376-RA TMHMM2.0 TMhelix 212 234

Fusarium_sp-PT_000376-RA TMHMM2.0 inside 235 246

Fusarium_sp-PT_000376-RA TMHMM2.0 TMhelix 247 269

Fusarium_sp-PT_000376-RA TMHMM2.0 outside 270 283

Fusarium_sp-PT_000376-RA TMHMM2.0 TMhelix 284 306

Fusarium_sp-PT_000376-RA TMHMM2.0 inside 307 339

Fusarium_sp-PT_000376-RA TMHMM2.0 TMhelix 340 359

Fusarium_sp-PT_000376-RA TMHMM2.0 outside 360 407

Fusarium_sp-PT_000376-RA TMHMM2.0 TMhelix 408 430

Fusarium_sp-PT_000376-RA TMHMM2.0 inside 431 436

Fusarium_sp-PT_000376-RA TMHMM2.0 TMhelix 437 456

Fusarium_sp-PT_000376-RA TMHMM2.0 outside 457 520

Fusarium_sp-PT_000376-RA TMHMM2.0 TMhelix 521 543

Fusarium_sp-PT_000376-RA TMHMM2.0 inside 544 582

# Fusarium_sp-PT_000378-RA Length: 124

# Fusarium_sp-PT_000378-RA Number of predicted TMHs: 0

# Fusarium_sp-PT_000378-RA Exp number of AAs in TMHs: 0.10717

# Fusarium_sp-PT_000378-RA Exp number, first 60 AAs: 0.06419

# Fusarium_sp-PT_000378-RA Total prob of N-in: 0.08685

Fusarium_sp-PT_000378-RA TMHMM2.0 outside 1 124

# Fusarium_sp-PT_000379-RA Length: 942

# Fusarium_sp-PT_000379-RA Number of predicted TMHs: 0

# Fusarium_sp-PT_000379-RA Exp number of AAs in TMHs: 0.16235

# Fusarium_sp-PT_000379-RA Exp number, first 60 AAs: 0.02987

# Fusarium_sp-PT_000379-RA Total prob of N-in: 0.00765

Fusarium_sp-PT_000379-RA TMHMM2.0 outside 1 942

# Fusarium_sp-PT_000381-RA Length: 682

# Fusarium_sp-PT_000381-RA Number of predicted TMHs: 0

# Fusarium_sp-PT_000381-RA Exp number of AAs in TMHs: 5.32008

# Fusarium_sp-PT_000381-RA Exp number, first 60 AAs: 0.19226

# Fusarium_sp-PT_000381-RA Total prob of N-in: 0.09802

Fusarium_sp-PT_000381-RA TMHMM2.0 outside 1 682

# Fusarium_sp-PT_000383-RA Length: 737

# Fusarium_sp-PT_000383-RA Number of predicted TMHs: 0

# Fusarium_sp-PT_000383-RA Exp number of AAs in TMHs: 0.00761999999999999

# Fusarium_sp-PT_000383-RA Exp number, first 60 AAs: 0

# Fusarium_sp-PT_000383-RA Total prob of N-in: 0.00095

Fusarium_sp-PT_000383-RA TMHMM2.0 outside 1 737

# Fusarium_sp-PT_000384-RA Length: 870

# Fusarium_sp-PT_000384-RA Number of predicted TMHs: 0

# Fusarium_sp-PT_000384-RA Exp number of AAs in TMHs: 0.000990000000000001

# Fusarium_sp-PT_000384-RA Exp number, first 60 AAs: 0.00018

# Fusarium_sp-PT_000384-RA Total prob of N-in: 0.00004

Fusarium_sp-PT_000384-RA TMHMM2.0 outside 1 870

# Fusarium_sp-PT_000385-RA Length: 1222

# Fusarium_sp-PT_000385-RA Number of predicted TMHs: 0

# Fusarium_sp-PT_000385-RA Exp number of AAs in TMHs: 0.000890000000000001

# Fusarium_sp-PT_000385-RA Exp number, first 60 AAs: 0

# Fusarium_sp-PT_000385-RA Total prob of N-in: 0.00000

Fusarium_sp-PT_000385-RA TMHMM2.0 outside 1 1222

# Fusarium_sp-PT_000386-RA Length: 461

# Fusarium_sp-PT_000386-RA Number of predicted TMHs: 0

# Fusarium_sp-PT_000386-RA Exp number of AAs in TMHs: 0.576270000000001

# Fusarium_sp-PT_000386-RA Exp number, first 60 AAs: 0

# Fusarium_sp-PT_000386-RA Total prob of N-in: 0.01419

Fusarium_sp-PT_000386-RA TMHMM2.0 outside 1 461

# Fusarium_sp-PT_000387-RA Length: 82

# Fusarium_sp-PT_000387-RA Number of predicted TMHs: 0

# Fusarium_sp-PT_000387-RA Exp number of AAs in TMHs: 0

# Fusarium_sp-PT_000387-RA Exp number, first 60 AAs: 0

# Fusarium_sp-PT_000387-RA Total prob of N-in: 0.67305

Fusarium_sp-PT_000387-RA TMHMM2.0 inside 1 82

# Fusarium_sp-PT_000390-RA Length: 151

# Fusarium_sp-PT_000390-RA Number of predicted TMHs: 0

# Fusarium_sp-PT_000390-RA Exp number of AAs in TMHs: 0

# Fusarium_sp-PT_000390-RA Exp number, first 60 AAs: 0

# Fusarium_sp-PT_000390-RA Total prob of N-in: 0.47426

Fusarium_sp-PT_000390-RA TMHMM2.0 outside 1 151

# Fusarium_sp-PT_000395-RA Length: 549

# Fusarium_sp-PT_000395-RA Number of predicted TMHs: 0

# Fusarium_sp-PT_000395-RA Exp number of AAs in TMHs: 0.00122

# Fusarium_sp-PT_000395-RA Exp number, first 60 AAs: 0.00014

# Fusarium_sp-PT_000395-RA Total prob of N-in: 0.00048

Fusarium_sp-PT_000395-RA TMHMM2.0 outside 1 549

# Fusarium_sp-PT_000377-RA Length: 161

# Fusarium_sp-PT_000377-RA Number of predicted TMHs: 4

# Fusarium_sp-PT_000377-RA Exp number of AAs in TMHs: 91.37552

# Fusarium_sp-PT_000377-RA Exp number, first 60 AAs: 29.72341

# Fusarium_sp-PT_000377-RA Total prob of N-in: 0.29264

# Fusarium_sp-PT_000377-RA POSSIBLE N-term signal sequence

Fusarium_sp-PT_000377-RA TMHMM2.0 inside 1 11

Fusarium_sp-PT_000377-RA TMHMM2.0 TMhelix 12 34

Fusarium_sp-PT_000377-RA TMHMM2.0 outside 35 53

Fusarium_sp-PT_000377-RA TMHMM2.0 TMhelix 54 76

Fusarium_sp-PT_000377-RA TMHMM2.0 inside 77 88

Fusarium_sp-PT_000377-RA TMHMM2.0 TMhelix 89 111

Fusarium_sp-PT_000377-RA TMHMM2.0 outside 112 125

Fusarium_sp-PT_000377-RA TMHMM2.0 TMhelix 126 148

Fusarium_sp-PT_000377-RA TMHMM2.0 inside 149 161

# Fusarium_sp-PT_000380-RA Length: 404

# Fusarium_sp-PT_000380-RA Number of predicted TMHs: 0

# Fusarium_sp-PT_000380-RA Exp number of AAs in TMHs: 0.20735

# Fusarium_sp-PT_000380-RA Exp number, first 60 AAs: 0

# Fusarium_sp-PT_000380-RA Total prob of N-in: 0.02721

Fusarium_sp-PT_000380-RA TMHMM2.0 outside 1 404

# Fusarium_sp-PT_000382-RA Length: 182

# Fusarium_sp-PT_000382-RA Number of predicted TMHs: 0

# Fusarium_sp-PT_000382-RA Exp number of AAs in TMHs: 0.00385

# Fusarium_sp-PT_000382-RA Exp number, first 60 AAs: 0.00351

# Fusarium_sp-PT_000382-RA Total prob of N-in: 0.04007

Fusarium_sp-PT_000382-RA TMHMM2.0 outside 1 182

# Fusarium_sp-PT_000388-RA Length: 482

# Fusarium_sp-PT_000388-RA Number of predicted TMHs: 0

# Fusarium_sp-PT_000388-RA Exp number of AAs in TMHs: 0

# Fusarium_sp-PT_000388-RA Exp number, first 60 AAs: 0

# Fusarium_sp-PT_000388-RA Total prob of N-in: 0.00527

Fusarium_sp-PT_000388-RA TMHMM2.0 outside 1 482

# Fusarium_sp-PT_000389-RA Length: 717

# Fusarium_sp-PT_000389-RA Number of predicted TMHs: 0

# Fusarium_sp-PT_000389-RA Exp number of AAs in TMHs: 0.85982

# Fusarium_sp-PT_000389-RA Exp number, first 60 AAs: 0

# Fusarium_sp-PT_000389-RA Total prob of N-in: 0.00019

Fusarium_sp-PT_000389-RA TMHMM2.0 outside 1 717

# Fusarium_sp-PT_000391-RA Length: 349

# Fusarium_sp-PT_000391-RA Number of predicted TMHs: 0

# Fusarium_sp-PT_000391-RA Exp number of AAs in TMHs: 0.05022

# Fusarium_sp-PT_000391-RA Exp number, first 60 AAs: 0.00096

# Fusarium_sp-PT_000391-RA Total prob of N-in: 0.05344

Fusarium_sp-PT_000391-RA TMHMM2.0 outside 1 349

# Fusarium_sp-PT_000392-RA Length: 354

# Fusarium_sp-PT_000392-RA Number of predicted TMHs: 0

# Fusarium_sp-PT_000392-RA Exp number of AAs in TMHs: 0.1021

# Fusarium_sp-PT_000392-RA Exp number, first 60 AAs: 0.0002

# Fusarium_sp-PT_000392-RA Total prob of N-in: 0.02050

Fusarium_sp-PT_000392-RA TMHMM2.0 outside 1 354

# Fusarium_sp-PT_000393-RA Length: 499

# Fusarium_sp-PT_000393-RA Number of predicted TMHs: 1

# Fusarium_sp-PT_000393-RA Exp number of AAs in TMHs: 19.72312

# Fusarium_sp-PT_000393-RA Exp number, first 60 AAs: 19.60433

# Fusarium_sp-PT_000393-RA Total prob of N-in: 0.99568

# Fusarium_sp-PT_000393-RA POSSIBLE N-term signal sequence

Fusarium_sp-PT_000393-RA TMHMM2.0 inside 1 6

Fusarium_sp-PT_000393-RA TMHMM2.0 TMhelix 7 24

Fusarium_sp-PT_000393-RA TMHMM2.0 outside 25 499

# Fusarium_sp-PT_000394-RA Length: 398

# Fusarium_sp-PT_000394-RA Number of predicted TMHs: 0

# Fusarium_sp-PT_000394-RA Exp number of AAs in TMHs: 0.00096

# Fusarium_sp-PT_000394-RA Exp number, first 60 AAs: 0

# Fusarium_sp-PT_000394-RA Total prob of N-in: 0.00577

Fusarium_sp-PT_000394-RA TMHMM2.0 outside 1 398

# Fusarium_sp-PT_000396-RA Length: 277

# Fusarium_sp-PT_000396-RA Number of predicted TMHs: 0

# Fusarium_sp-PT_000396-RA Exp number of AAs in TMHs: 0.00357000000000001

# Fusarium_sp-PT_000396-RA Exp number, first 60 AAs: 0.0016

# Fusarium_sp-PT_000396-RA Total prob of N-in: 0.06841

Fusarium_sp-PT_000396-RA TMHMM2.0 outside 1 277

# Fusarium_sp-PT_000397-RA Length: 735

# Fusarium_sp-PT_000397-RA Number of predicted TMHs: 0

# Fusarium_sp-PT_000397-RA Exp number of AAs in TMHs: 0.603850000000001

# Fusarium_sp-PT_000397-RA Exp number, first 60 AAs: 0.59914

# Fusarium_sp-PT_000397-RA Total prob of N-in: 0.03246

Fusarium_sp-PT_000397-RA TMHMM2.0 outside 1 735

# Fusarium_sp-PT_000398-RA Length: 983

# Fusarium_sp-PT_000398-RA Number of predicted TMHs: 0

# Fusarium_sp-PT_000398-RA Exp number of AAs in TMHs: 0.01168

# Fusarium_sp-PT_000398-RA Exp number, first 60 AAs: 0

# Fusarium_sp-PT_000398-RA Total prob of N-in: 0.00052

Fusarium_sp-PT_000398-RA TMHMM2.0 outside 1 983

# Fusarium_sp-PT_000399-RA Length: 450

# Fusarium_sp-PT_000399-RA Number of predicted TMHs: 0

# Fusarium_sp-PT_000399-RA Exp number of AAs in TMHs: 14.94948

# Fusarium_sp-PT_000399-RA Exp number, first 60 AAs: 0.0758299999999999

# Fusarium_sp-PT_000399-RA Total prob of N-in: 0.03156

Fusarium_sp-PT_000399-RA TMHMM2.0 outside 1 450

# Fusarium_sp-PT_000400-RA Length: 285

# Fusarium_sp-PT_000400-RA Number of predicted TMHs: 0

# Fusarium_sp-PT_000400-RA Exp number of AAs in TMHs: 0.19714

# Fusarium_sp-PT_000400-RA Exp number, first 60 AAs: 0.01163

# Fusarium_sp-PT_000400-RA Total prob of N-in: 0.01928

Fusarium_sp-PT_000400-RA TMHMM2.0 outside 1 285

# Fusarium_sp-PT_000402-RA Length: 140

# Fusarium_sp-PT_000402-RA Number of predicted TMHs: 1

# Fusarium_sp-PT_000402-RA Exp number of AAs in TMHs: 22.58936

# Fusarium_sp-PT_000402-RA Exp number, first 60 AAs: 22.58936

# Fusarium_sp-PT_000402-RA Total prob of N-in: 0.00112

# Fusarium_sp-PT_000402-RA POSSIBLE N-term signal sequence

Fusarium_sp-PT_000402-RA TMHMM2.0 outside 1 19

Fusarium_sp-PT_000402-RA TMHMM2.0 TMhelix 20 42

Fusarium_sp-PT_000402-RA TMHMM2.0 inside 43 140

# Fusarium_sp-PT_000403-RA Length: 1668

# Fusarium_sp-PT_000403-RA Number of predicted TMHs: 0

# Fusarium_sp-PT_000403-RA Exp number of AAs in TMHs: 0.000550000000000001

# Fusarium_sp-PT_000403-RA Exp number, first 60 AAs: 0

# Fusarium_sp-PT_000403-RA Total prob of N-in: 0.00003

Fusarium_sp-PT_000403-RA TMHMM2.0 outside 1 1668

# Fusarium_sp-PT_000404-RA Length: 337

# Fusarium_sp-PT_000404-RA Number of predicted TMHs: 1

# Fusarium_sp-PT_000404-RA Exp number of AAs in TMHs: 79.19643

# Fusarium_sp-PT_000404-RA Exp number, first 60 AAs: 0.54689

# Fusarium_sp-PT_000404-RA Total prob of N-in: 0.68547

Fusarium_sp-PT_000404-RA TMHMM2.0 inside 1 206

Fusarium_sp-PT_000404-RA TMHMM2.0 TMhelix 207 229

Fusarium_sp-PT_000404-RA TMHMM2.0 outside 230 337

# Fusarium_sp-PT_000408-RA Length: 486

# Fusarium_sp-PT_000408-RA Number of predicted TMHs: 0

# Fusarium_sp-PT_000408-RA Exp number of AAs in TMHs: 0.00018

# Fusarium_sp-PT_000408-RA Exp number, first 60 AAs: 0

# Fusarium_sp-PT_000408-RA Total prob of N-in: 0.00029

Fusarium_sp-PT_000408-RA TMHMM2.0 outside 1 486

# Fusarium_sp-PT_000411-RA Length: 404

# Fusarium_sp-PT_000411-RA Number of predicted TMHs: 0

# Fusarium_sp-PT_000411-RA Exp number of AAs in TMHs: 12.19266

# Fusarium_sp-PT_000411-RA Exp number, first 60 AAs: 12.17191

# Fusarium_sp-PT_000411-RA Total prob of N-in: 0.54306

# Fusarium_sp-PT_000411-RA POSSIBLE N-term signal sequence

Fusarium_sp-PT_000411-RA TMHMM2.0 outside 1 404

# Fusarium_sp-PT_000412-RA Length: 223

# Fusarium_sp-PT_000412-RA Number of predicted TMHs: 0

# Fusarium_sp-PT_000412-RA Exp number of AAs in TMHs: 0

# Fusarium_sp-PT_000412-RA Exp number, first 60 AAs: 0

# Fusarium_sp-PT_000412-RA Total prob of N-in: 0.02898

Fusarium_sp-PT_000412-RA TMHMM2.0 outside 1 223

# Fusarium_sp-PT_000413-RA Length: 1016

# Fusarium_sp-PT_000413-RA Number of predicted TMHs: 3

# Fusarium_sp-PT_000413-RA Exp number of AAs in TMHs: 65.62178

# Fusarium_sp-PT_000413-RA Exp number, first 60 AAs: 21.49355

# Fusarium_sp-PT_000413-RA Total prob of N-in: 0.99299

# Fusarium_sp-PT_000413-RA POSSIBLE N-term signal sequence

Fusarium_sp-PT_000413-RA TMHMM2.0 inside 1 6

Fusarium_sp-PT_000413-RA TMHMM2.0 TMhelix 7 29

Fusarium_sp-PT_000413-RA TMHMM2.0 outside 30 124

Fusarium_sp-PT_000413-RA TMHMM2.0 TMhelix 125 147

Fusarium_sp-PT_000413-RA TMHMM2.0 inside 148 273

Fusarium_sp-PT_000413-RA TMHMM2.0 TMhelix 274 296

Fusarium_sp-PT_000413-RA TMHMM2.0 outside 297 1016

# Fusarium_sp-PT_000416-RA Length: 489

# Fusarium_sp-PT_000416-RA Number of predicted TMHs: 3

# Fusarium_sp-PT_000416-RA Exp number of AAs in TMHs: 66.27351

# Fusarium_sp-PT_000416-RA Exp number, first 60 AAs: 0

# Fusarium_sp-PT_000416-RA Total prob of N-in: 0.71454

Fusarium_sp-PT_000416-RA TMHMM2.0 inside 1 154

Fusarium_sp-PT_000416-RA TMHMM2.0 TMhelix 155 177

Fusarium_sp-PT_000416-RA TMHMM2.0 outside 178 252

Fusarium_sp-PT_000416-RA TMHMM2.0 TMhelix 253 275

Fusarium_sp-PT_000416-RA TMHMM2.0 inside 276 279

Fusarium_sp-PT_000416-RA TMHMM2.0 TMhelix 280 302

Fusarium_sp-PT_000416-RA TMHMM2.0 outside 303 489

# Fusarium_sp-PT_000418-RA Length: 530

# Fusarium_sp-PT_000418-RA Number of predicted TMHs: 12
[truncated: 4,771,944 more chars]
