# Supplementary figures and images for: Draft Genome Sequence of a New Fusarium Isolate Belonging to Fusarium tricinctum Species Complex Collected From Hazelnut in Central Italy
Source: Front Plant Sci. 2021 Dec 16;12:788584. doi: 10.3389/fpls.2021.788584 (PMC8718101; doi:10.3389/fpls.2021.788584)

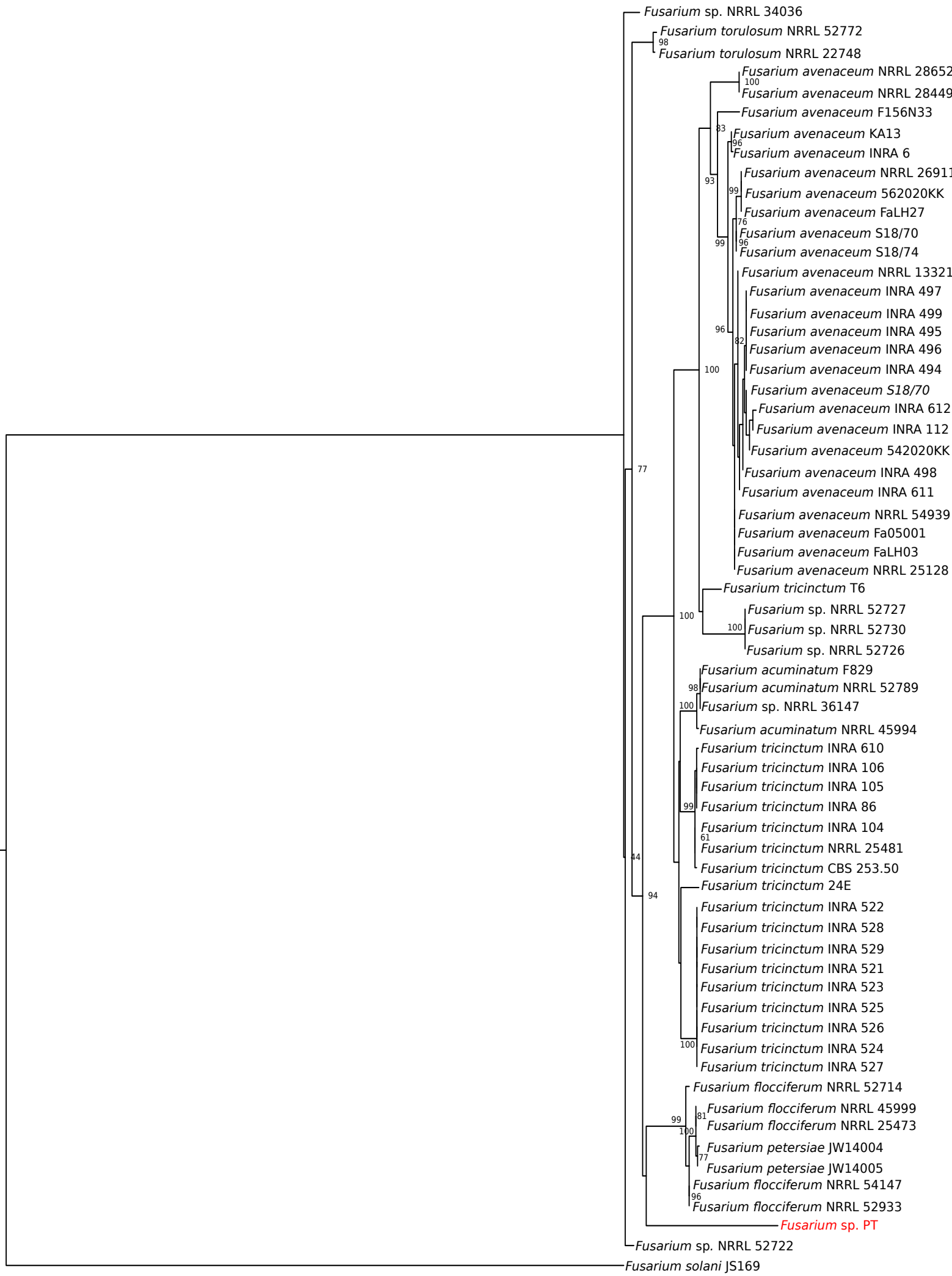

0.06

Supplement: Supplementary Figure 1 — Phylogenetic tree of RPB1 and RPB2 concatenated sequences among FTSC strains. The nucleotide sequence of RPB1 and RPB2 genes of a selection of 63 strains belonging to the FTSC were concatenated, aligned, and used to build a ML tree using RAxML, from which only bootstraps higher than 60 are shown. [file Data_Sheet_1.zip › Supplementary Figure 1.PDF]

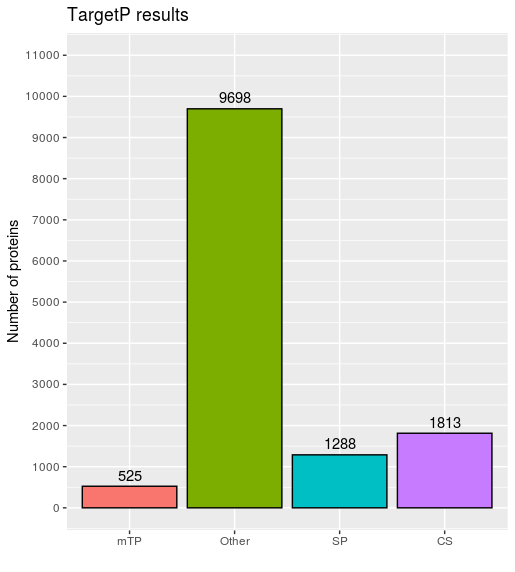

Supplement: Supplementary Figure 1 — Phylogenetic tree of RPB1 and RPB2 concatenated sequences among FTSC strains. The nucleotide sequence of RPB1 and RPB2 genes of a selection of 63 strains belonging to the FTSC were concatenated, aligned, and used to build a ML tree using RAxML, from which only bootstraps higher than 60 are shown. [file Data_Sheet_1.zip › Supplementary Figure 2.TIFF]
